# Proteomic Identification of the Galectin-1-Involved Molecular Pathways in Urinary Bladder Urothelial Carcinoma

**DOI:** 10.3390/ijms19041242

**Published:** 2018-04-19

**Authors:** Chien-Feng Li, Kun-Hung Shen, Lan-Hsiang Chien, Cheng-Hao Huang, Ting-Feng Wu, Hong-Lin He

**Affiliations:** 1 Department of Biotechnology, Southern Taiwan University of Science and Technology, Tainan 710, Taiwan; angelo.p@yahoo.com.tw (C.-F.L.); plane32033@gmail.com (C.-H.H.); 2 Departments of Pathology, Chi Mei Medical Center, Tainan 710, Taiwan; baltic1023@gmail.com; 3 National Institute of Cancer Research, National Health Research Institutes, Miaoli 350, Taiwan; 4 Department of Urology, Chi Mei Medical Center, Tainan 710, Taiwan; robert.shen@msa.hinet.net; 5 Department of Medical Research, Chi Mei Medical Center, Tainan 710, Taiwan; m96h0207@stust.edu.tw

**Keywords:** urinary bladder urothelial carcinoma, galectin-1, fatty acid binding protein 4, glutamine synthetase, two-dimensional gel electrophoresis

## Abstract

Among various heterogeneous types of bladder tumors, urothelial carcinoma is the most prevalent lesion. Some of the urinary bladder urothelial carcinomas (UBUCs) develop local recurrence and may cause distal invasion. Galectin-1 de-regulation significantly affects cell transformation, cell proliferation, angiogenesis, and cell invasiveness. In continuation of our previous investigation on the role of galectin-1 in UBUC tumorigenesis, in this study, proteomics strategies were implemented in order to find more galectin-1-associated signaling pathways. The results of this study showed that galectin-1 knockdown could induce 15 down-regulated proteins and two up-regulated proteins in T24 cells. These de-regulated proteins might participate in lipid/amino acid/energy metabolism, cytoskeleton, cell proliferation, cell-cell interaction, cell apoptosis, metastasis, and protein degradation. The aforementioned dys-regulated proteins were confirmed by western immunoblotting. Proteomics results were further translated to prognostic markers by analyses of biopsy samples. Results of cohort studies demonstrated that over-expressions of glutamine synthetase, alcohol dehydrogenase (NADP^+^), fatty acid binding protein 4, and toll interacting protein in clinical specimens were all significantly associated with galectin-1 up-regulation. Univariate analyses showed that de-regulations of glutamine synthetase and fatty acid binding protein 4 in clinical samples were respectively linked to disease-specific survival and metastasis-free survival.

## 1. Introduction

The surfaces of bladders and ureters are mostly covered by urothelium. Among various heterogeneous types of bladder tumors, urothelial carcinoma is the most prevalent lesion. Urinary bladder urothelial carcinoma (UBUC) is often recognized as non-invasively papillary or superficially invasive tumors [1]. Nevertheless, some of the aforementioned tumors develop local recurrence and may cause distal invasion [1]. UBUC cells are identified as low-grade or less differentiated and high-grade cells.

Galectin-1 is one of the fifteen mammalian proteins belonging to the β-galactoside binding family. It is a 14-kDa monomer with one carbohydrate-binding domain and usually forms a non-covalent homodimer in the cells [2]. The galectin-1 protein expression is precisely controlled by the methylation degree of the *LGALS1* gene promoter located on chromosome 22q12 [3].

Galectin-1 de-regulation significantly affects cell transformation [4], cell proliferation [2], angiogenesis [5], and cell adhesion and invasiveness [6,7,8], as well as immunosuppression [9,10]. Up-regulated galectin-1 expression has been observed in UBUC [11], colorectal cancer [12], breast cancer [10], lung cancer [13], head/neck cancer [14], ovarian cancer [15], prostate carcinoma [16], glioma [17], Kaposi’s sarcoma [18], and Hodgkin’s lymphoma [19]. It is found that tumor stages, tumor invasiveness, and metastasis are associated with the increased galectin-1 expression in UBUC [11]. Furthermore, there is a positive correlation between poor prognosis and increased galectin-1 amount in lesions in patients with UBUC [11] and glioblastoma [20]. Comparative immunohistochemical results showed a higher galectin-1 protein amount in invaded areas than that in non-invaded areas of human U87 and U373 xenografted glioblastoma in nude mice [21]. The aforementioned phenomena may be attributed to the modification of actin through increasing the small RhoAGTPase expression. In surprising similarity, immunohistochemistry (IHC) analyses of oral squamous cell carcinoma (OSCC) specimens demonstrated that galectin-1 is over-expressed at the invasion front [22]. Further galectin-1 augments the expression and activities of matrix metalloproteinase proteins (MMP) 2 and 9 to provoke OSCC cell invasion. It can also induce cytoskeleton re-organization to promote invasiveness by regulating the activity of cell division cycle 42 (cdc42), a member of the RhoGTPase family [8]. The above results implicate that the galectin-1 protein is intimately associated with tumor invasiveness.

In addition to the link to invasion, the galectin-1 protein has been shown to bind to GTPase HRas proto-Oncogene (H-Ras) and cause the membrane anchorage of H-Ras. Increased galectin-1 expression in tumor cells eventually enhances H-Ras membrane localization and evokes the RAF proto-oncogene serine/threonine-protein kinase (Raf-1)/mitogen-activated protein kinase (MEK)/ extracellular signal–regulated kinases (Erk) pathway to strengthen the cell transformation [23]. In addition to the involvement in cell transformation, Rubinstein et al. (2004) found that melanoma cells can secret the galectin-1 protein to prevent cell-mediated immunity by provoking activated T cell apoptosis, thus giving rise to the immune privilege of tumor cells [9].

Our previous studies show that galectin-1 over-expression in tumor cells is correlated with tumor stages, grades, and invasion [11]. It can also significantly predict disease specific survival and metastasis-free survival at the univariate and multivariate levels. Cell signaling examination indicates that the galectin-1 protein takes part in UBUC cell invasion by regulating the MMP9 activity via the Ras–Ras-related C3 botulinum toxin substrate 1 (Rac1)–mitogen-activated protein kinase kinase kinase 4 (MEKK4)–c-Jun N-terminal kinase (JNK)–Activator protein 1 (AP1) signaling pathway [24]. In continuation of our previous investigation on the role of galectin-1 in UBUC tumorigenesis, in this study, proteomics strategies were implemented in order to find more key signaling pathways which are initiated by the galectin-1 protein in tumor cells further to our existing findings. Proteomics results demonstrated that de-regulated proteins in galectin-1 knockdown T24 cells might participate in lipid/amino acid/energy metabolism, cytoskeleton, cell proliferation, cell-cell interaction, cell apoptosis, metastasis, and protein degradation. Furthermore, the results of cohort studies showed that dys-regulations of glutamine synthetase and fatty acid binding protein 4 in clinical samples were respectively linked to disease-specific survival and metastasis-free survival in univariate analyses.

## 2. Results

### 2.1. Search for the De-Regulated Proteins in Sh-Gal(+120) T24 Cells by Two-Dimensional Gel Electrophoresis

**Figure 1:**
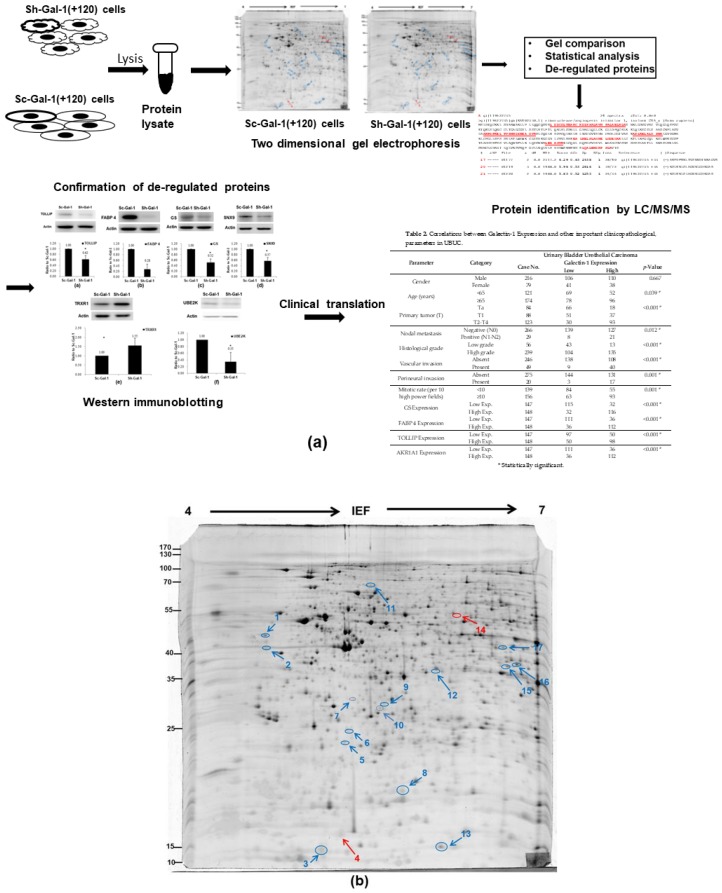

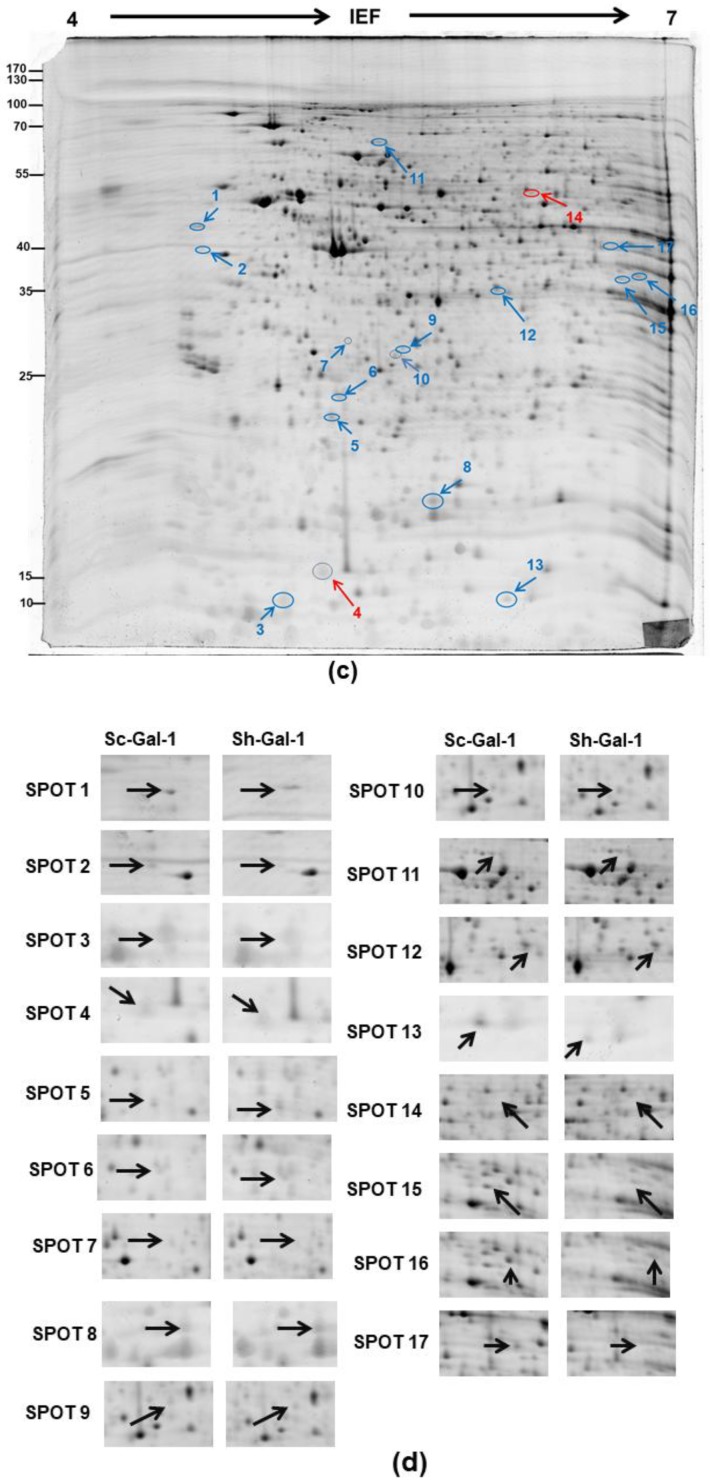
2-DE protein profiles of Sc-Gal-1(+120) and Sh-Gal-1(+120) T24 cells. (**a**) The experimental design; (**b**) Sc-Gal-1 (+120) T24 cells; (**c**) Sh-Gal (+120) T24 cells. (**d**) The expanded images of de-regulated protein spots. A total of 200 μg of T24 lysate proteins was used in each 2-DE gel, carried out as described in Materials and Methods. Down-regulated protein spots were indicated by blue arrows, while up-regulated ones were indicated by red arrows.

In our previous studies, we found that the galectin-1 protein is correlated with UBUC cell invasive capability by regulating the MMP9 activity through the Ras–Rac1–MEKK4–JNK–AP1 signaling pathway [24]. In this study, we exploited proteomics to find more novel molecular pathways evoked by galectin-1 dysregulation in UBUC cells, which might be related to UBUC carcinogenesis. The experimental design for this study is summarized in Figure 1. To obtain the above goal, Lavapurple^TM^-stained two-dimensional gel electrophoresis (2-DE) was first carried out to acquire the protein profiles of Sh-Gal-1(+120) (galectin-1 knockdown stable cell line) and Sc-Gal-1(+120) (scrambled control) T24 cells, as described in Materials and Methods. Then, the 2-DE protein pictures of Sh-Gal-1(+120) T24 cells were compared to those of Sc-Gal-1(+120) T24 cells to search for the differentially expressed protein features which were recognized according to the definition, as described in Materials and Methods. To prevent the gel-to-gel variation, 11 replica gel pairs were obtained to find those de-regulated proteins provoked by galectin-1 knockdown. The typical 2-DE gel diagrams of Sh-Gal-1(+120) and Sc-Gal-1(+120) T24 cells are shown in Figure 1 and the other 10 pairs are provided in the supplementary materials (Supplementary Figure S1).

The protein profiles of Sh-Gal-1(+120) T24 cells were very similar to those of Sc-Gal-1(+120) T24 cells, but the amount of some identical proteins in the knockdown cell line was more than twice as high as that in its scrambled counterpart. Fifteen under-expressed and two over-expressed protein spots were found, as indicated by the arrows in Figure 1. Spots 4 (*p* = 0.07) and 10 (*p* = 0.05) were near *p* < 0.05. Spot 7 (*p* < 0.01) was not listed because it was not identified by liquid chromatography/tandem mass spectrometry (LC-MS/MS) (Table 1 and Supplementary Table S1).

### 2.2. Identification of the Differentially Expressed Proteins in Sh-Gal-1(+120) T24 Cells

After the recognition of dys-regulated proteins by 2-DE gel comparison, de-regulated proteins were identified using LC-MS/MS, as described in Materials and Methods. The spectrometric results of down- and up-regulated proteins are shown in Table 1. The experimental molecular weight and pI of each de-regulated protein were close to the theoretical numbers and most of the spectrometric protein coverages were near or over 20%, except for spots 2 and 17. The fragment ion spectra (MS2 spectra) are presented in Supplementary Figure S2. Literature reviews indicated that the identified de-regulated proteins were associated with amino acid/energy metabolism (GS) [25], lipid/energy metabolism (FABP 4) [26], cytoskeleton (STMN1, TBCA) [27,28], cell proliferation (STMN1, PMF1, RNH1) [29,30,31], cell-cell interaction (NANS) [32], cell apoptosis (TRXR1, RCN1) [33,34], metastasis (NANS, SNX9) [32,35], protein degradation (TOLLIP, UBE2K) [36], and glucose metabolism (AKR1A1).

### 2.3. Confirmation of De-Regulated Proteins Found in Sh-Gal-1(+120) T24 Cells by Western Immunoblotting

**Figure 2:**
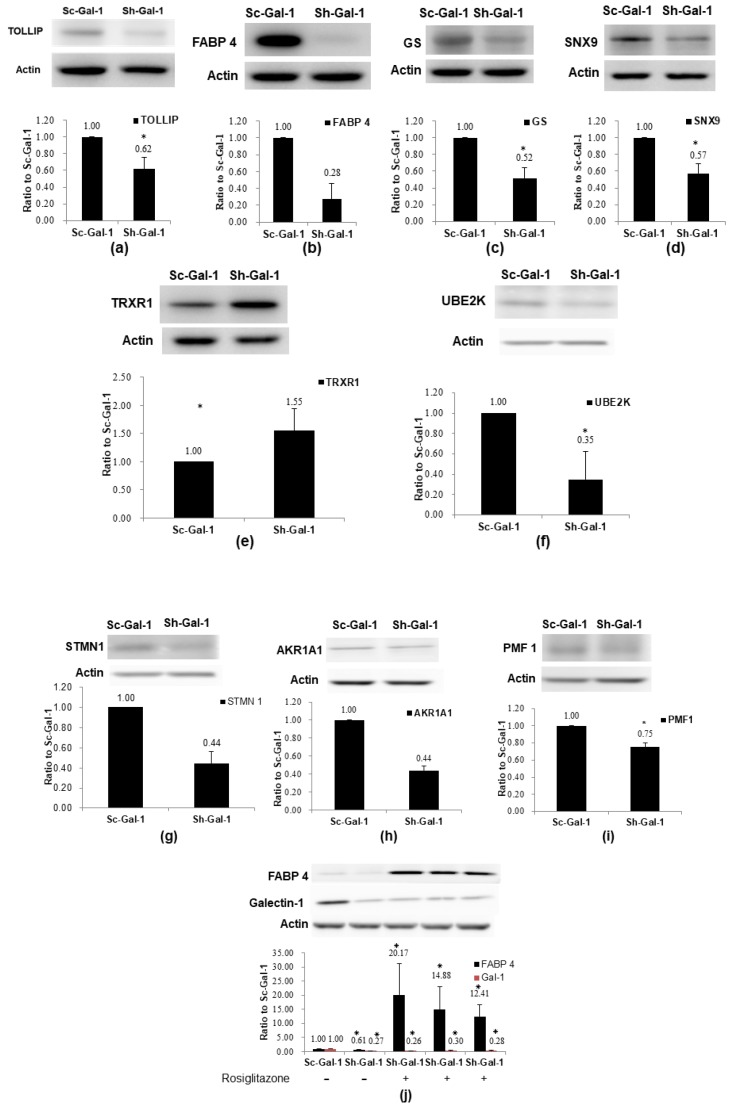
Confirmation of de-regulated proteins evoked by galectin-1 knockdown in T24 cells. (**a**) TOLLIP; (**b**) FABP 4; (**c**) GS; (**d**) SNX9; (**e**) TRXR1; (**f**) UBE2K; (**g**) STMN1; (**h**) AKR1A1; (**i**) PMF1; (**j**) Effects of rosiglitazone (PPAR-γ agonist) on the FABP 4 expression. Western immunoblotting was carried out as described in Materials and Methods. The blot was the representative result of three independent experiments. The protein expression fold (mean ± S.D.) was expressed as the ratio of normalized intensity of the protein of interest (observed protein/actin) in Sh-Gal-1(+120) T24 cells to that in Sc-Gal-1(+120) T24 cells. * *p *< 0.05.

After the identification of de-regulated proteins, we validated our findings via proteomics examination using western immunoblotting. The expressions of TOLLIP, FABP 4, GS, SNX9, UBE2K, PMF1, AKR1A1, STMN1, and TRXR1 were investigated in Sh-Gal-1(+120) T24 cells. These nine proteins were selected from de-regulated proteins for validation due to their respective high-fold changes, except for STMN1, and possible association with carcinogenesis. Results of western immunoblotting demonstrated that the expressions of TOLLIP, FABP 4, GS, SNX9, TRXR1, UBE2K, STMN1, AKR1A1, and PMF1 were under-expressed in Sh-Gal-1(+120) T24 cells, while TRXR1 was over-expressed in Sh-Gal-1(+120) cells. The above results were in line with proteomics results. In this study, our results showed that galectin-1 knockdown in T24 cells could lower FABP 4 protein expression. Currently, no documented literature has reported the relationship between FABP 4 and galectin-1 in cancer cells. Boiteux et al. (2009) found that the peroxisome proliferator-activated receptor γ (PPAR-γ) can regulate FABP 4 protein expression [37]. Thus, we then explored the impacts of rosiglitazone, a PPAR-γ agonist, on the FABP 4 expression in Sh-Gal-1(+120) T24 cells. The results in Figure 2 demonstrated that rosiglitazone treatment could profoundly rescue FABP 4 protein expression in Sh-Gal-1(+120) T24 cells, but not galectin-1 expression, suggesting that galectin-1 was upstream from PPAR-γ and FABP 4 was downstream from PPAR-γ. Galectin-1 might regulate FABP 4 expression through PPAR-γ.

### 2.4. Confirmation of Proteomics Data Using Cohort Studies of GS, FABP 4, TOLLIP, and AKR1A1

The results from western immunoblotting validated the dys-regulated proteins provoked by galectin-1 knockdown in Sh-Gal-1(+120) T24 cells. Furthermore, we designed cohort studies to confirm the expressed levels of GS, FABP 4, TOLLIP, and AKR1A1 in clinical specimens. Results of cohort studies showed that galectin-1 expression in biopsy samples was significantly correlated with the primary tumor (pT) status, grade, nodal metastasis, vascular invasion, perineural invasion, and mitotic rate. More importantly, the expression levels of GS, FABP 4, TOLLIP, and AKR1A1 in clinical samples were associated with the galectin-1 amount (Table 2), respectively. This finding was consistent with that of proteomics studies.

**Figure 3:**
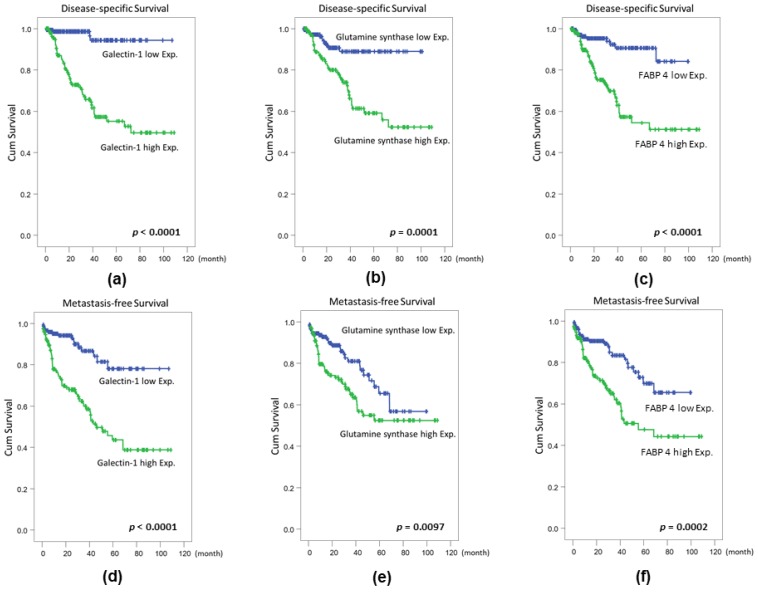
Survival analyses by log-rank tests. Disease-specific survival (DSS) and metastasis-free survival (MFS) of patients with urinary bladder urothelial carcinoma (UBUC) were significantly associated with (**a**,**d**) galectin-1 over-expression, (**b**,**e**) GS over-expression, (**c**,**f**), and FABP 4 over-expression.

The results of the univariate log-rank analysis on disease-specific survival (DSS) and metastasis-free survival (MFS) indicated that primary tumor status, histological grade, nodal metastasis, the presence of vascular invasion and perineural invasion, the high mitotic rate, galectin-1 (Figure 3a), GS (Figure 3b), and FABP 4 (Figure 3c) expression levels were significantly correlated with DSS (Table 3). However, the impacts on both TOLLIP and AKR1A1 abundance were not significant in predicting DSS. In line with our previous results [11] and the above proteomics data, a high galectin-1 expression level was predictive of a shorter metastasis-free survival time (Figure 3d). The same results were also observed in patients with high amounts of GS (Figure 3e) or the FABP 4 (Figure 3f) protein. However, the impacts on both of the TOLLIP and AKR1A1 amounts were not significant (Table 3). Further multivariate analyses demonstrated that factors of primary tumor status (T1, relative risk (R.R.) = 2.732; T2–T4, R. R. = 9.346), mitotic rate (R.R. = 2.048), and galectin-1 level (R.R. = 4.628) independently predicted MFS (Table 3), respectively. Moreover, the aforementioned three factors were also significantly predictive of worse MFS, including primary tumor status (T1, R.R. = 4.181; T2–T4, R.R. = 5.543), a high mitotic rate (R.R. = 1.885), and a high galectin-1 level (R.R. = 2.386) (Table 3).

## 3. Discussion

Our previous findings suggest that the galectin-1-increased expression in UBUC is intimately associated with the primary tumor status, grade, and invasion [11]. Univariate and multivariate analyses demonstrate that galectin-1 over-expression in UBUC can also independently predict DSS and MFS. Further signaling pathway investigation indicates that the galectin-1 protein participates in UBUC cell invasion by modulating the MMP9 activity through the Ras–Rac1–MEKK4–JNK–AP1 signaling pathway [24]. To expand our knowledge regarding the role of galectin-1 in UBUC carcinogenesis, in this study, proteomic approaches were exploited to find more key signaling pathways which were provoked by galectin-1 up-regulation in UBUC cells.

In this study, the proteome maps of Sh-Gal-1(+120), a galectin-1 knockdown stable cell line, were compared to those of scrambled control cells, Sc-Gal-1(+120) cells. Our results showed that fifteen under-expressed and two over-expressed proteins were attributed to galectin-1 depletion in T24 cells. These impaired-regulated proteins found by proteomics could be categorized into a wide range of protein types involved in amino acid/energy metabolism (GS), lipid/energy metabolism (FABP 4), cytoskeleton (STMN1, TBCA), cell proliferation (STMN1, PMF1, RNH1), cell-cell interaction (NANS), cell apoptosis (TRXR1), invasion (SNX9), protein degradation (UBE2K, TOLLIP), and glucose metabolism (AKR1A1). SNX9, UBE2K, PMF1, STMN1, and TRXR1 were confirmed by western immunoblotting in the next step, while FABP 4, GS, TOLLIP, and AKR1A1 were validated by western immunoblotting and cohort studies. The aforementioned four proteins are found to be correlated with galectin-1 expression, tumor progression, and DSS/MFS with substantial clinical significance. The FABP 4 protein may participate in the trafficking of lipids to specific compartments. Abnormal FABP 4 functions may disrupt the lipid-mediated biological processes and result in diseases such as diabetes, obesity, and cancer [26]. Recently, Nieman et al. (2011) reported that FABP 4 demonstrates a higher up-regulation level in human omental metastatic ovarian lesions than in primary clinical ovarian tumor specimens and is over-expressed at the adipocyte/cancer cell interface [38]. Their results suggested that adipocytes support the cancer cells to metastasize from ovary to omentum. In omental adipocytes, lipolysis is induced to produce fatty acid and FABP 4 participates in fatty acid trafficking to cancer cells. In tumor cells, fatty acid is broken down by β-oxidation to fuel cancer cells for fast cell proliferation [38]. In contrast to the ovarian cancer, FABP 4 augmented expression is found to be associated with pTa UBUC with subsequent progression [39]. Our present studies suggested that galectin-1 knockdown reduced FABP 4 expression, likely through modulating PPAR-γ. Taken together, galectin-1 might take part in UBUC progression and invasion through regulating PPAR-γ/FABP 4.

Rapidly growing tumor cells would increase nucleotide and protein synthesis. They rely on the continuous provision of amino acids. Glutamine contains the most abundant amino acid in the body and is also the richest source for the nontoxic form of ammonia. Thus, tumor cells are highly dependent upon glutamine. When tumors begin to grow in the body, they elicit the host to mobilize and augment circulating glutamine. In addition, tumor cells use aerobic glycolysis and glutaminolysis (for breaking down glutamine to α-ketoglutarate to enter tricarboxylic acid (TCA) cycle) to quickly obtain the energy and precursor molecules for the synthesis of macromolecules [25]. Glutamine synthetase catalyzes the conversion of glutamate to glutamine and is thus involved in glutamine metabolism. GS has been implied as an early maker for hepatocellular carcinoma [40] and its positive immunostaining is correlated with both specific and overall mortality at the multivariate level [41]. Our data and the above observations all lead to the implication that galectin-1 might be involved in metabolic transformation. TOLLIP and UBE2K are shown to be associated with protein degradation. TOLLIP is originally recognized as an effector in interleukine-1 and Toll-like receptor signaling pathways [36]. However, it is also considered to take part in the trafficking of ubiquitinated proteins [36]. The increased protein turnover contributes to carcinogenesis by giving anti-apoptotic protection to tumor cells and augmented elimination of abnormal proteins [42]. Our present data showed that galectin-1 knockdown reduced TOLLIP expression and galectin-1 expression was closely associated with TOLLIP expression in the findings of biopsy samples. It might be likely that galectin-1 favors UBUC development by increasing the protein turnover. This is the first observation that TOLLIP was correlated with galectin-1 expression and UBUC patient’s DSS/MFS. More molecular and functional studies are required to uncover the role of TOLLIP in UBUC carcinogenesis. In this study, we found that AKR1A1 expression was significantly associated with galectin-1 expression. Since the above results were the first findings, further work should be carried out to elucidate the signaling route by which galectin-1 regulates AKR1A1 expression.

Among the confirmed de-regulated proteins by western immunoblotting, STMN1 is a regulatory protein for microtubule assembly, and thus modulates the cytoskeleton [27]. It also participates in cell cycle regulation [29]. Cytoskeleton changes and impaired cell cycle regulation are essential for carcinogenesis. STMN 1 has been shown to be up-regulated in multiple tumors and is significantly linked to a poor survival rate of the patients [43]. Results in this investigation showed that galectin-1 knockdown decreased STMN 1 expression, suggesting that galectin-1 might control UBUC progression, probably through modulating STMN 1 expression. SNX9 is shown to provoke breast cancer metastasis via modulating RhoGTPase [35]. SNX9 can control the activation of RhoA and cdc42GTPase. It also controls breast cancer cell invasion through the RhoA-ROCK pathway and N-WASP. Our previous findings suggested that galectin-1 may promote UBUC invasion via the Ras–Rac1–MEKK4–JNK pathway [24]. The present observation might uncover an alternative pathway for galectin-1 to contribute to UBUC invasion. PMF1 is recognized as one of the proteins participating in polyamine homeostasis. Polyamine homeostasis impacts the cellular transcription rate and the decreased expression of early growth-associated genes including c-jun, c-myc, and c-fos [30]. Besides, Aleman et al. (2008) reported that PMF1 methylation is significantly linked to UBUC progression [44]. Our present data provided the novel observation that galectin-1 could regulate PMF1 expression.

In conclusion, the results of this study implicated that multi-functional galectin-1 contributed to UBUC carcinogenesis, probably through regulating amino acid/lipid/glucose metabolism, cytoskeleton rearrangement, cellular transcription, cell invasion, and protein degradation.

## 4. Materials and Methods

### 4.1. Cell Lines

Human UBUC T24 cells (high grade/invasive) were bought from Bioresource Collection and Research Center, Hsinchu, Taiwan and cultured at 37 °C in McCoy’s5A (GIBCO (Life Technologies), Grand Island, NY, USA) medium, supplemented with 10% (*v*/*v*) fetal bovine serum (FBS). Human UBUC J82 cells (high grade) were gifted by Chien-Feng Li from the Department of Pathology, Chi-Mei Medical Center, Tainan, Taiwan and cultured at 37 °C in Dulbecco’s Modified Eagle Medium supplemented with 10% (*v*/*v*) FBS (GIBCO, Grand Island, NY, USA).

### 4.2. Construction of Galectin-1 Knockdown T24 Cells with Short-Hairpin RNA (shRNA)

Galectin-1 knockdown stable clones were established as described previously [24]. In brief, custom-made DNA precursor oligonucleotides of silencing RNA (siRNA) were annealed and the DNA duplex was cloned between *Bgl*II and *Hind*III restriction enzyme sites of the expression vector pSUPER-NEO (Oligoengine Corporate, Seattle, WA, USA), which has a neomycin resistance gene as the selection marker. Two complementary oligonucleotide strands of the siRNA DNA precursor, which would be transcribed into a shRNA processed into a 19-mer small interference RNA (siRNA), were designed with Oligoengine software (version 2.0, Oligoengine Corporate, Seattle, WA, USA). Oligonucleotide DNA sequences are listed in the supplementary File S1. T24 cells were transfected with the siRNA expression plasmid using PolyJet™ transfection reagents (SignaGen Laboratories, Ijamsville, MD, USA) according to the manufacturer’s suggestions. Stable knockdown T24 cells were screened in the medium containing 150 μg/mL of G418 antibiotics. T24 cell clones with the best knockdown results were chosen using western immunoblotting and real-time reverse transcriptase polymerase chain reaction (RT-PCR) from 20 cell clones with targeting at +120 nucleotide (nt), named Sh-Gal-1(+120). Control cells were constructed by the stable transfection of T24 cells with the scrambled siRNA DNA precursor and T24 cell clones with the least impacts on galectin-1 protein amount were chosen from 20 cell clones scrambled at +120 nt and recognized as Sc-Gal-1(+120).

### 4.3. Preparation of Protein Lysates for Two-Dimensional Gel Electrophoresis

In this study, 70–80% confluent Sh-Gal-1(+120) and Sc-Gal-1(+120) T24 cells were trypsinized and recovered by centrifugation at 1000× *g* for 5 min at 25 °C and lysed with lysis solution (7 M urea (J.T Baker, Center Valley, PA, USA), 2 M thiourea (J.T Baker, Center Valley, PA, USA), 100 mM dithiothreitol (DTT) (USB Corcorporation, Cleveland, OH, USA), 4% (*v*/*v*) 3-[(3-cholamidopropyl) dimethylammonio]-1-propanesulfonate (CHAPS) (aMResco, Solon, OH, USA), 40 mM Tris-base (pH 10) (aMResco, Solon, OH, USA), 1 mM phenylmethanesulfonylfluoride (PMSF) (aMResco, Solon, OH, USA), and one Complete Mini protease inhibitor cocktail tablet (Roche, Diagnostics, Indianapolis, USA) per liter) by shaking at room temperature for 1 h. Then, the lysates were centrifuged at 349,000× *g* for 2 h at 15 °C in Type 90 Ti rotor (Beckman Coulter, Fullerton, CA, USA) and proteins present in the supernatant were precipitated with the 2-D Clean-up Kit (GE Healthcare Bio-Sciences AB, Uppsala, Sweden) according to the manufacturer’s procedure. The pellet from the 2D Cleanup kit was first re-suspended in MRB buffer (7M Urea, 4% (*v*/*v*) CHAPS) and then incubated at room temperature for 1 h with shaking to dissolve the pellet. Then, the protein concentration of the lysate was quantitated by the Bio-Rad DC protein assay. After measurement, an appropriate amount of thiourea, IPG buffer, and DTT were added into the lysate to re-constitute the rehydration buffer (7 M urea, 2 M thiourea, 0.5% (*v*/*v*) immobilized pH gradient (IPG) buffer (pH 4–7), 4% (*v*/*v*) CHAPS, 1 mM DTT and 0.1 (*w*/*v*) bromophenol blue (aMResco, Solon, OH, USA)) and incubated at room temperature with shaking for another 1 h. Following this, the protein lysates were stored at −80 °C until isoelectric focusing.

### 4.4. Isoelectric Focusing (IEF) and SDS-Polyacrylamide Gel Electrophoresis (SDS-PAGE)

Isoelectric focusing (IEF) and sodium dodecyl sulfate polyacrylamide gel electrophoresis (SDS-PAGE) were conducted as described previously [45]. The 18-cm immobibline dry strips (pH 4–7) (GE Healthcare Bio-Sciences AB, Uppsala, Sweden) were rehydrated within the BioRad Protean IEF Cell for 16 h at 20 °C with 300 μL rehydration buffer including 200 μg protein lysates respectively prepared from Sh-Gal-1(+120) and Sc-Gal-1(+120) T24 cells. Then, the proteins were concentrated at 20 °C at 50, 100, 200, 500, 1000, 5000, and 8000 V, respectively, with a total of 81,434 voltage-hours. After isoelectric focusing, the gel strips were equilibrated in the equilibration buffer (6 M urea, 30% (*v*/*v*) glycerol (Kanto Chemical, Portland, OR, USA), 2% (*w*/*v*) SDS (aMResco, Solon, OH, USA)) containing 2% (*w*/*v*) DTT for 15 min and then in the equilibration buffer containing 5% (*w*/*v*) iodoacetamide (aMResco, Solon, OH, USA) for a further 15 min. The equilibrated gel was loaded onto the top of a 12.5% (*w*/*v*) polyacrylamide gel and sealed with 0.5% (*w*/*v*) agarose (aMResco, Solon, OH, USA) and the proteins were separated at 420 V using BioRad Protean IIxi until bromophenol blue reached the bottom of the gel.

### 4.5. Lavapurple Staining, Image Analysis, and Statistical Analysis

Image analysis and statistical analysis were carried out as described formerly with some exceptions [45]. LavaPurple staining was carried out following the manufacturer’s suggested protocol (Fluorotechnics, Sydney, Australia). In brief, the 2-DE gels were first soaked within solution I ((1% (*w*/*v*) citric acid and 15% (*v*/*v*) ethanol) for 1.5 h and then incubated in solution II (0.63% (*w*/*v*) boric acid, 0.385% (*w*/*v*) and 0.5% (*v*/*v*) Lavapurple) for 1.5 h in the dark. Lastly, the gels were put in solution III (15% (*v*/*v*) ethanol) for 30 min and then in solution I for 30 min. Lavapurple-stained 2-DE gels were scanned using a Typhoon 9400 fluorescence scanner (GE healthcare, Little Chalfont, UK) with a green laser (green laser PMT: 600 volt and emission filter: 580 BP) to obtain the digital images. To find the de-regulated proteins, a total of 11 pairs of well-separated gel images acquired from Sh-Gal-1(+120) and Sc-Gal-1(+120) T24 cells were compared with PDQuest 8.0.1 (BioRad, Hercules, CA, USA) software. Differentially expressed protein spots recognized by computer analysis were further verified by visualization. The intensity (volume) of the spot was quantitated and normalized as a percentage (ppm) of the total intensities of all spots in a gel (total normalized volume). Normalized volumes of individual de-regulated protein spots across all the replica gels of Sh-Gal-1(+120) and Sc-Gal-1(+120) T24 cells were first analyzed by the normal distribution test and then the Student’s *t*-Test (STATISTICA Version 10.0 MR1, StatSoft, Tulsa, OK, USA) was carried out when reaching normal distribution. However, when normal distribution was not obtained, log transformation was carried out, followed by the normal distribution test and then the Student’s *t*-Test. The normalized volume of each spot in Sh-Gal-1(+120) cells was compared to that of the same spot in Sc-Gal-1(+120) cells. In all cases, statistical variance of the Sh-Gal-1(+120): Sc-Gal-1(+120) spot normalized volume ratio within 95% (Student’s *t*-Test; *p* < 0.05) was deemed as significantly different. Furthermore, the differentially expressed proteins present in at least six out of 11 gel pairs were regarded as galectin-1-associated proteins.

### 4.6. In-Gel Digestion and Protein Identification Analysis Via Liquid Chromatography-Tandem Mass Spectrometry (LC-MS/MS)

Since the ideal identification of Lavapurple stained protein spot was not obtained, the silver stained protein spots of interest were picked up for in-gel digestion. The protocol of silver staining is listed in the supplementary File S1. The in-gel digestion and mass spectrometric protein identification were carried out as described previously [45]. Briefly, the peptides present in protein digest were resolved in the LTQ-Orbitrap hybrid tandem mass spectrometer (ThermoFisher, Waltham, MA, USA) in-line coupled with the Agilent 1200 nanoflow HPLC system mounted with LC Packing C18 PepMap 100 (length: 5 mm; internal diameter: 300 μm; bead size: 5 μm) as the trap column and Agilent ZORBAX XDB-C18 (length: 50 mm; internal diameter: 75 μm; bead size: 3.5 μm) as the resolving column. File Converter in the Xcalibur 2.0SR package (ThermoFisher, Waltham, MA, USA), as well as an in-house program, were exploited to obtain the MS/MS information and the charge and mass of each analyzed peptide were calculated. TurboSequest program (ver. 27, rev. 11; Thermo Fisher Scientific, Waltham, MA, USA) was then implemented to determine the best matched peptides from a non-redundant protein database whose FASTA sequences were downloaded from the National Center for Biotechnology Information (ftp://ftp.ncifcrf.gov/pub/nonredun/) on 12 October 2010 with 541,927 entries. Only the tryptic peptides with ≤2 missed cuts were recorded. During the database search, the mass ranges were 1 and 3.5 m/z for fragment and precursor ions, respectively. The protein identities were only confirmed when there were at least two peptides matched and search results had a high Xcore (i.e., ≥2.0 for doubly charged peptides and ≥3.0 for triply charged ones) and minimal differences between the observed and hypothetical masses (i.e., ΔM < 10 ppm). For each set of MS/MS analyses, 25 fmol of bovine serum albumin (BSA) (aMResco, Solon, OH, USA) in gel was analyzed in parallel for verification of the effectiveness of the entire protein identification procedure, including in-gel digestion, nanoflow HPLC, MS/MS, and informatics analyses. The experimental data were only taken into account when a 10 ppm mass accuracy and over 70% coverage were observed in the co-processed BSA samples.

### 4.7. Western Immunoblotting

Western immunoblotting was carried out as mentioned before [45]. Sh-Gal-1(+120) and Sc-Gal-1(+120) cells were collected and lysed in the lysis buffer (10 mM Tris (pH 8.0), 0.32 M sucrose (Avantor Performance Matericals, Center Valley, PA, USA), 1% (*v*/*v*) Triton X-100 (Merck Millipore, Darmstadt, Germany), 5 mM EDTA (Merck Millipore, Darmstadt, Germany), 2 mM DTT and 1 mM PMSF). After measurement of the protein concentration with the Bio-Rad DC protein assay kit, an equal volume of 2× sample buffer (0.1M Tris (pH 6.8), 2% (*w*/*v*) SDS, 0.2% (*v*/*v*) β-mercaptoethanol (aMResco, Solon, OH, USA), 10% (*v*/*v*) glycerol, and 0.0016% (*w*/*v*) bromophenol blue) was mixed with the protein lysate. Suitable amounts of the protein lysates were resolved using electrophoresis at 100 V with 10% (*w*/*v*) SDS-PAGE, and further transferred onto PVDF membranes (Strategene, La Jolla, CA, USA). After blocking for 1 h in 3% (*w*/*v*) bovine albumin serum (BSA) (aMResco, Solon, OH, USA) at room temperature, membranes were probed for 2 h at room temperature with primary antibodies (UBE2K (1:10,000), STMN1 (1:8000), TOLLIP (1:2000), FABP 4 (1:1000), AKR1A1(1:5000), GS (1:500) and actin (1:5000) from Merck Millipore, Darmstadt, Germany; PMF1 (1:1000), SNX9 (1:2000), TRXR1 (1:1000) from Santa Cruz Biotechnology, Dallas, TX, USA). The membranes were washed and then hybridized with appropriate secondary antibodies for 1 h at room temperature. Secondary antibodies binding on the membranes were detected by the chemiluminescence ECL detection system (GE Healthcare Bio-Sciences AB, Uppsala, Sweden) using the Fujifilm LAS-4000 Luminescent Image Analyzer (Fujifilm Corporation, Tokyo, Japan). The intensity of each protein band, normalized with the actin protein expression level, was determined by PDQUEST Quantity One software (Bio-Rad Laboratory, Hercules, CA, USA) and analyzed with the Student’s *t*-Test (STATISTICA Ver 10.0 MR1, StatSoft, Tulsa, OK, USA).

### 4.8. Case Selection

For immunohistochemistry, the Institutional Review Board (IRB) of Chi Mei Medical Center admitted the retrospective retrieval of 295 available primary UBUC blocks (Identification code: IRB10102-004, approved by IRB of Chi Mei Medical Center, project started from 14 Feb. 2012), which were adopted from the patients undergoing surgical resection with curative intent between January 1998 and May 2004. However, those who received the palliative resection were excluded. Patients with validated or suspicious lymph node metastasis underwent regional lymph node dissection. Cisplatin-based postoperative adjuvant chemotherapy was carried out in patients with pT3-pT4 status or nodal linkage. The UBUC histologic diagnosis was validated in all specimens according to the recent World Health Organization classification. Histologic grading was identified based upon Edmonson–Steiner criteria, while tumor staging was recognized according to the seventh edition of the American Joint Committee on Cancer system. Medical charts were reviewed for each patient to ensure the accuracy of other related clinicopathologic data. Follow-up information was available in all cases with a median period of 42 months (ranging 3–176 months).

### 4.9. Immunohistochemistry (IHC) and Statistical Analysis

IHC was conducted on representative tissue sections dissected from formalin-fixed, paraffin-embedded tumor blocks at a 3-μm thickness. Xylene was first used to deparaffinize the slides. Then, the slides were rehydrated with ethanol and heated using a microwave in a 10 mM citrate buffer (pH 6) for 7 min to retrieve the antigen epitopes. A total of 3% H_2_O_2_ was used to quench endogenous peroxidase. After antigen retrieval, slides were washed in Tris-buffered saline for 15 min and then hybridized with a primary antibody again for 1 h, followed by antibody detection using a ChemMate EnVision^TM^ kit (K5001; DAKO, Glostrup, Denmark). Two expert pathologists (CF Li and HL He) blinded to clinicopathological information and patient outcomes interpreted the immunostainings. The immune-intensities of membranous and cytosolic staining of Galectin 1, FABP 4, GS, TOLLIP, and AKRAK1 were respectively recorded by *H* score. Scoring of the immunoreactivity was measured based on a composite of both the percentage and intensity of the cytoplasm of positively stained tumor cells to generate an *H* score, using the equation:

$$H\;\mathrm{score}=\;\sum P_{i}\left(I\;+\;1\right)$$

where *i* is the intensity of the stained tumor cells (0 to 3+), and Pi is the percentage of stained tumor cells for each intensity varying from 0% to 100%. The specimens that showed an H-score above the median value were defined as high expression, while the cases below the median value were defined as low expression. The median H-score of all specimens was regarded as the cutoff point to divide high and low expression.

Statistical analyses were carried out with SPSS 14.0. The immuno-expressions of Galectin 1 (Invitrogen, Carlsbad, CA, USA), FABP 4 (Epitoimcs, Burlingame, CA, USA), GS (BD Transduction Laboratories™-BD Bioscience, San Jose, CA, USA), TOLLIP (Novus Biologicals, Littleton Colorado, USA), and AKRAK1 (Abcam, Cambridge, MA, USA) were compared by various parameters with the chi-square test. The end points evaluated in the whole cohort were DSS and MFS measured based on the date of UBUC operation to disease-related mortality and metastasis. In stepwise forward fashion, parameters at univariate *p <* 0.05 were basically analyzed for their relative prognostic importance using the Cox regression model. Of all analyses, the two-sided test of significance with *p *< 0.05 was considered significant.

## 5. Conclusions

The results of this study implicated that multi-functional galectin-1 contributed to UBUC carcinogenesis, probably through regulating amino acid/lipid/glucose metabolism, cytoskeleton rearrangement, cellular transcription, cell invasion, and protein degradation.

## Figures and Tables

**Table 1:** Mass spectrometric data and identification of the galectin-1-associated de-regulated proteins.

Spot	Identity	Accession Number	Theo. ^b^ PI/Mr.	Exper. ^c^ PI/Mr.	Matched ^d^ Peptide Number	Coverage (%) ^e^	Incidence	Fold ^f^ Difference	*p*-Value
1	ribonuclease/angiogenin inhibitor 1 (RNH1) ^a^	P13489	4.5/49	4.74/49.4	11	22.1	6/11	−2.77	0.003
2	reticulocalbin 1 (RCN1)	Q15293	4.6/42.7	4.86/38.9	3	11.8	6/11	−3.74	0.04
3	galectin 1 (LGALS1)	P09382	4.8/16.7	5.3/14.7	8	52.6	8/11	−56.5	0.01
4	tubulin specific chaperone A (TBCA)	O75347	5.3/12.9	4.9/17.8	8	63.8	6/11	+2.92	0.07
5	ubiquitin conjugating enzyme E2 K (UBE2K)	P61086	5/26.1	5.33/22.6	9	46.5	6/11	−3.71	0.0005
6	polyamine-modulated factor 1 (PMF1)	Q6P1K2	5/27.7	5.37/23.3	5	33.7	7/11	−5.1	0.00003
7	Unidentified	-	-	5.0/34.0	-	-	7/11	−5.33	0.0047
8	stathmin 1 (STMN1)	P16949	6.1/20.5	5.75/17.3	17	29.5	6/11	−1.88	0.008
9	toll interacting protein (TOLLIP)	Q9H0E2	5.8/33.1	5.68/30.3	8	23.4	6/11	−3.52	0.04
10	protein CWC15 homolog (CWC15)	Q9P013	5.6/26.6	5.7/32.3	5	19	6/11	−3.35	0.05
11	sorting nexin 9 (SNX9)	Q9Y5X1	5.5/83.4	5.4/66.6	11	23	6/11	−2.98	0.00004
12	scavenger mRNA-decapping enzyme (DCPS)	Q96C86	6.5/37.3	5.93/38.6	12	32.3	6/11	−2.82	0.028
13	fatty acid binding protein 4 (FABP 4)	P15090	6.5/16.6	7.93/15.2	5	41.9	10/11	−5.16	0.00003
14	thioredoxin reductase 1 (TRXR1)	Q16881	6.6/58.3	6.07/54.4	13	24.1	6/11	+4.32	0.00001
15	alcohol dehydrogenase [NADP+] (AKR1A1)	P14550	6.9/37.5	6.32/36.6	14	33.2	6/11	−2.24	0.005
16	*N*-acetylneuraminate synthase (sialic acid synthase) (NANS)	Q9NR45	6.9/37.4	6.29/40.3	7	22.6	7/11	−2.3	0.005
17	glutamine synthetase (GS)	P15104	6.8/41.4	6.43/42.1	7	14.7	9/11	−5.02	0.0001

^a^ HUPO Gene Nomenclature Committee approved symbol; ^b^ Theo.: Theoretical; ^c^ Exper.: Experimental; ^d^ Matched peptide number: Number of peptides matched with protein in MS/MS query. The detailed data of MS/MS identification for each peptide is provided in Supplementary Table S1, ^e^ Coverage: Total percentage of amino acid sequence covered by peptides identified by MS/MS analyses; ^f^ Fold difference: the difference between mean of normalized volume of Sh-Gal-1(+120) and Sc-Gal-1(+120) cells.

**Table 2:** Correlations between Galectin-1 Expression and other important clinicopathological, parameters in UBUC.

Parameter	Category	Urinary Bladder Urothelial Carcinoma			
		Case No.	Galectin-1 Expression		*p*-Value
			Low	High	
Gender	Male	216	106	110	0.667
	Female	79	41	38	
Age (years)	<65	121	69	52	0.039 *
	≥65	174	78	96	
Primary tumor (T)	Ta	84	66	18	<0.001 *
	T1	88	51	37	
	T2–T4	123	30	93	
Nodal metastasis	Negative (N0)	266	139	127	0.012 *
	Positive (N1-N2)	29	8	21	
Histological grade	Low grade	56	43	13	<0.001 *
	High grade	239	104	135	
Vascular invasion	Absent	246	138	108	<0.001 *
	Present	49	9	40	
Perineural invasion	Absent	275	144	131	0.001 *
	Present	20	3	17	
Mitotic rate (per 10 high power fields)	<10	139	84	55	0.001 *
	≥10	156	63	93	
GS Expression	Low Exp.	147	115	32	<0.001 *
	High Exp.	148	32	116	
FABP 4 Expression	Low Exp.	147	111	36	<0.001 *
	High Exp.	148	36	112	
TOLLIP Expression	Low Exp.	147	97	50	<0.001 *
	High Exp.	148	50	98	
AKR1A1 Expression	Low Exp.	147	111	36	<0.001 *
	High Exp.	148	36	112	

* Statistically significant.

**Table 3:** Univariate log-rank and multivariate analyses for Disease-specific and Metastasis-free Survivals in urinary bladder urothelial carcinoma.

Parameter	Category	Case No.	Disease-Specific Survival					Metastasis-Free Survival				
			Univariate Analysis		Multivariate Analysis			Univariate Analysis		Multivariate Analysis		
			No. of Event	*p*-Value	R.R. ^a^	95% C.I.	*p*-Value	No. of Event	*p*-Value	R.R.	95% C.I.	*p*-Value
Gender	Male	216	39	0.5392	-	-	-	59	0.2999	-	-	-
	Female	79	11		-	-	-	16		-	-	-
Age (years)	<65	121	16	0.0972	-	-	-	30	0.5912	-	-	-
	≥65	174	34		-	-	-	45		-	-	-
Primary tumor (T)	Ta	84	1	<0.0001 *	1	-	0.003 *	4	<0.0001 *	1	-	0.033 *
	T1	88	9		2.732	1.232–6.061		23		4.181	1.996–2.591	
	T2–T4	123	40		9.346	1.027–83.333		48		5.543	1.961–23.810	
Nodal metastasis	Negative (N0)	266	41	0.0037 *	1	-	0.640	61	<0.0001 *	1	-	0.086
	Positive (N1-N2)	29	9		1.199	0.561–2.561		14		1.746	0.924–3.303	
Histological grade	Low grade	56	2	0.0017 *	1	-	0.703	5	0.0008 *	1	-	0.807
	High grade	239	48		1.359	0.282–6.558		75		0.873	0.293–2.598	
Vascular invasion	Absent	246	36	0.0052 *	1	-	0.209	54	0.0003 *	1	-	0.994
	Present	49	14		1.616	0.765–3.413		21		0.998	0.521–1.911	
Perineural invasion	Absent	275	44	0.0085 *	1	-	0.353	65	0.0006 *	1	-	0.202
	Present	20	6		1.586	0.599–4.196		10		1.646	0.765–3.542	
Mitotic rate (per 10 high power fields)	<10	139	12	<0.0001 *	1	-	0.044 *	22	<0.0001 *	1	-	0.021 *
	≥10	156	38		2.048	1.020–4.109		53		1.885	1.103–3.224	
Galectin 1 expression	High Exp.	147	4	<0.0001 *	1	-	0.007 *	16	<0.0001 *	1	-	0.012
	Low Exp.	148	46		4.628	1.5.5–14.225		59		2.386	1.215–4.685	
GS expression	Low Exp.	147	11	0.0001 *	1	-	0.867	27	0.0097 *	1	-	0.354
	High Exp.	148	39		1.066	0.504–2.254		48		0.763	0.431–1.351	
FABP 4 expression	Low Exp.	147	10	<0.0001 *	1	-	0.405	25	0.0002 *	1	-	0.904
	High Exp.	148	40		1.378	0.648–2.927		50		1.035	0.588–1.822	
TOLLIP expression	Low Exp.	147	28	0.3415	-	-	-	34	0.5027	-	-	-
	High Exp.	148	22		-	-	-	41		-	-	-
AKR1A1 expression	Low Exp.	147	21	0.2467	-	-	-	30	0.0779	-	-	-
	High Exp.	148	29		-	-	-	45		-	-	-

* Statistically significant. ^a^ R. R., relative risk.

## Supplementary Materials

The following are available online at http://www.mdpi.com/1422-0067/19/4/1242/s1.

## Abbreviations

UBUC	Urinary bladder urothelial carcinoma
IHC	Immunohistochemistry
MMP	Matrix metalloproteinase protein
OSCC	Oral squamous cell carcinoma
2-DE	Two-dimensional gel electrophoresis
LC-MS/MS	Liquid chromatography/tandem mass spectrometry
PPAR-γ	Peroxisome proliferator-activated receptor gamma
RNH1	Ribonuclease/angiogenin inhibitor 1
RCN1	Reticulocalbin 1
LGALS1	Galectin 1
TBCA	Tubulin specific chaperone A
UBE2K	Ubiquitin conjugating enzyme E2 K
PMF1	Polyamine-modulated factor 1
STMN1	Stathmin 1
TOLLIP	toll interacting protein
CWC15	protein CWC15 homolog
SNX9	sorting nexin 9
DCPS	Scavenger mRNA-decapping enzyme
FABP 4	fatty acid binding protein 4
TRXR1	thioredoxin reductase 1
AKR1A1	Alcohol dehydrogenase (NADP^+^)
NANS	N-acetylneuraminate synthase (sialic acid synthase)
GS	glutamine synthetase
R. R.	relative risk
