# Supplementary material for: Proteomic Identification of the Galectin-1-Involved Molecular Pathways in Urinary Bladder Urothelial Carcinoma
Source: Int J Mol Sci. 2018 Apr 19;19(4):1242. doi: 10.3390/ijms19041242 (PMC5979315; doi:10.3390/ijms19041242)

## Slide 1
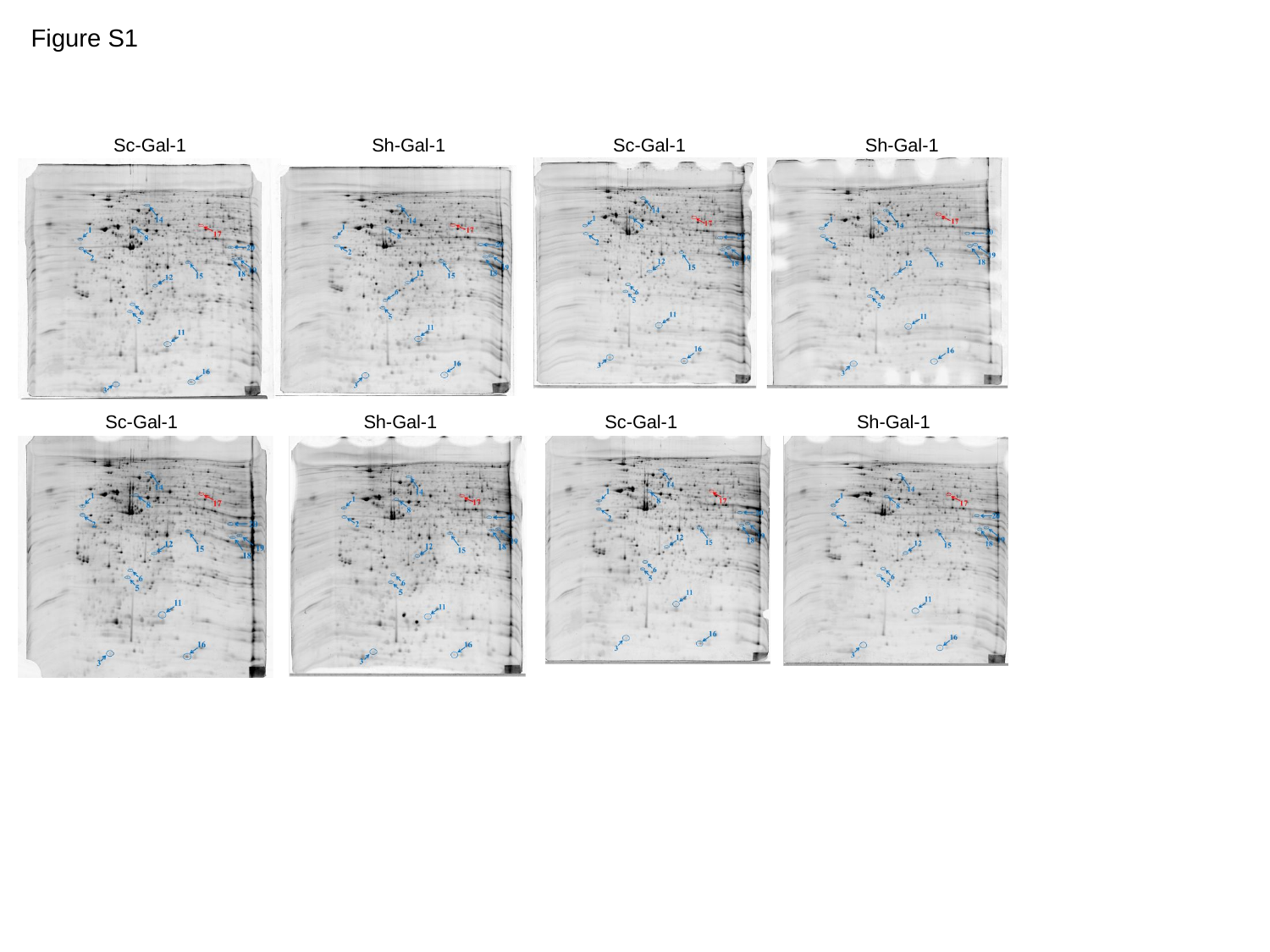

Figure S1
Sc-Gal-1
Sh-Gal-1
Sc-Gal-1
Sh-Gal-1
Sc-Gal-1
Sh-Gal-1
Sc-Gal-1
Sh-Gal-1

## Slide 2
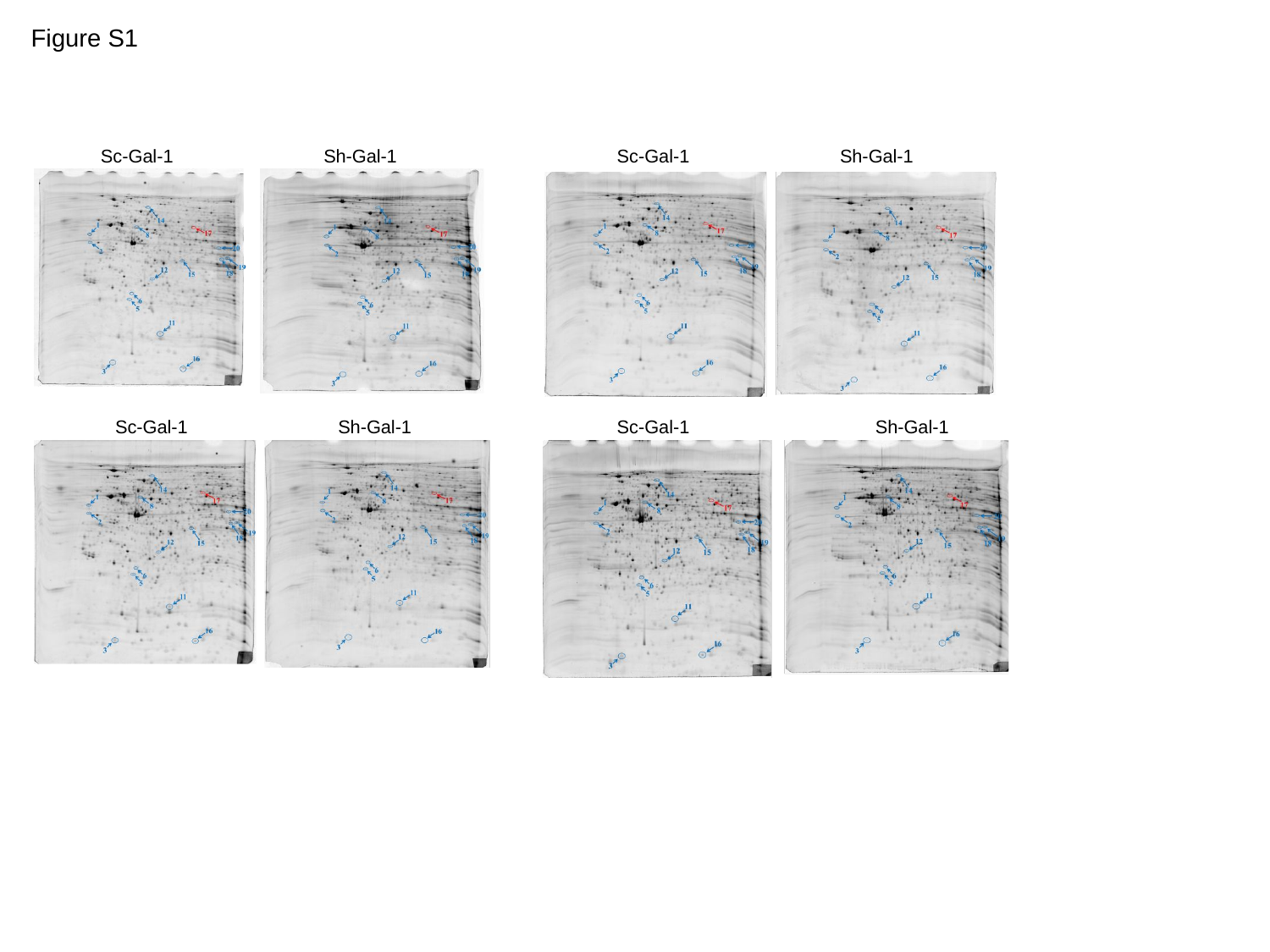

Figure S1
Sc-Gal-1
Sh-Gal-1
Sc-Gal-1
Sh-Gal-1
Sc-Gal-1
Sh-Gal-1
Sc-Gal-1
Sh-Gal-1

## Slide 3
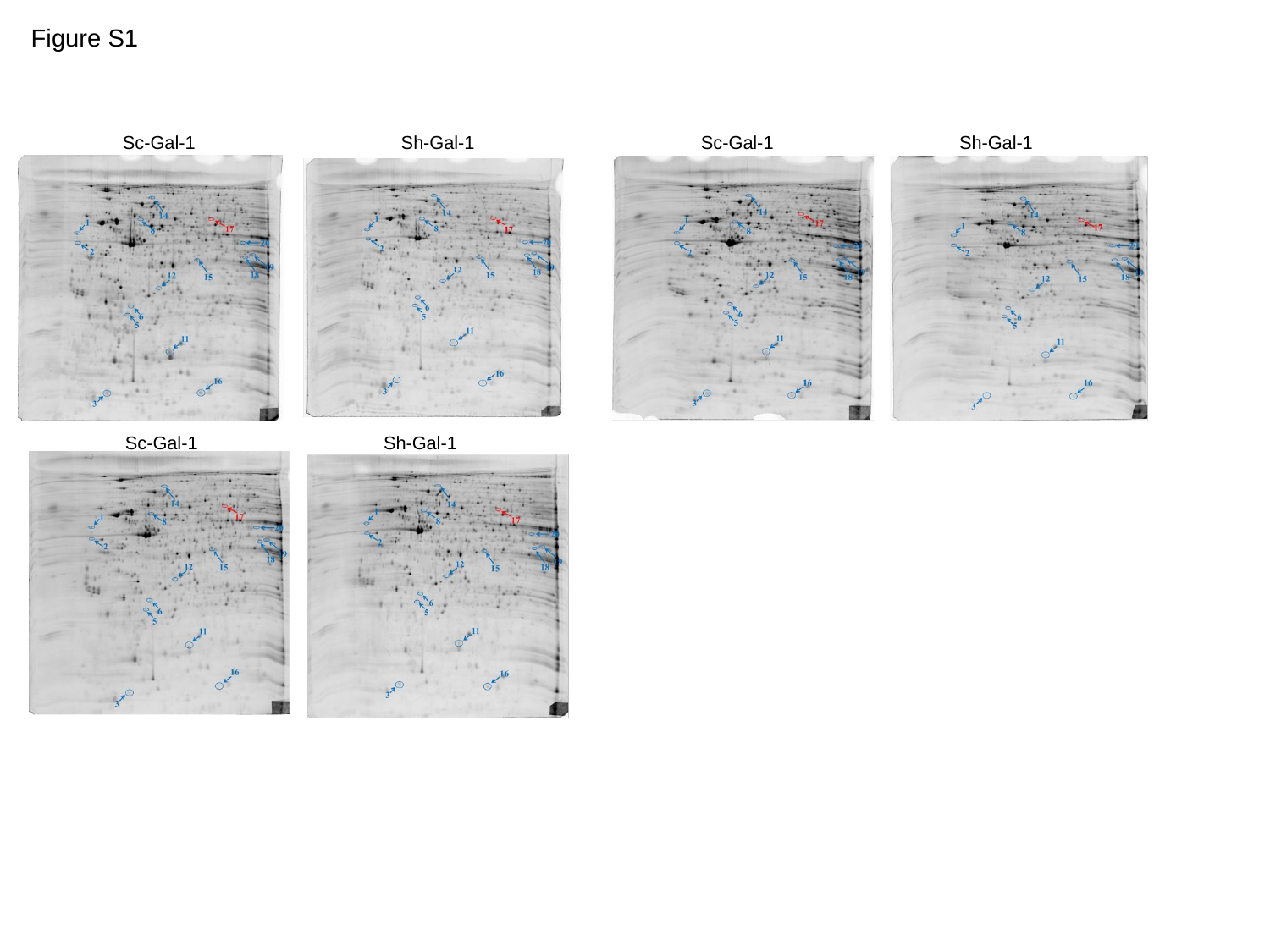

Figure S1
Sc-Gal-1
Sh-Gal-1
Sc-Gal-1
Sh-Gal-1
Sc-Gal-1
Sh-Gal-1

## Slide 4
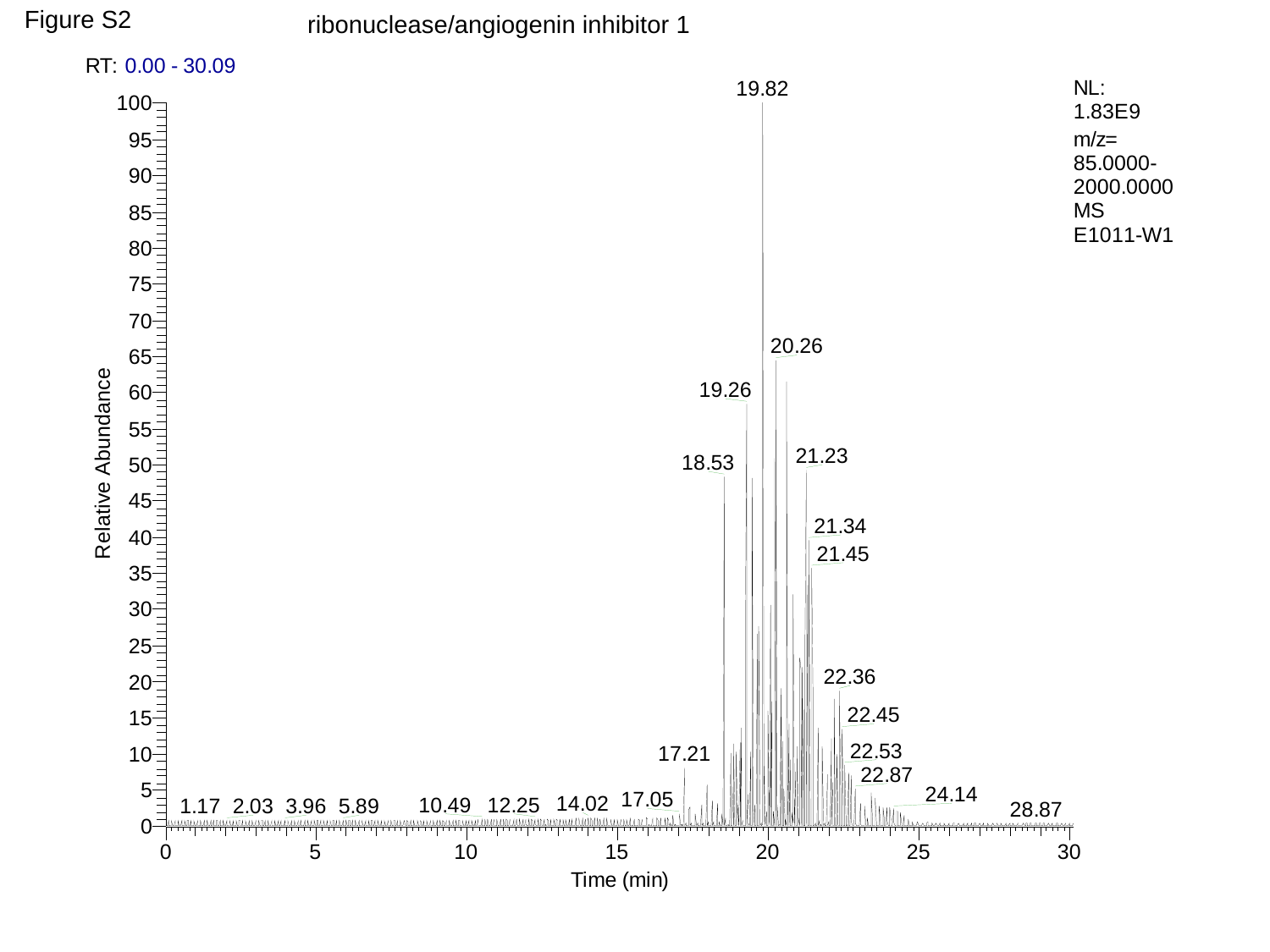

Figure S2
ribonuclease/angiogenin inhibitor 1

## Slide 5
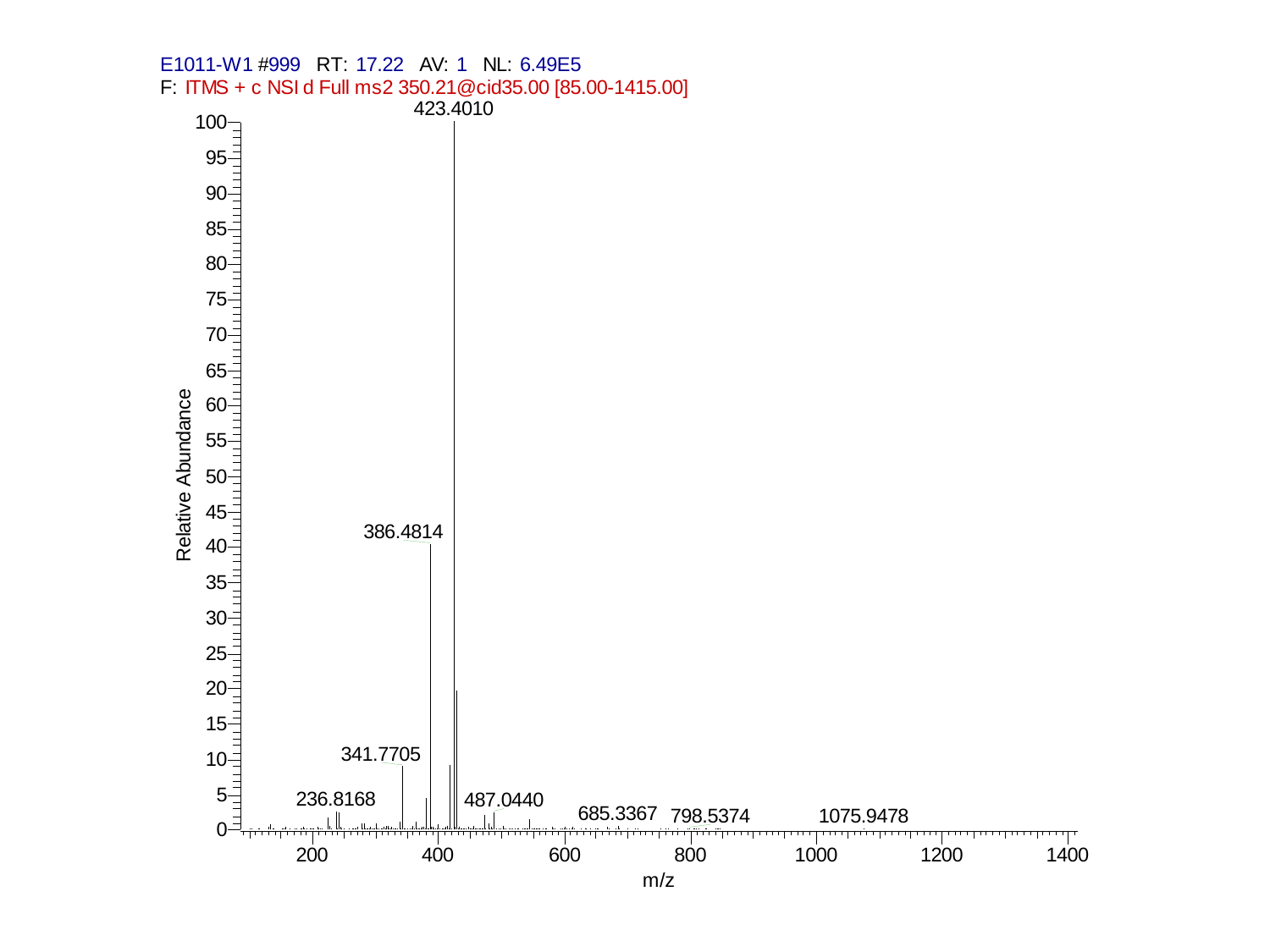

## Slide 6
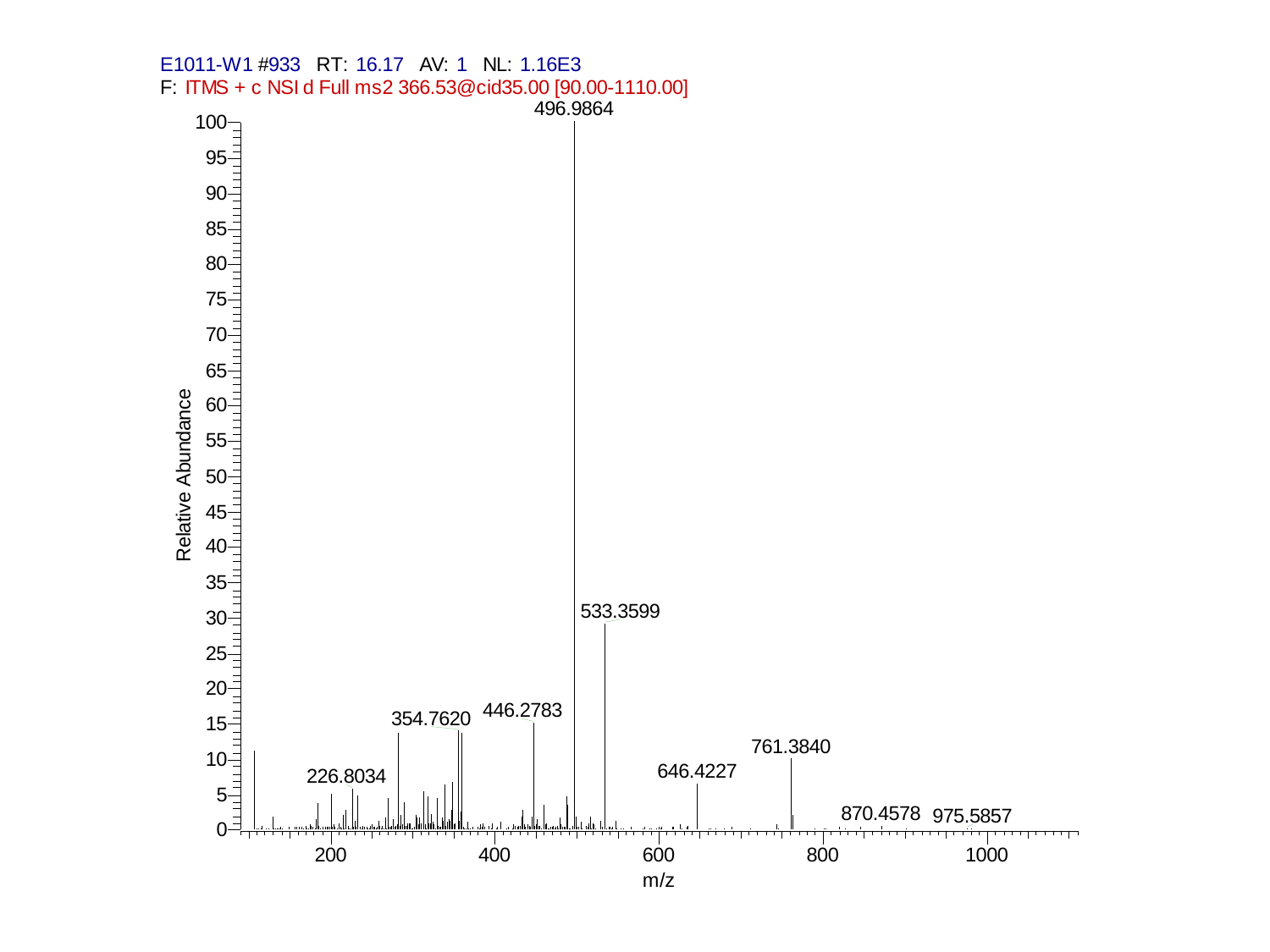

## Slide 7
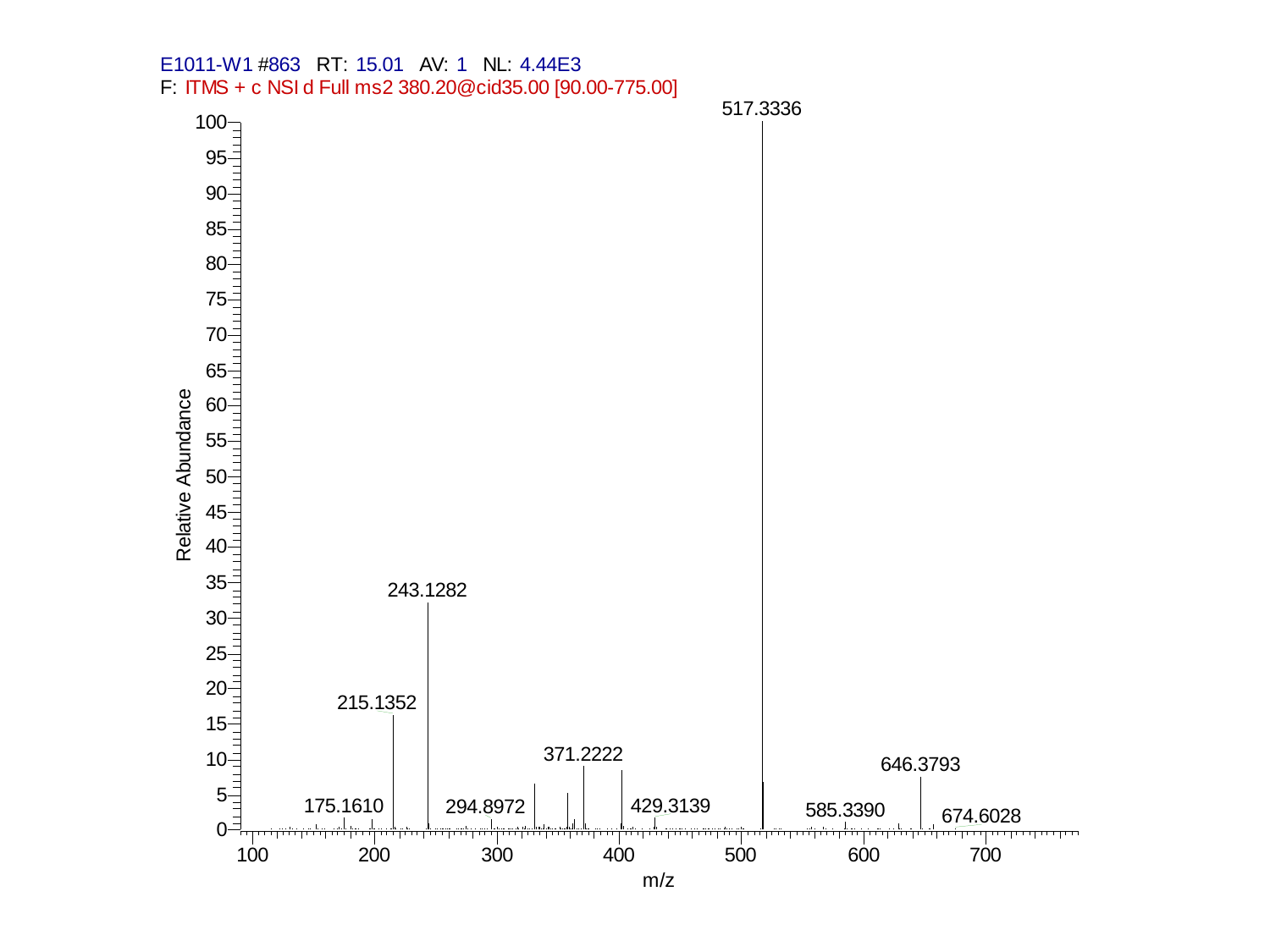

## Slide 8
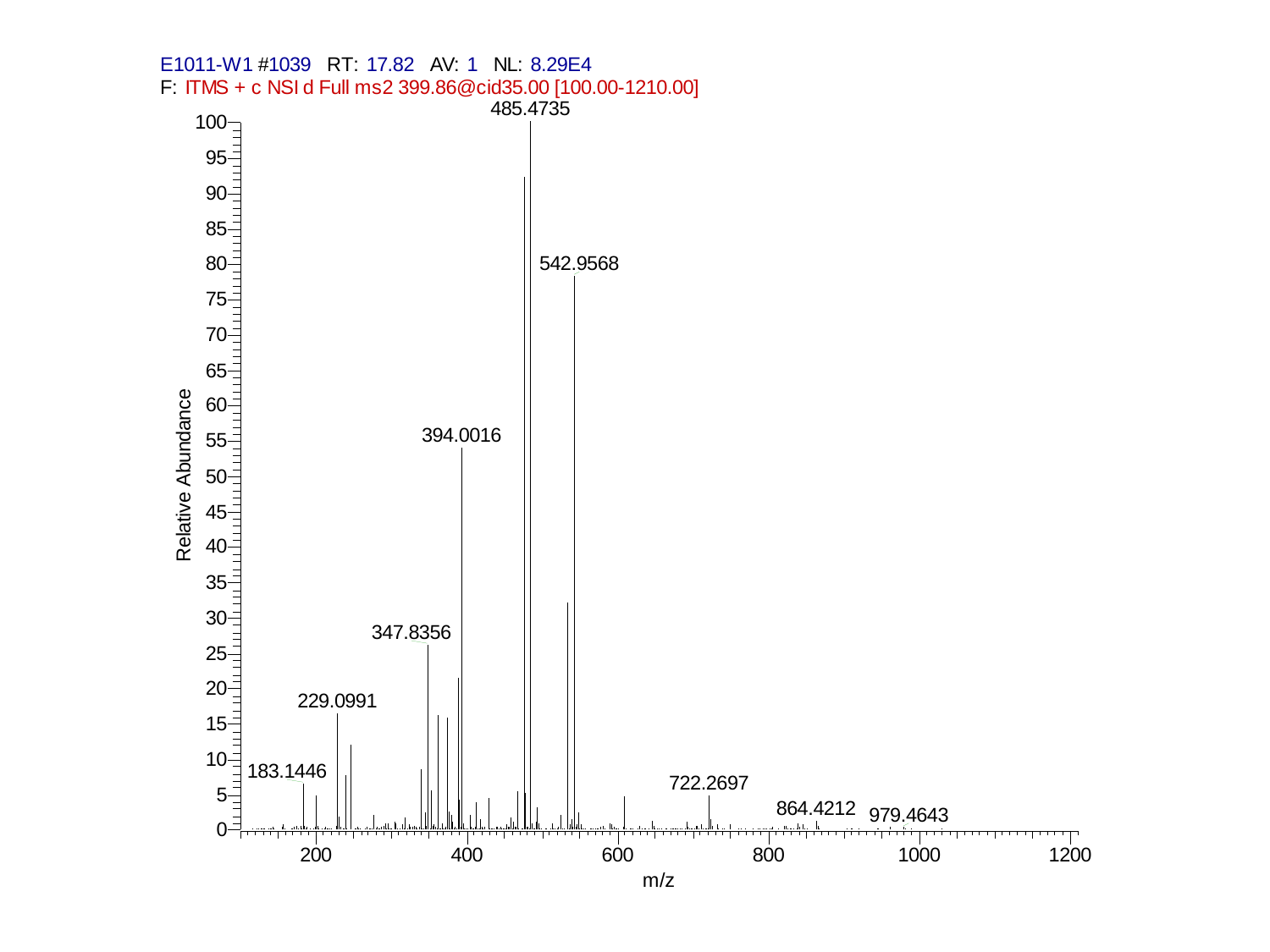

## Slide 9
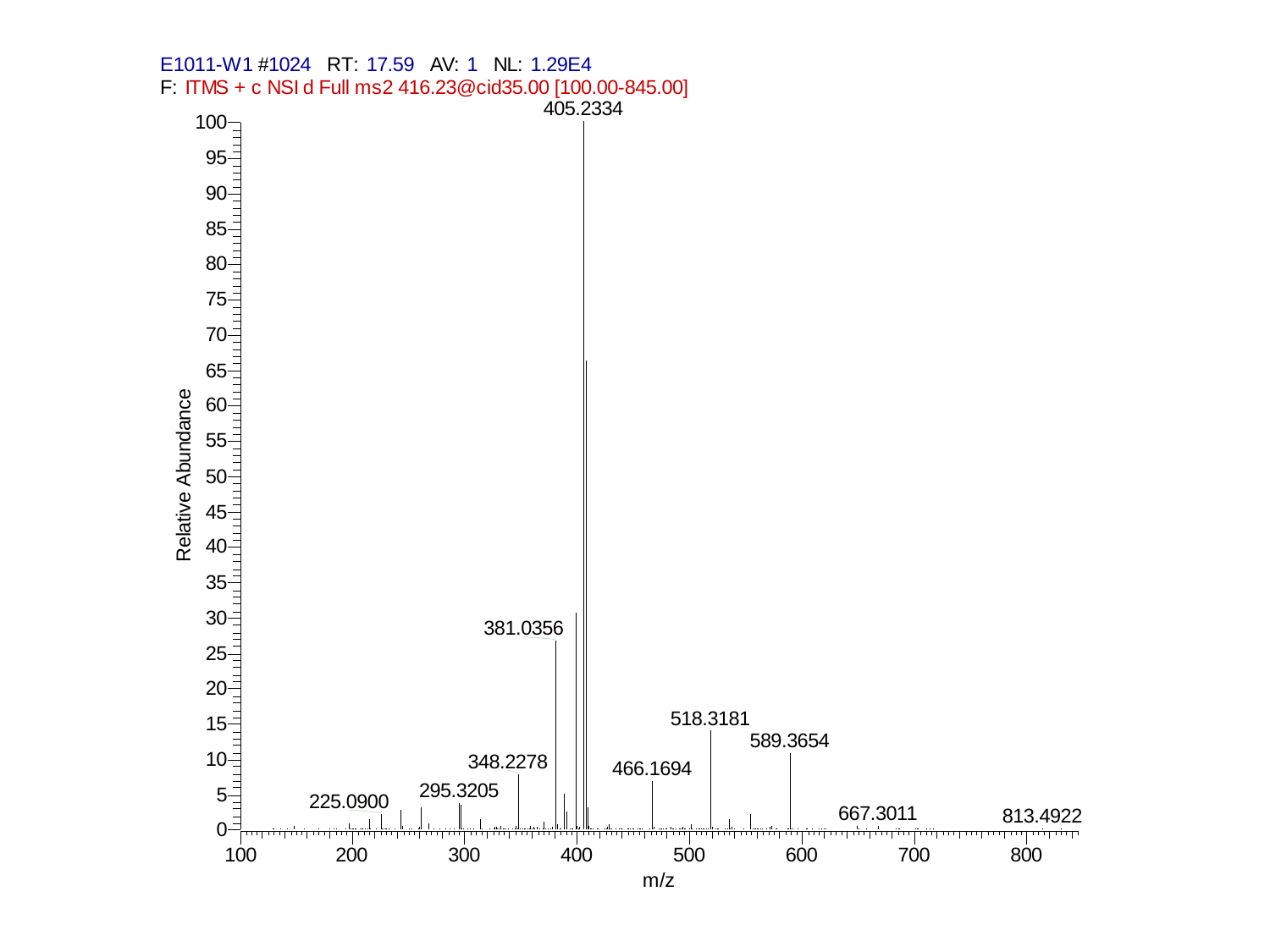

## Slide 10
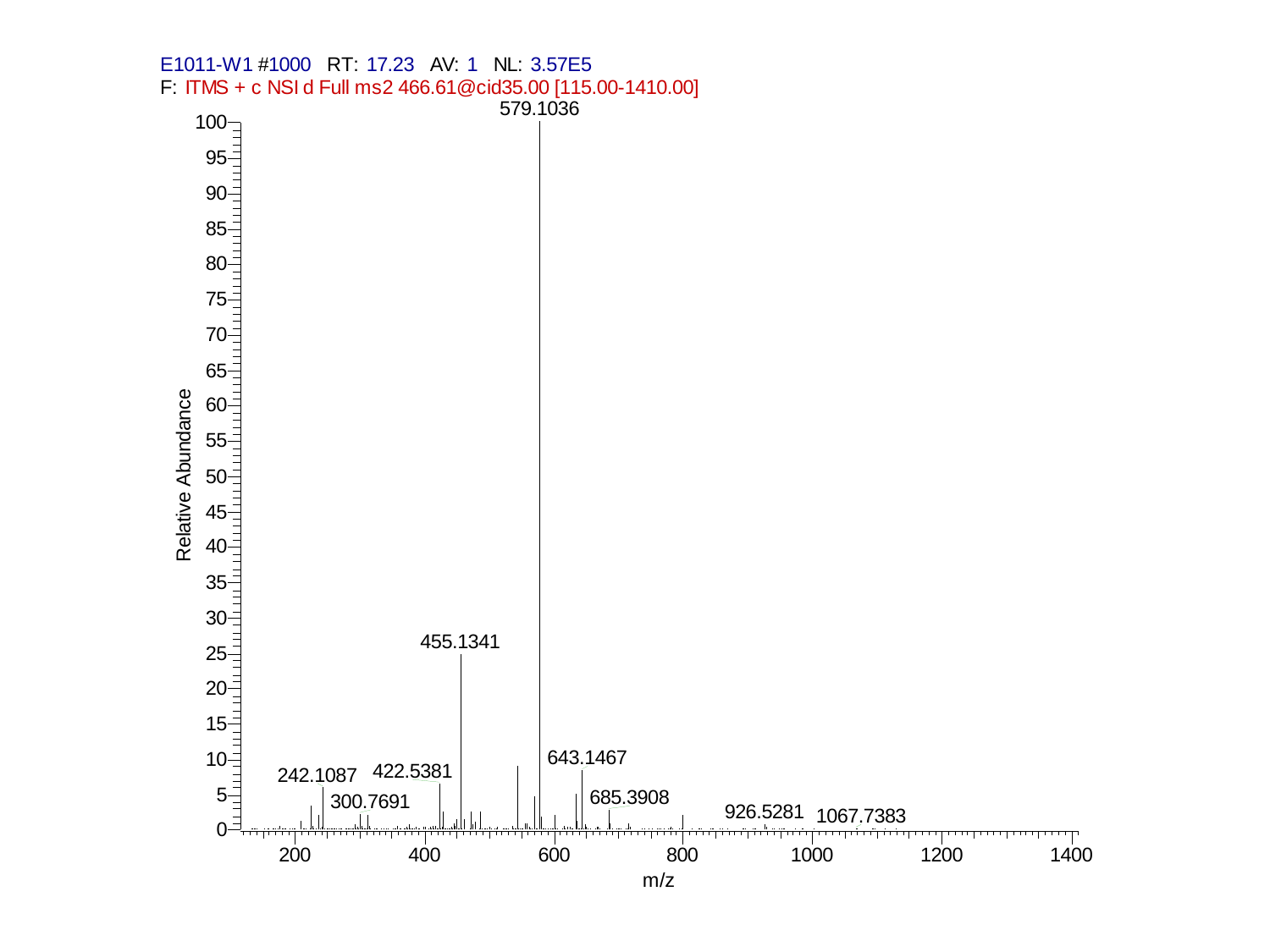

## Slide 11
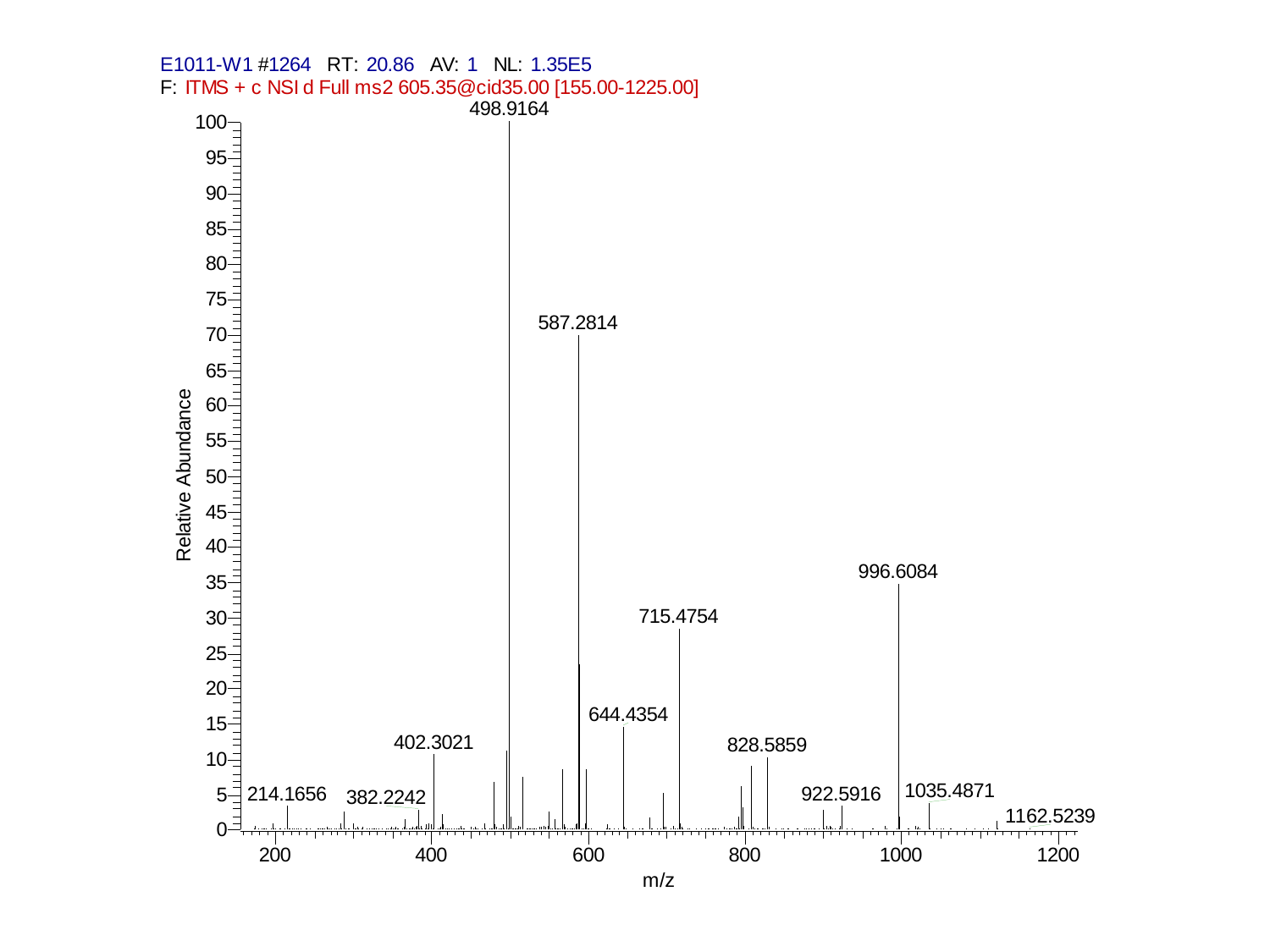

## Slide 12
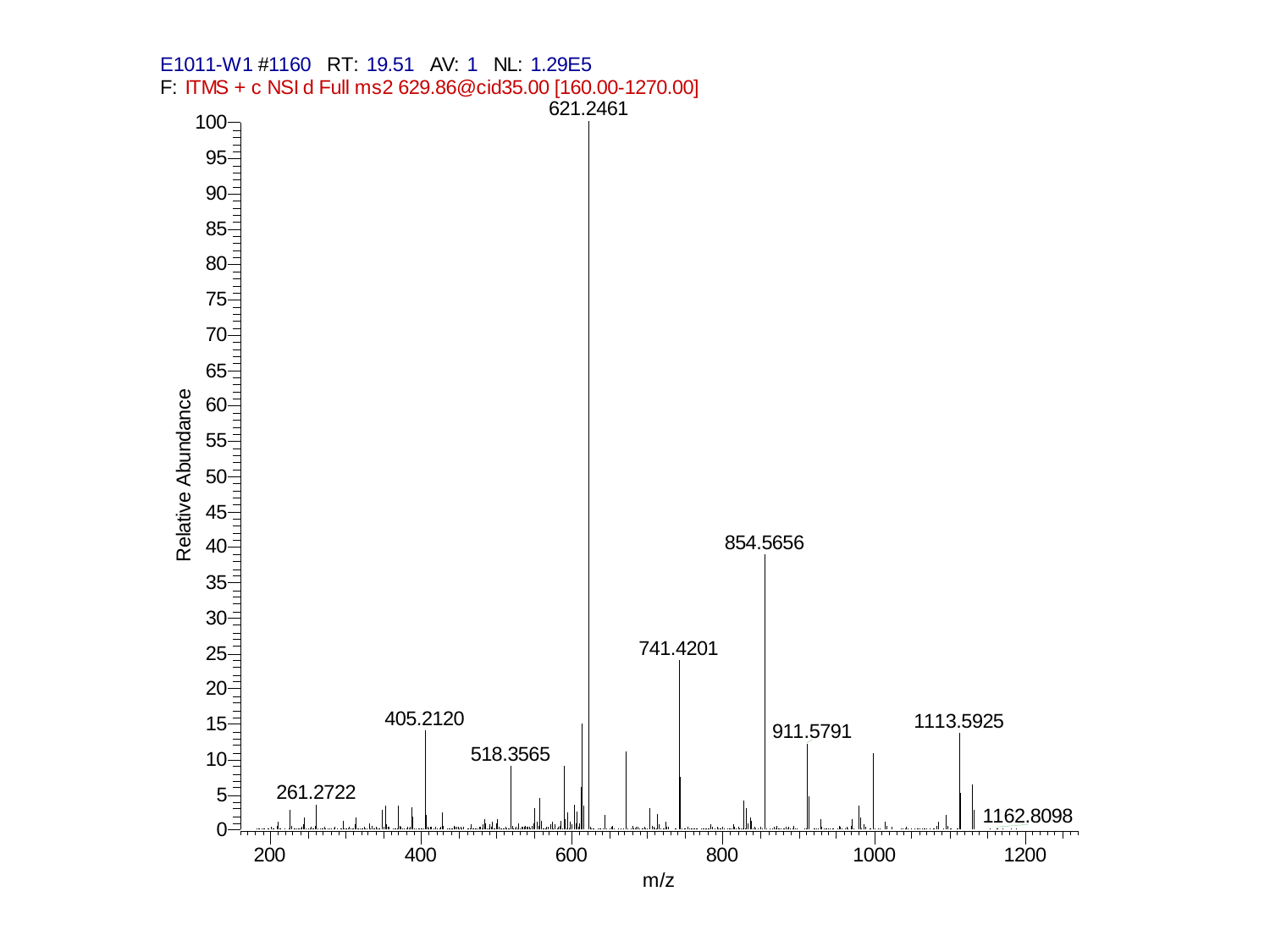

## Slide 13
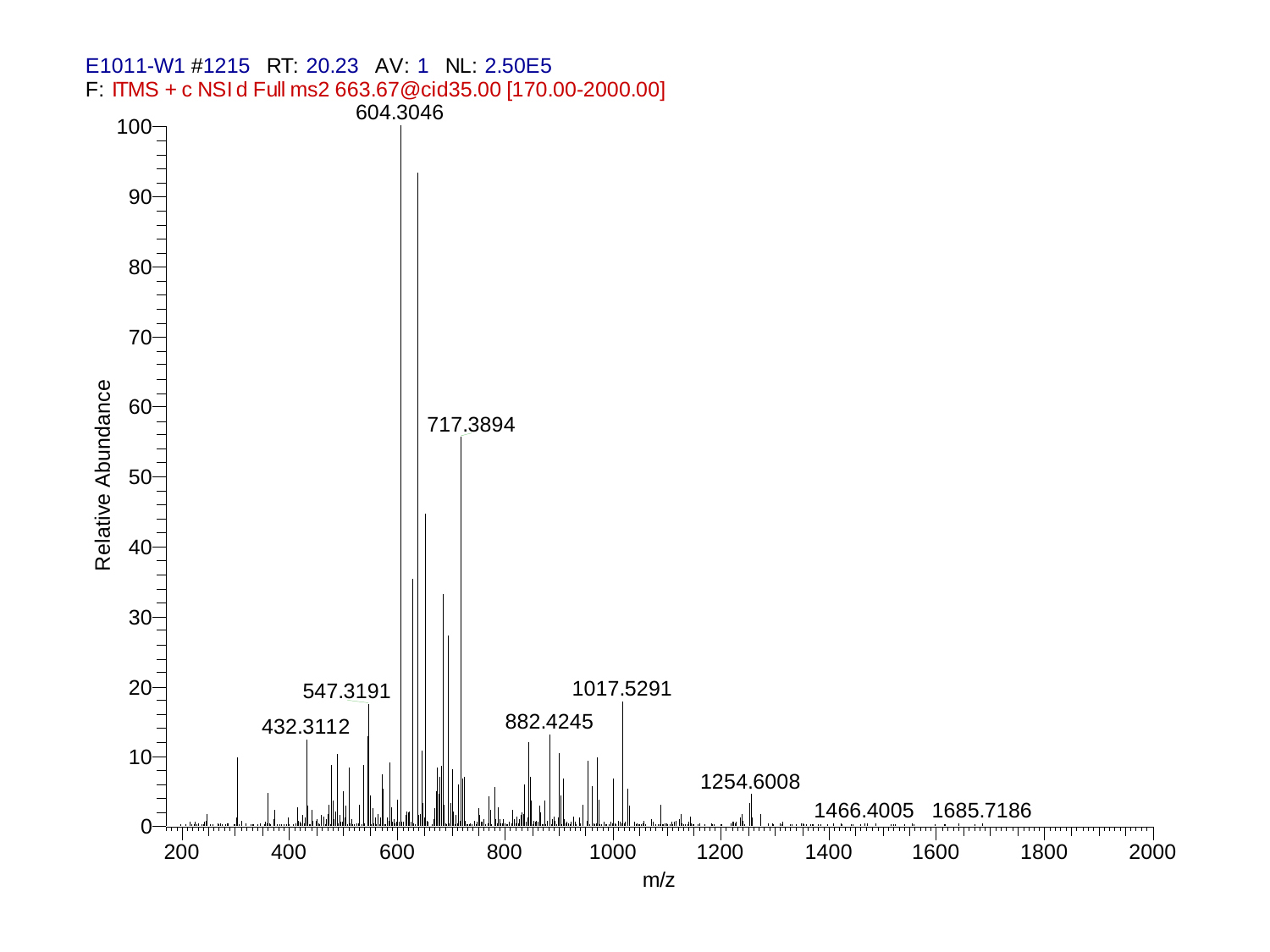

## Slide 14
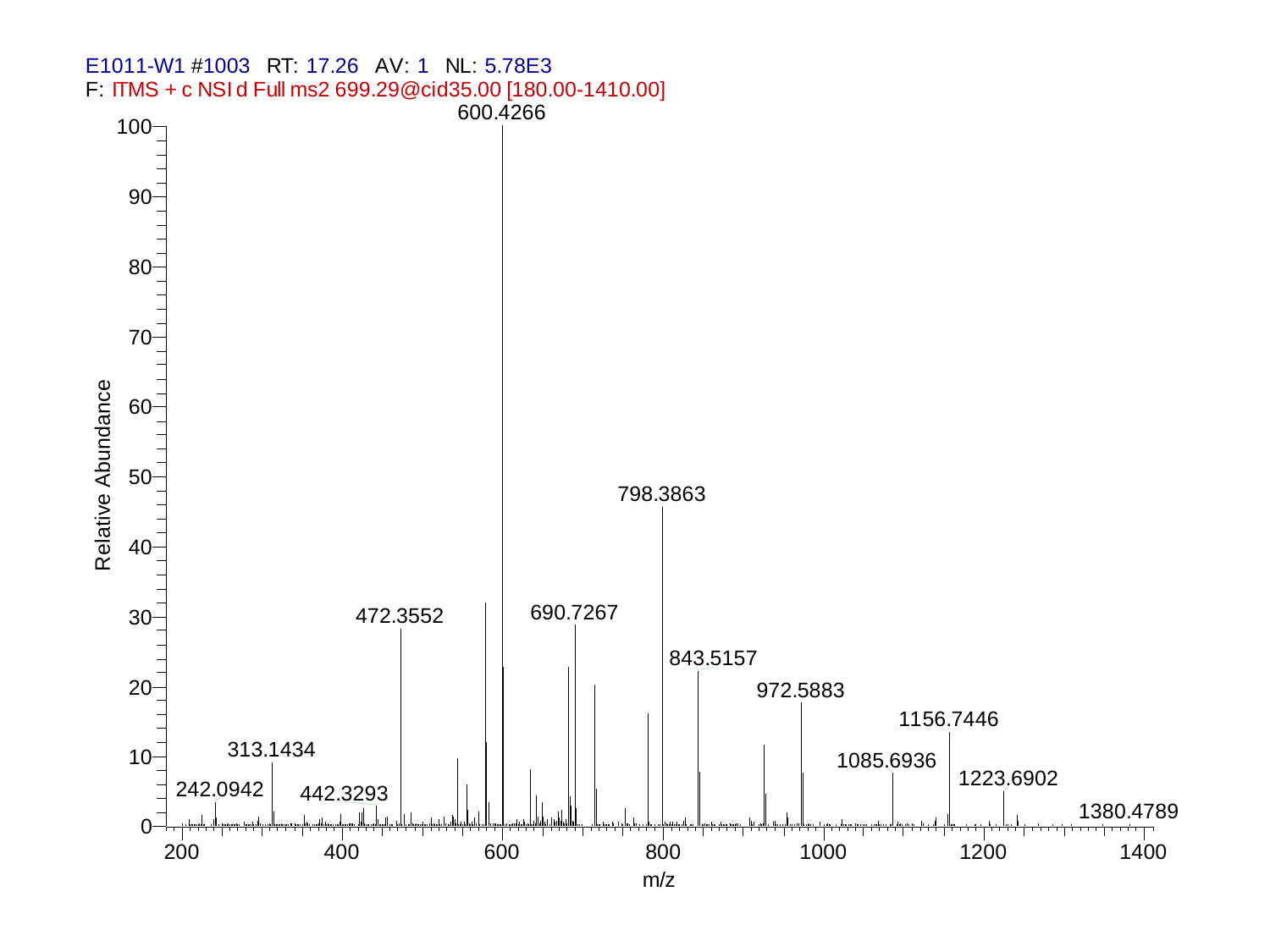

## Slide 15
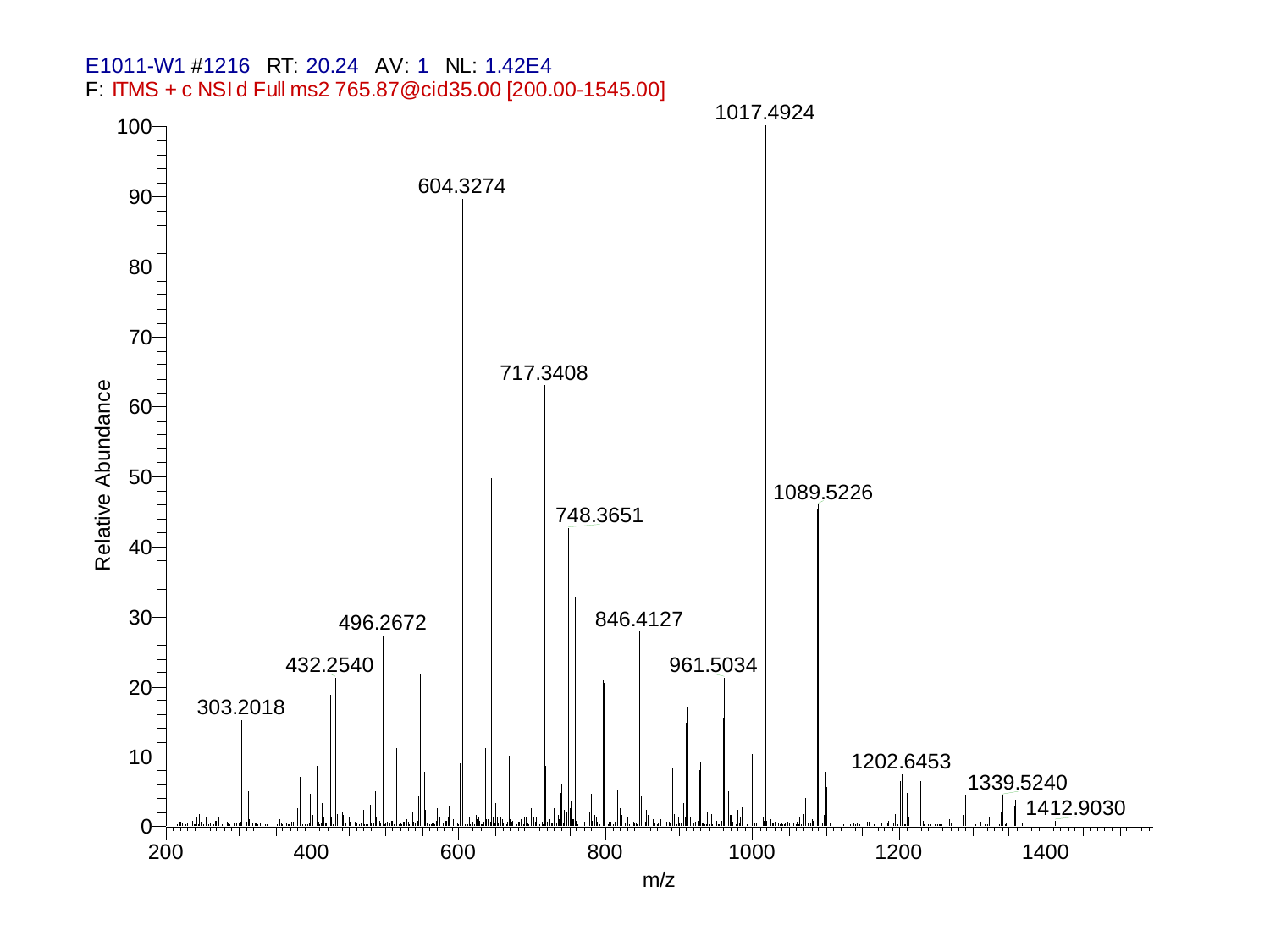

## Slide 16
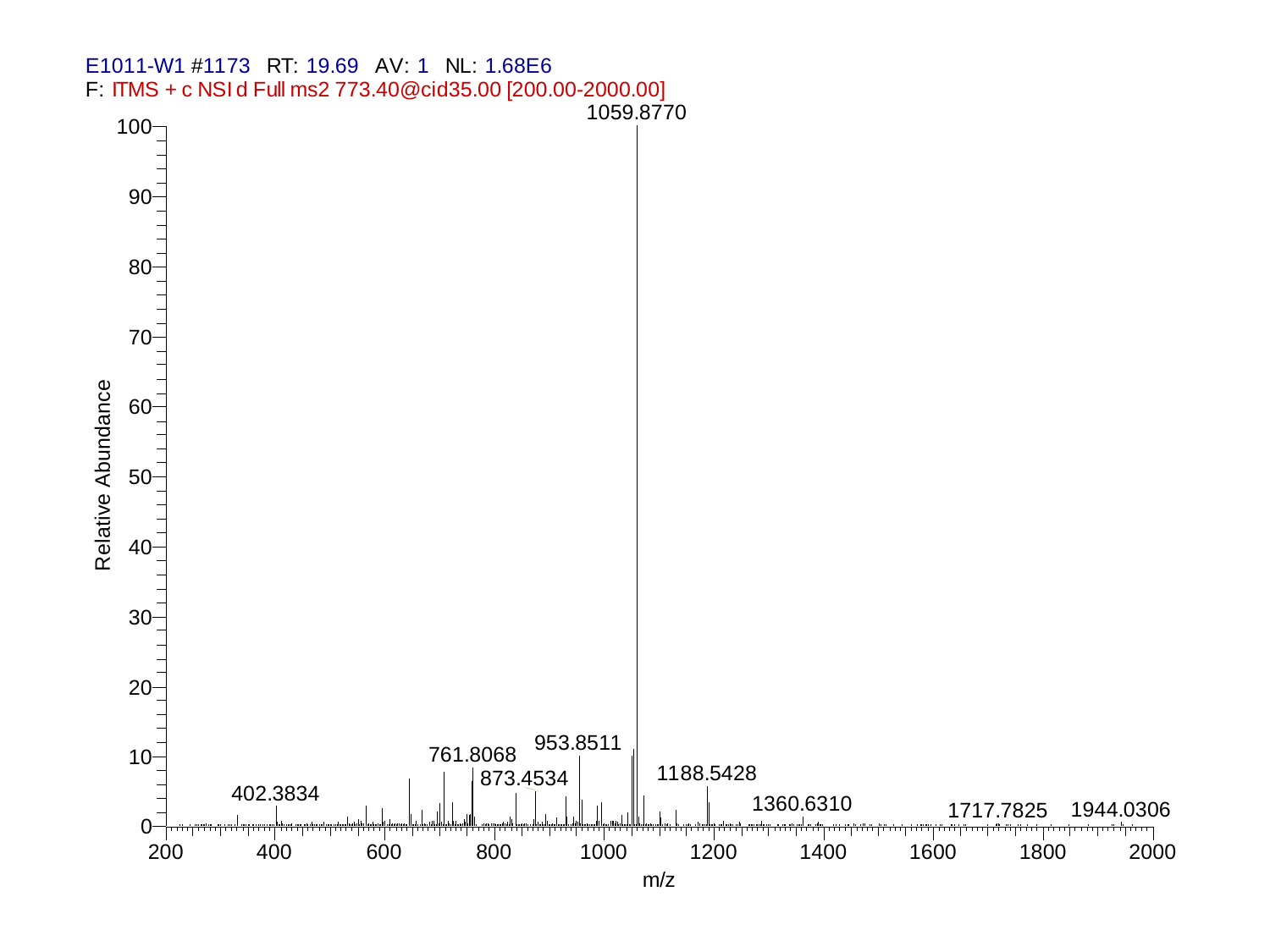

## Slide 17
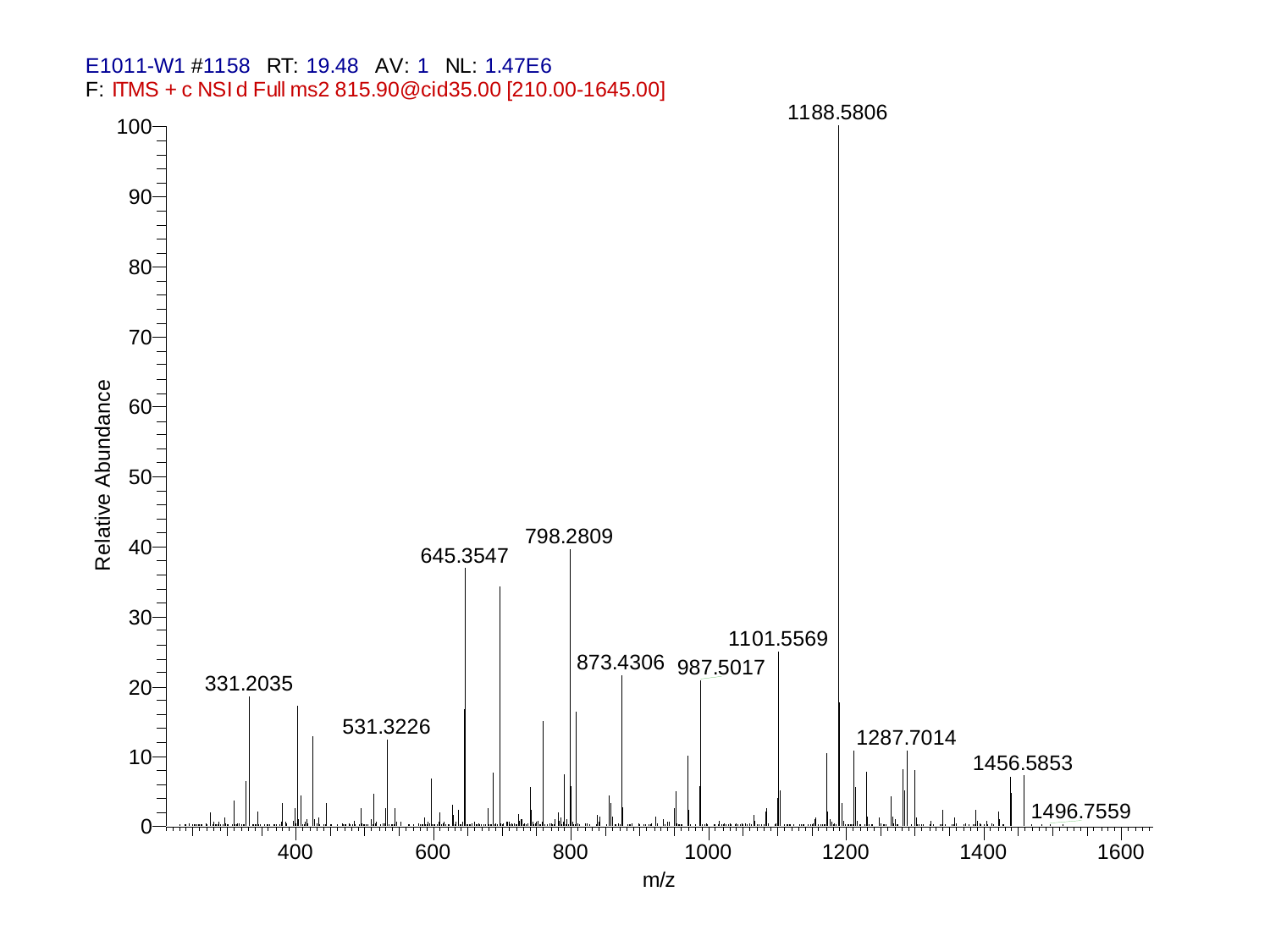

## Slide 18
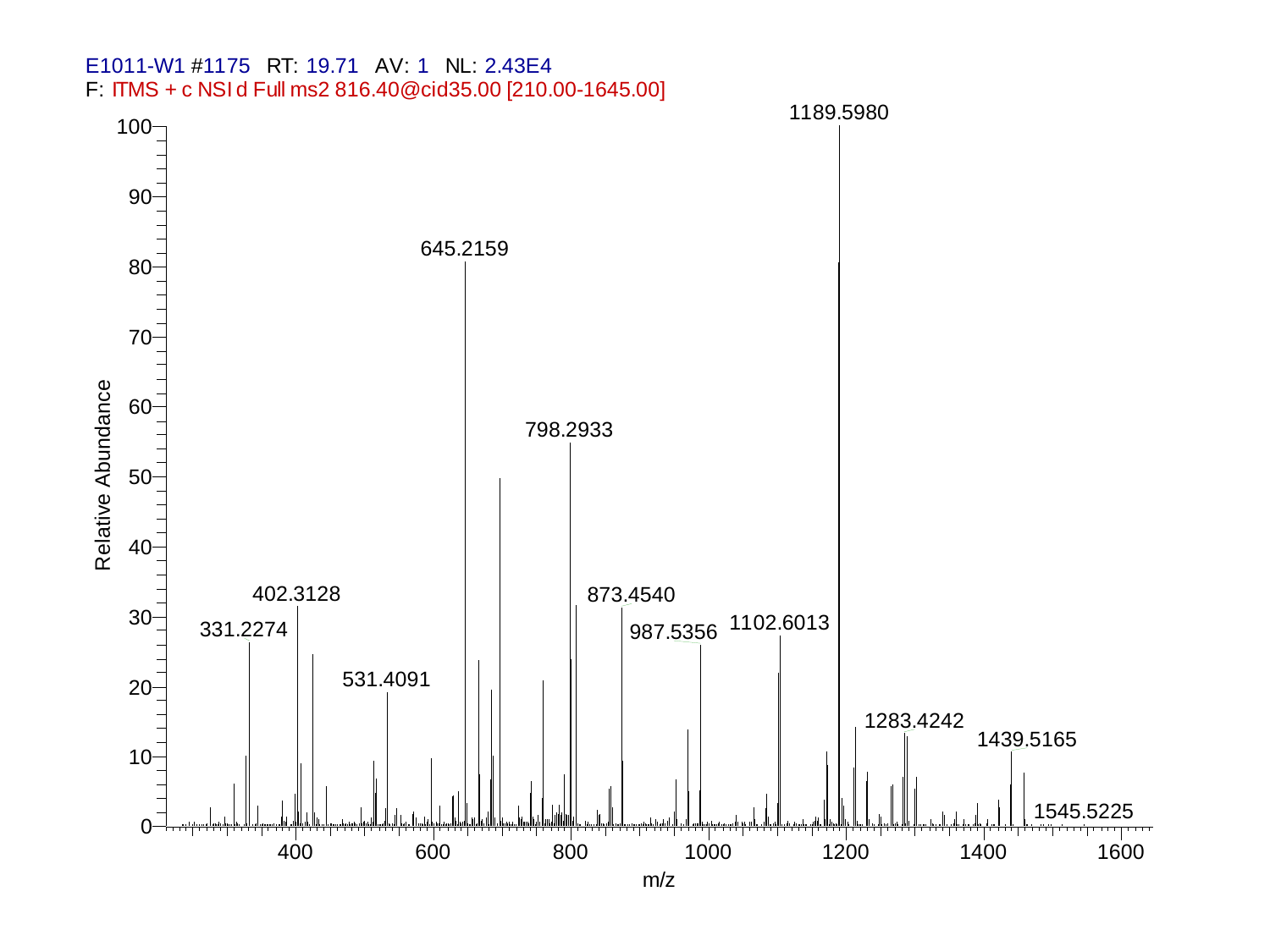

## Slide 19
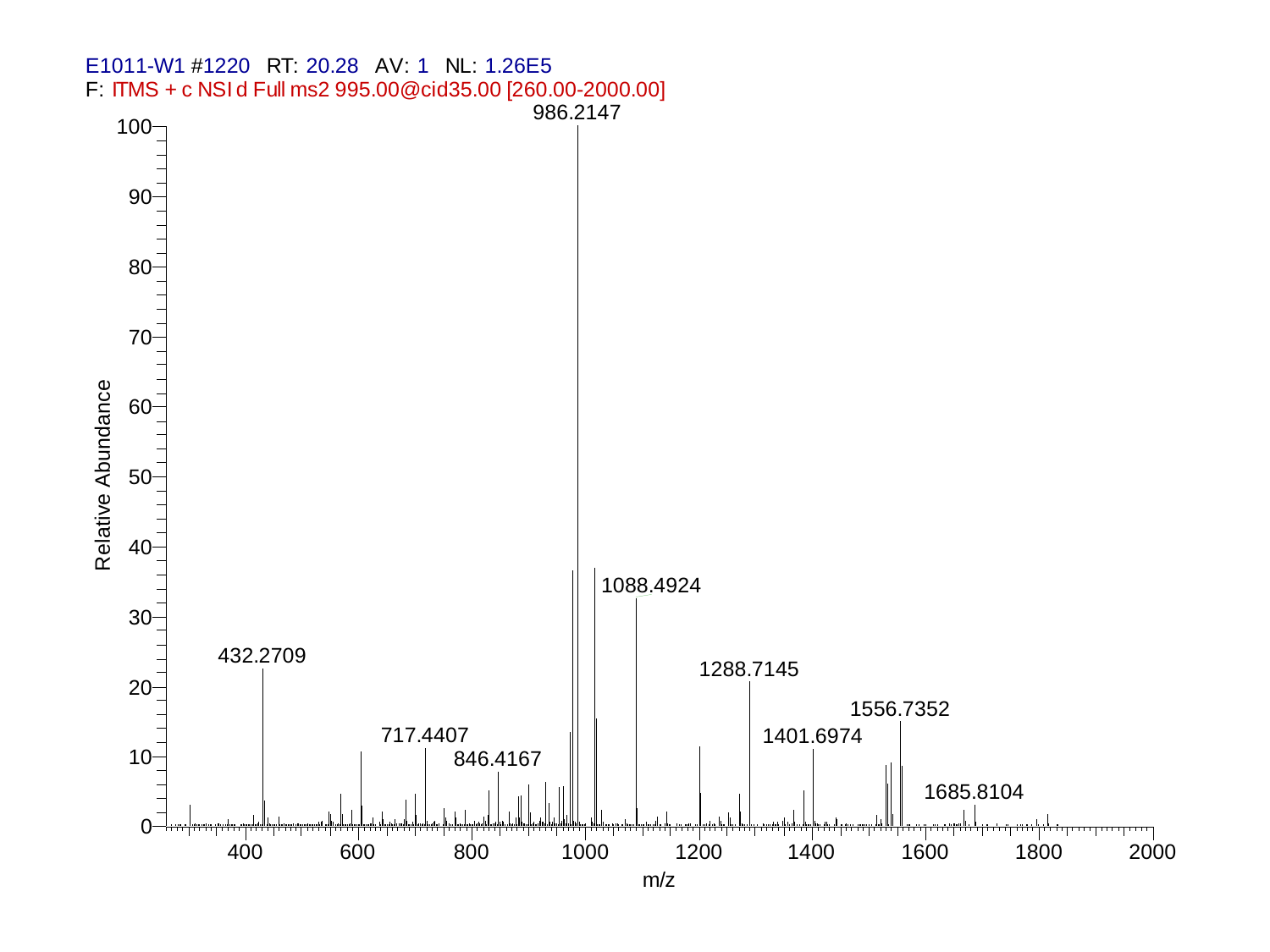

## Slide 20
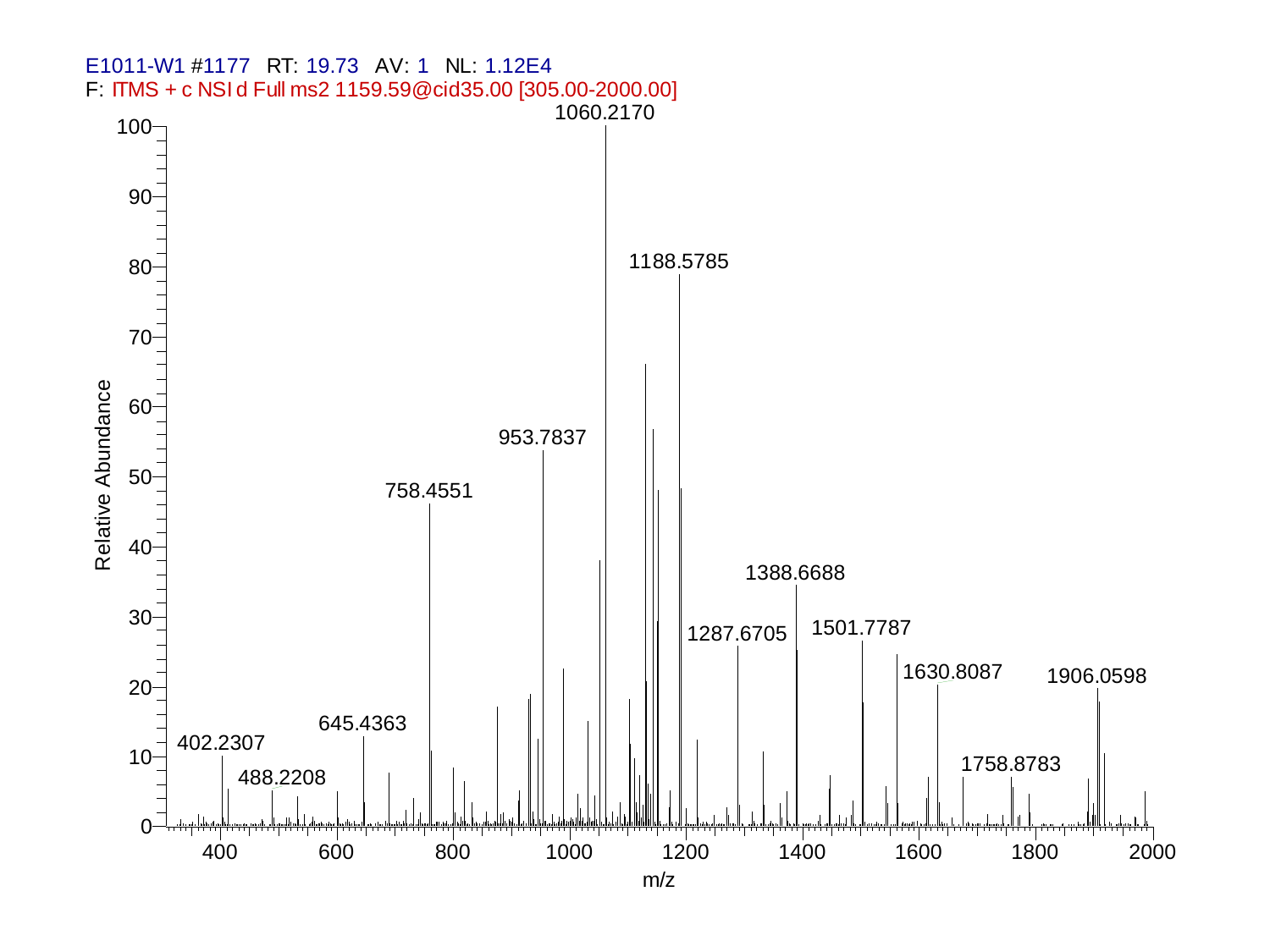

## Slide 21
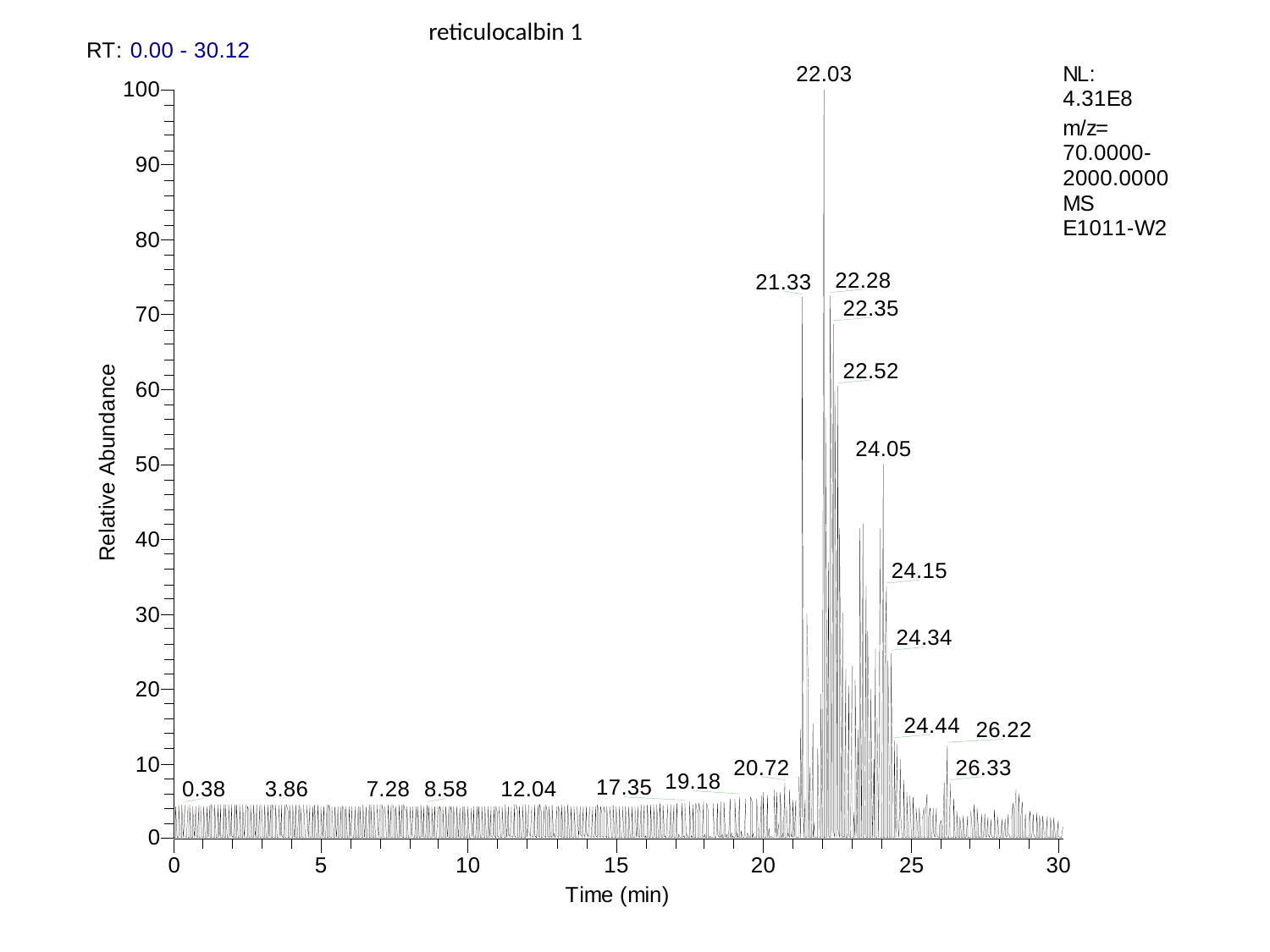

reticulocalbin 1

## Slide 22
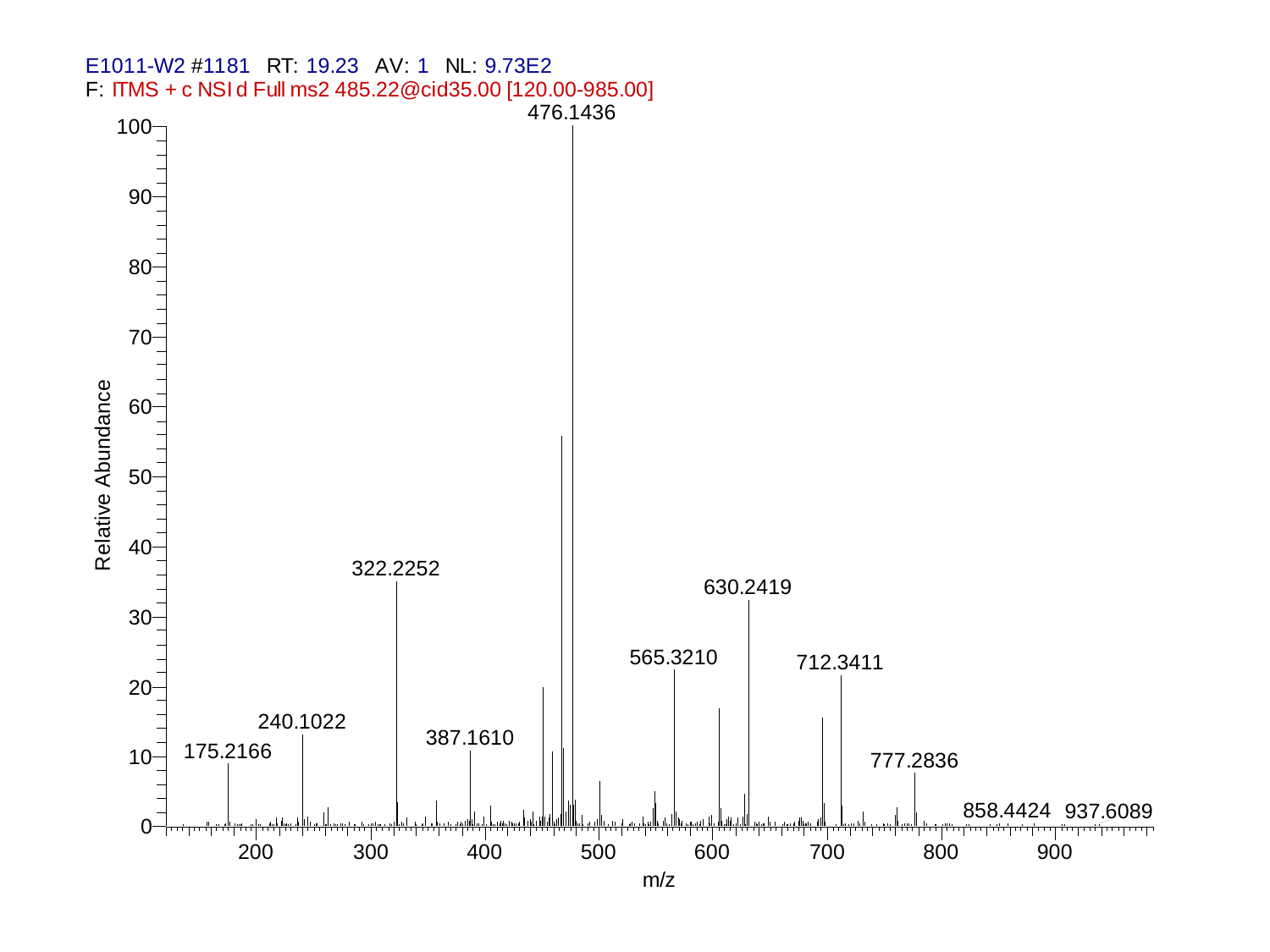

## Slide 23
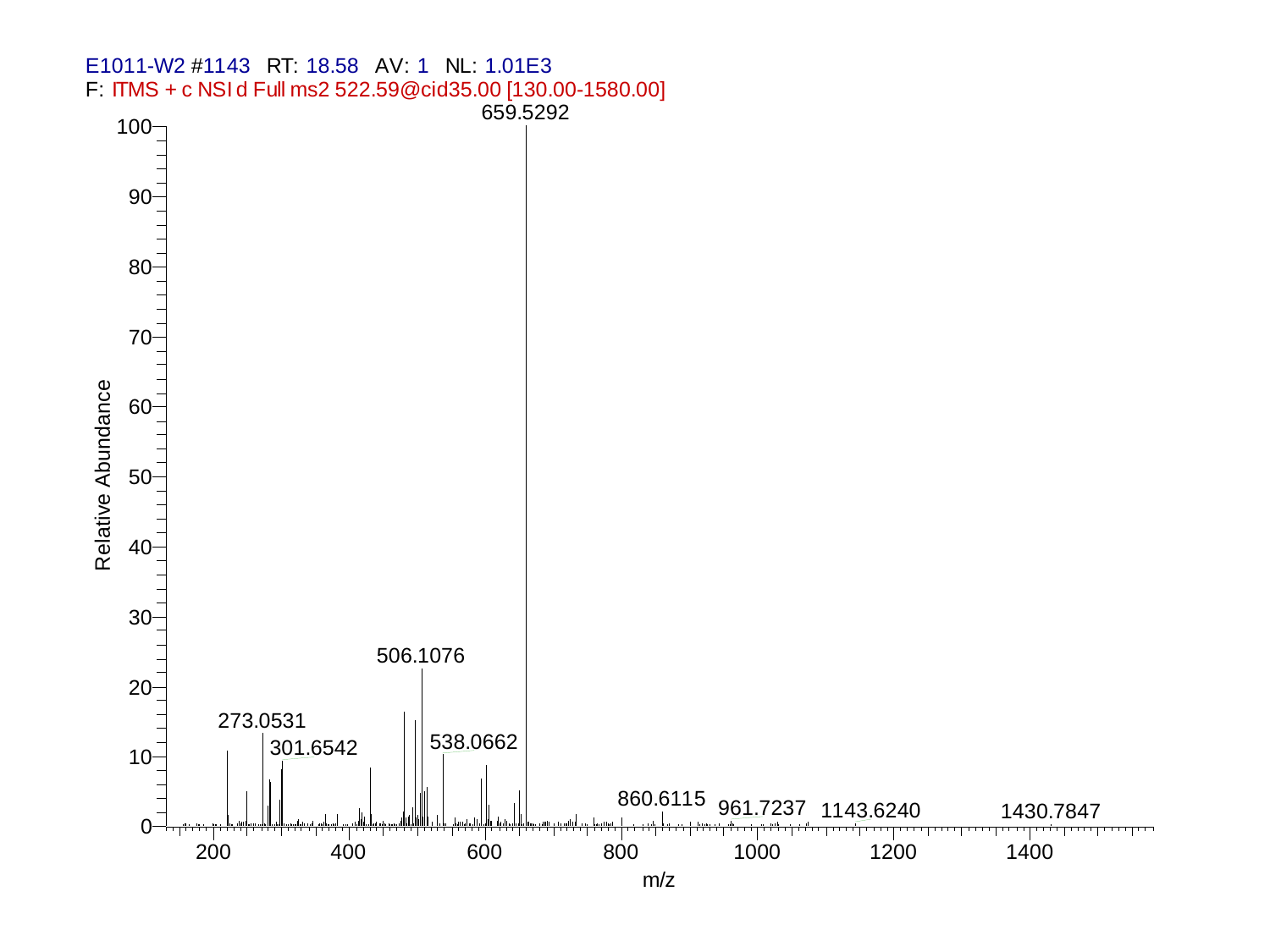

## Slide 24
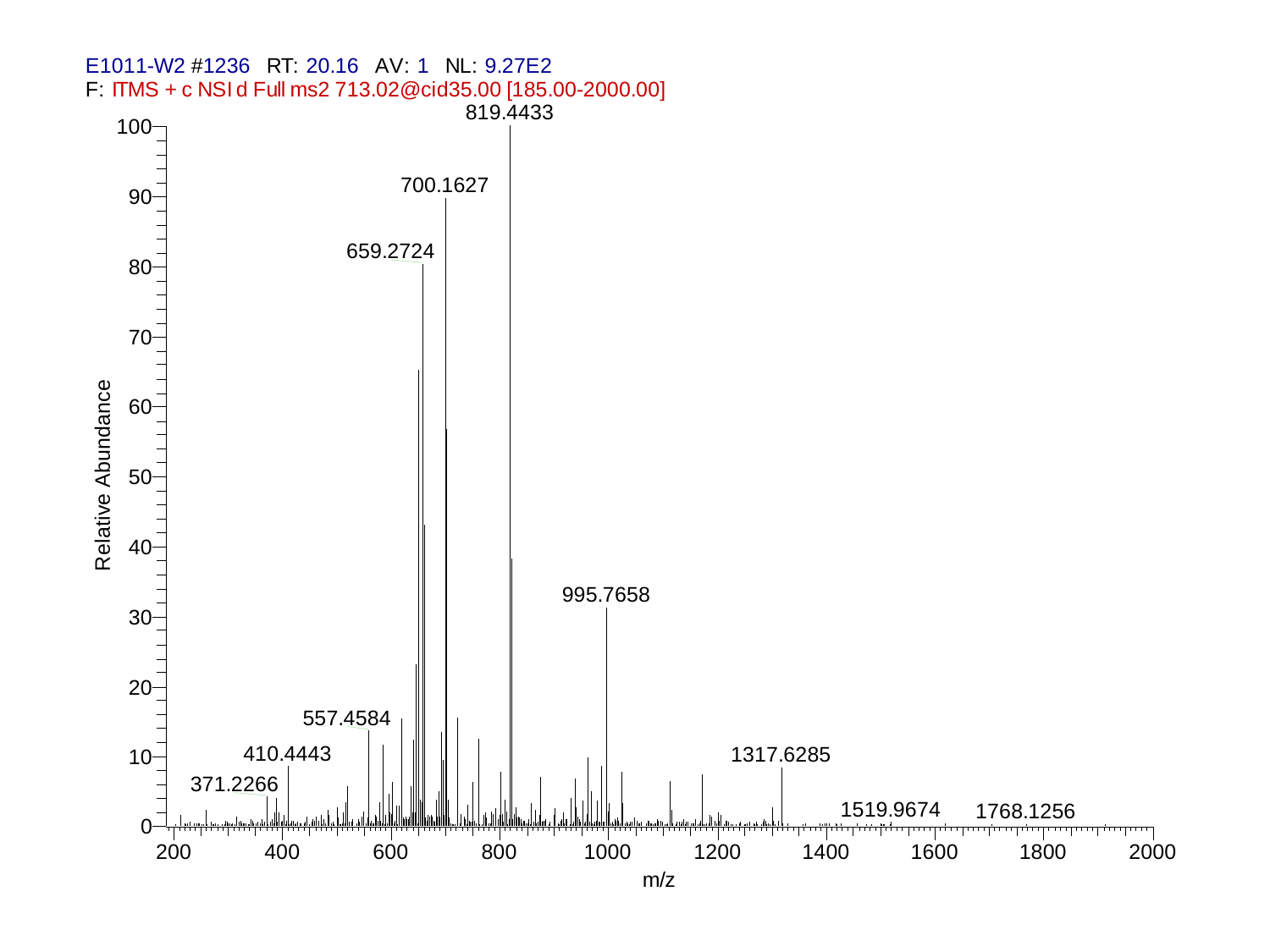

## Slide 25
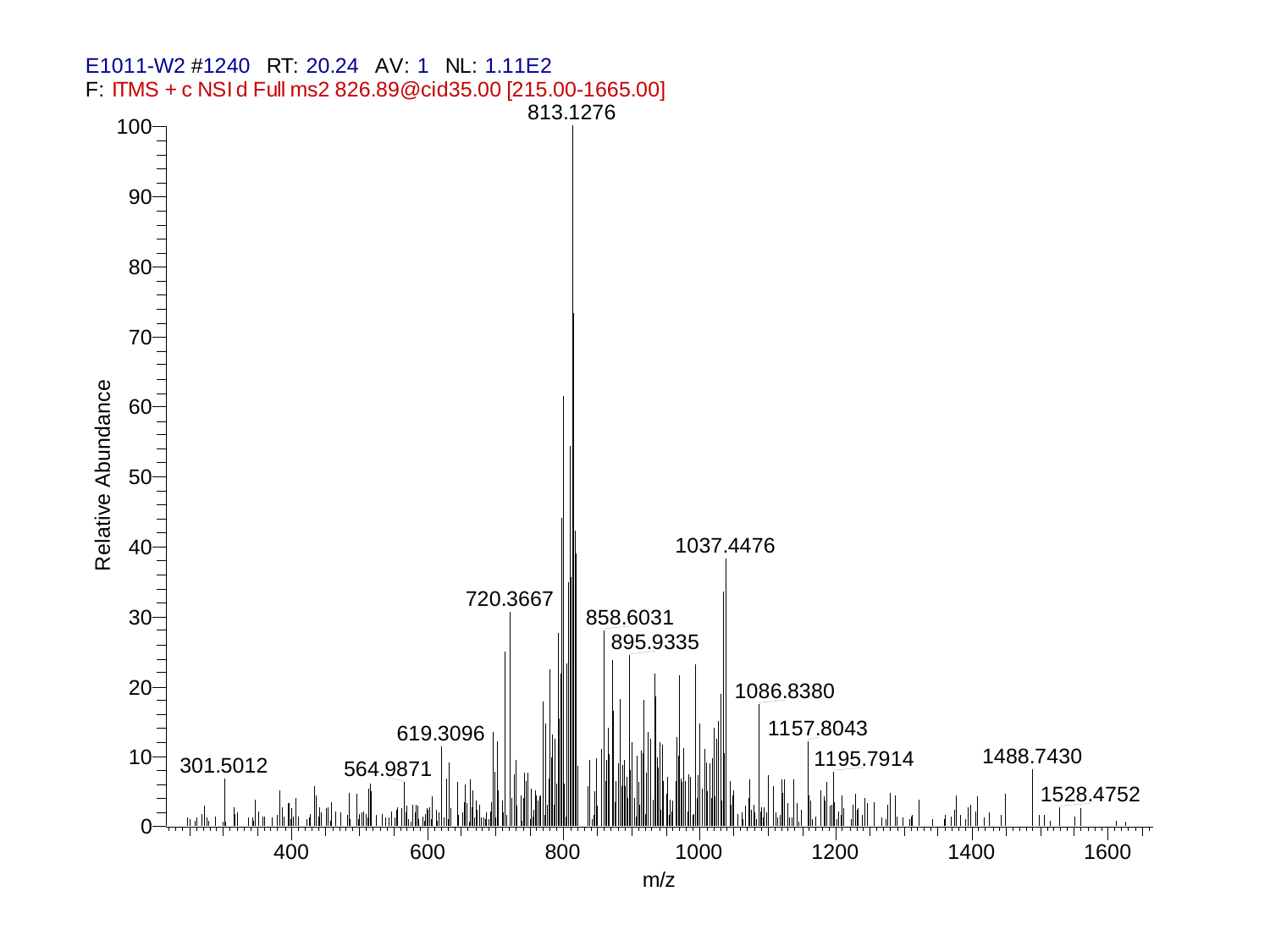

## Slide 26
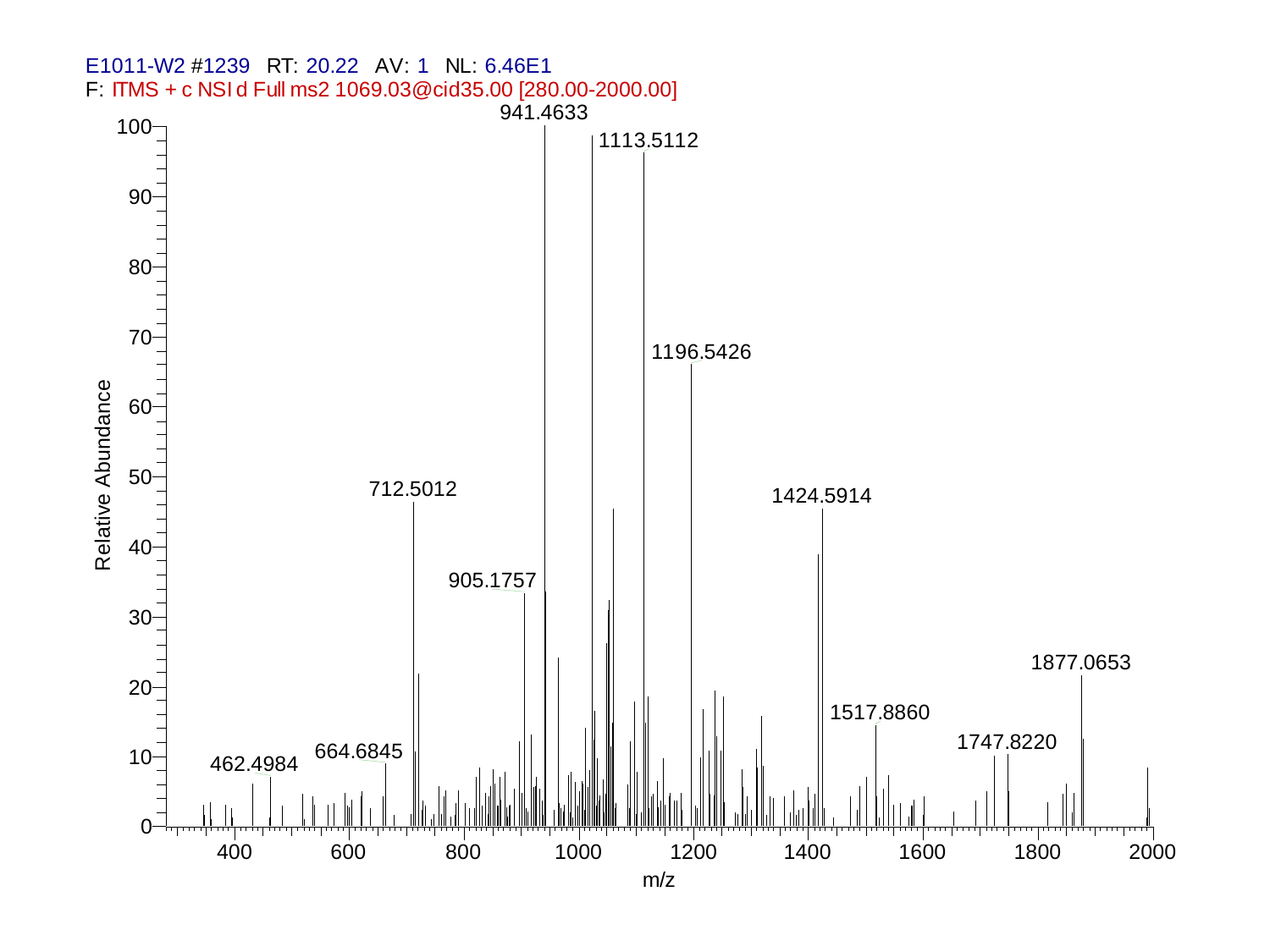

## Slide 27
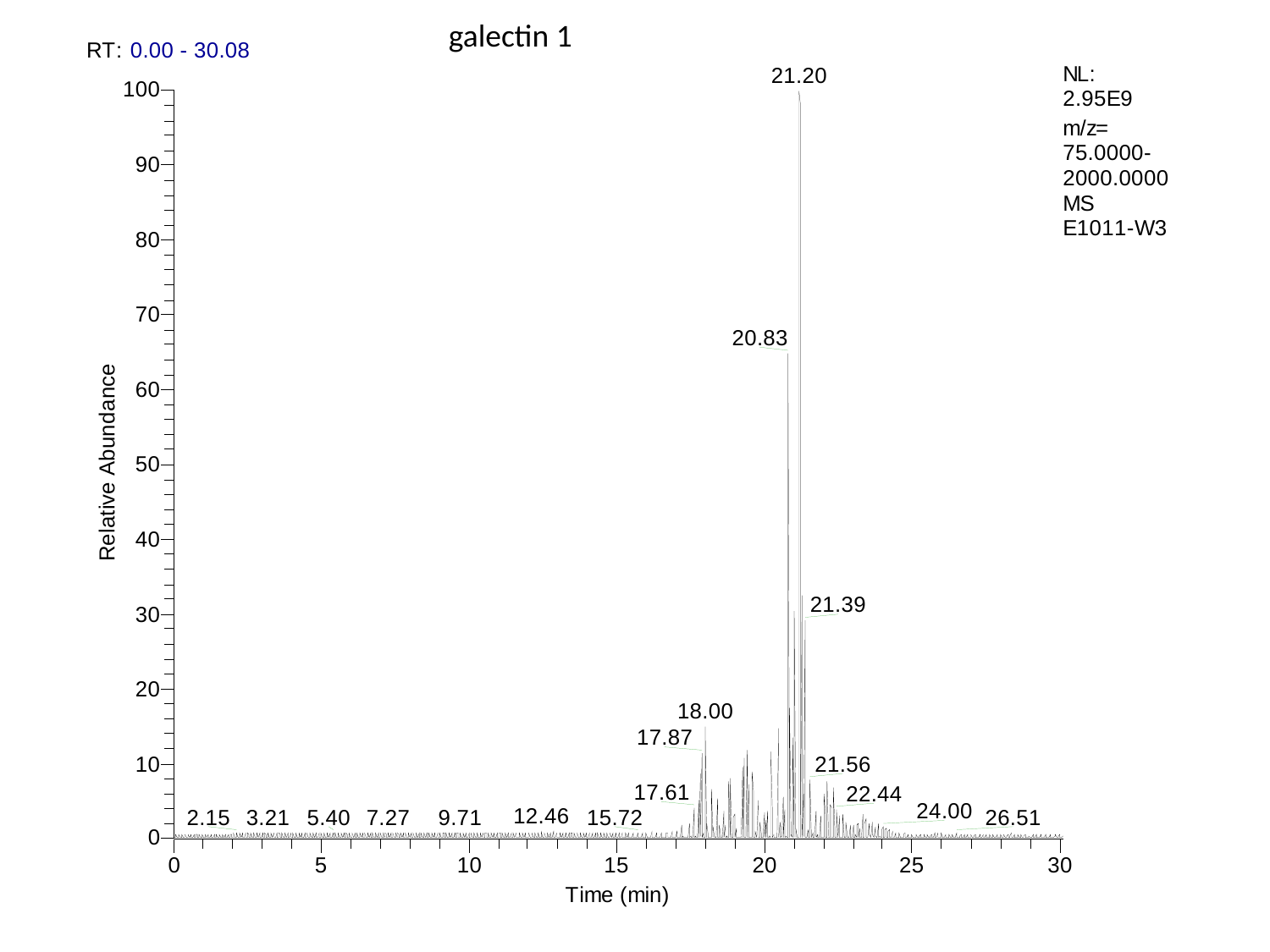

galectin 1

## Slide 28
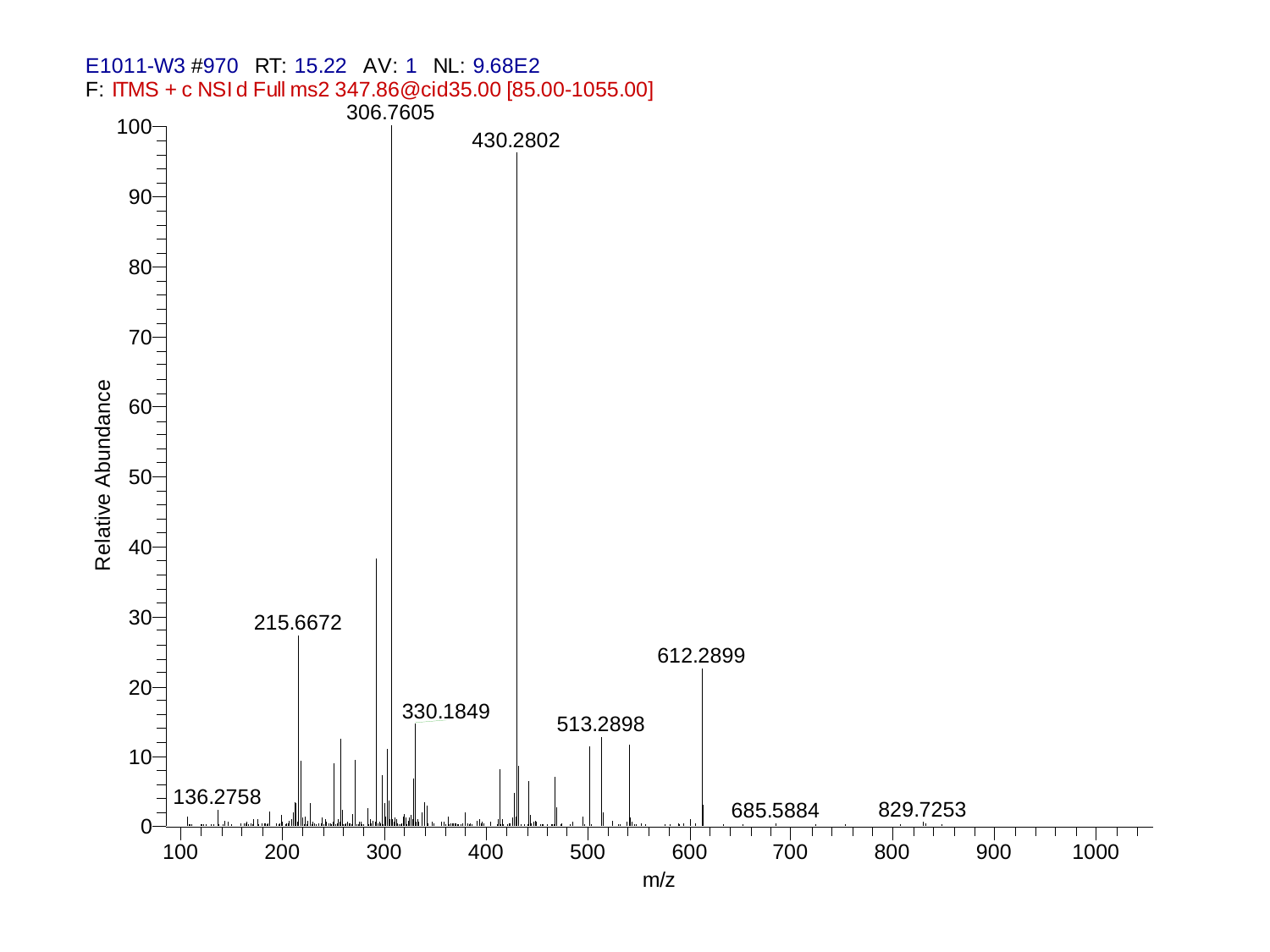

## Slide 29
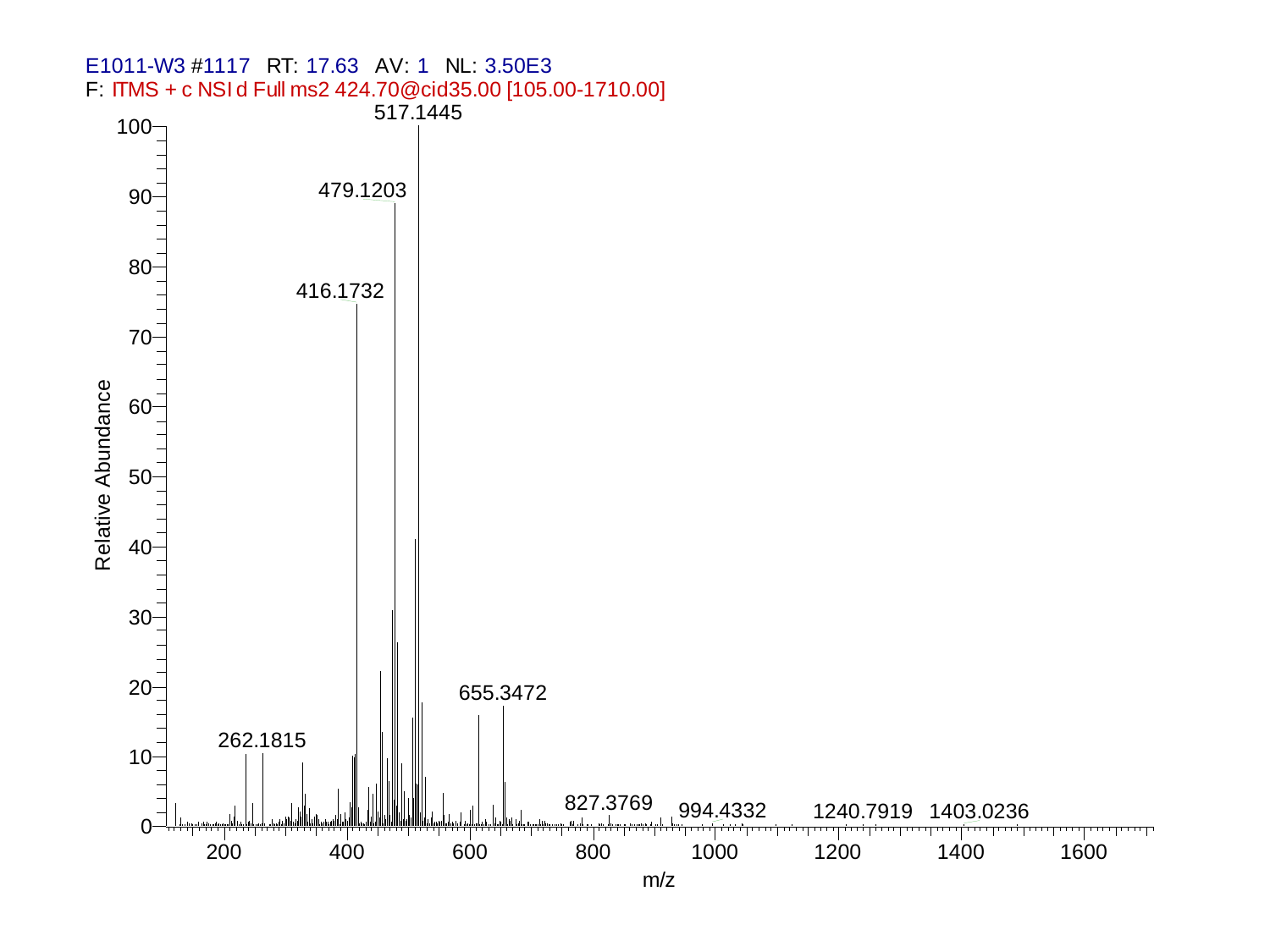

## Slide 30
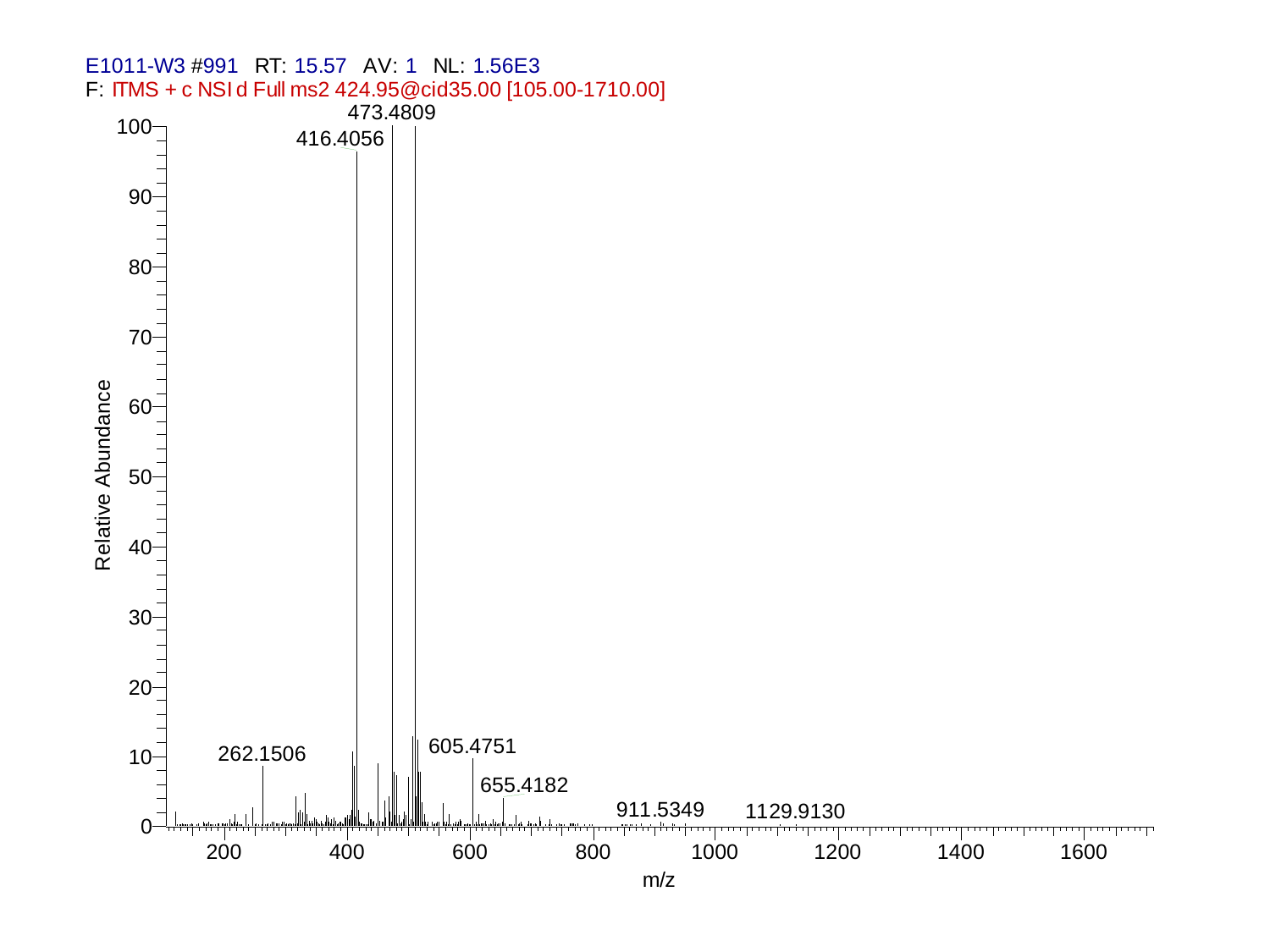

## Slide 31
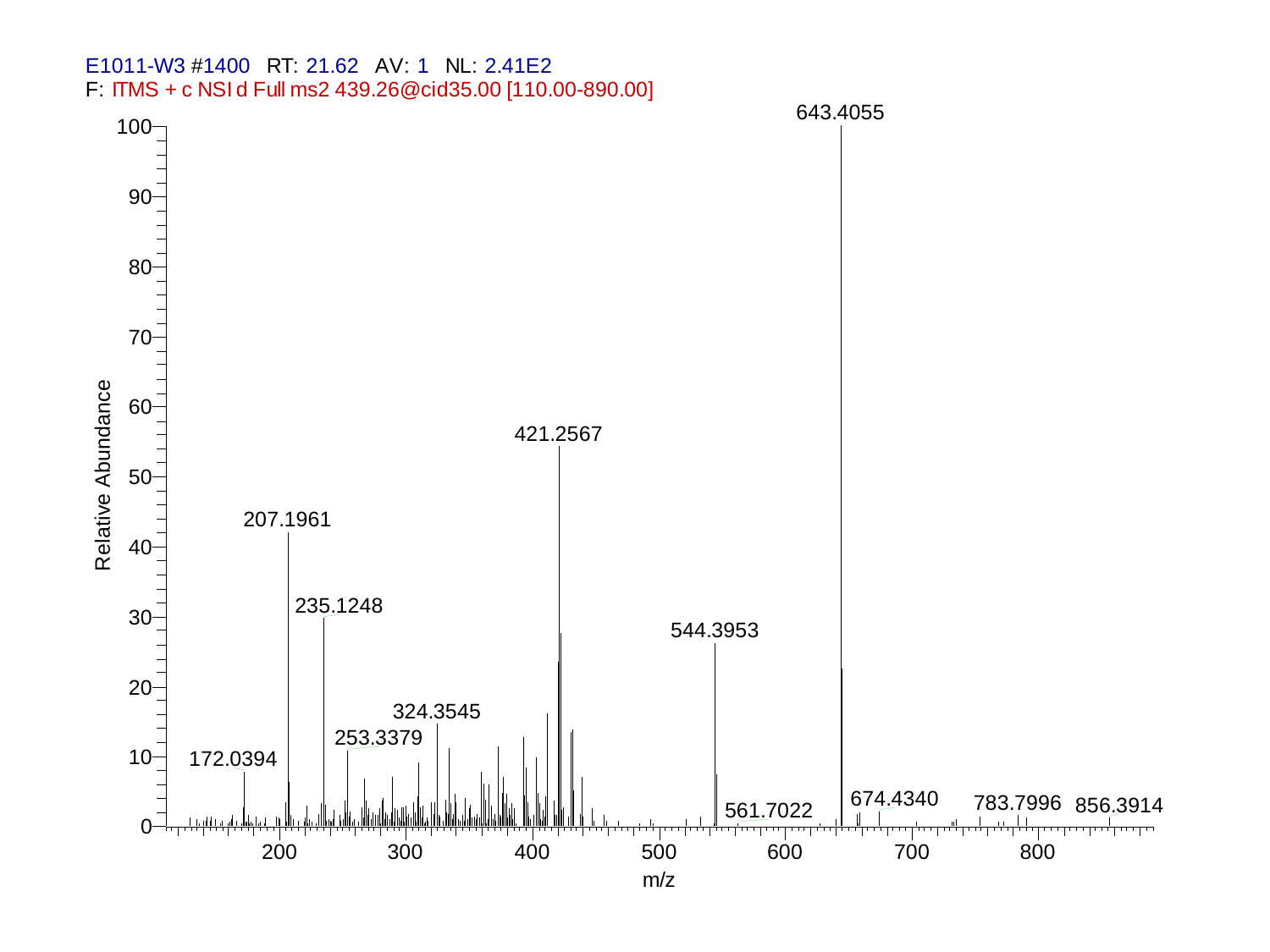

## Slide 32
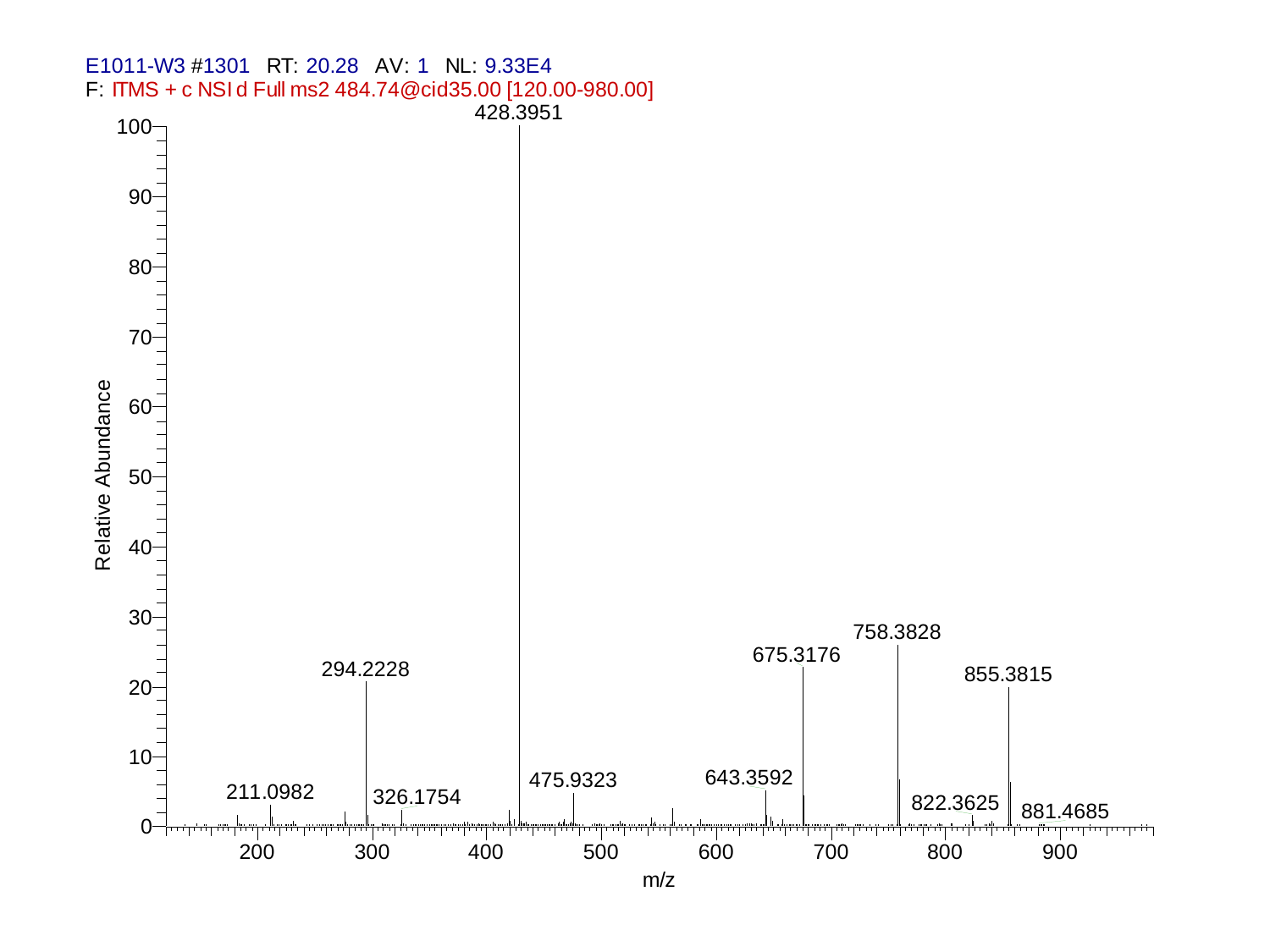

## Slide 33
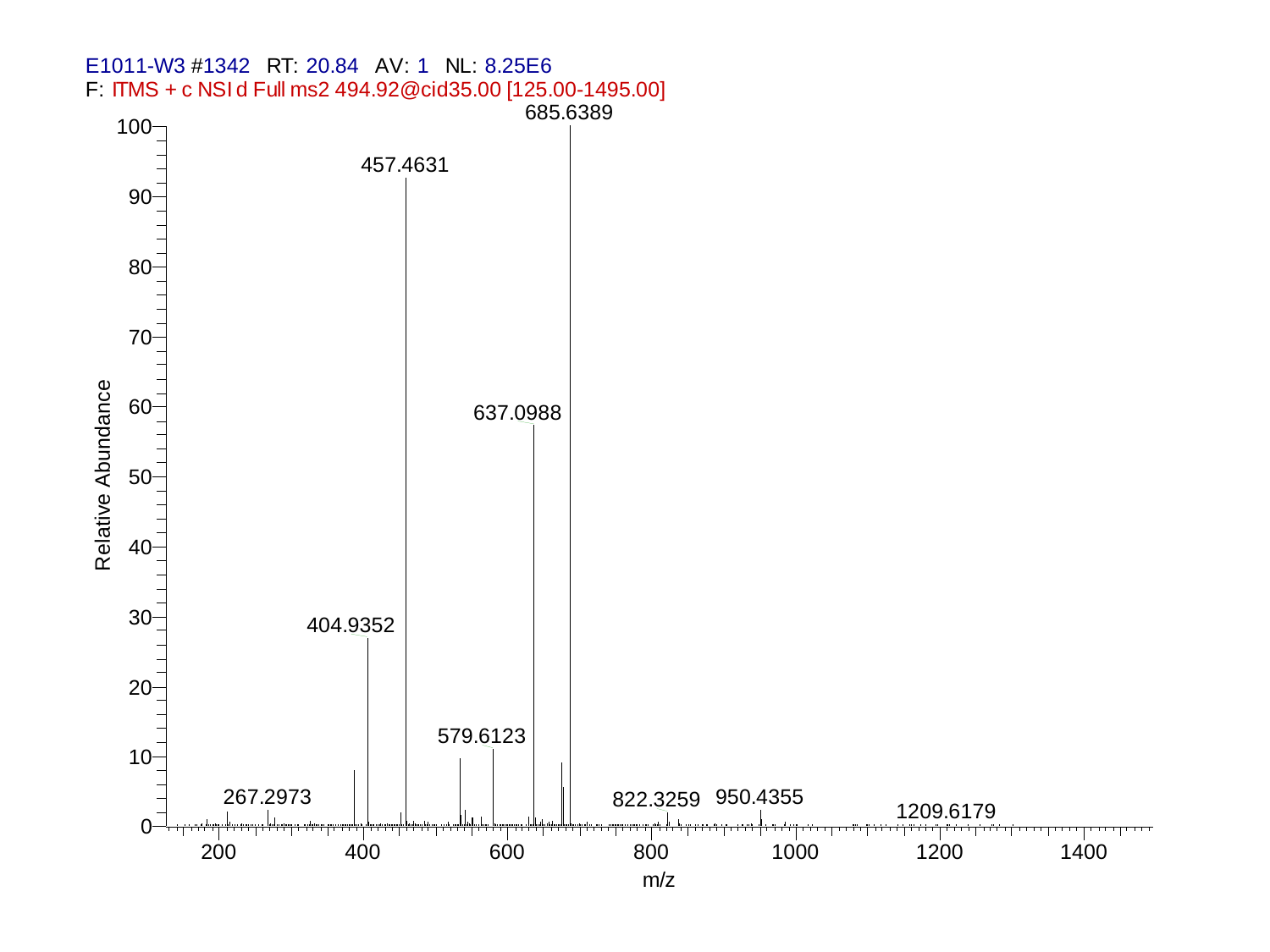

## Slide 34
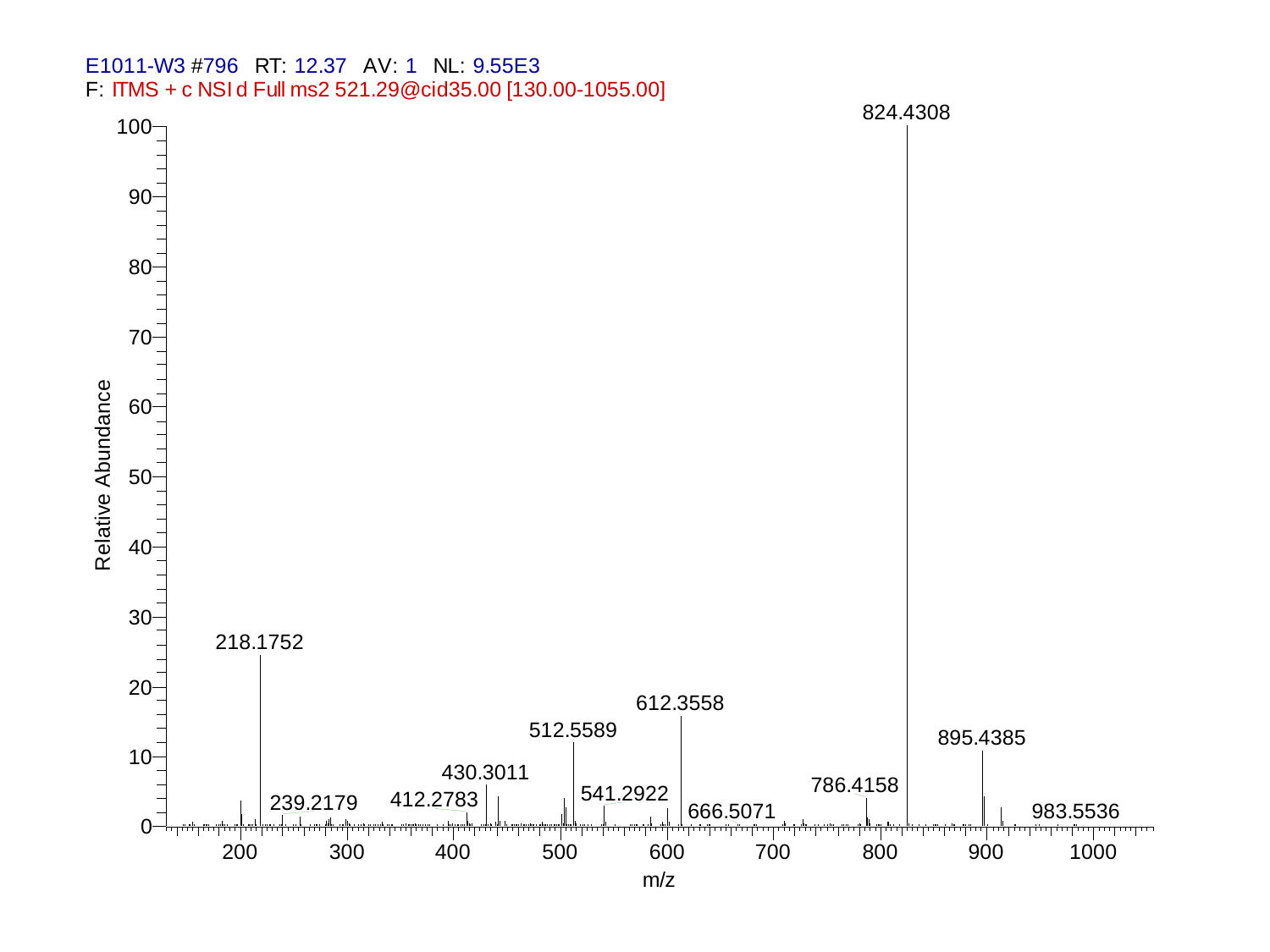

## Slide 35
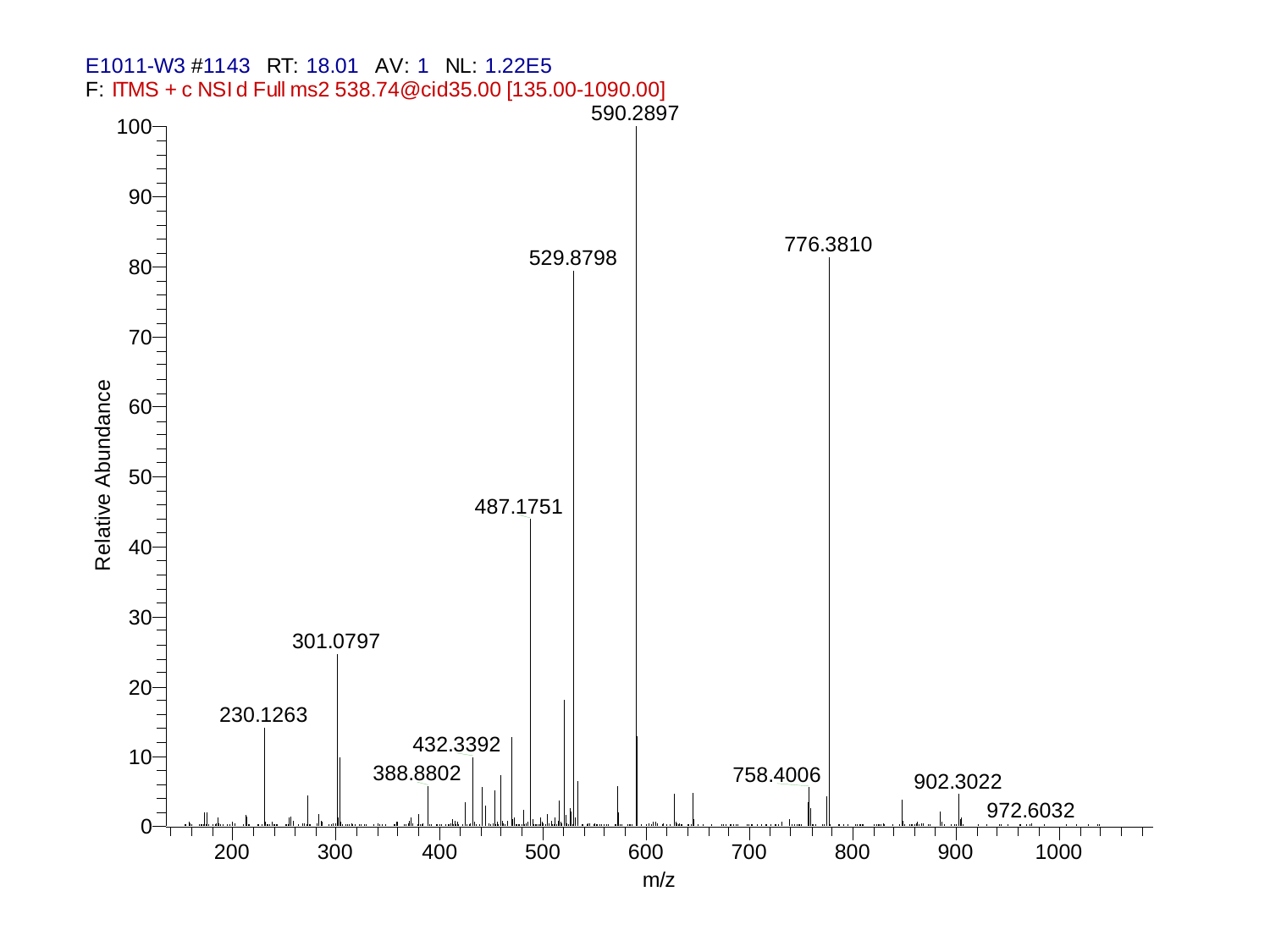

## Slide 36
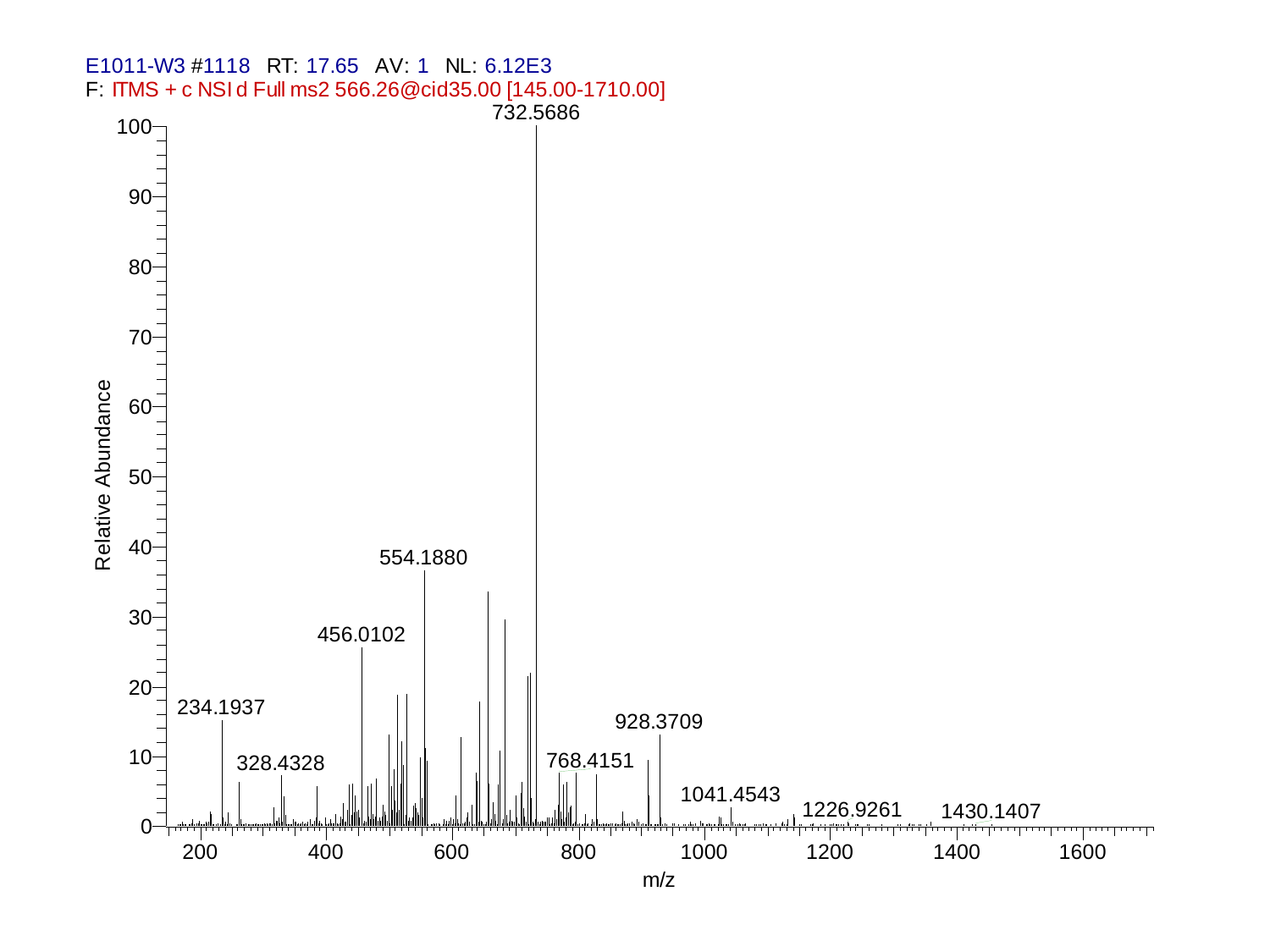

## Slide 37
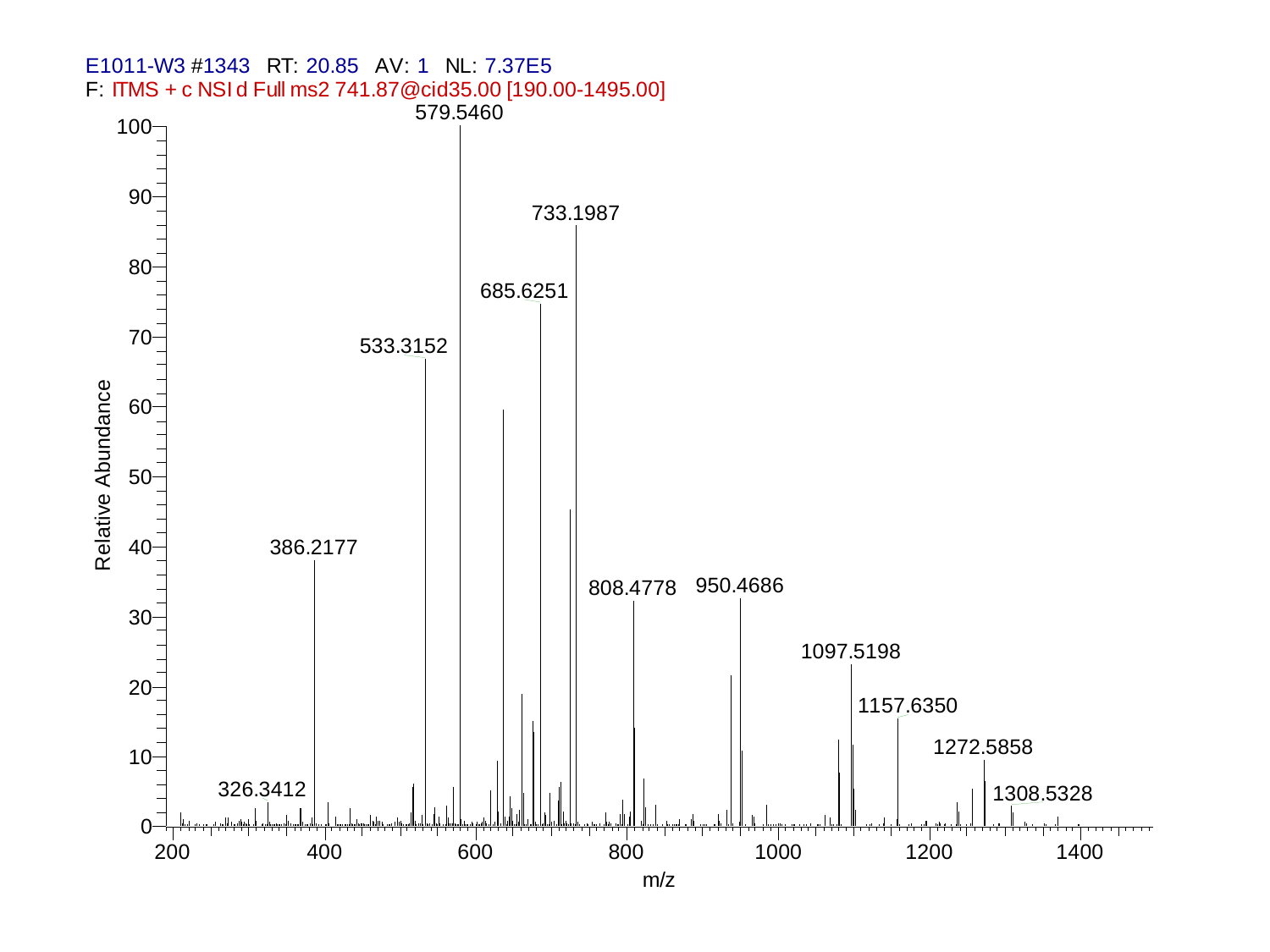

## Slide 38
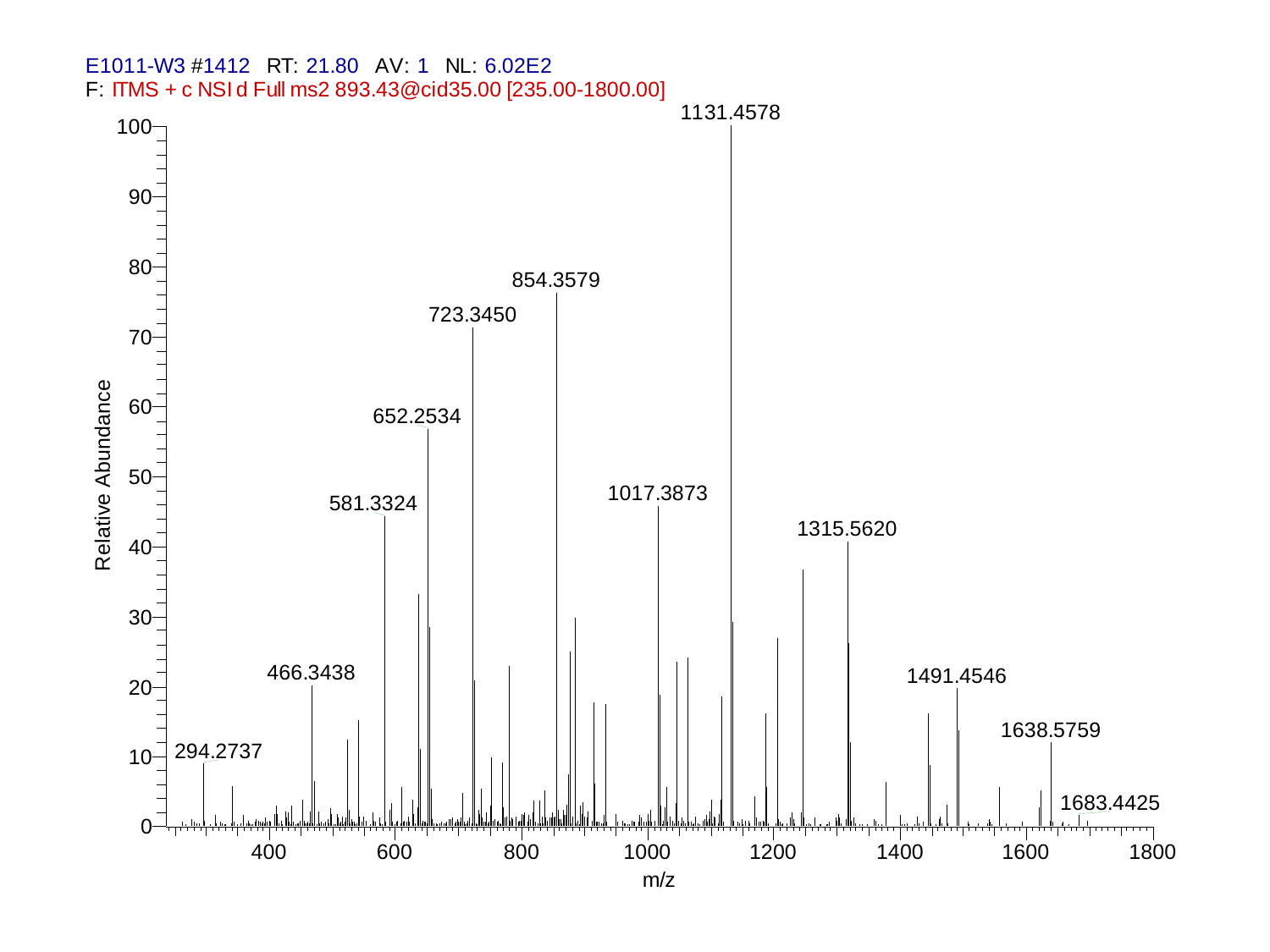

## Slide 39
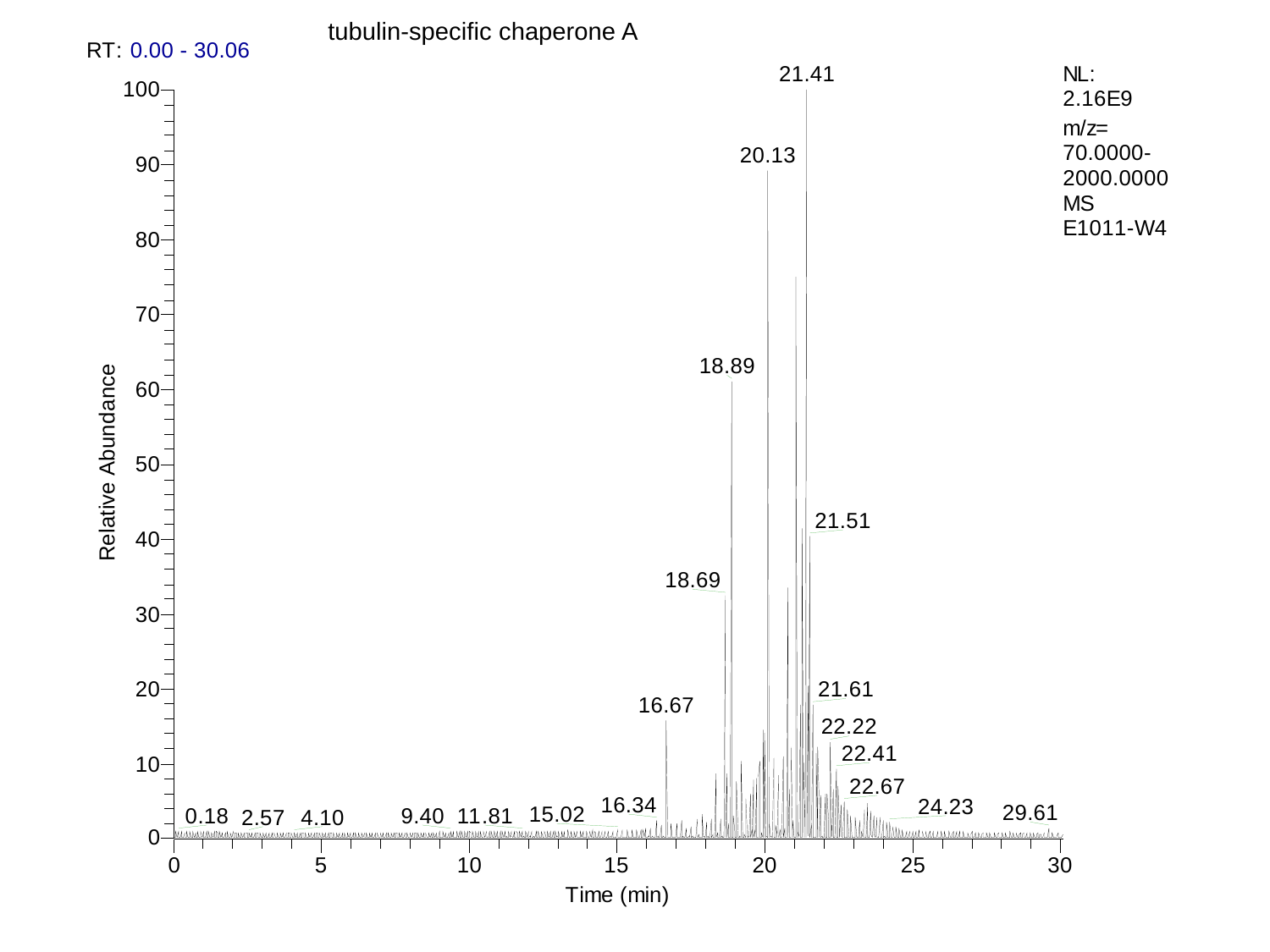

tubulin-specific chaperone A

## Slide 40
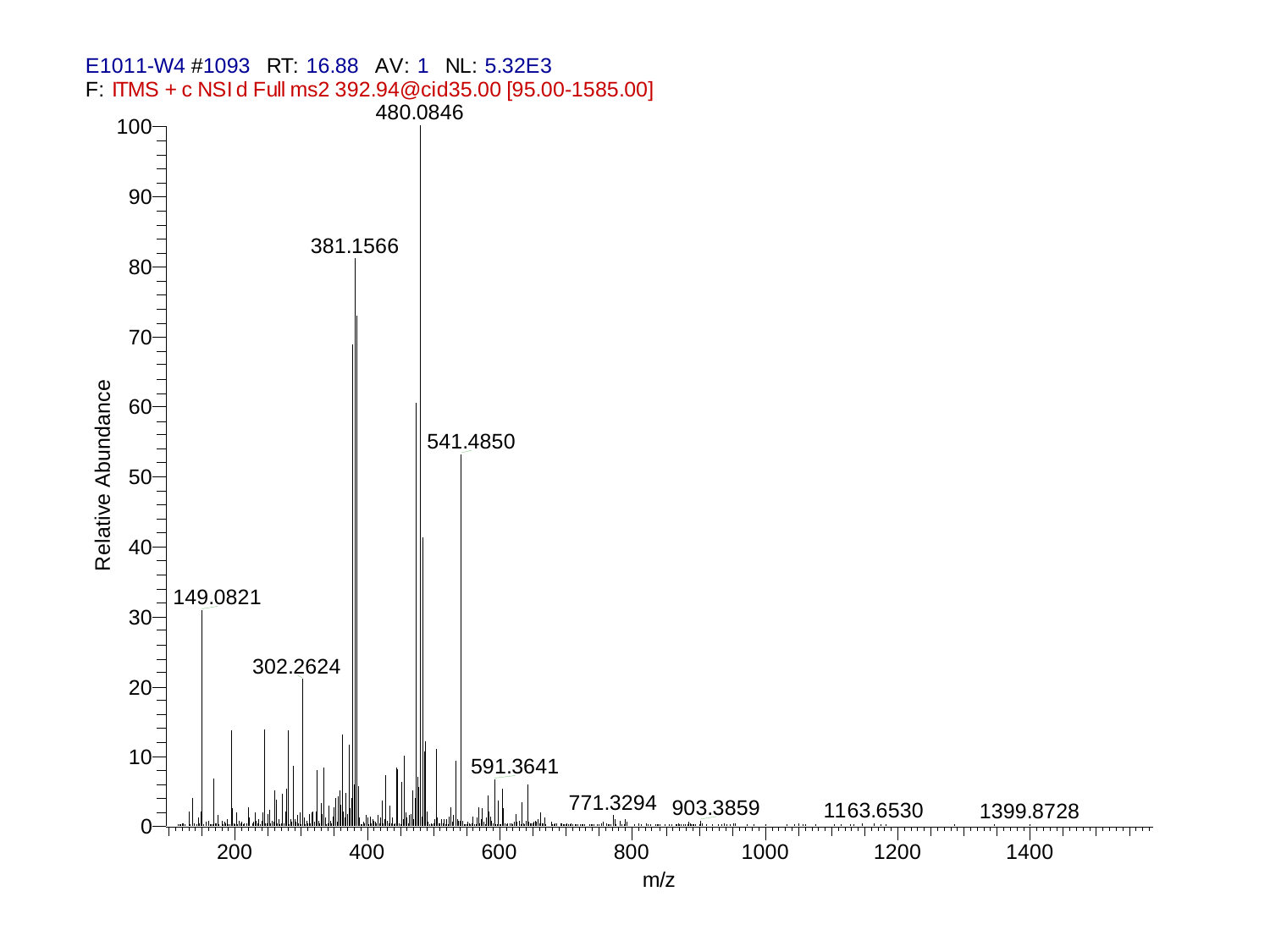

## Slide 41
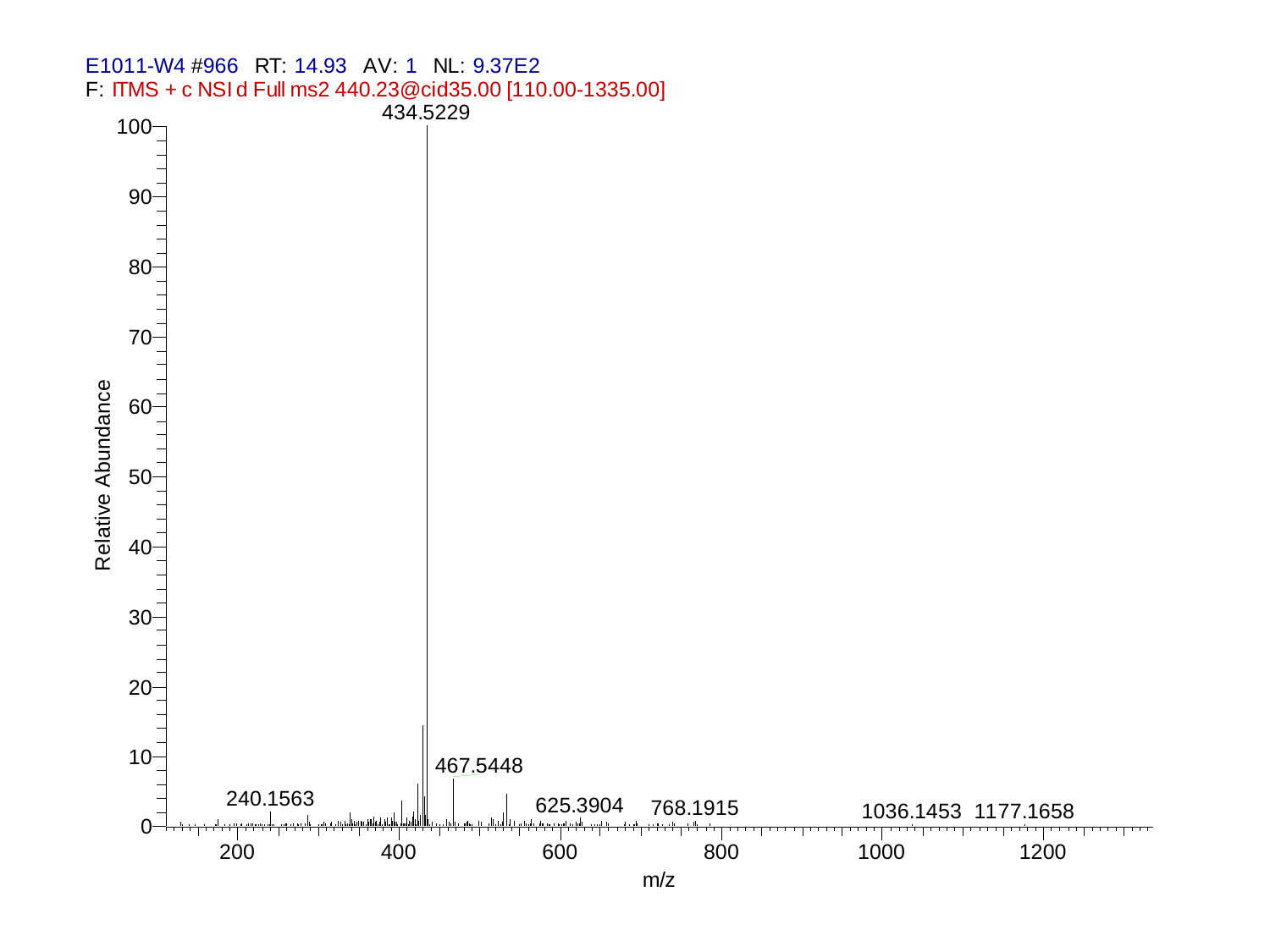

## Slide 42
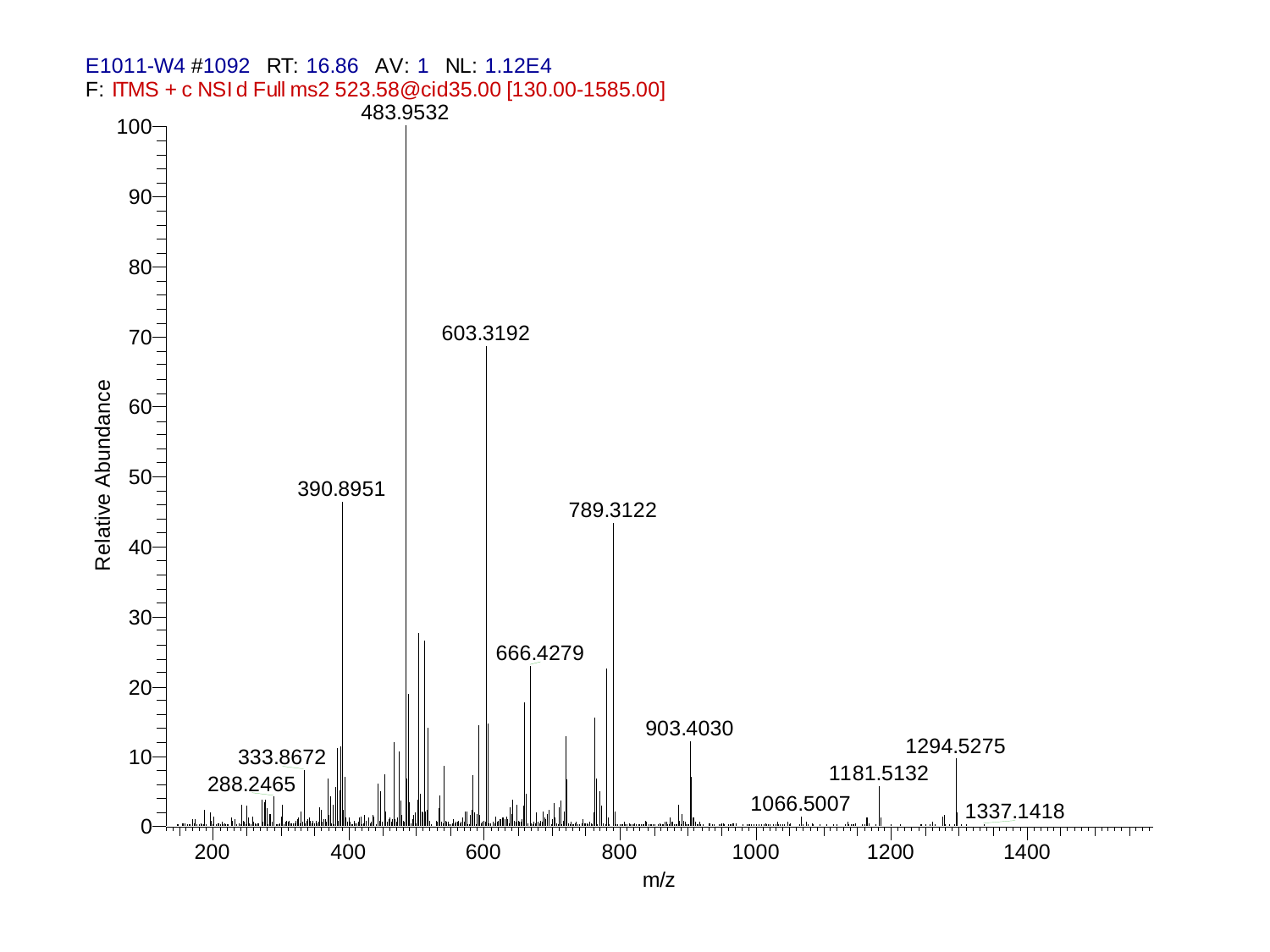

## Slide 43
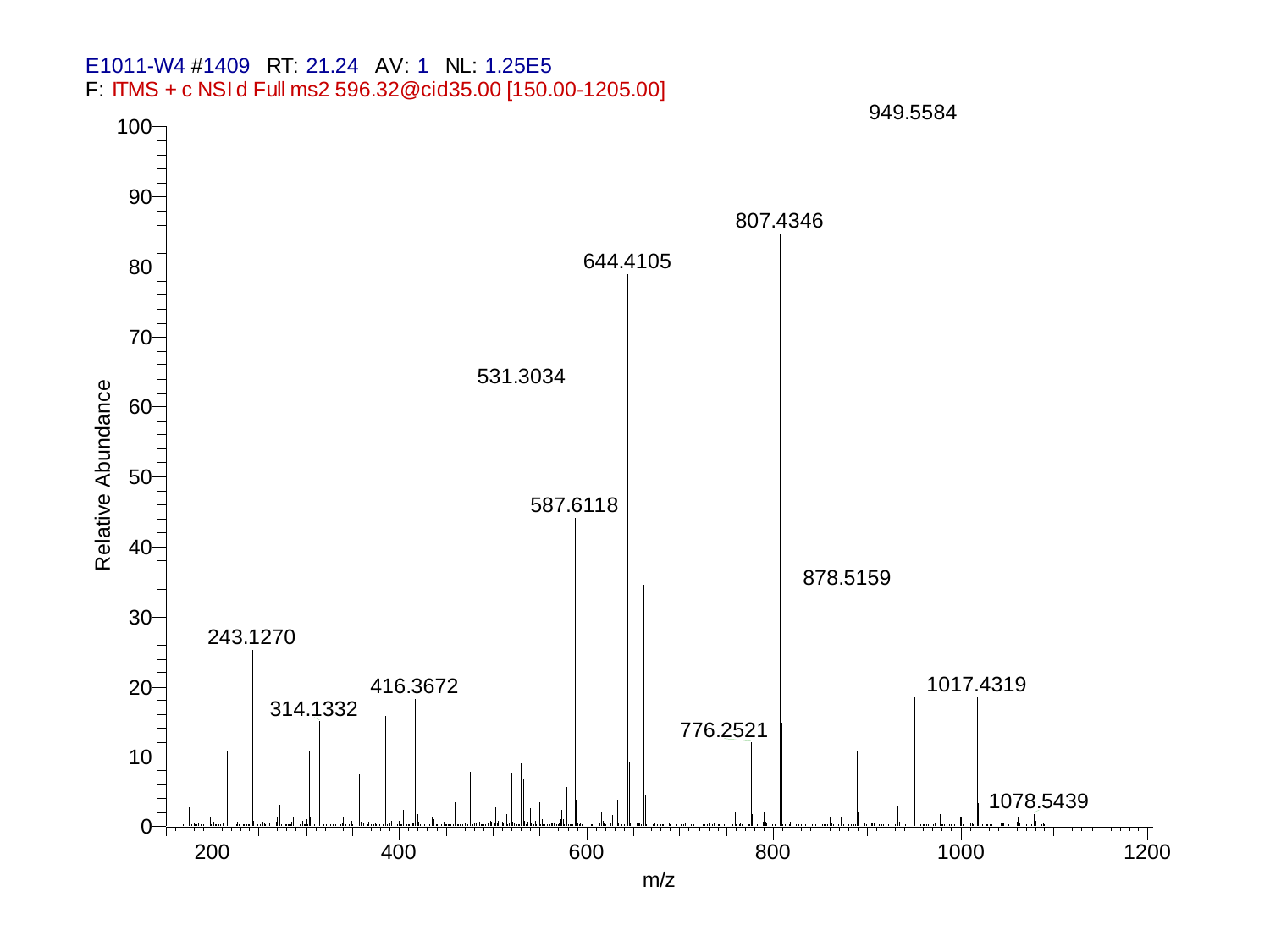

## Slide 44
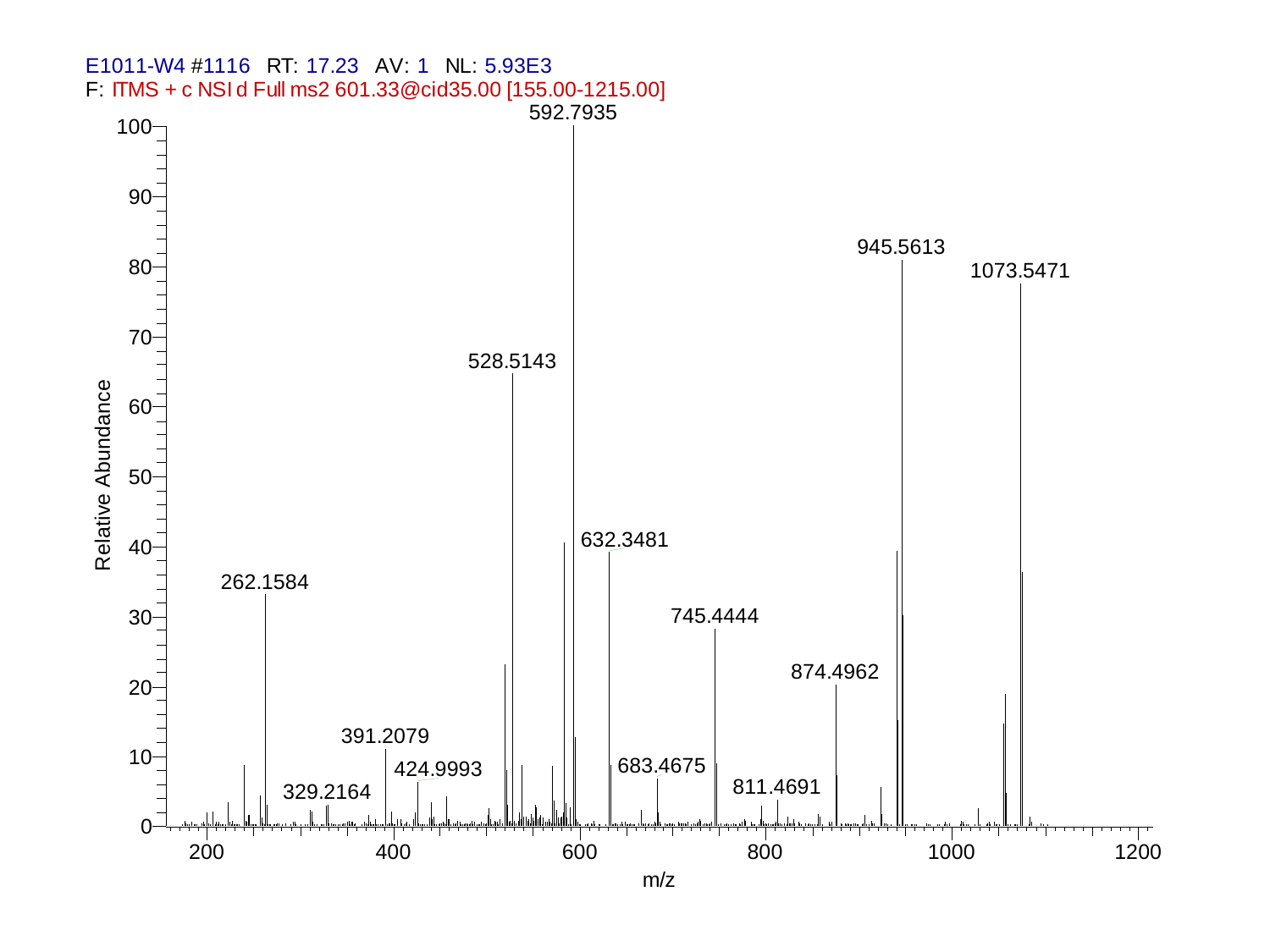

## Slide 45
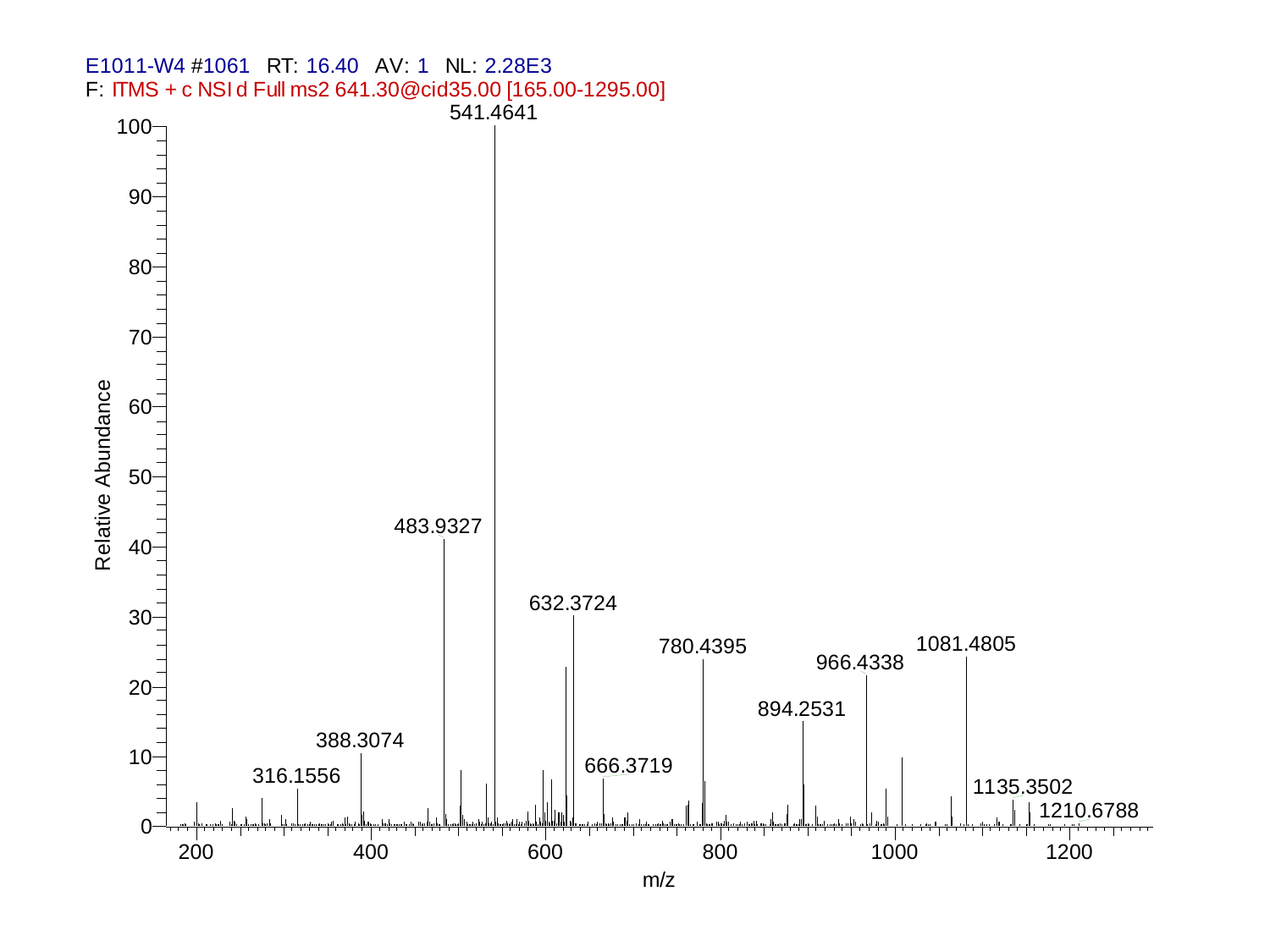

## Slide 46
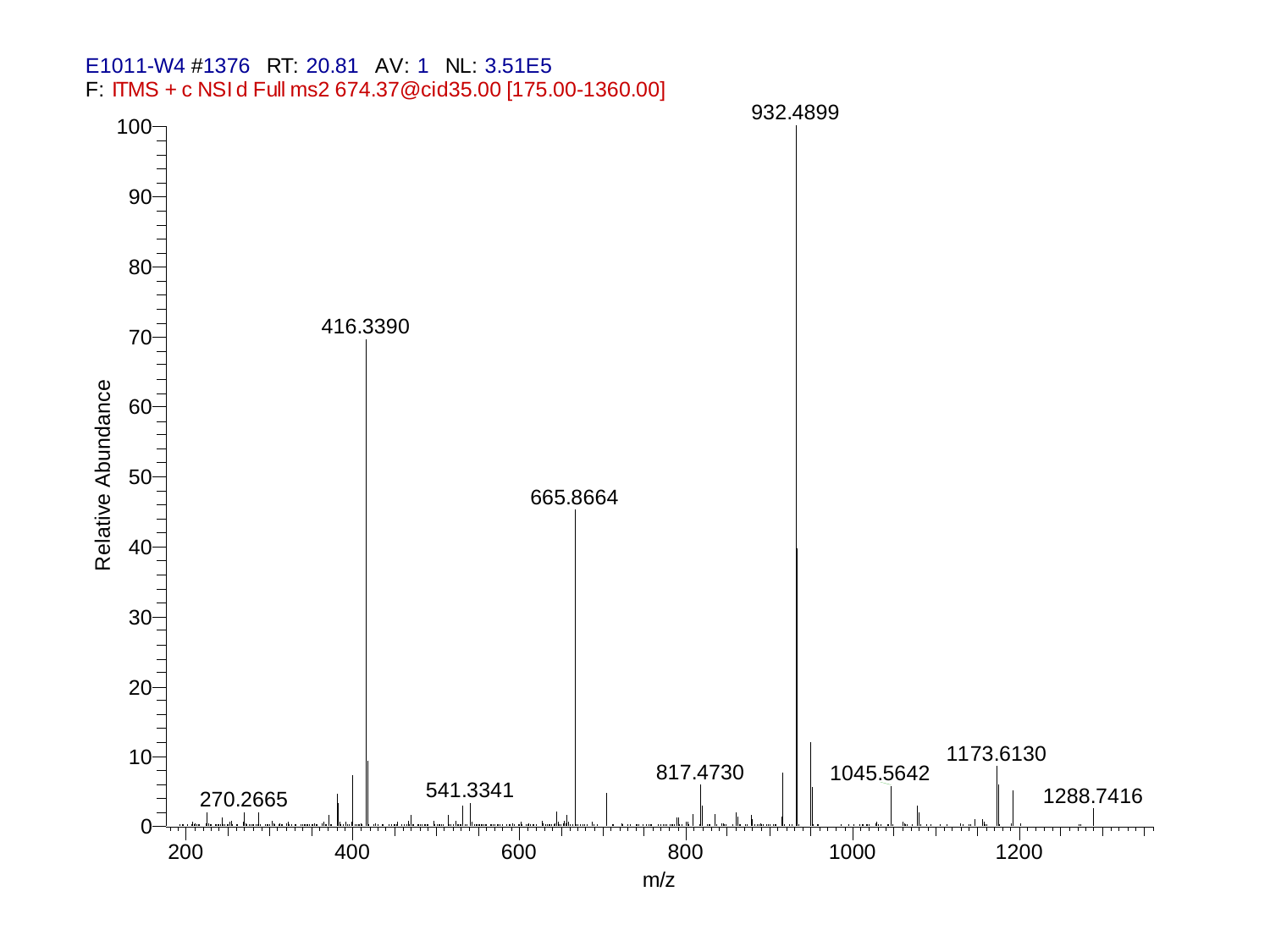

## Slide 47
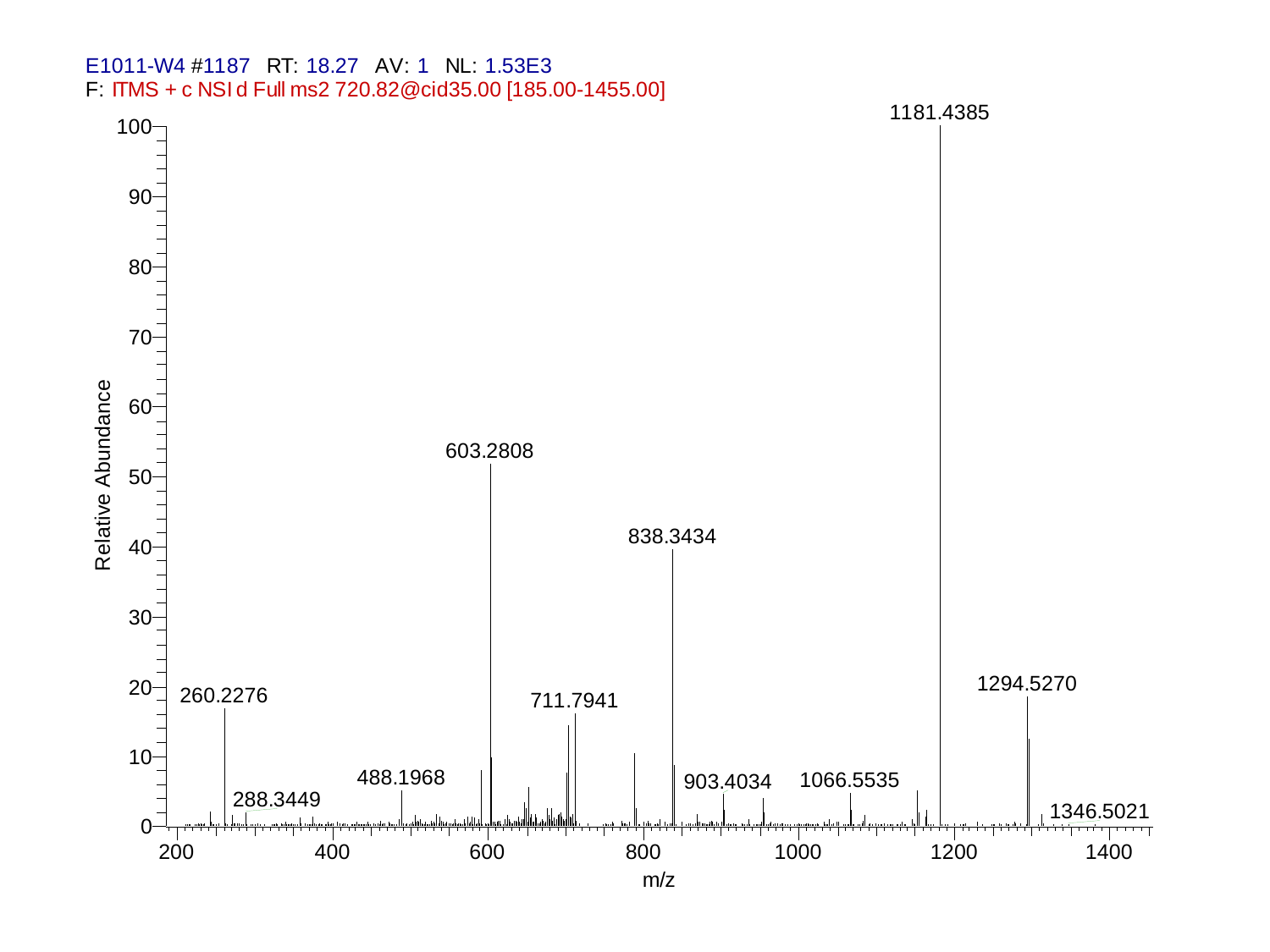

## Slide 48
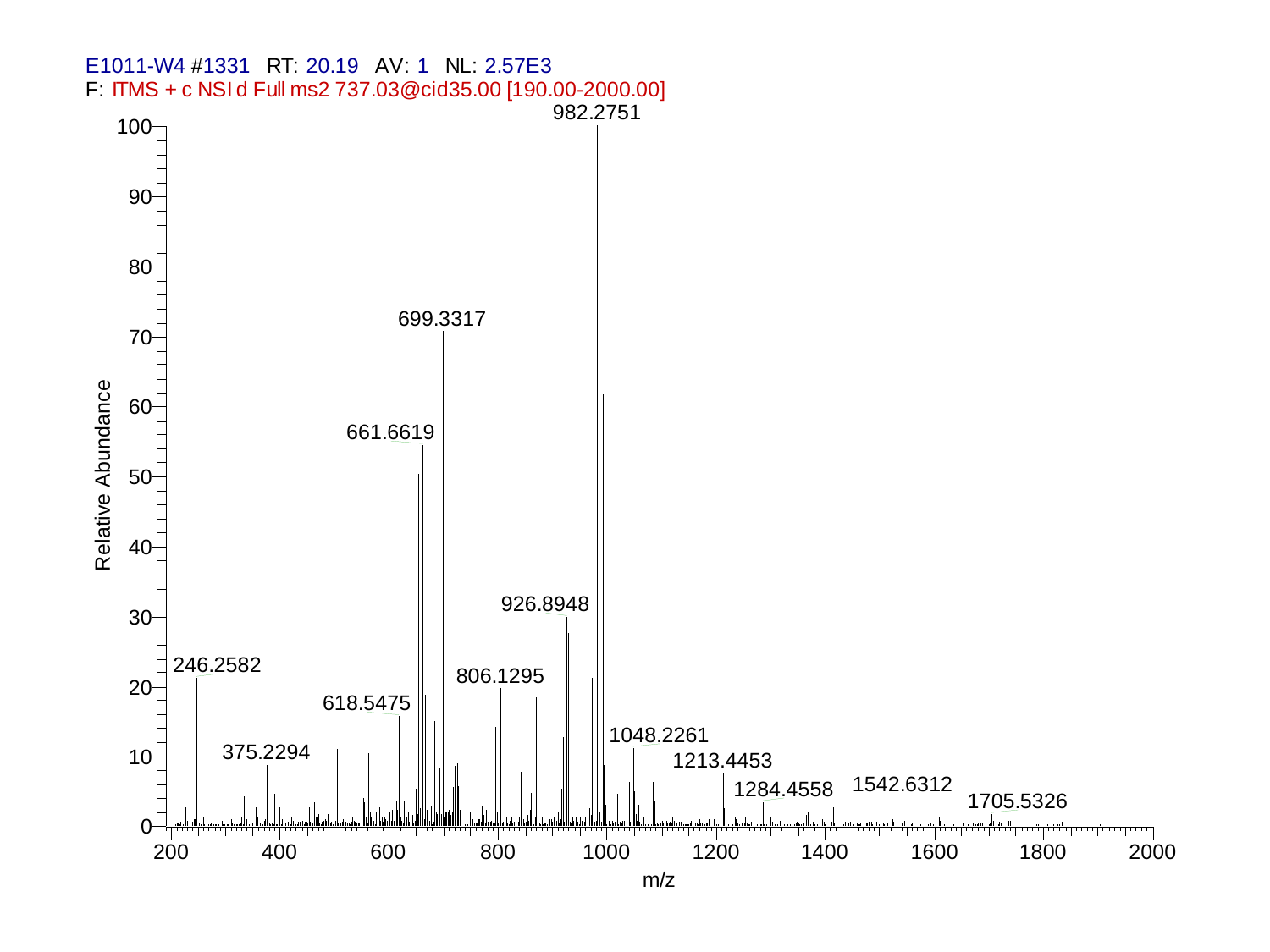

## Slide 49
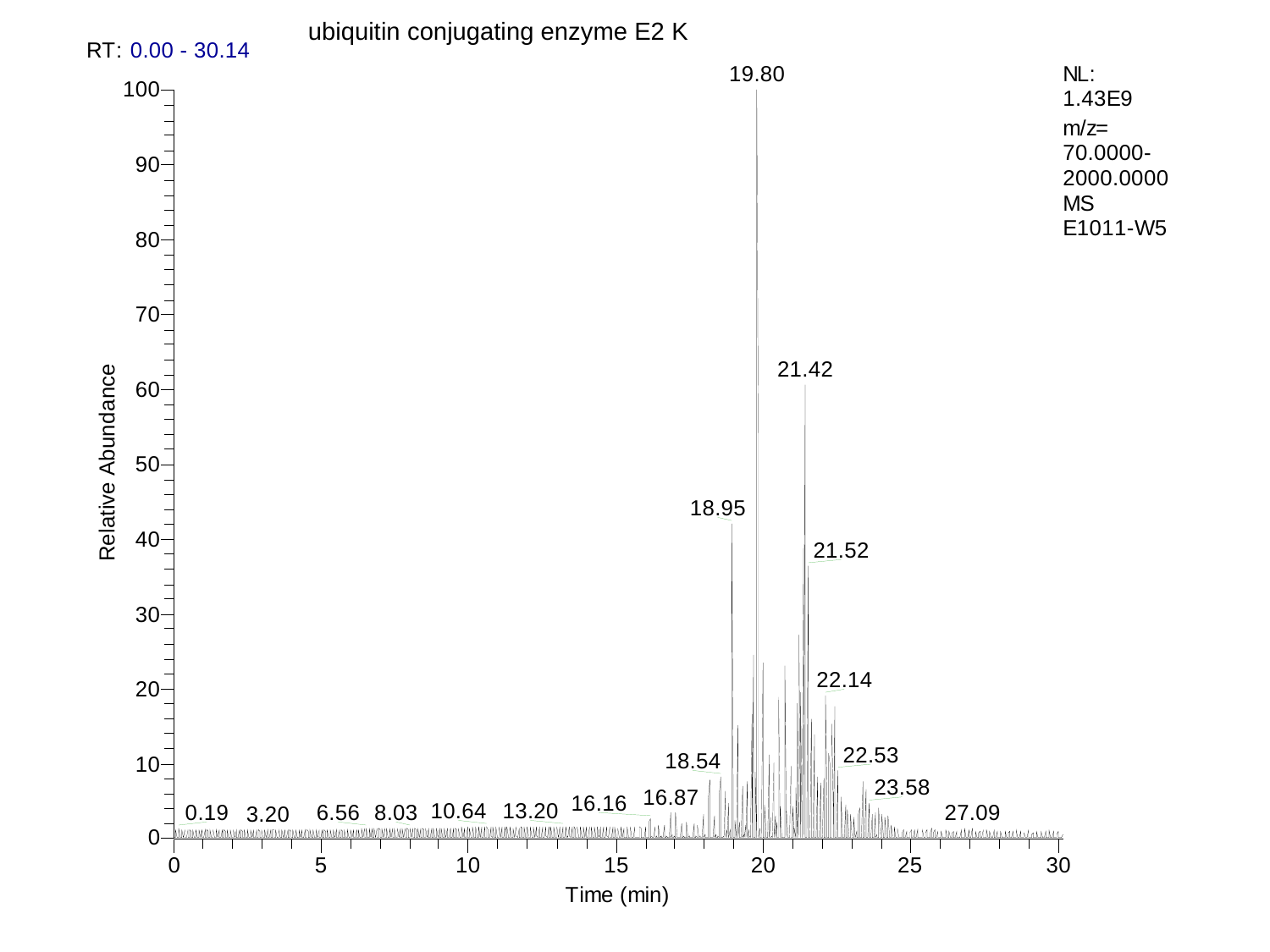

ubiquitin conjugating enzyme E2 K

## Slide 50
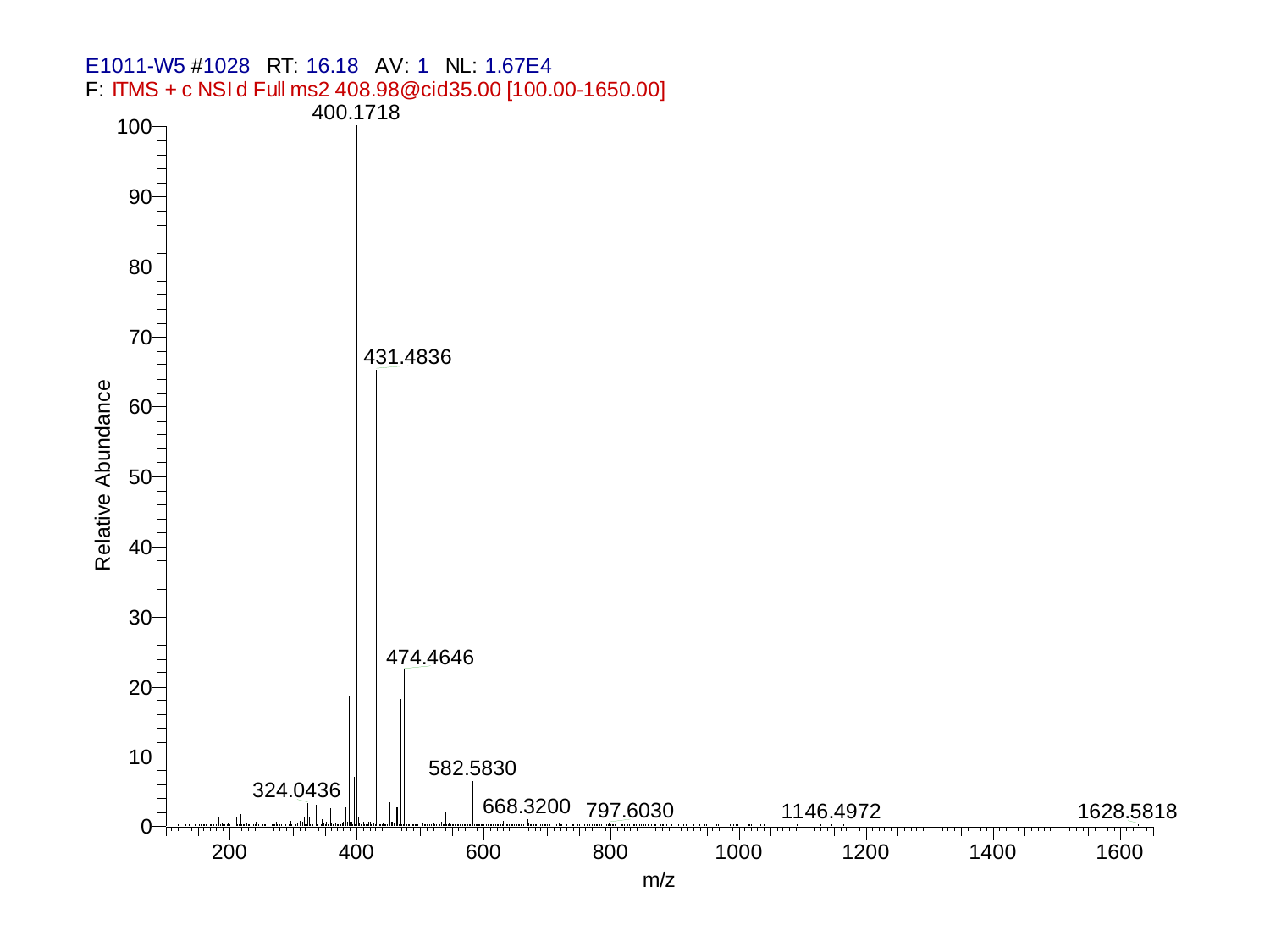

## Slide 51
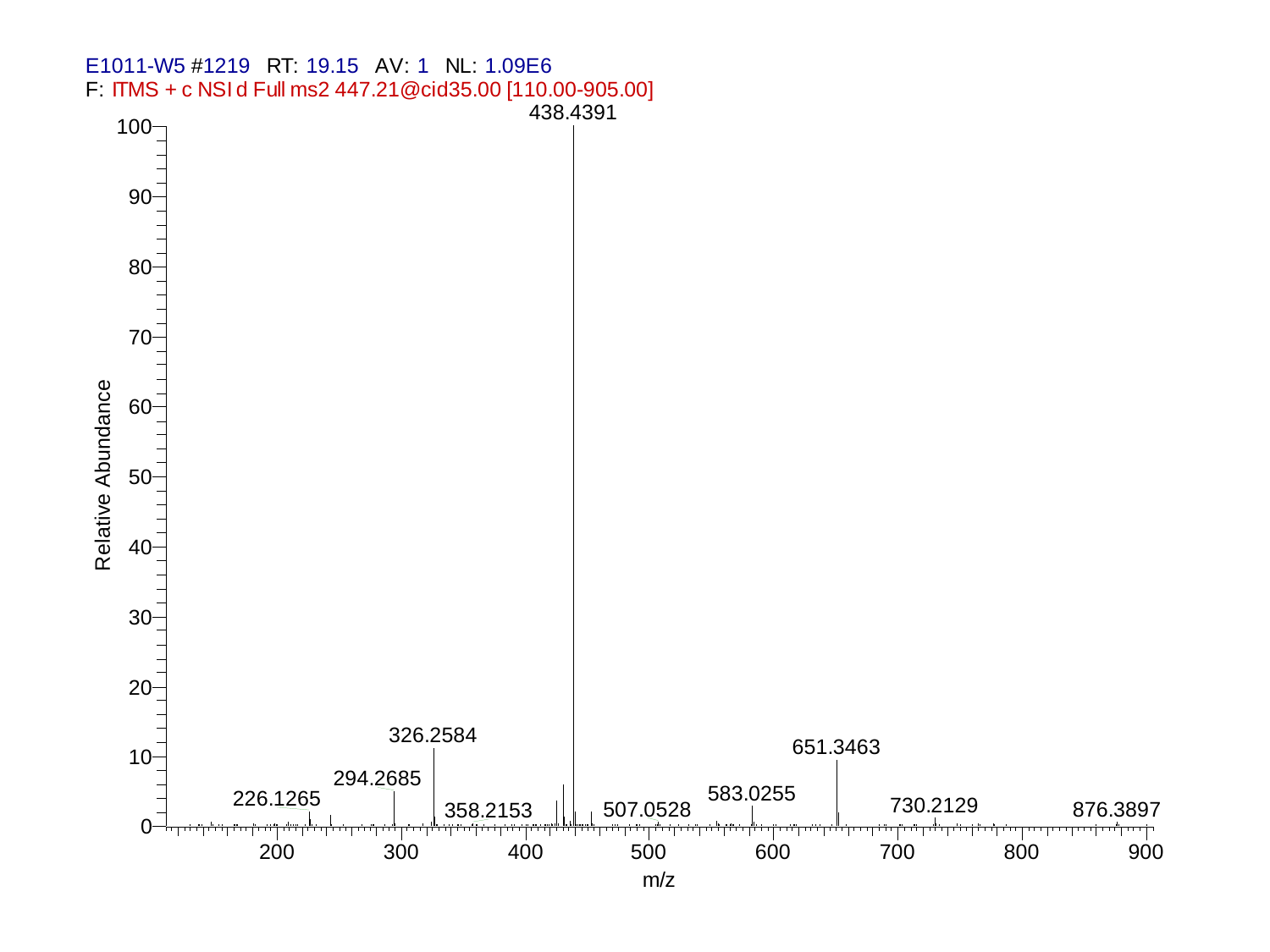

## Slide 52
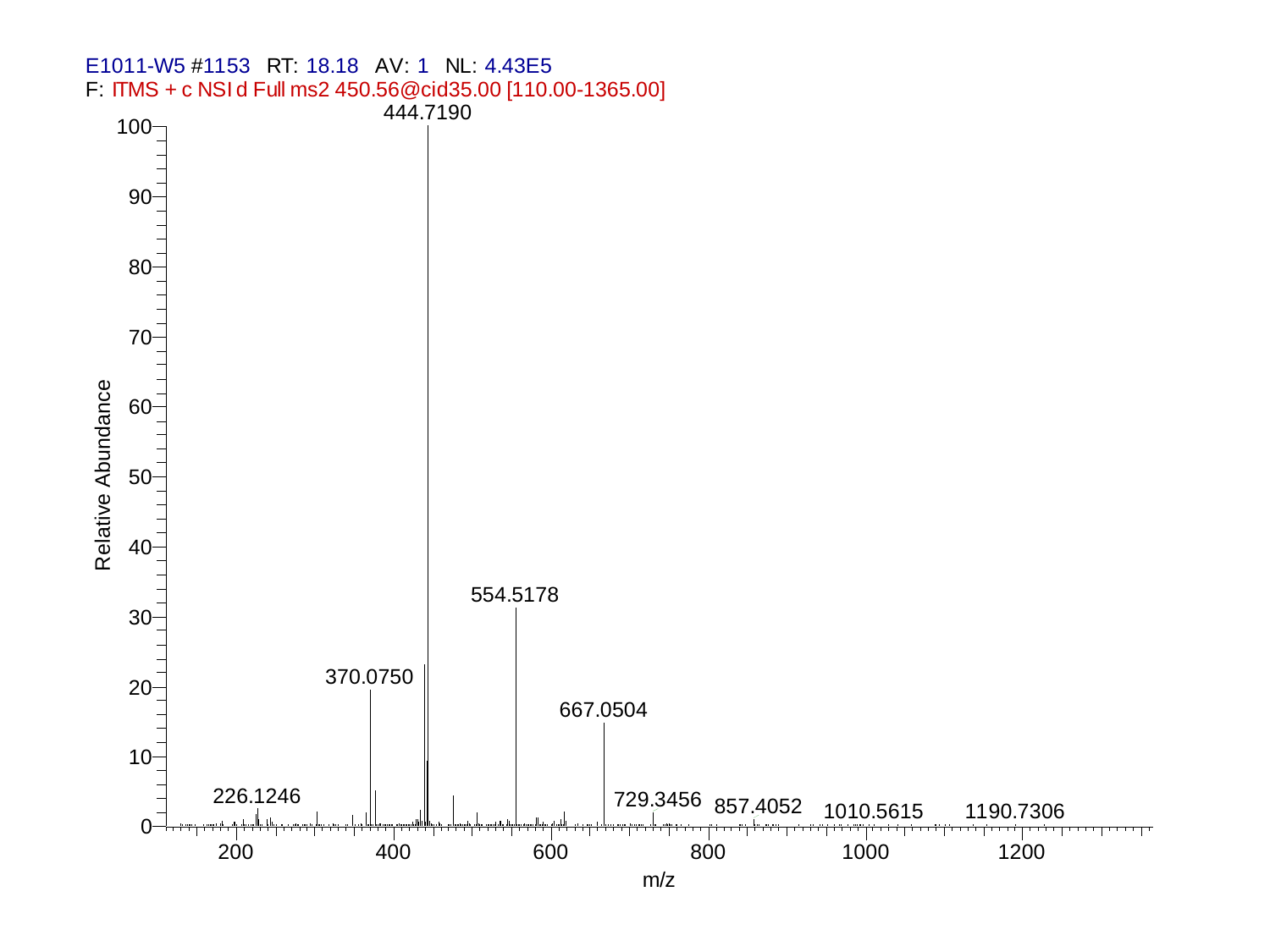

## Slide 53
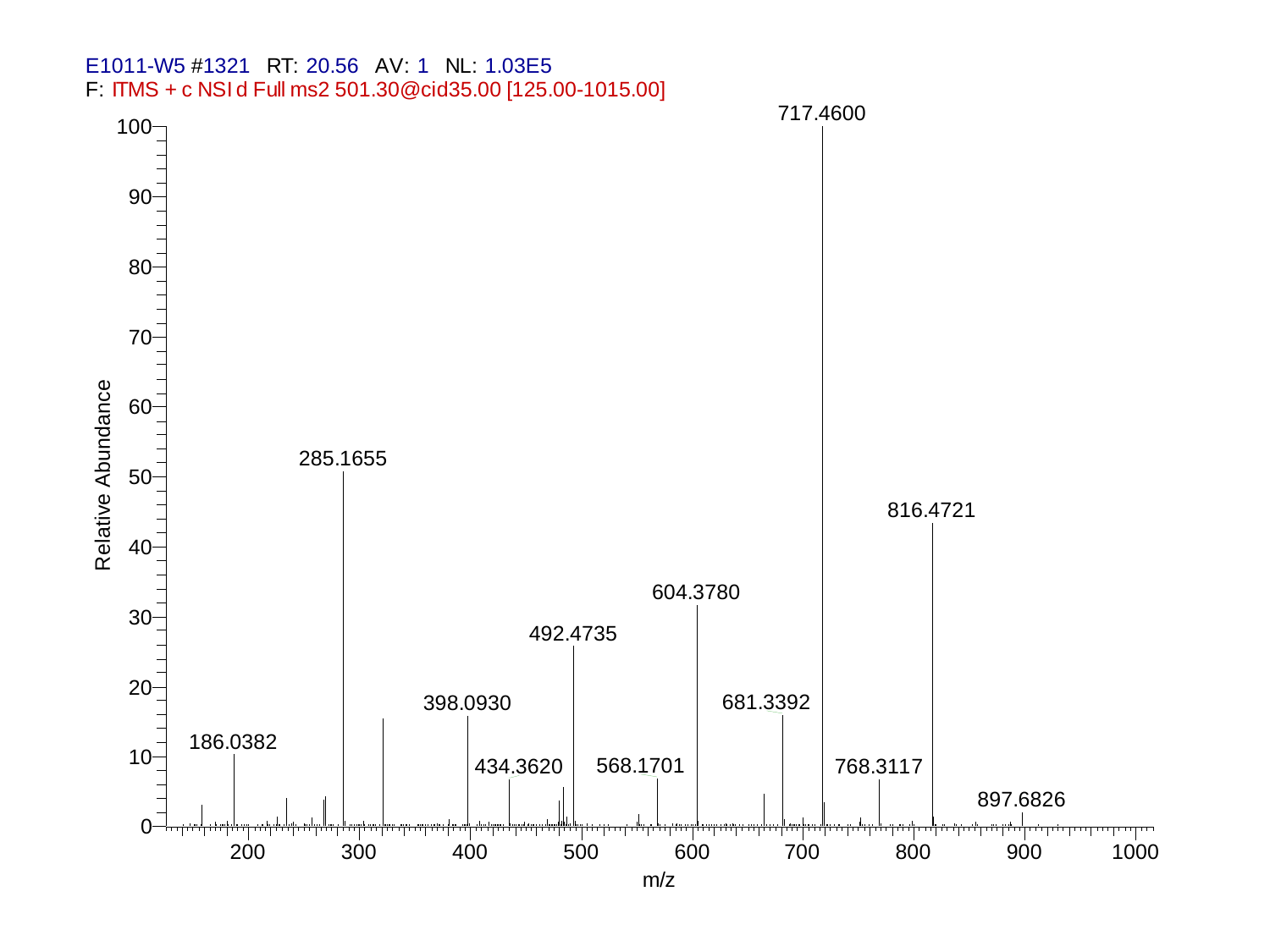

## Slide 54
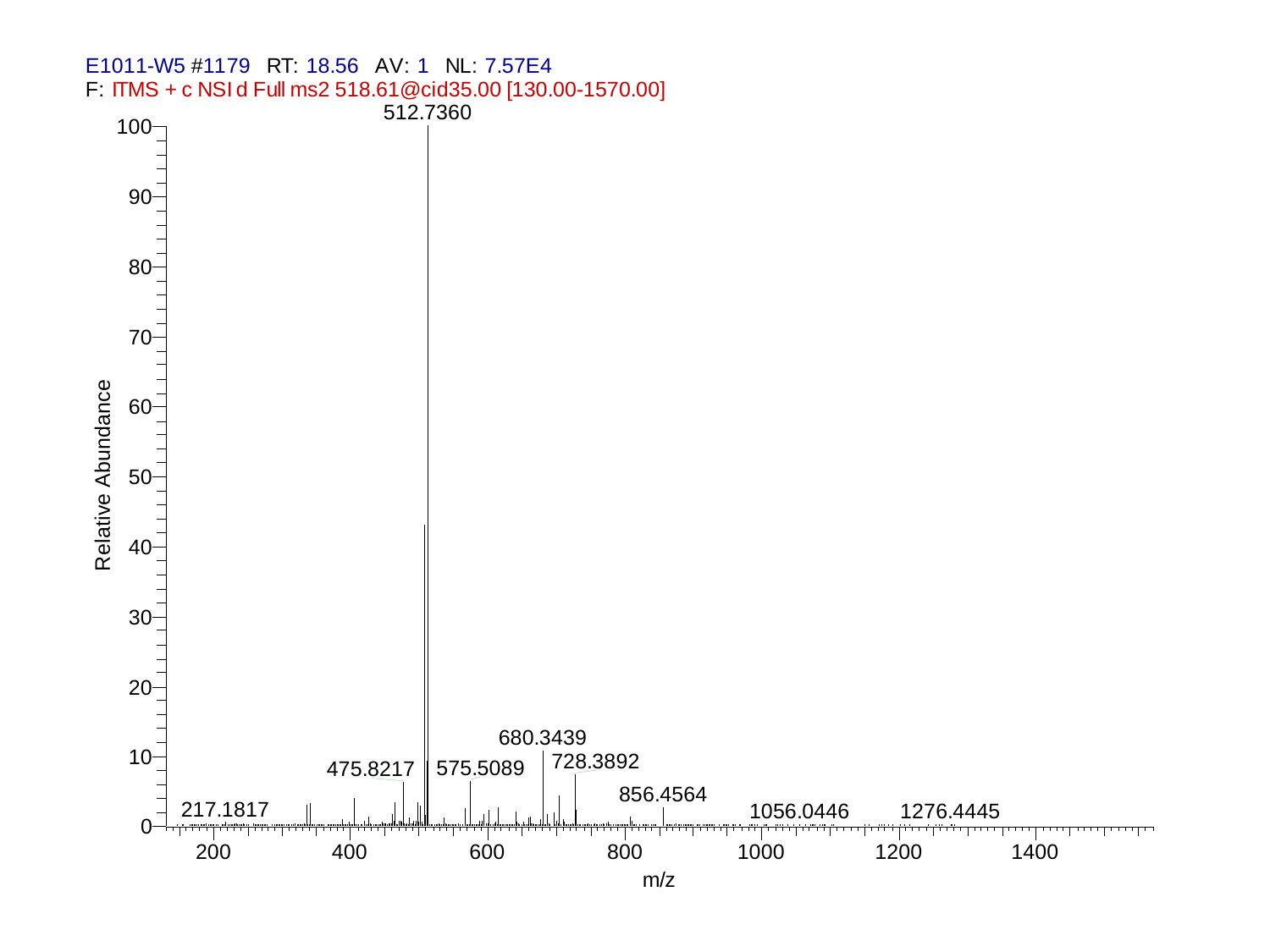

## Slide 55
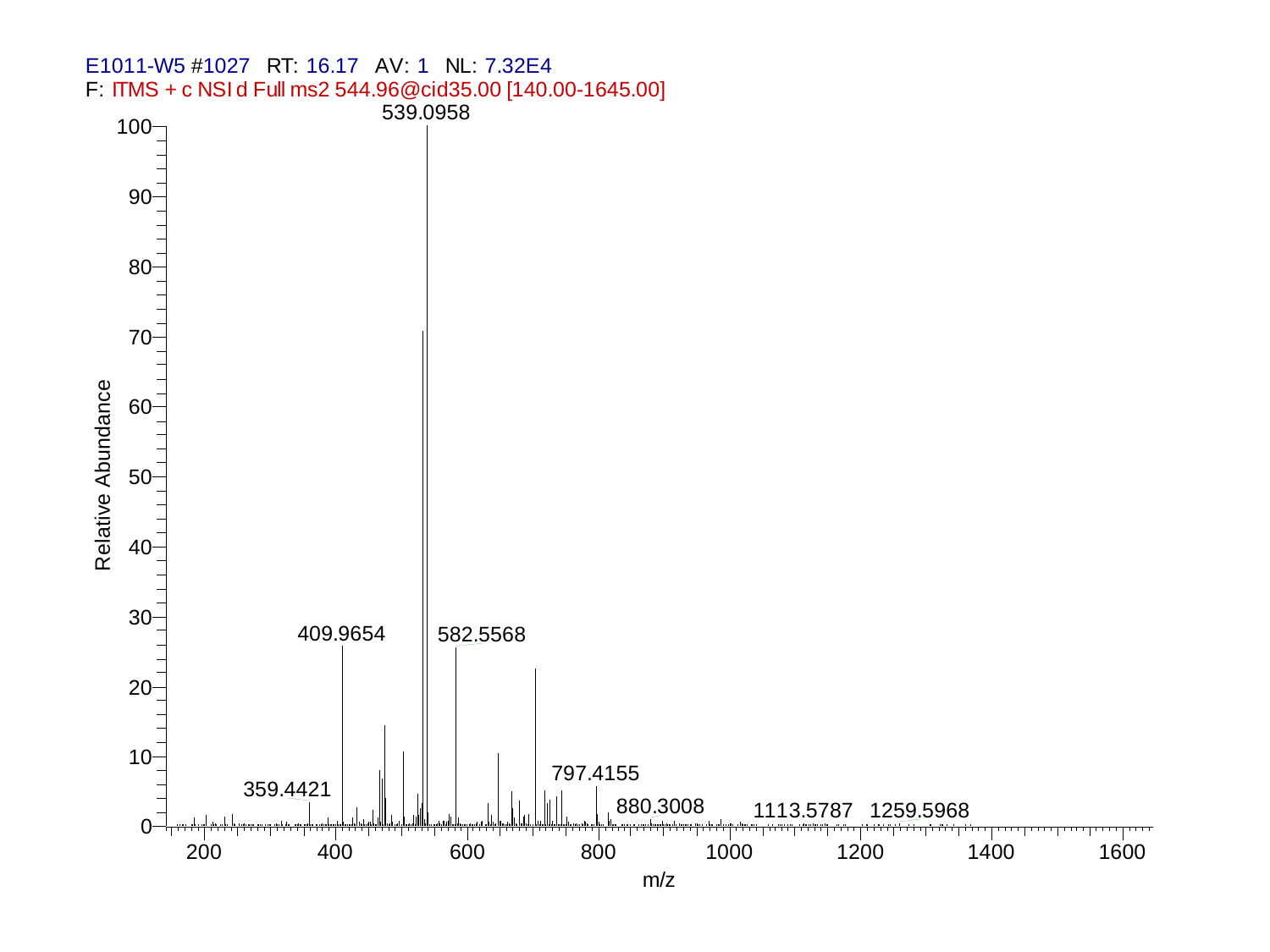

## Slide 56
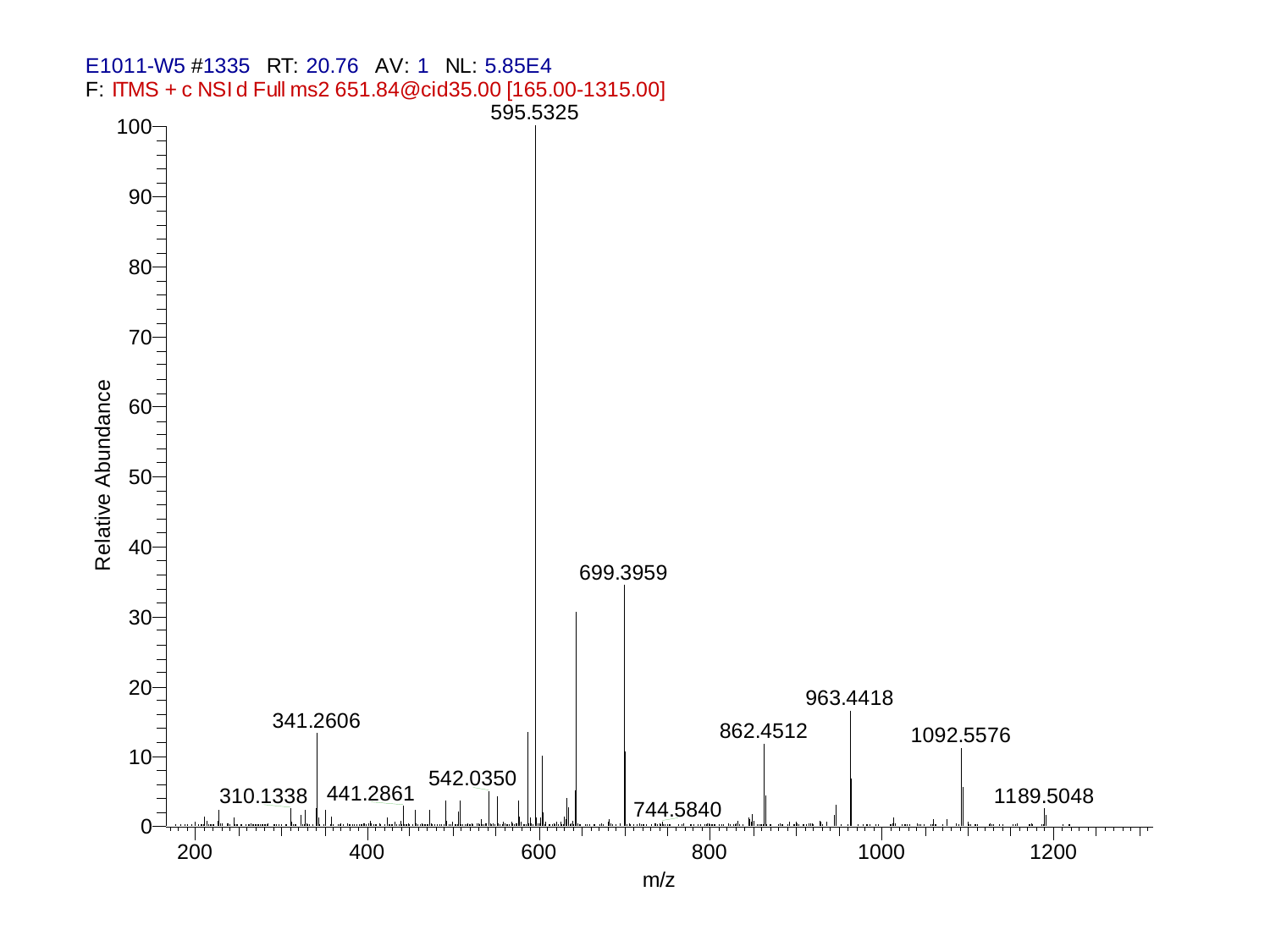

## Slide 57
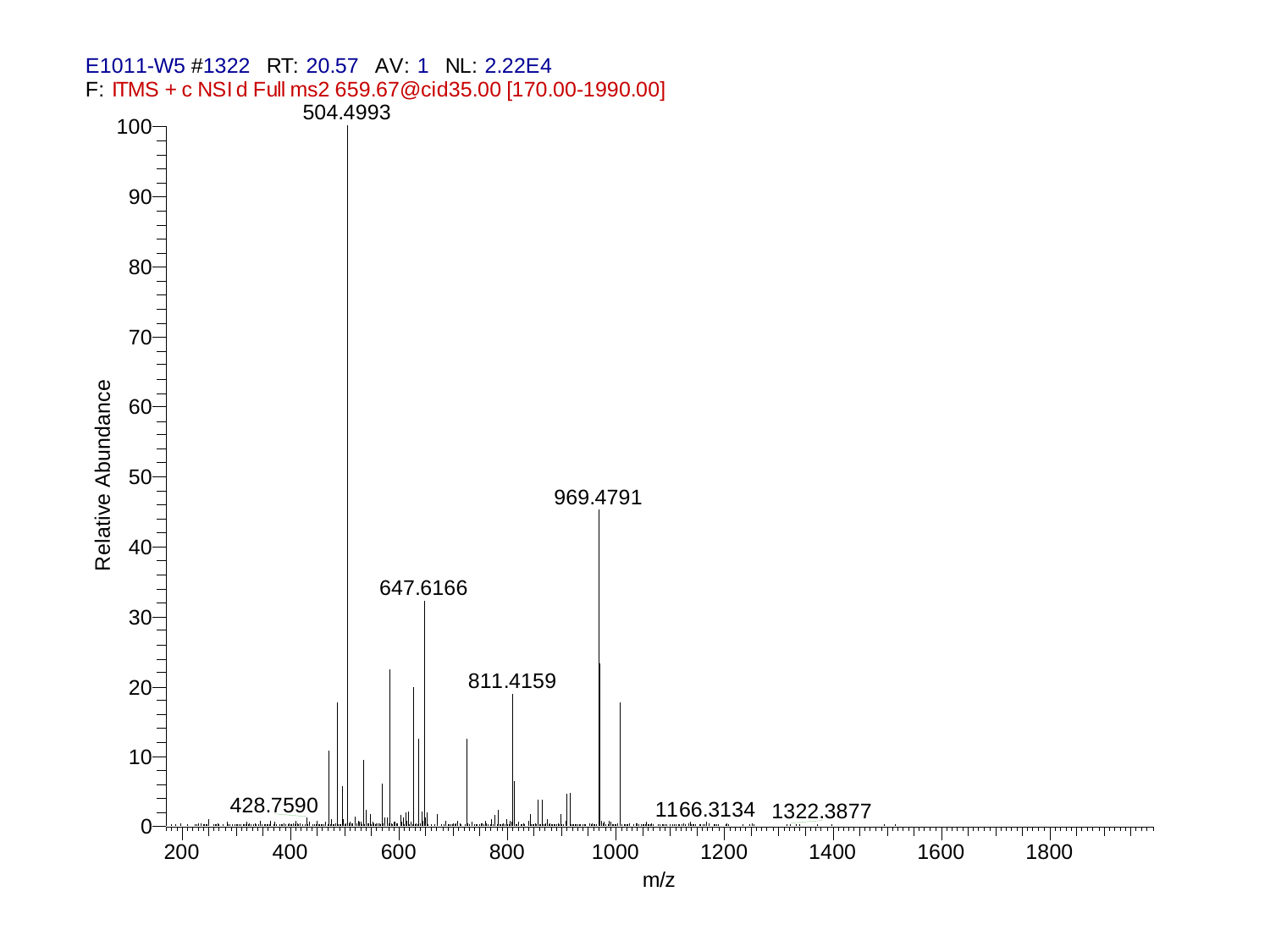

## Slide 58
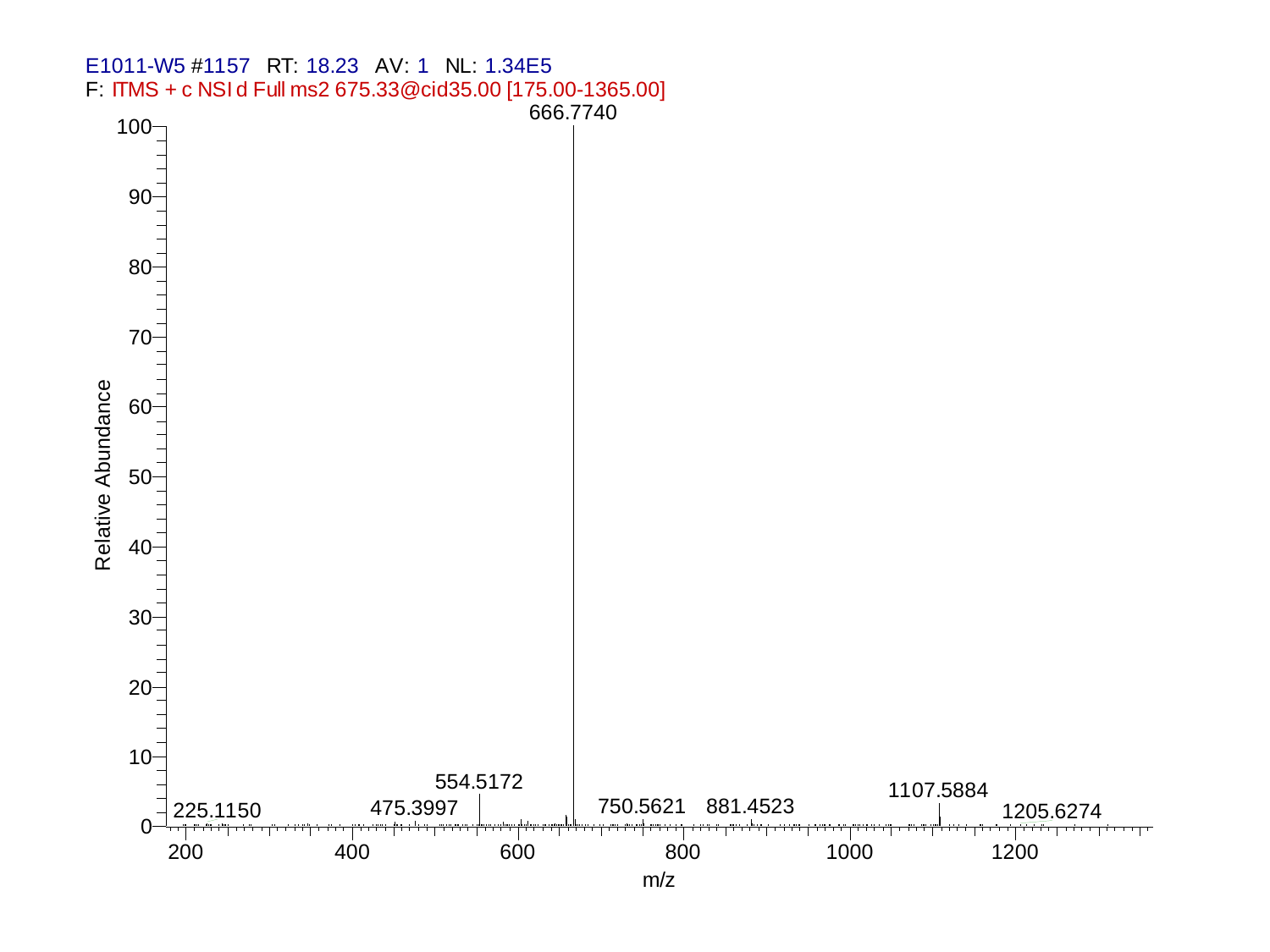

## Slide 59
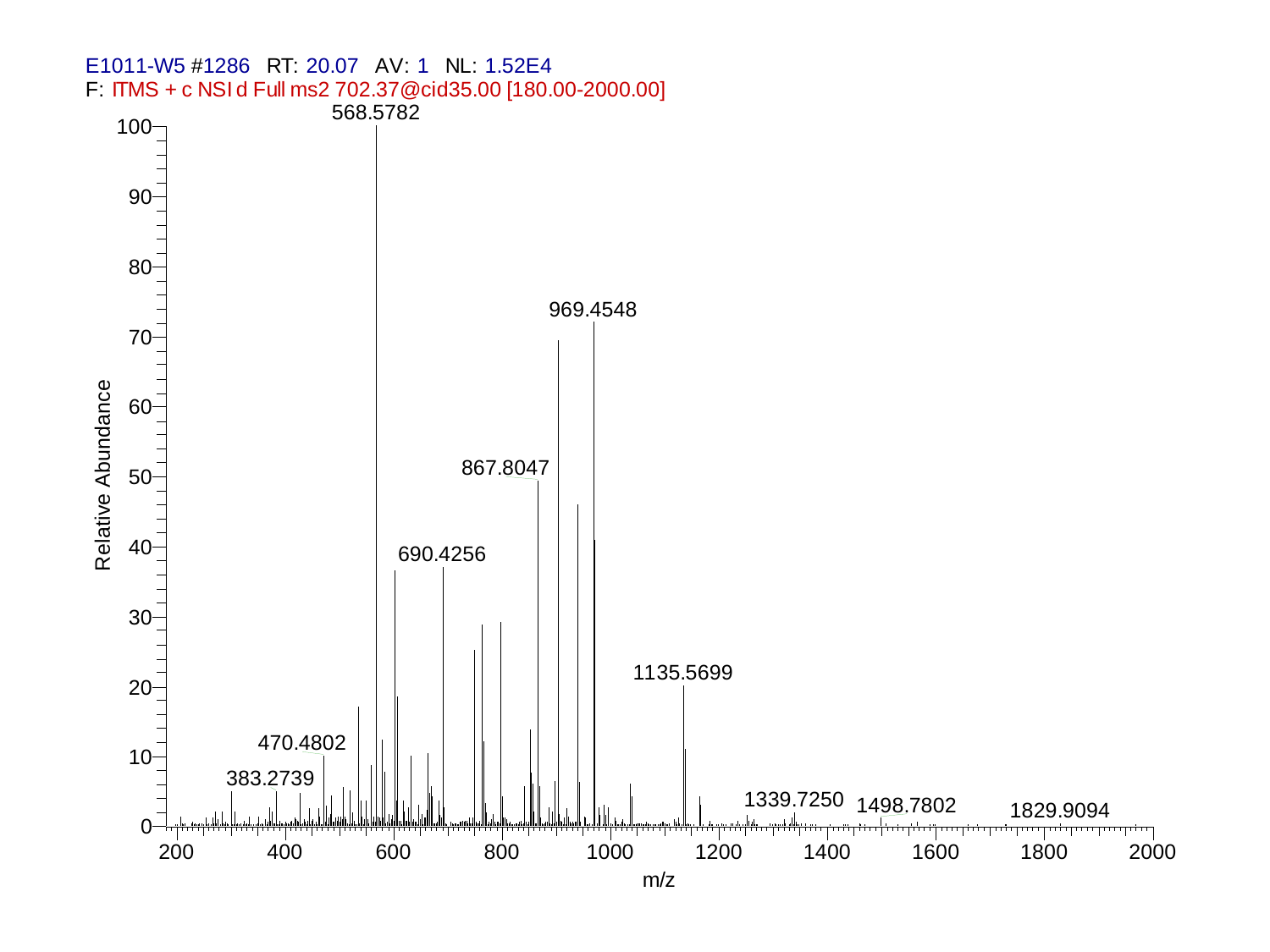

## Slide 60
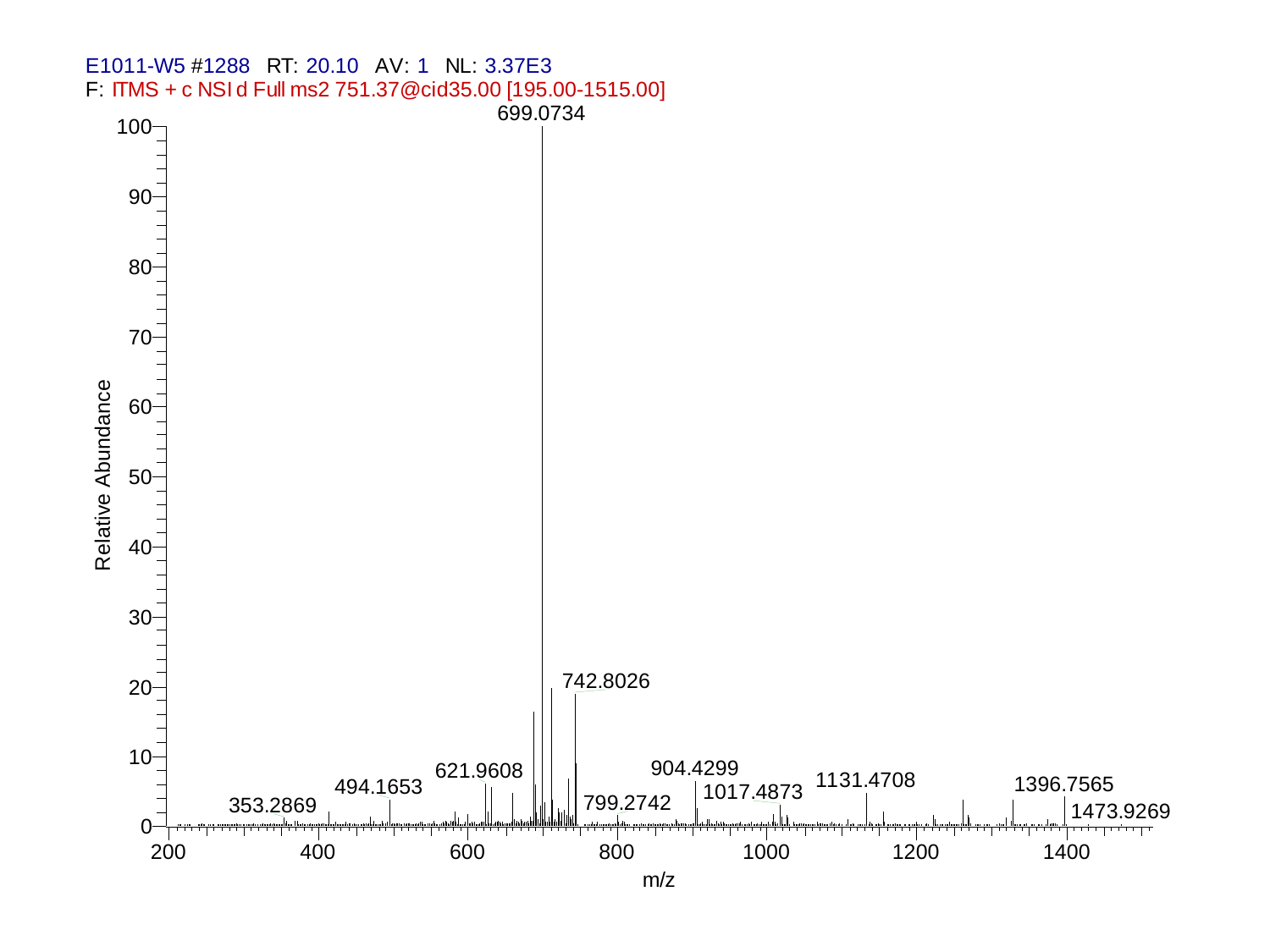

## Slide 61
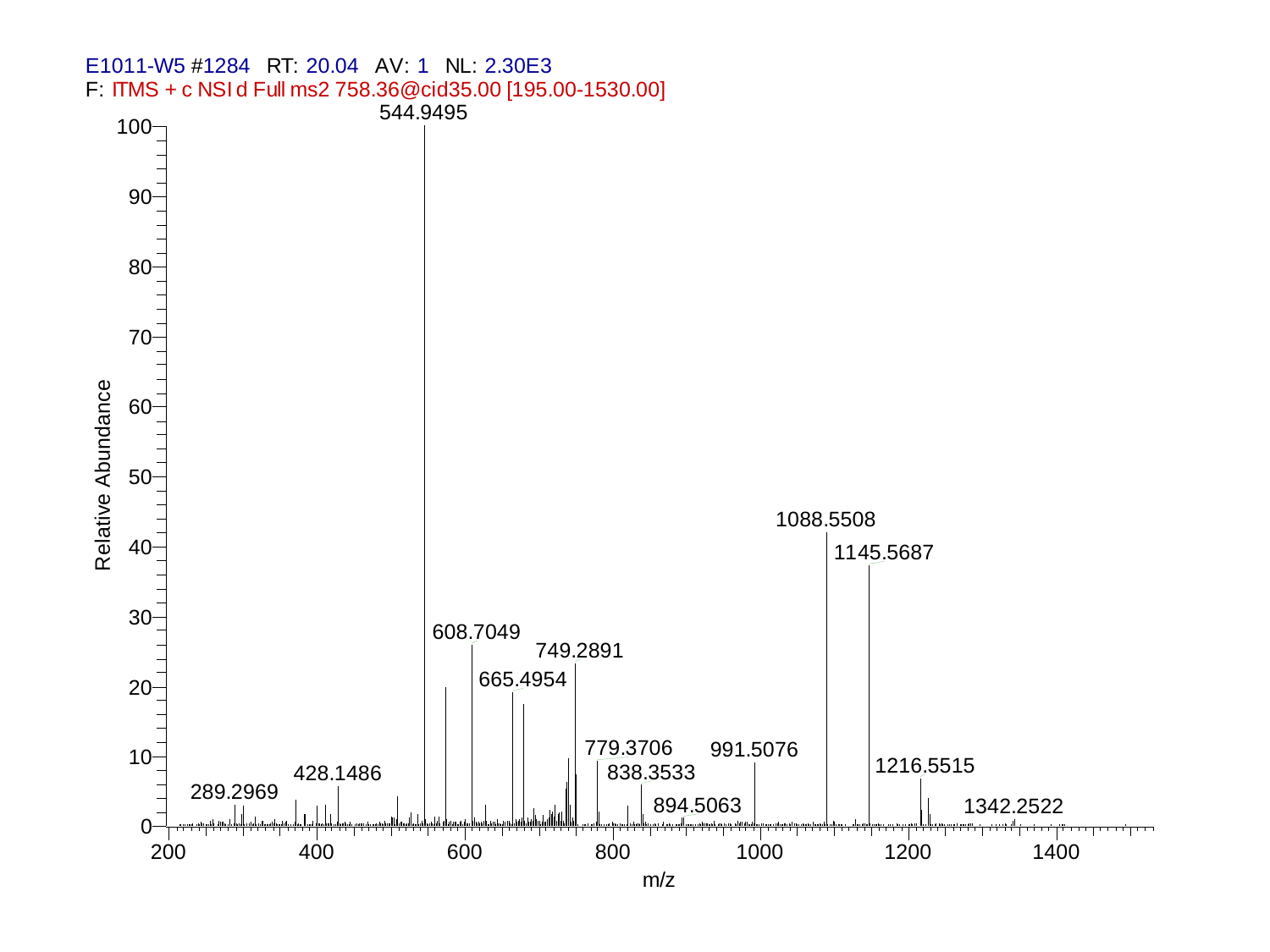

## Slide 62
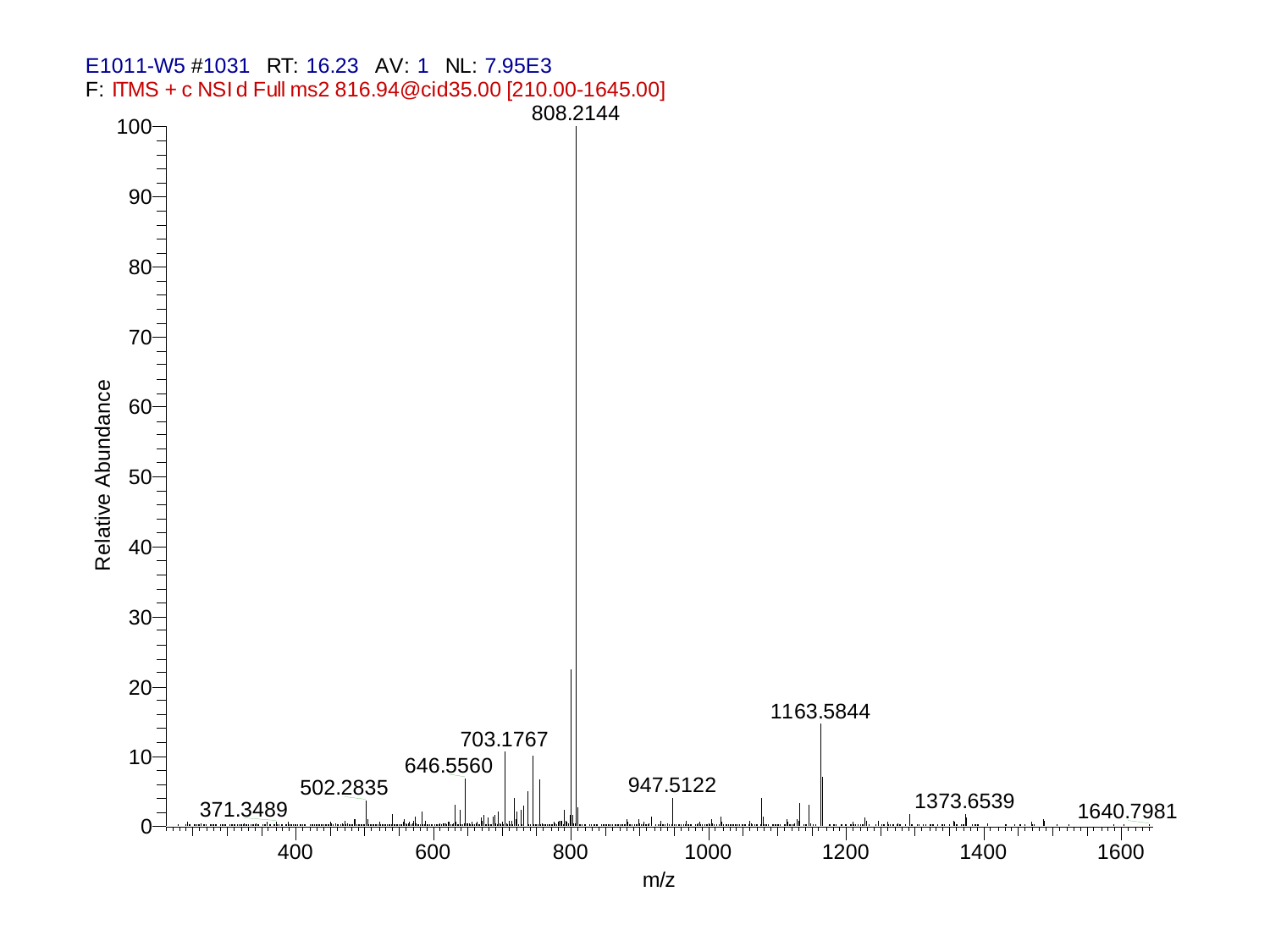

## Slide 63
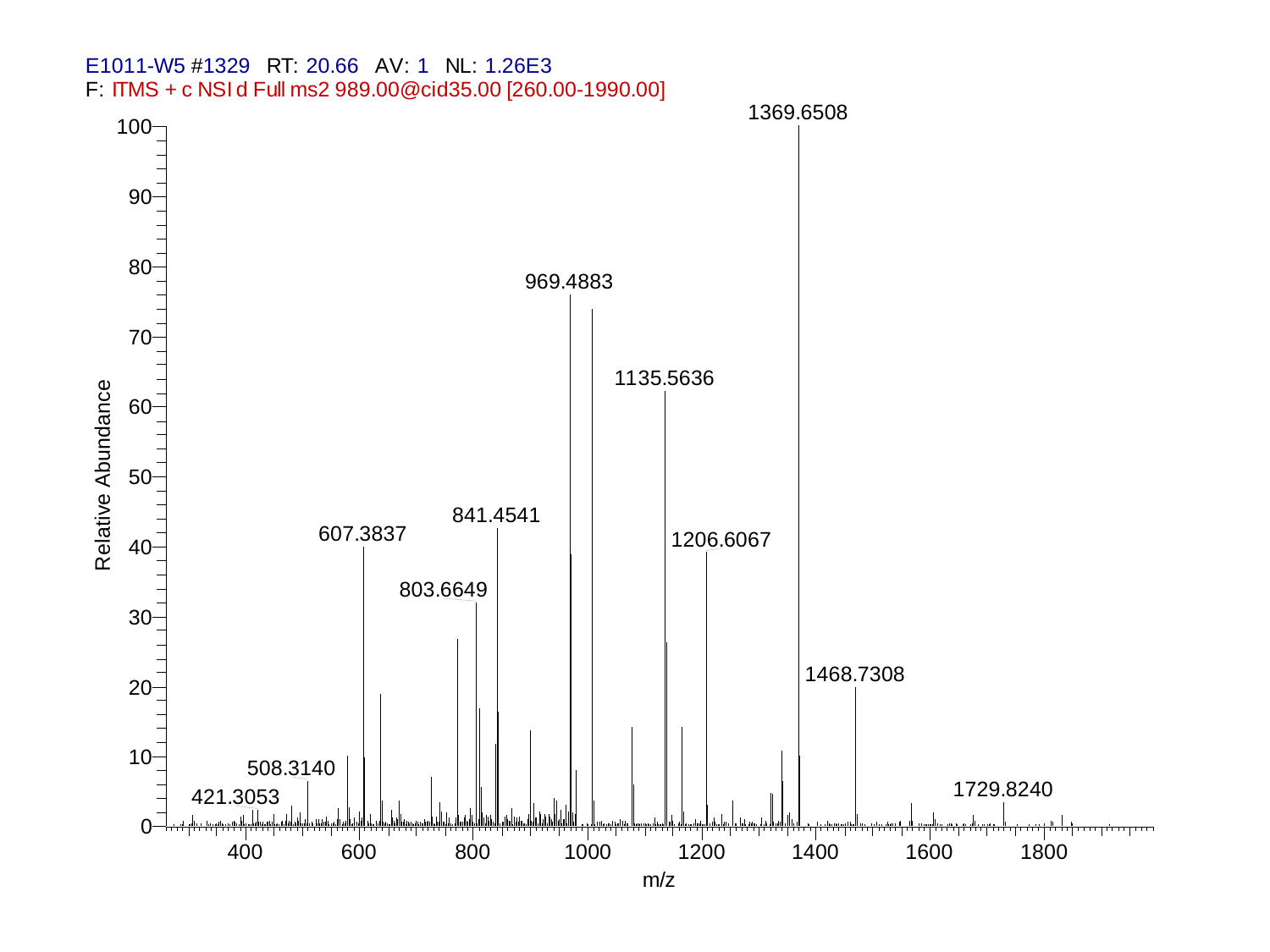

## Slide 64
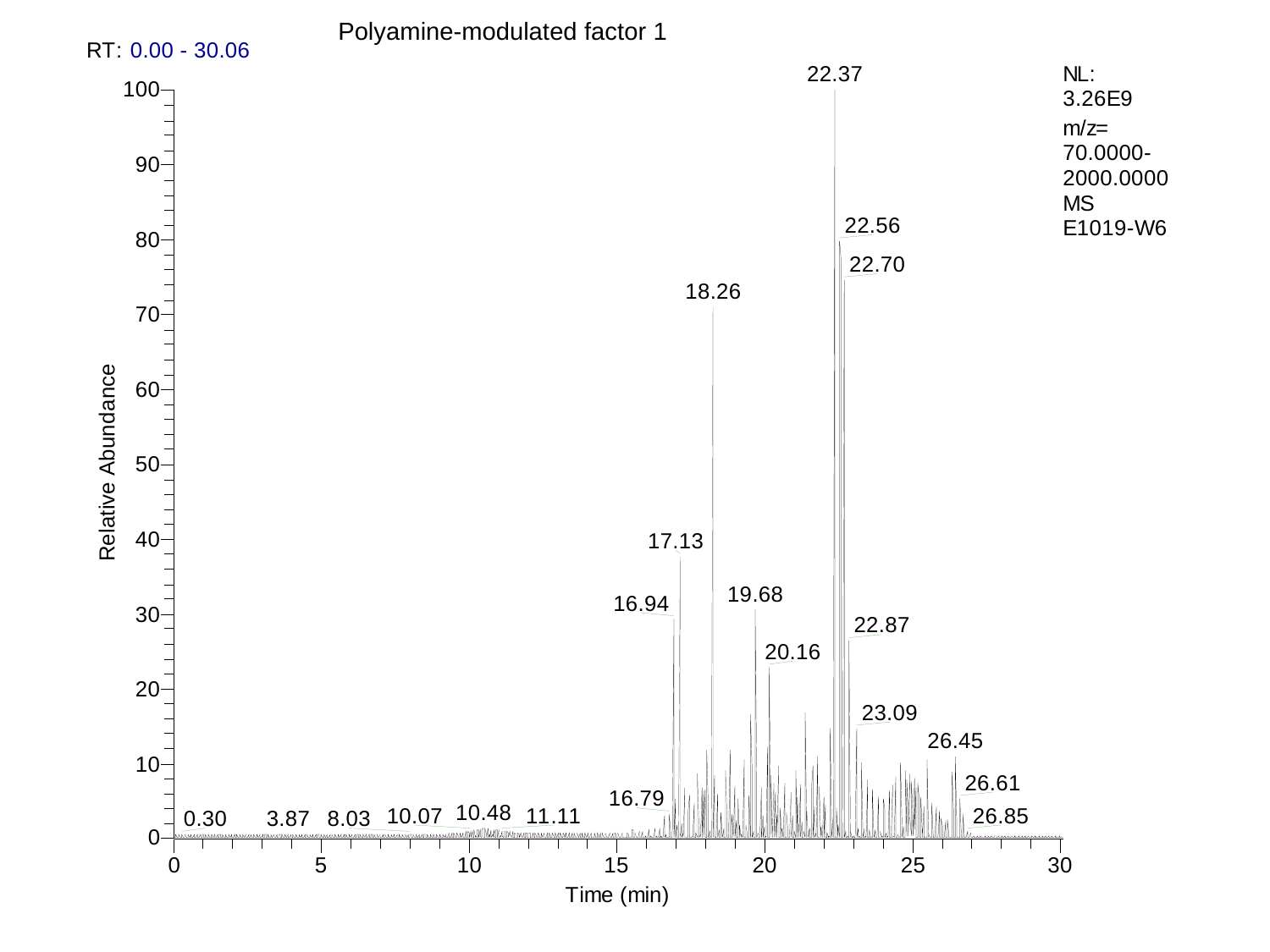

Polyamine-modulated factor 1

## Slide 65
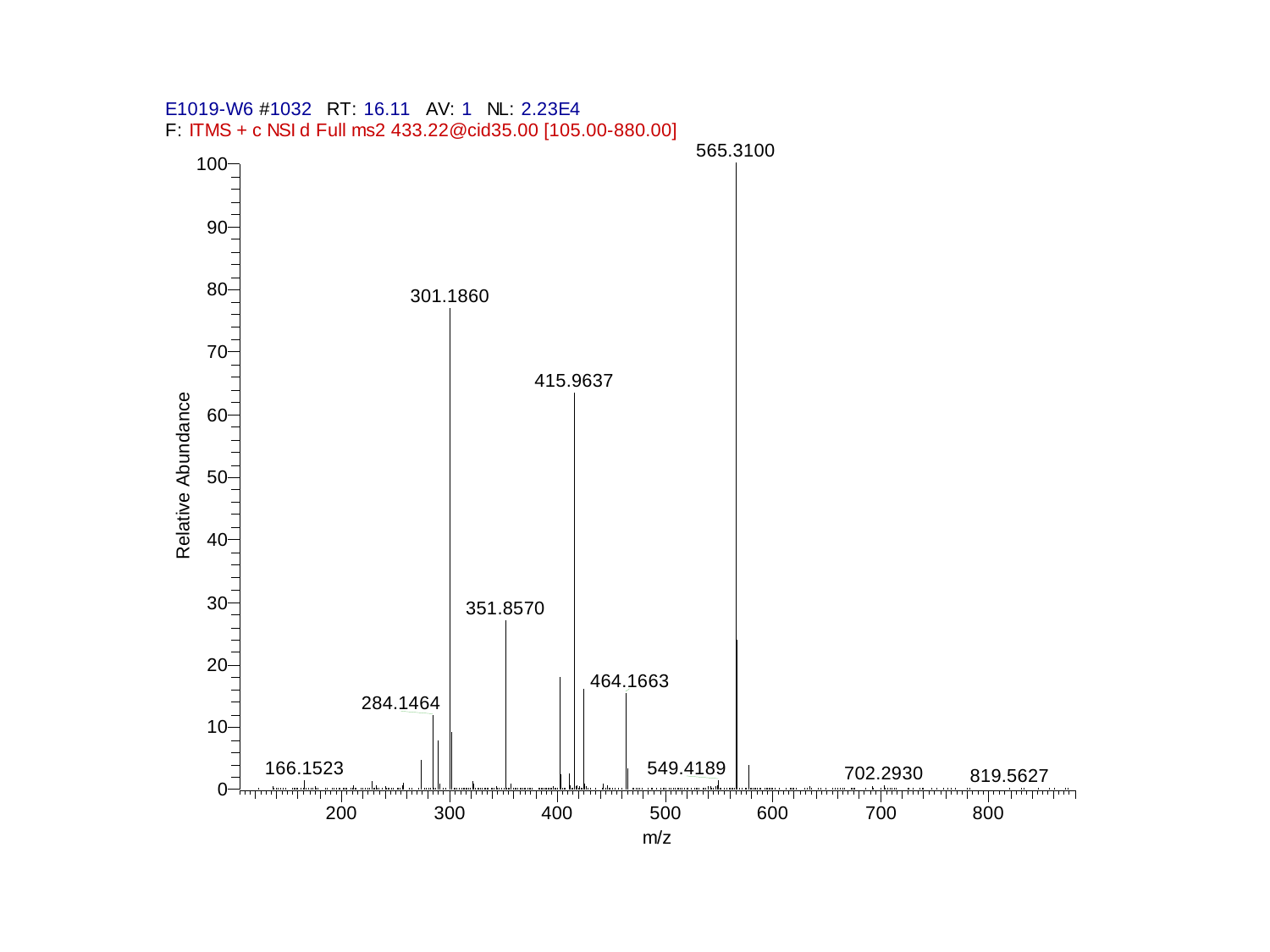

## Slide 66
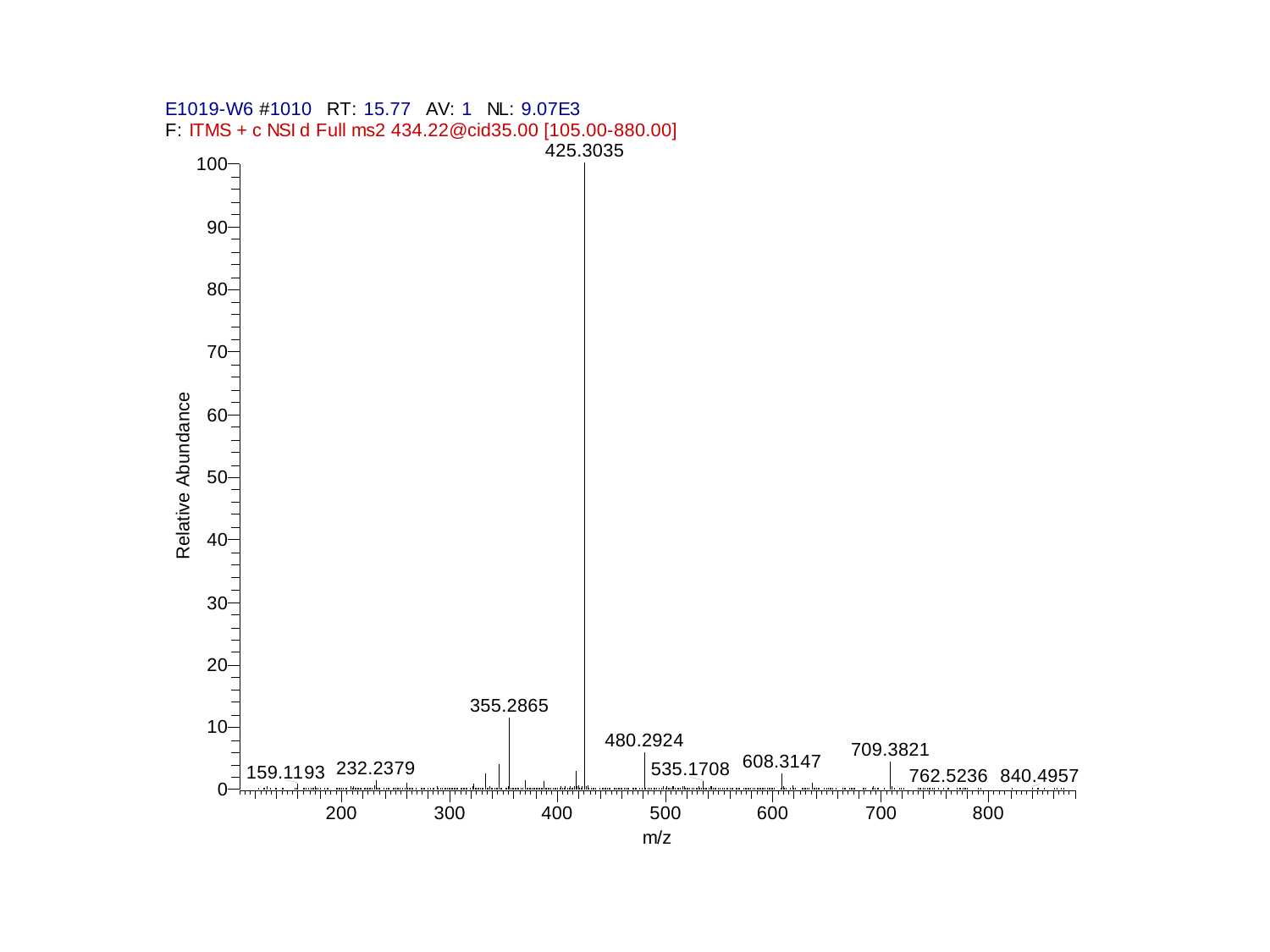

## Slide 67
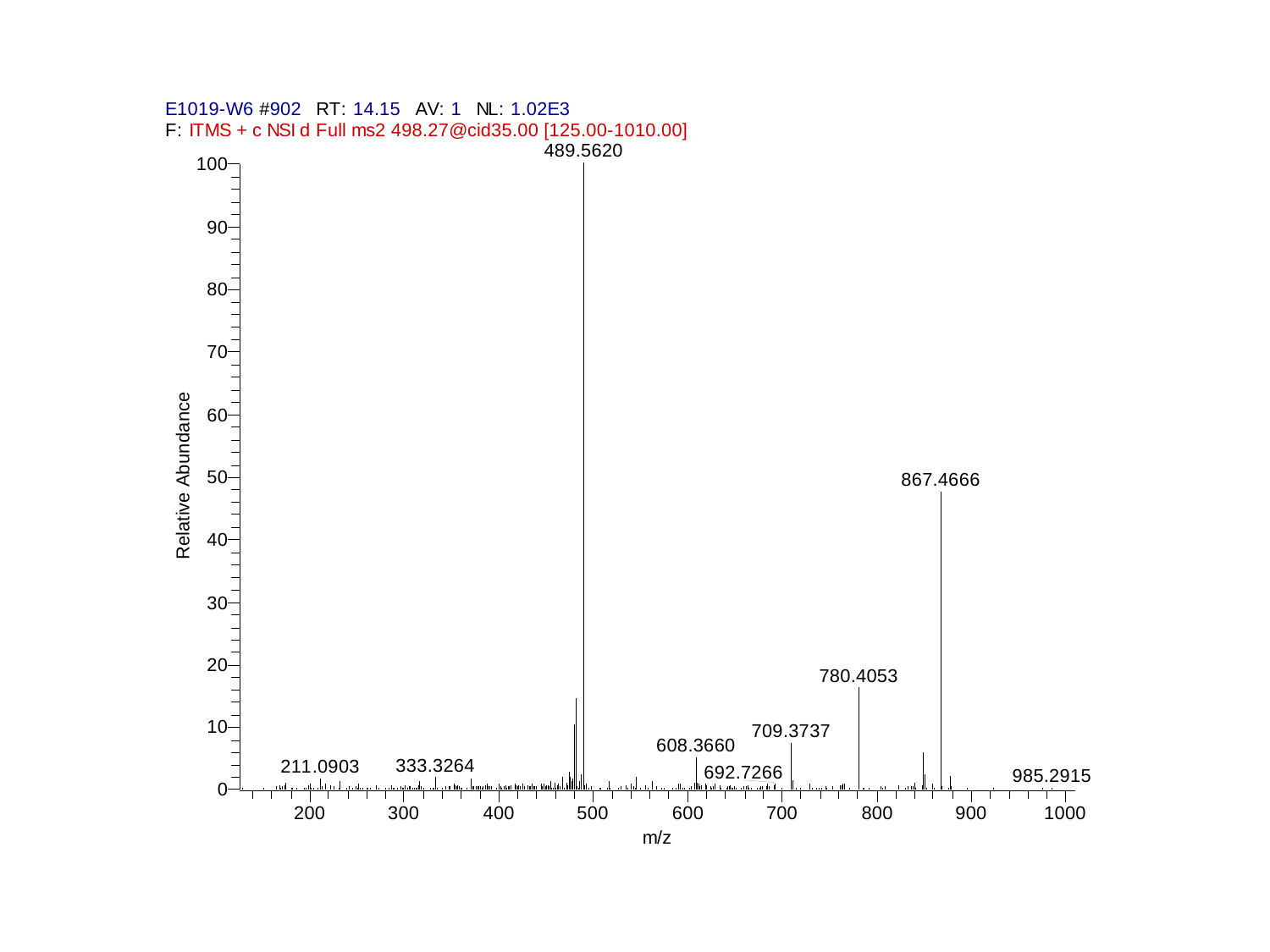

## Slide 68
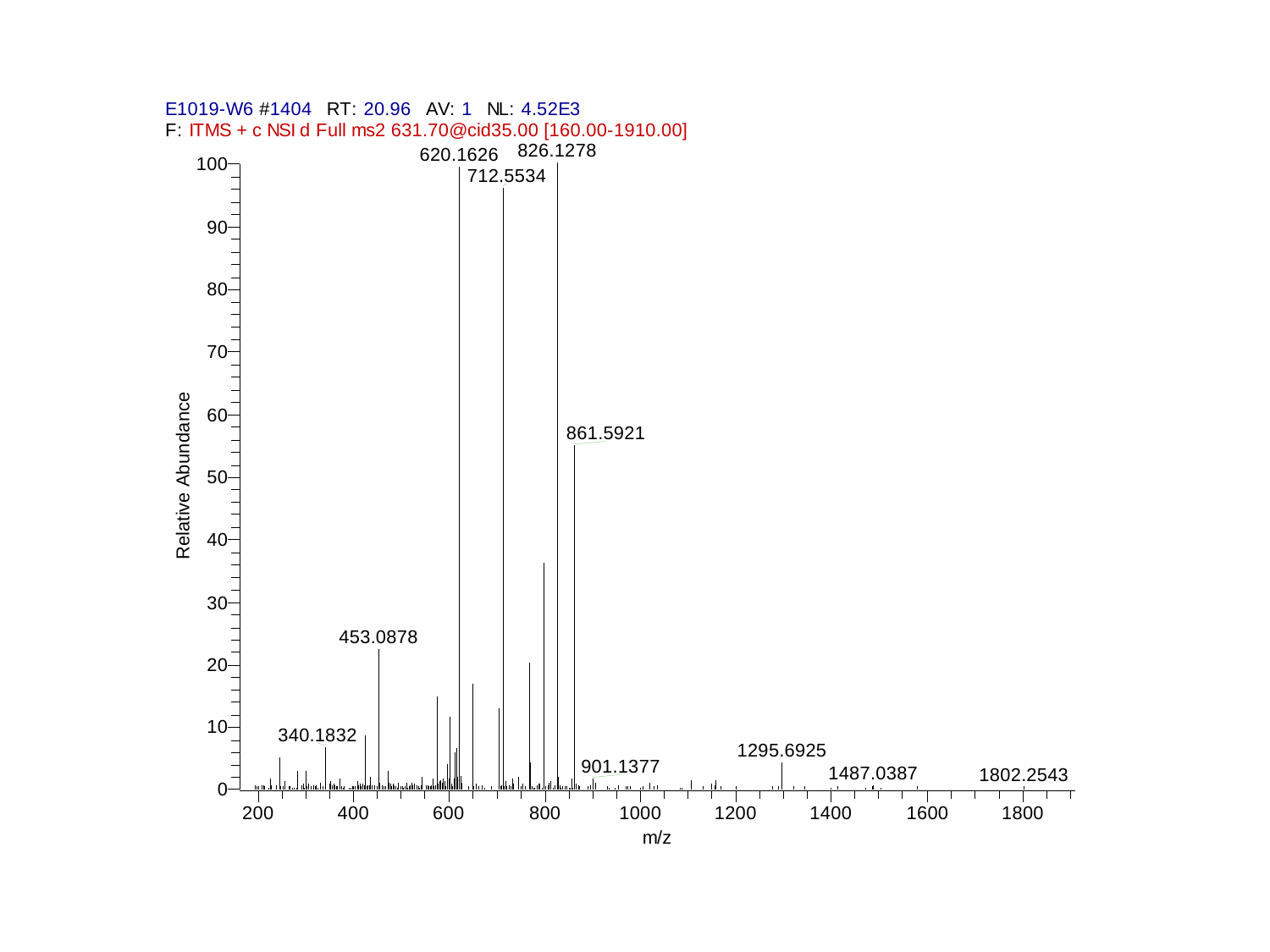

## Slide 69
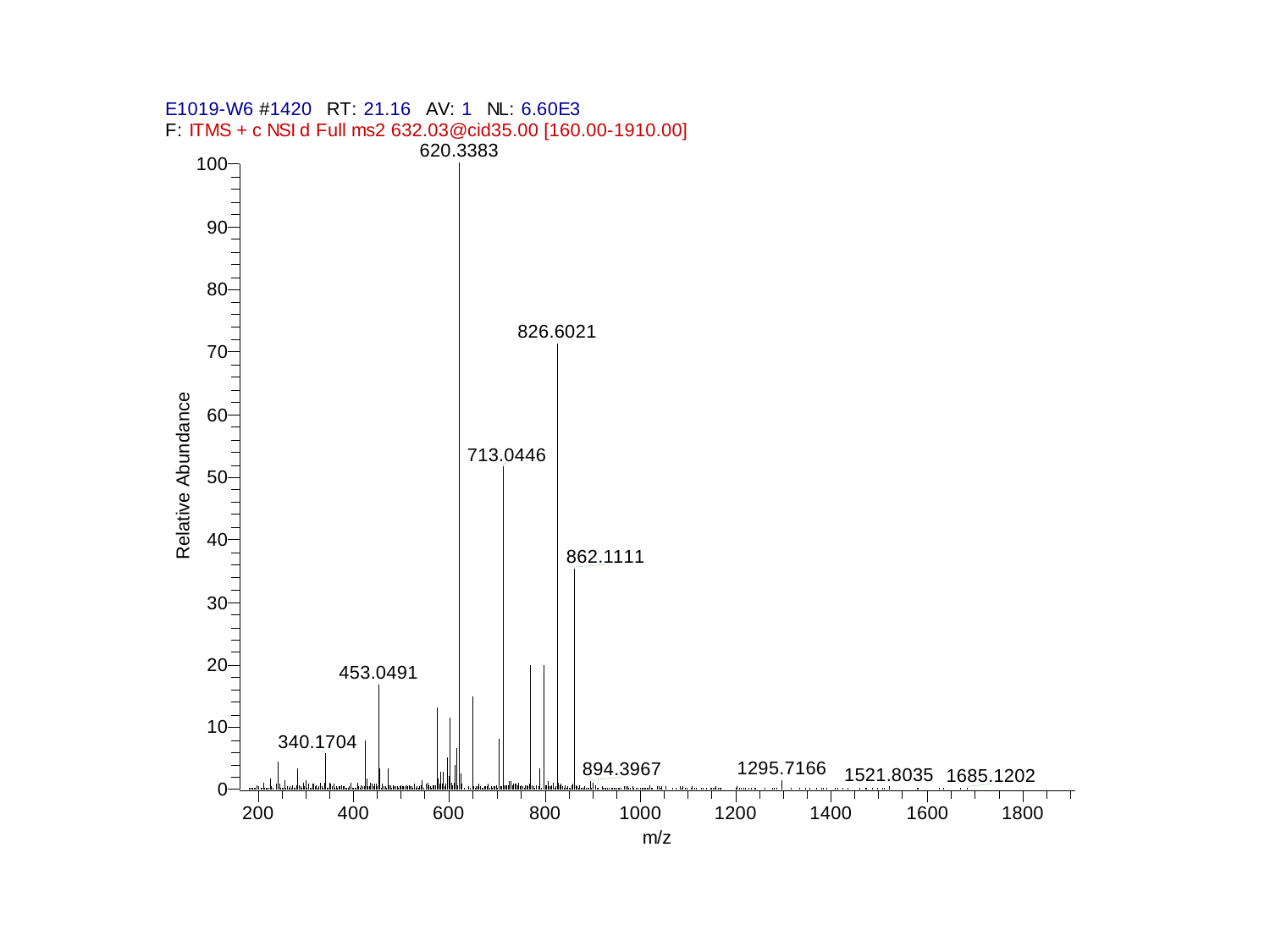

## Slide 70
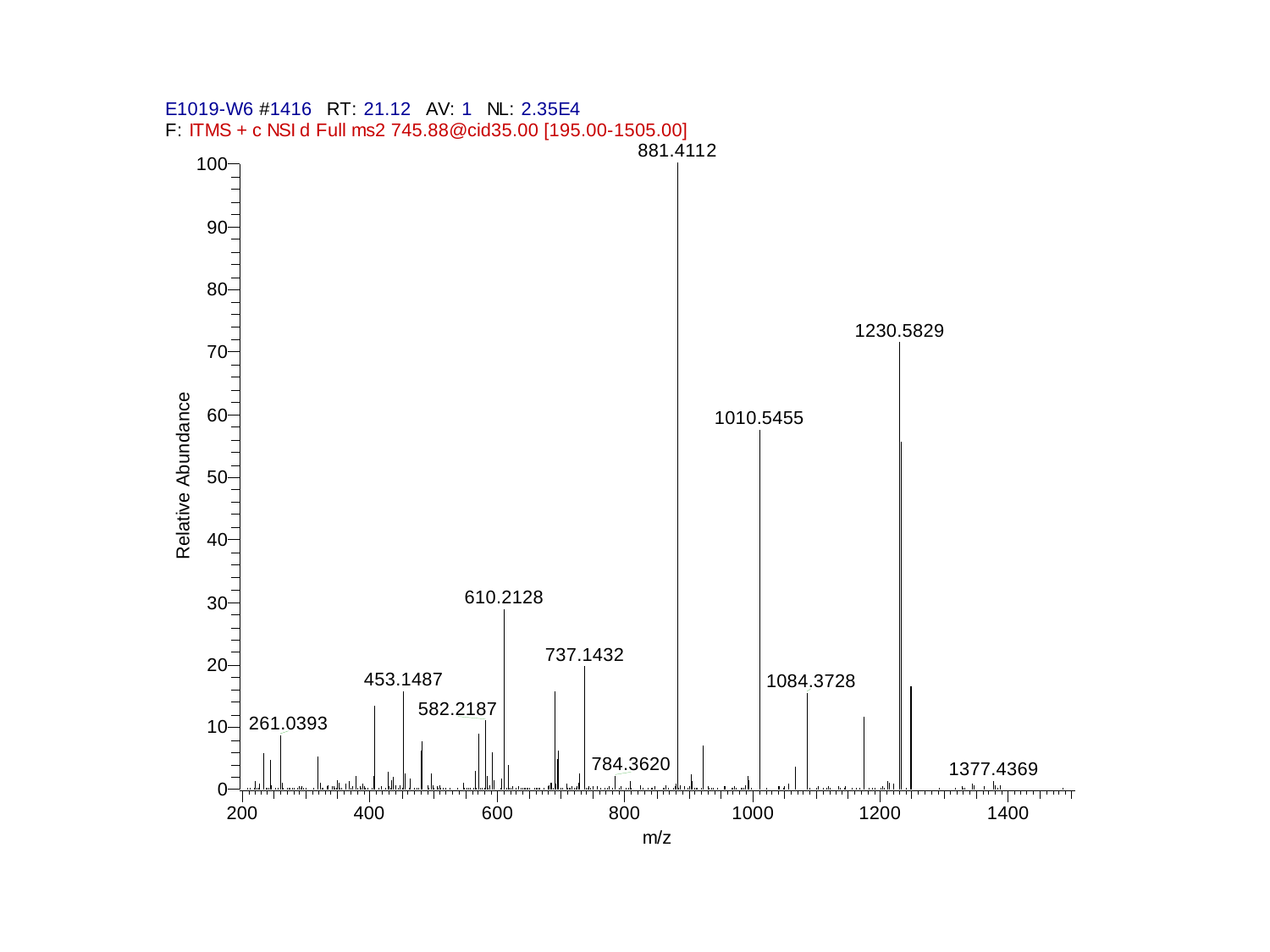

## Slide 71
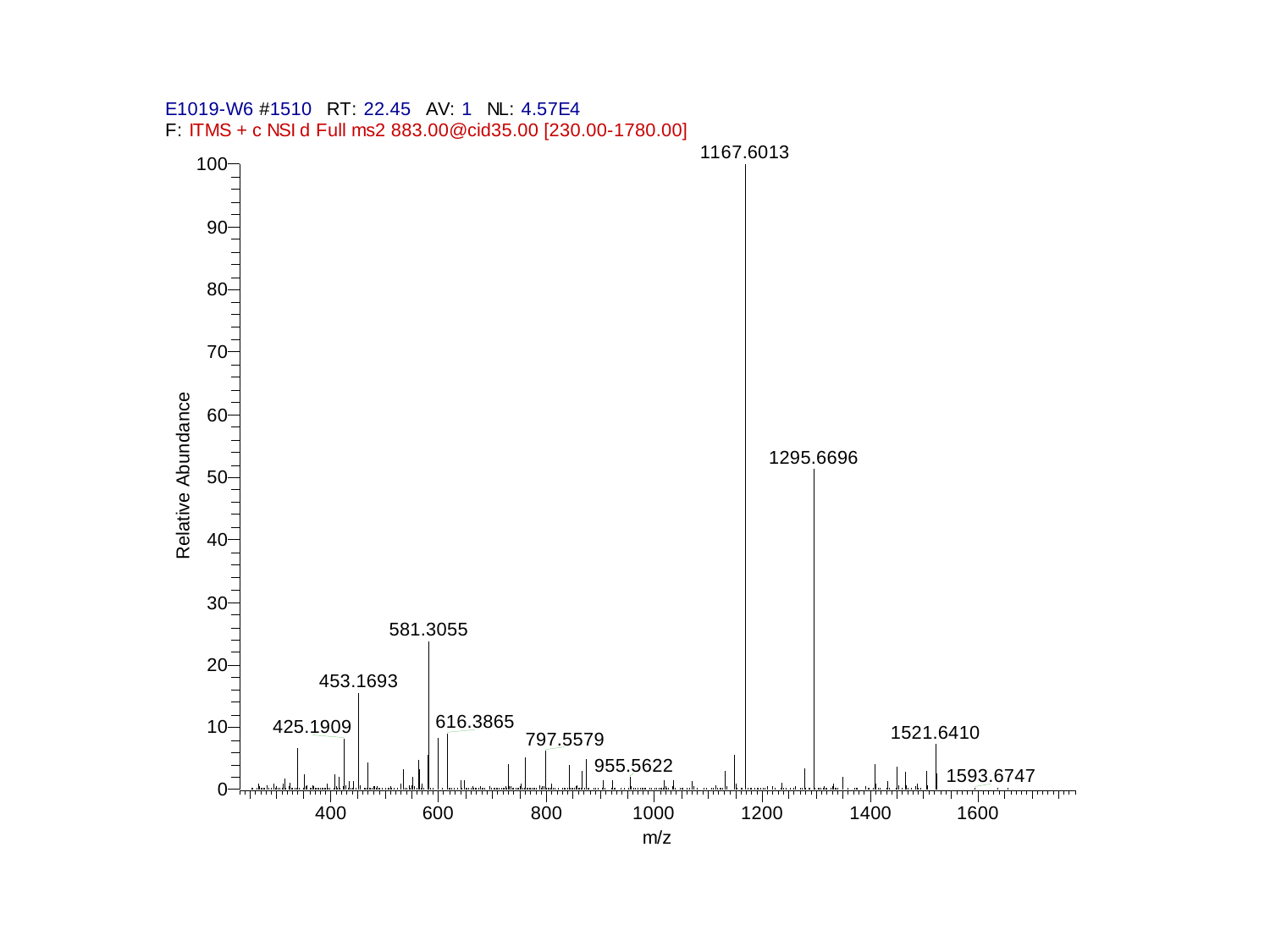

## Slide 72
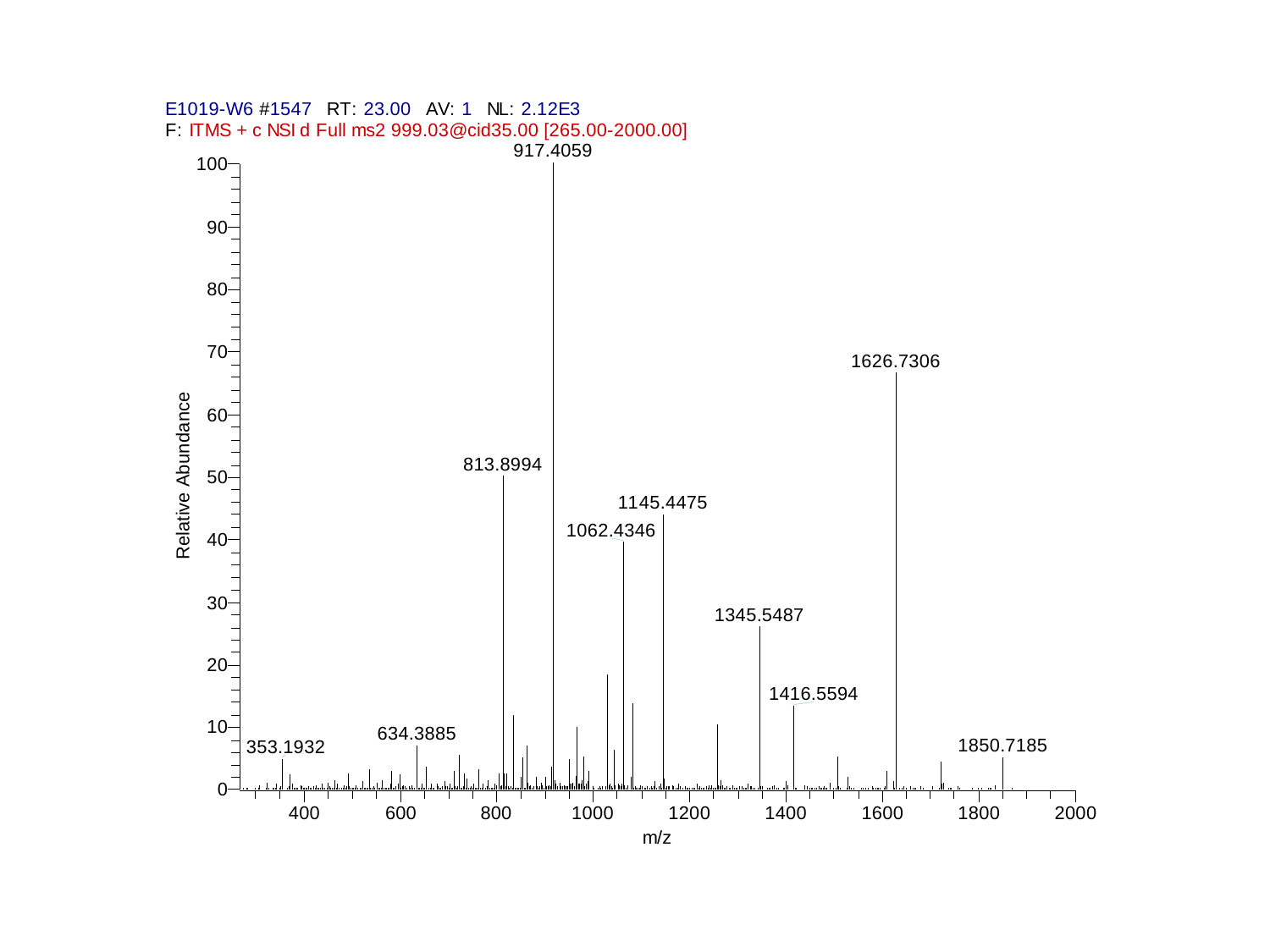

## Slide 73
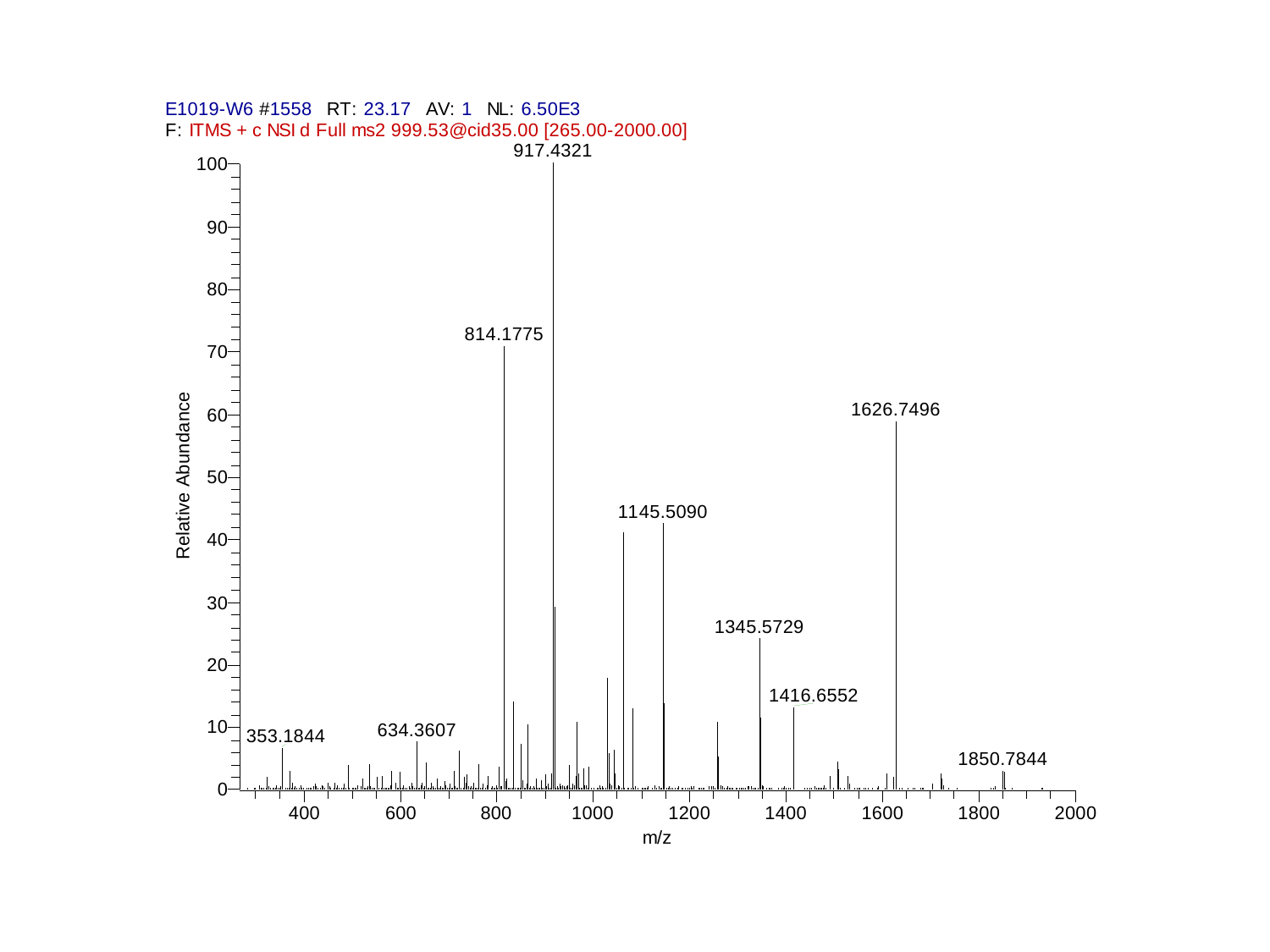

## Slide 74
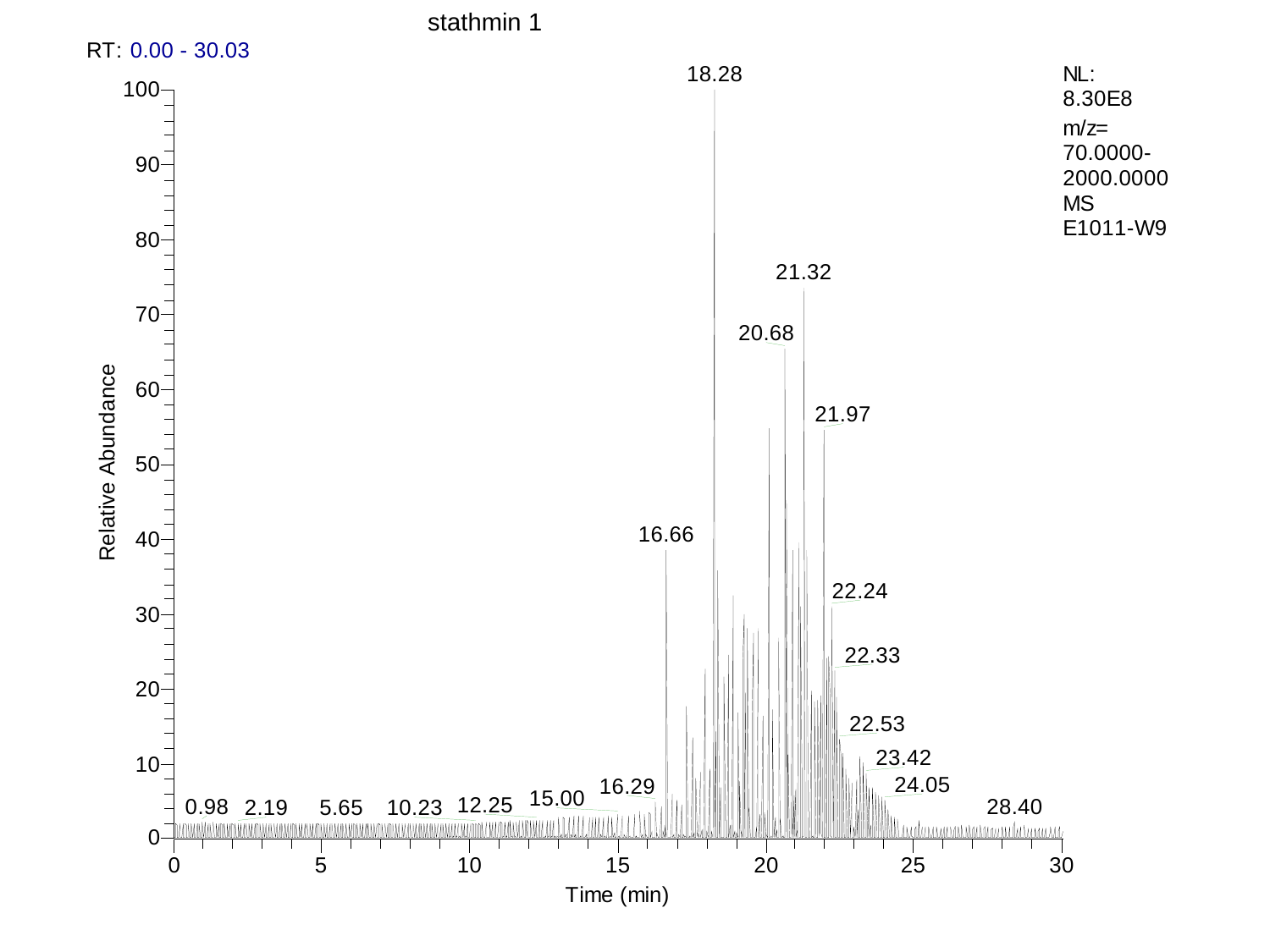

stathmin 1

## Slide 75
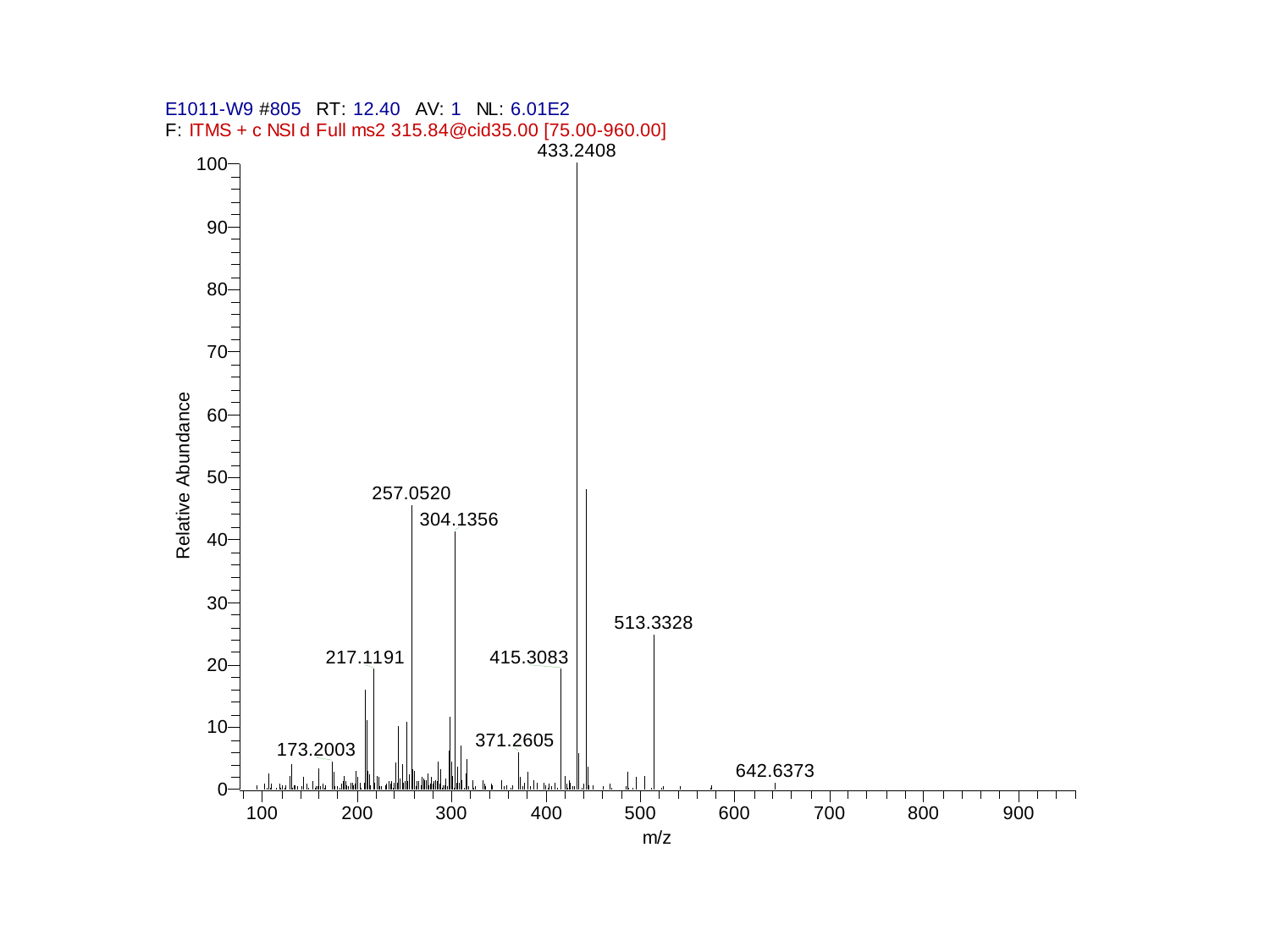

## Slide 76
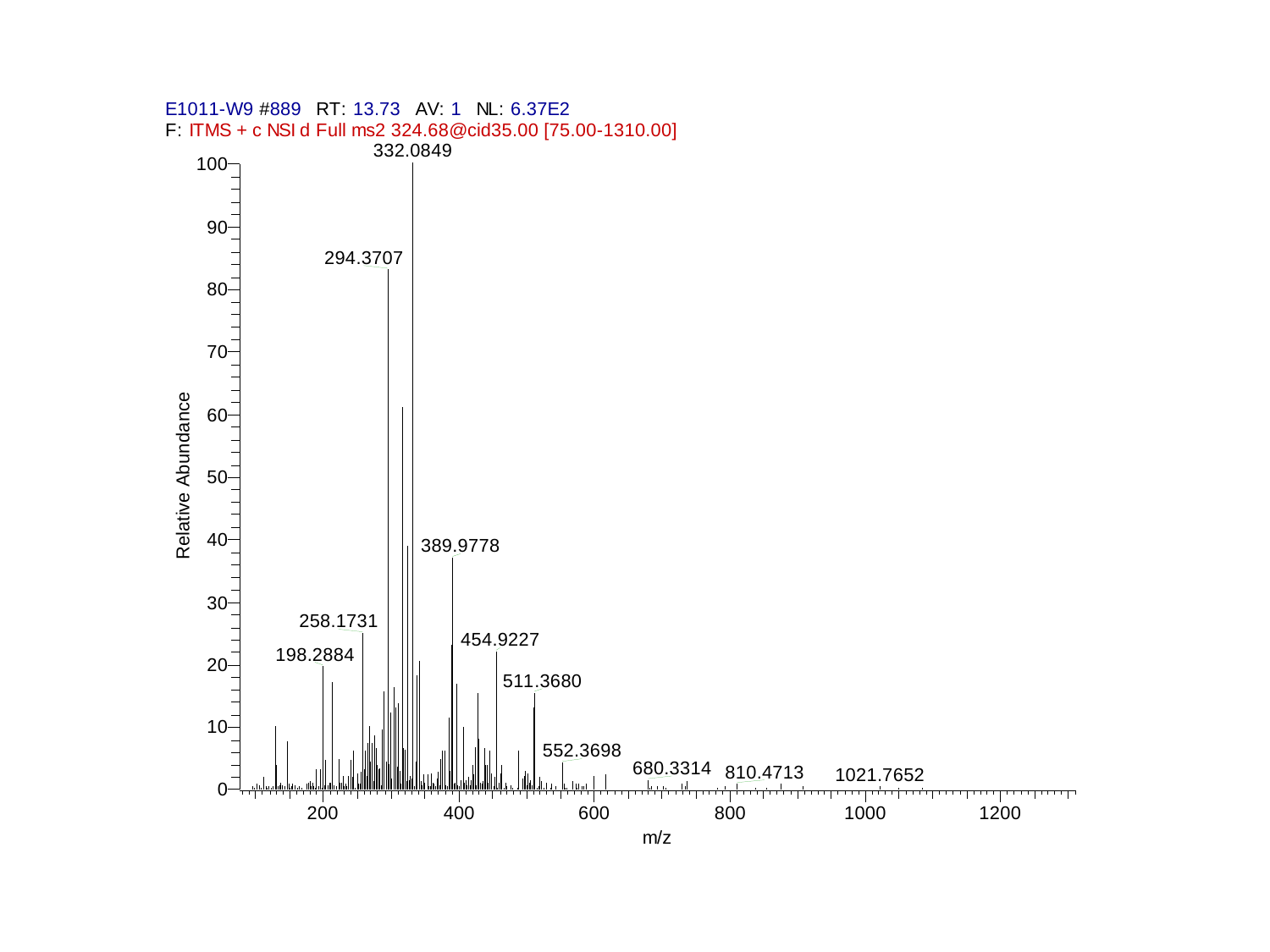

## Slide 77
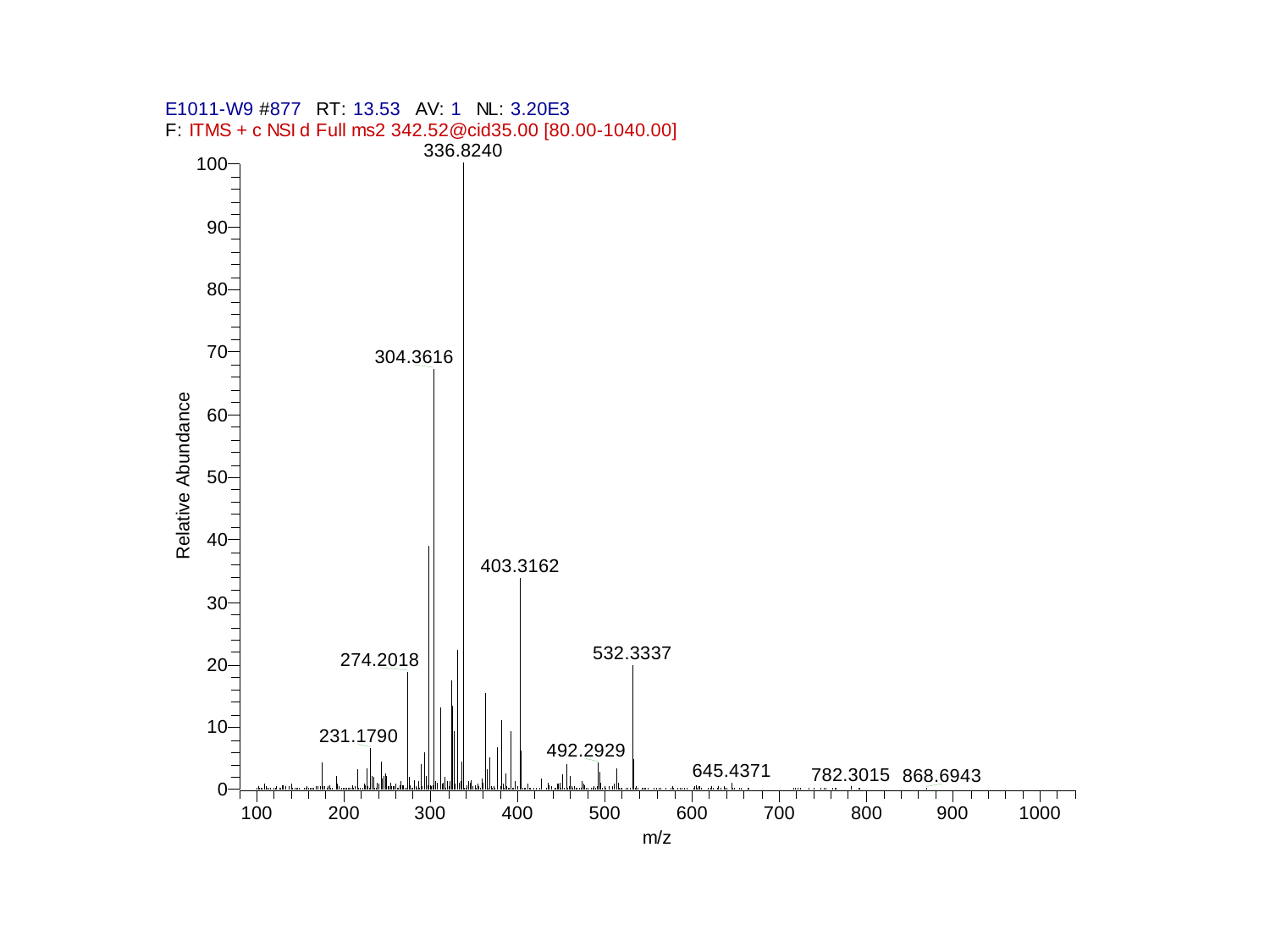

## Slide 78
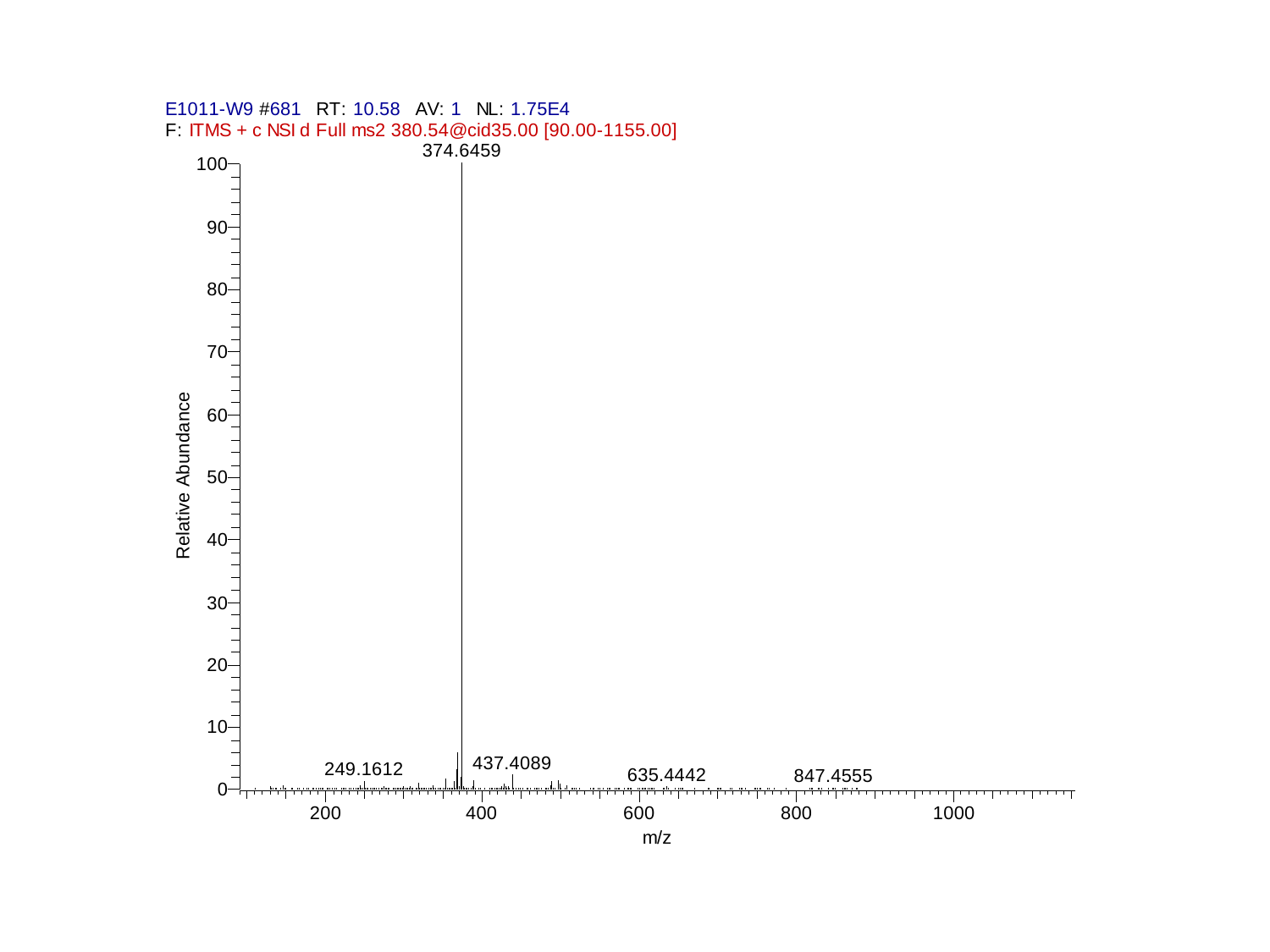

## Slide 79
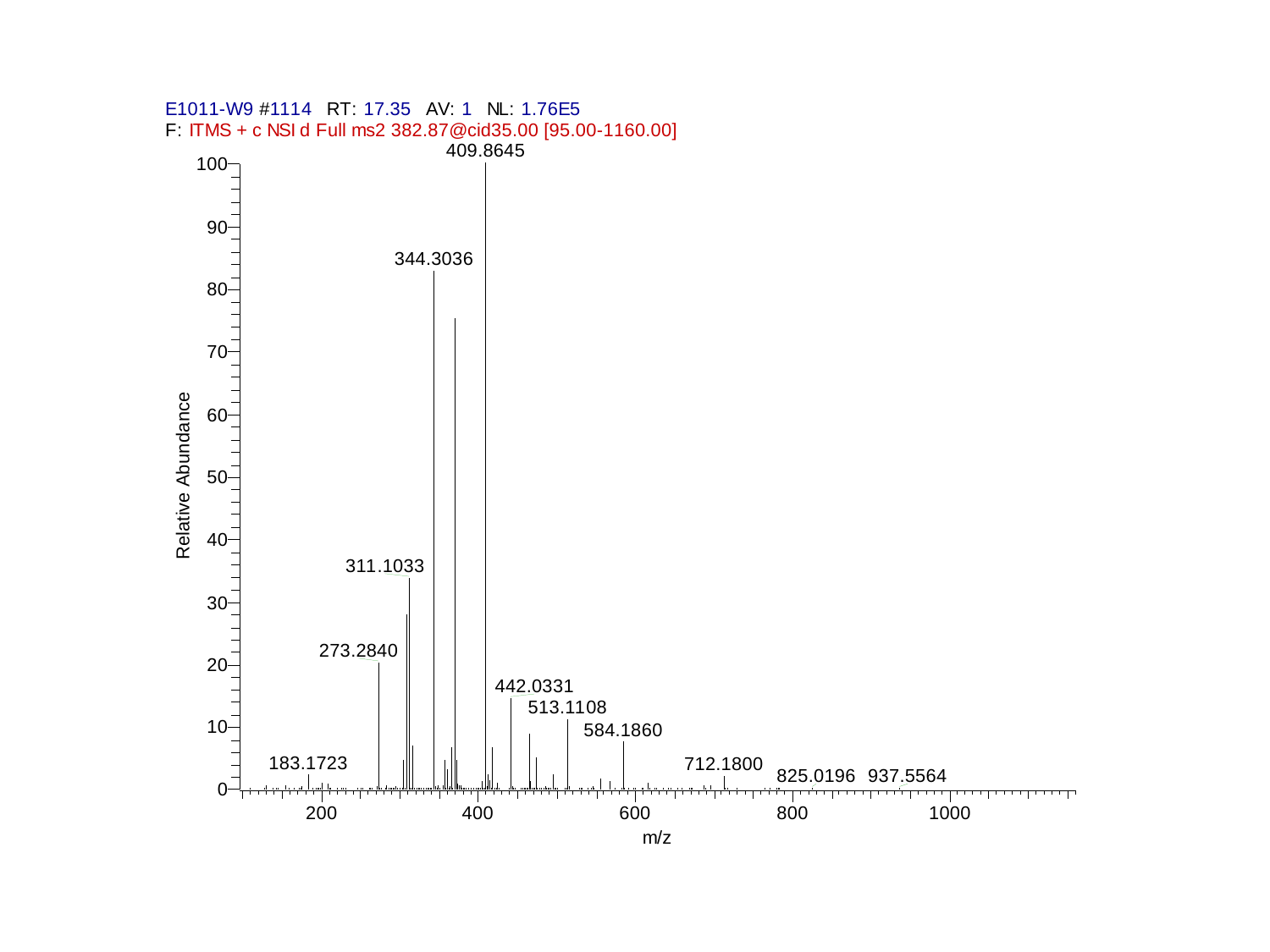

## Slide 80
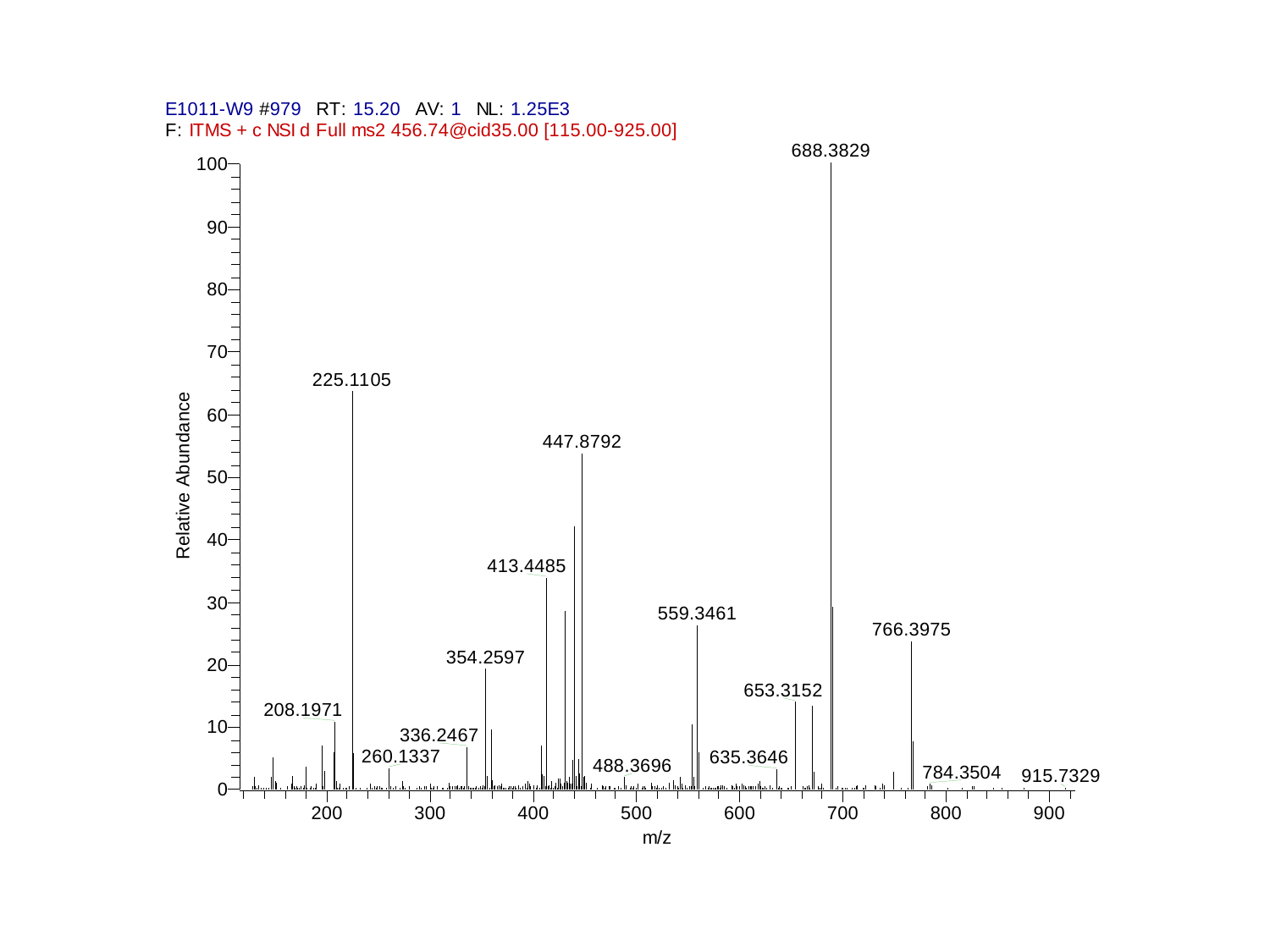

## Slide 81
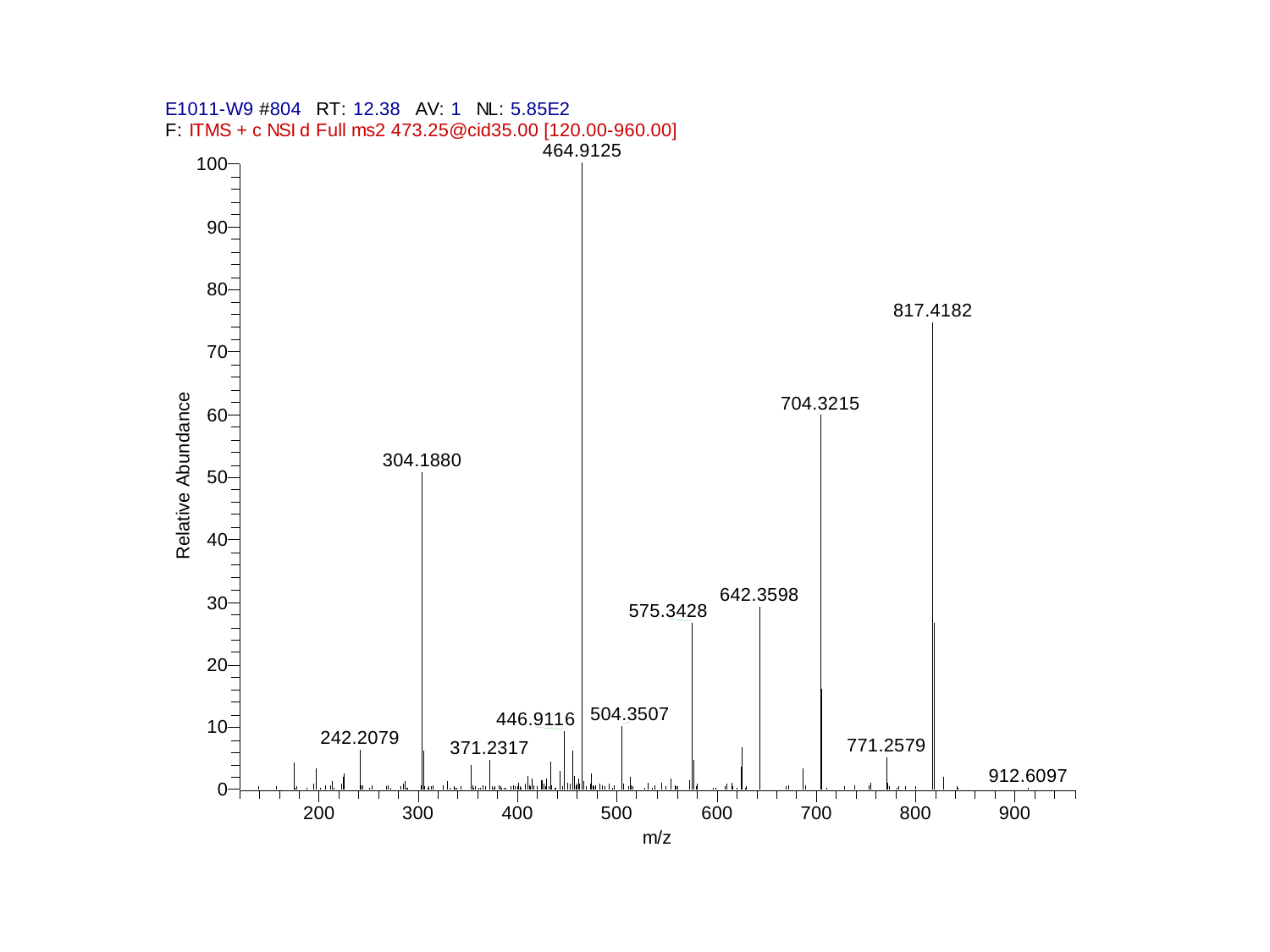

## Slide 82
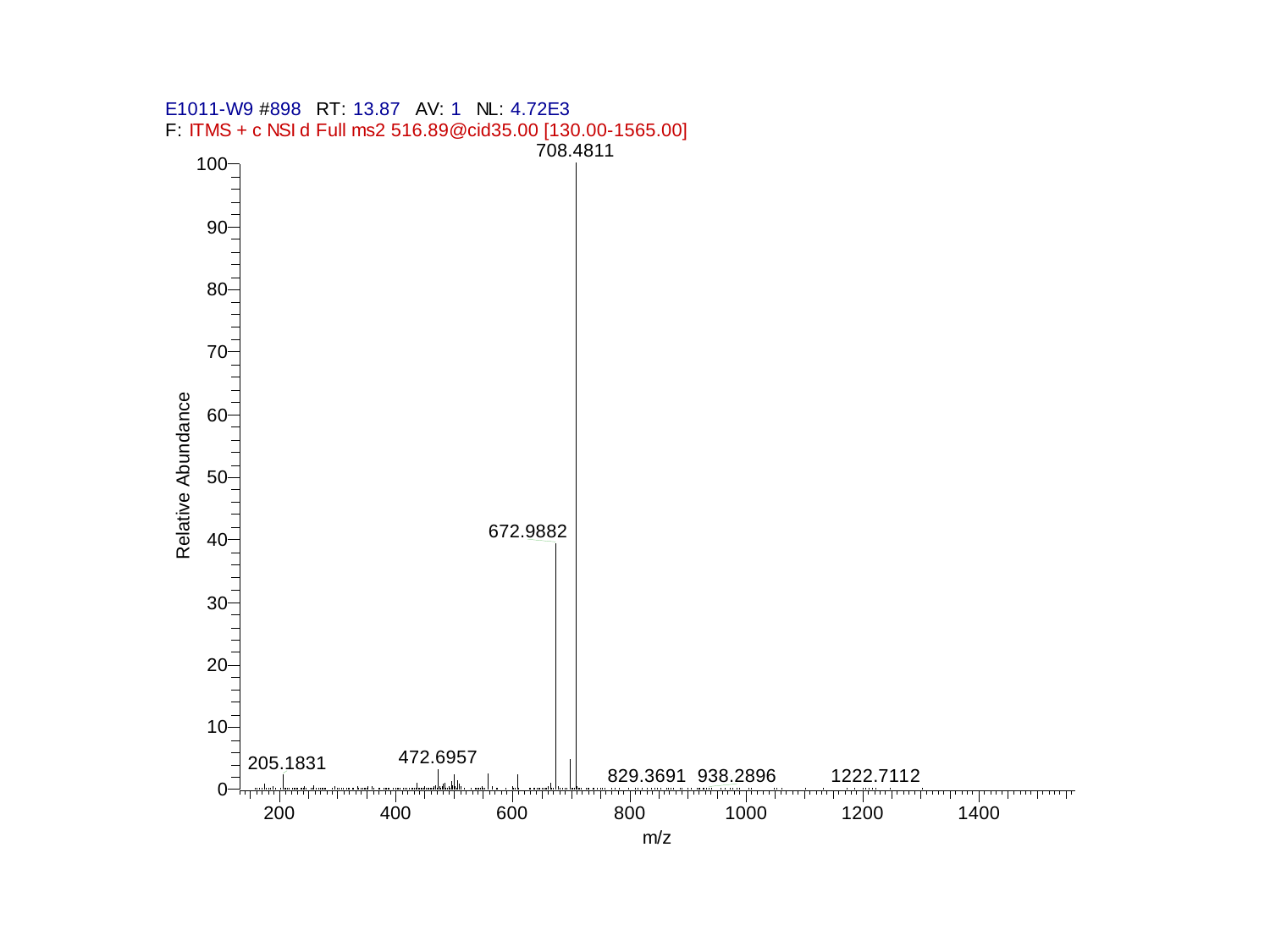

## Slide 83
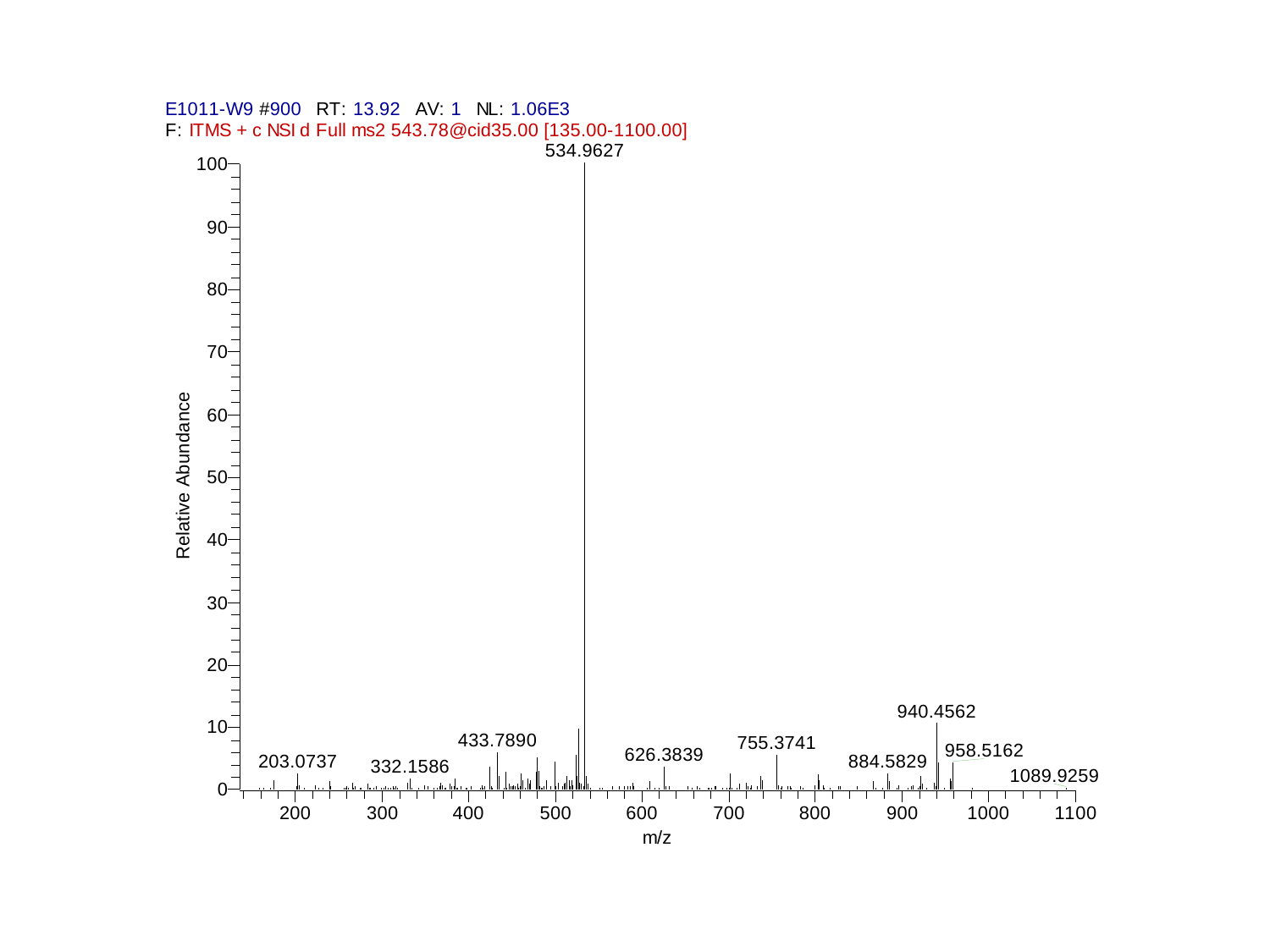

## Slide 84
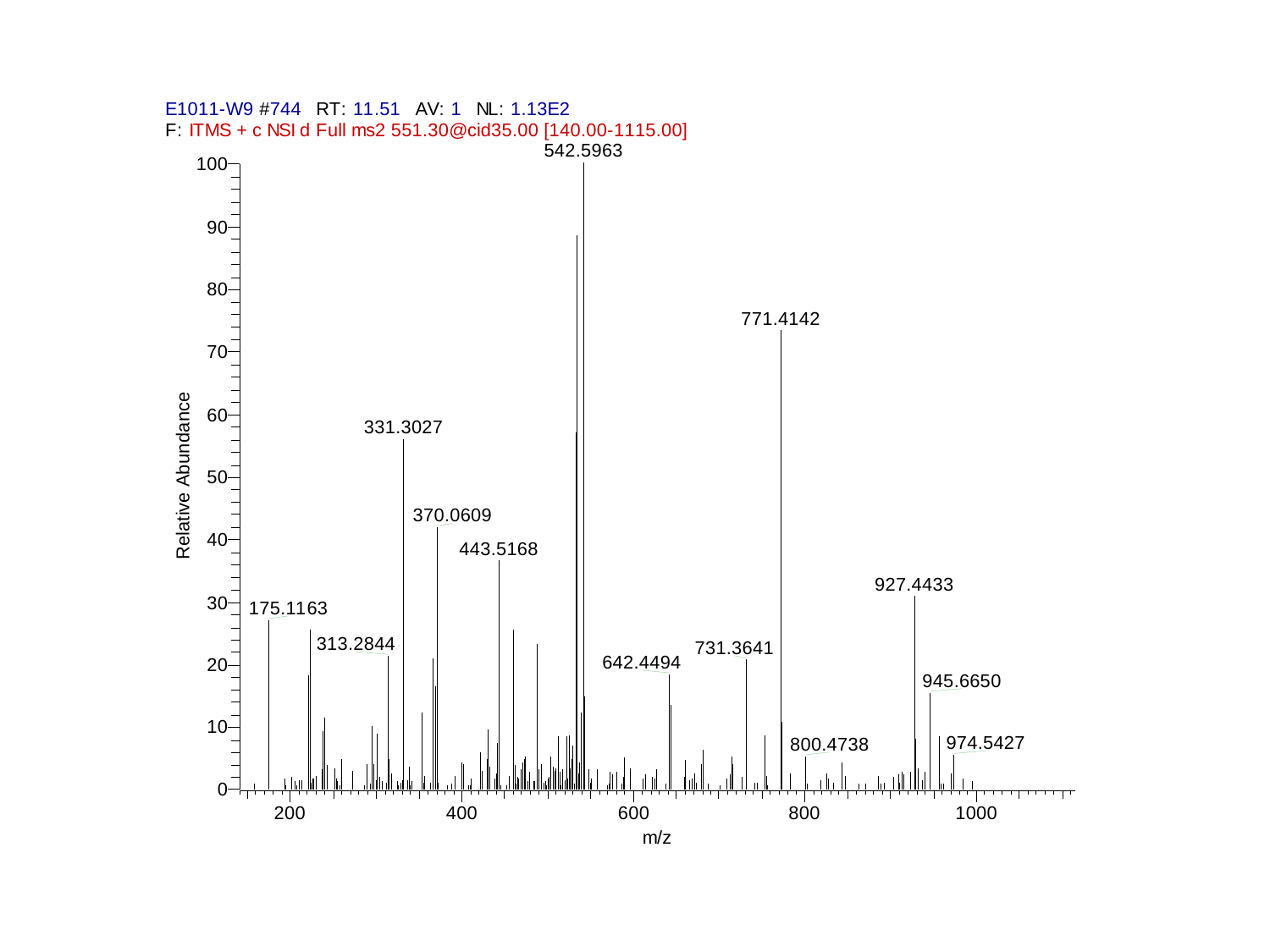

## Slide 85
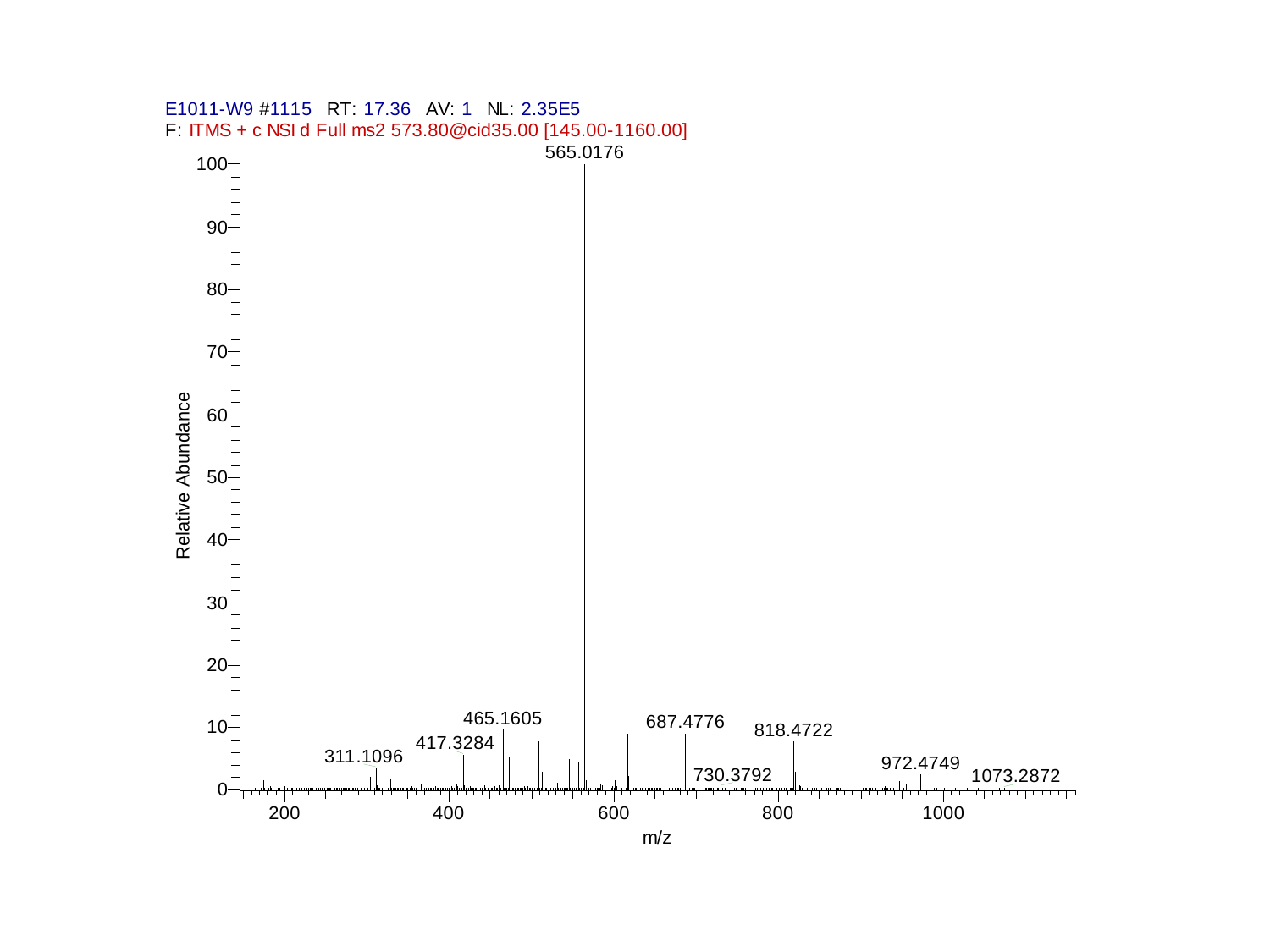

## Slide 86
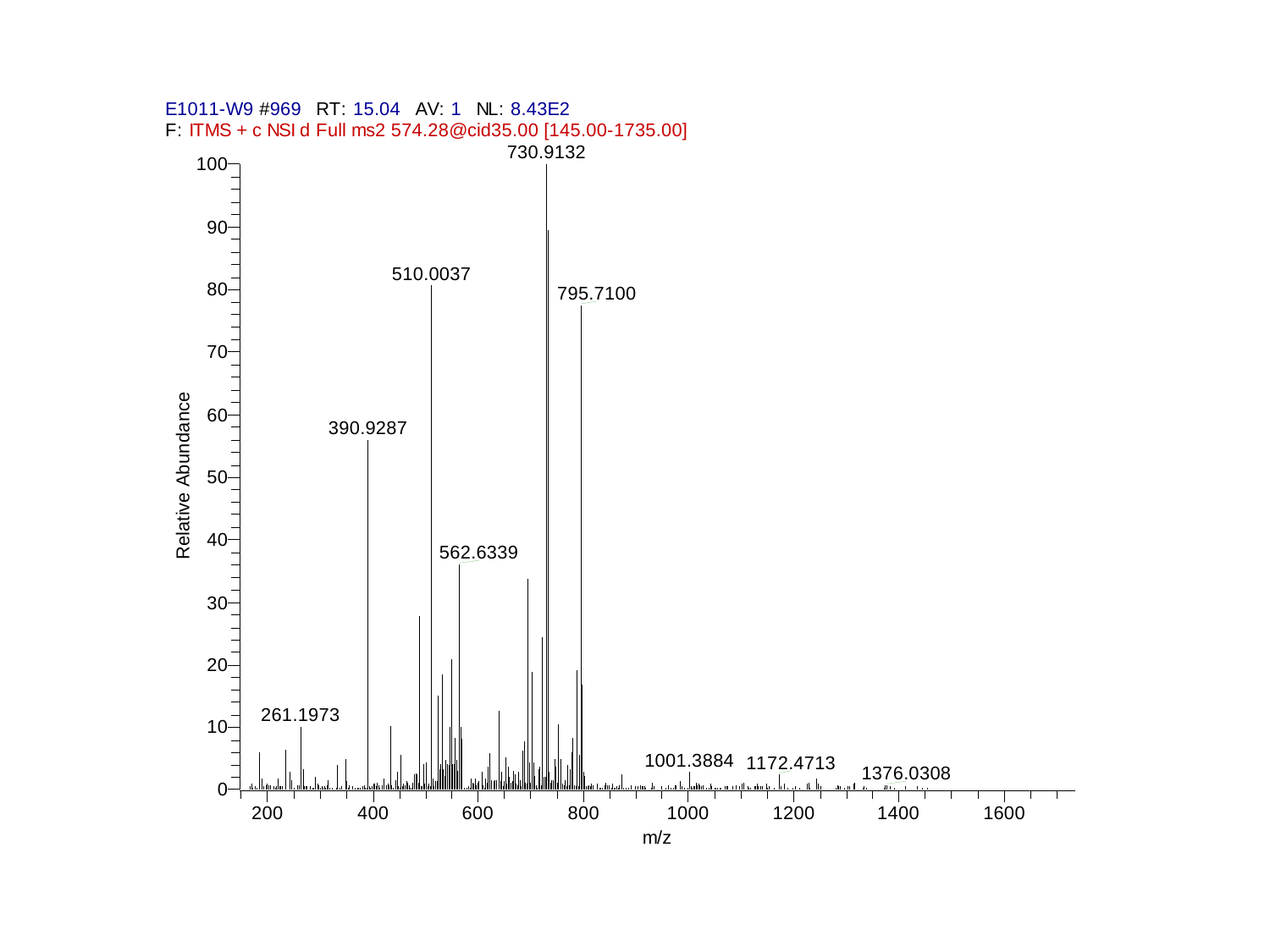

## Slide 87
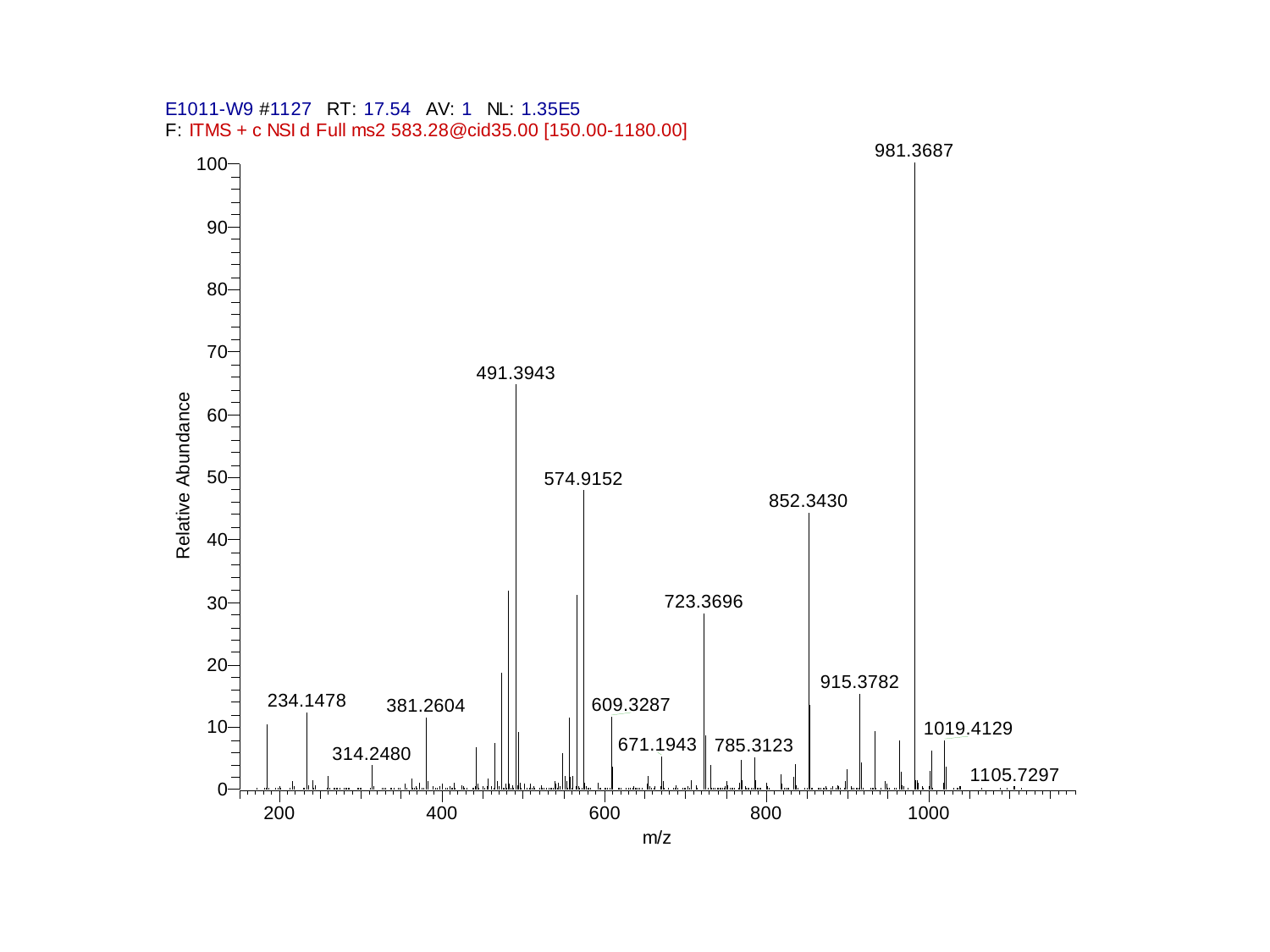

## Slide 88
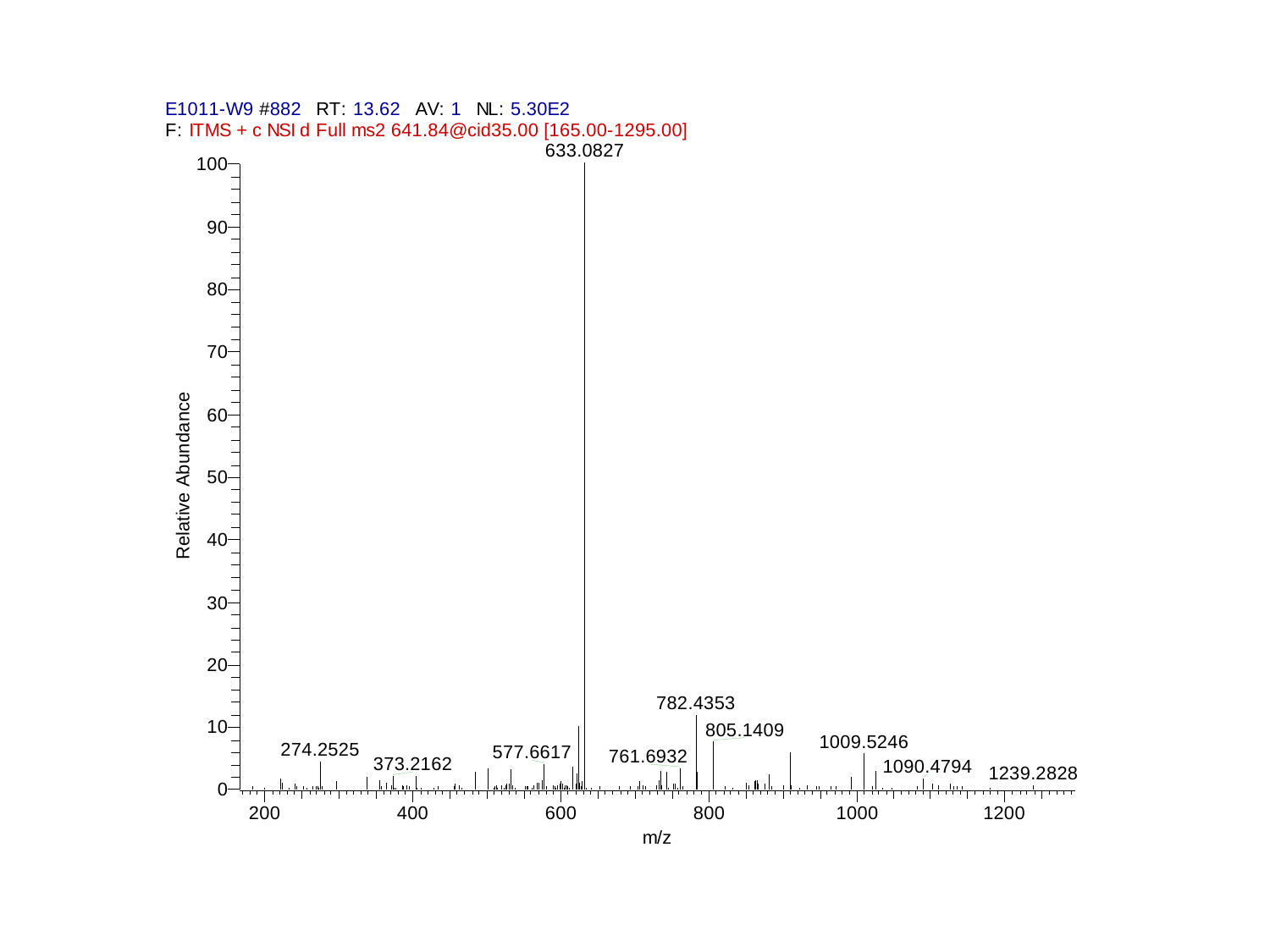

## Slide 89
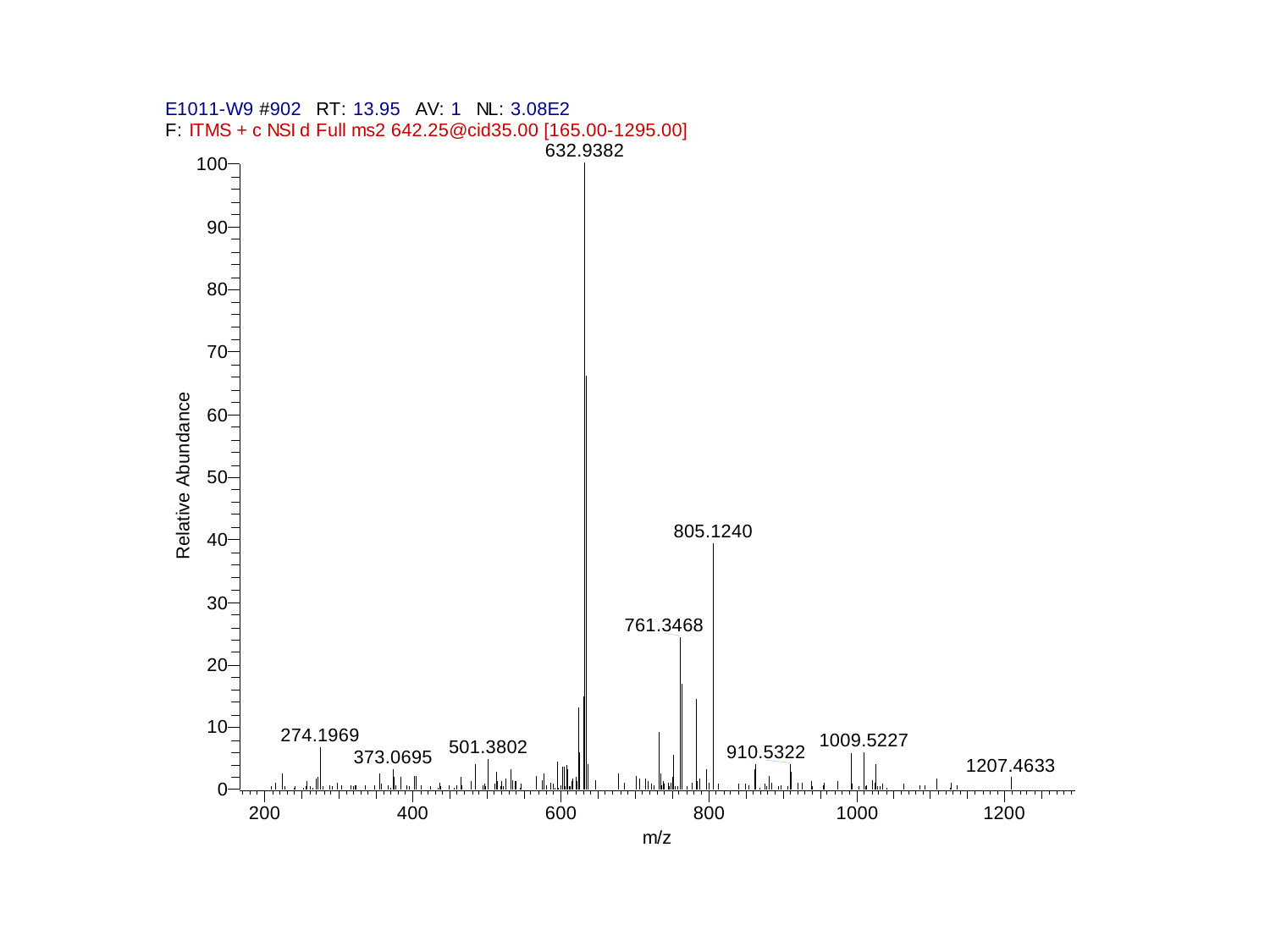

## Slide 90
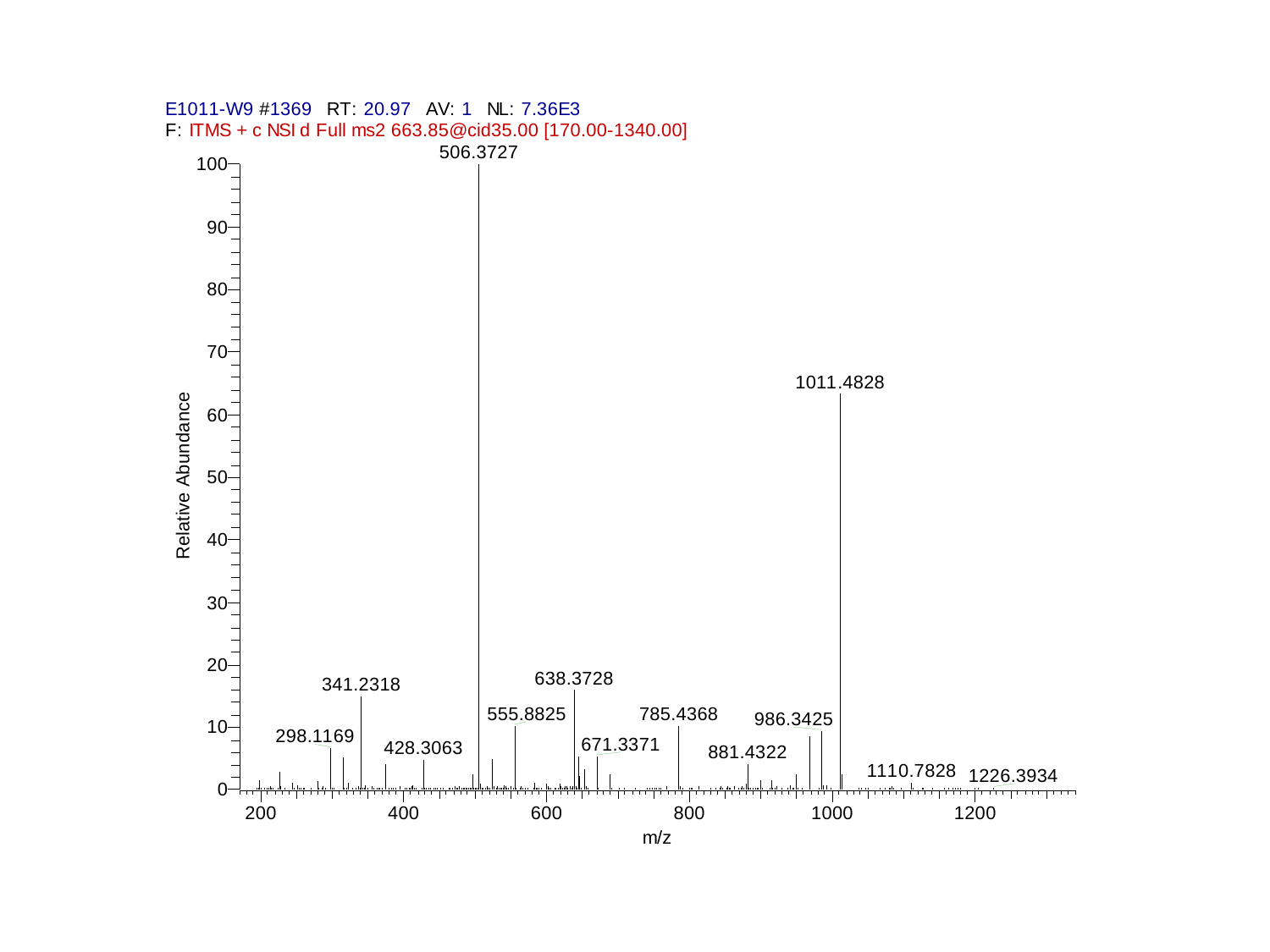

## Slide 91
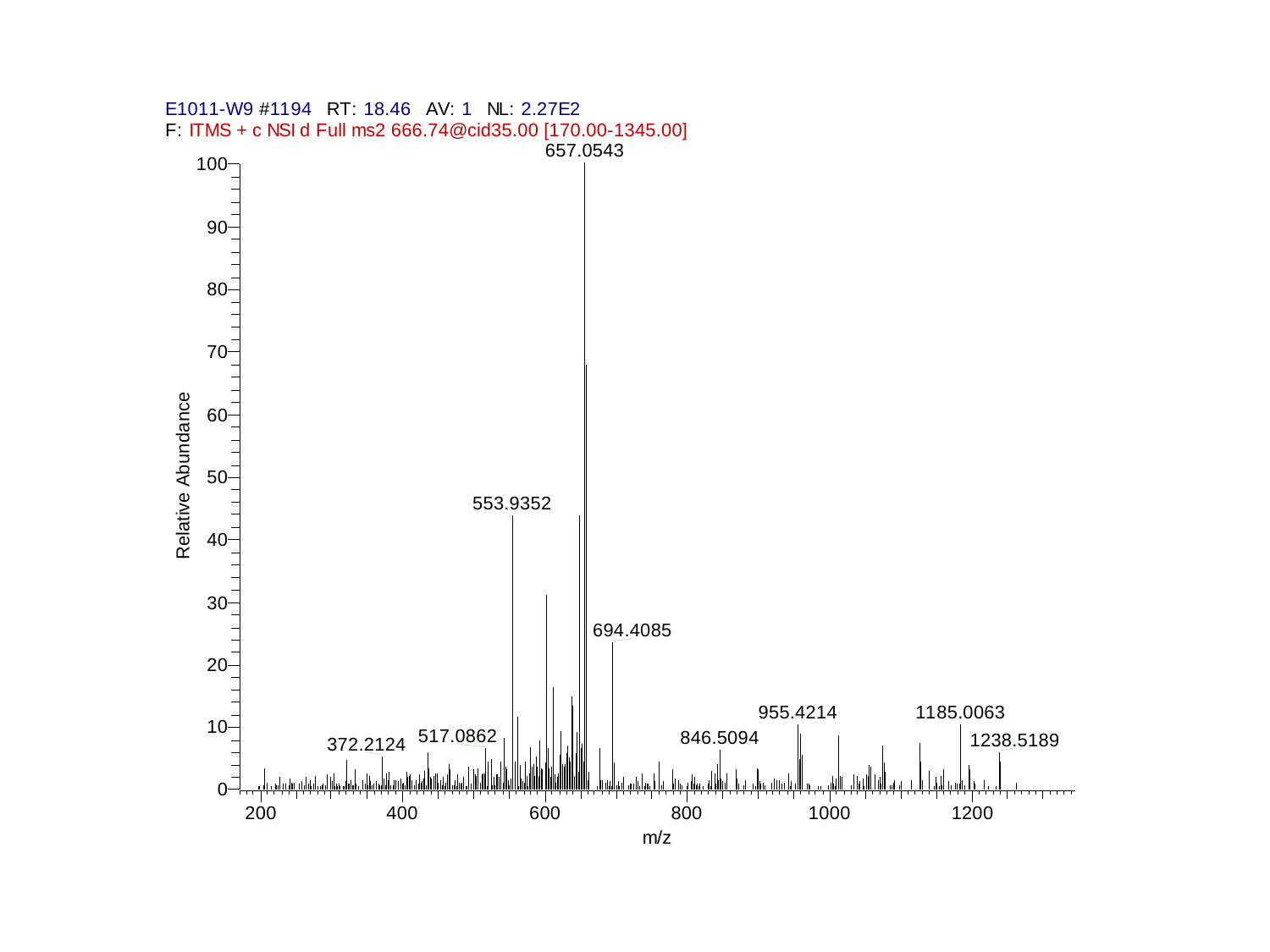

## Slide 92
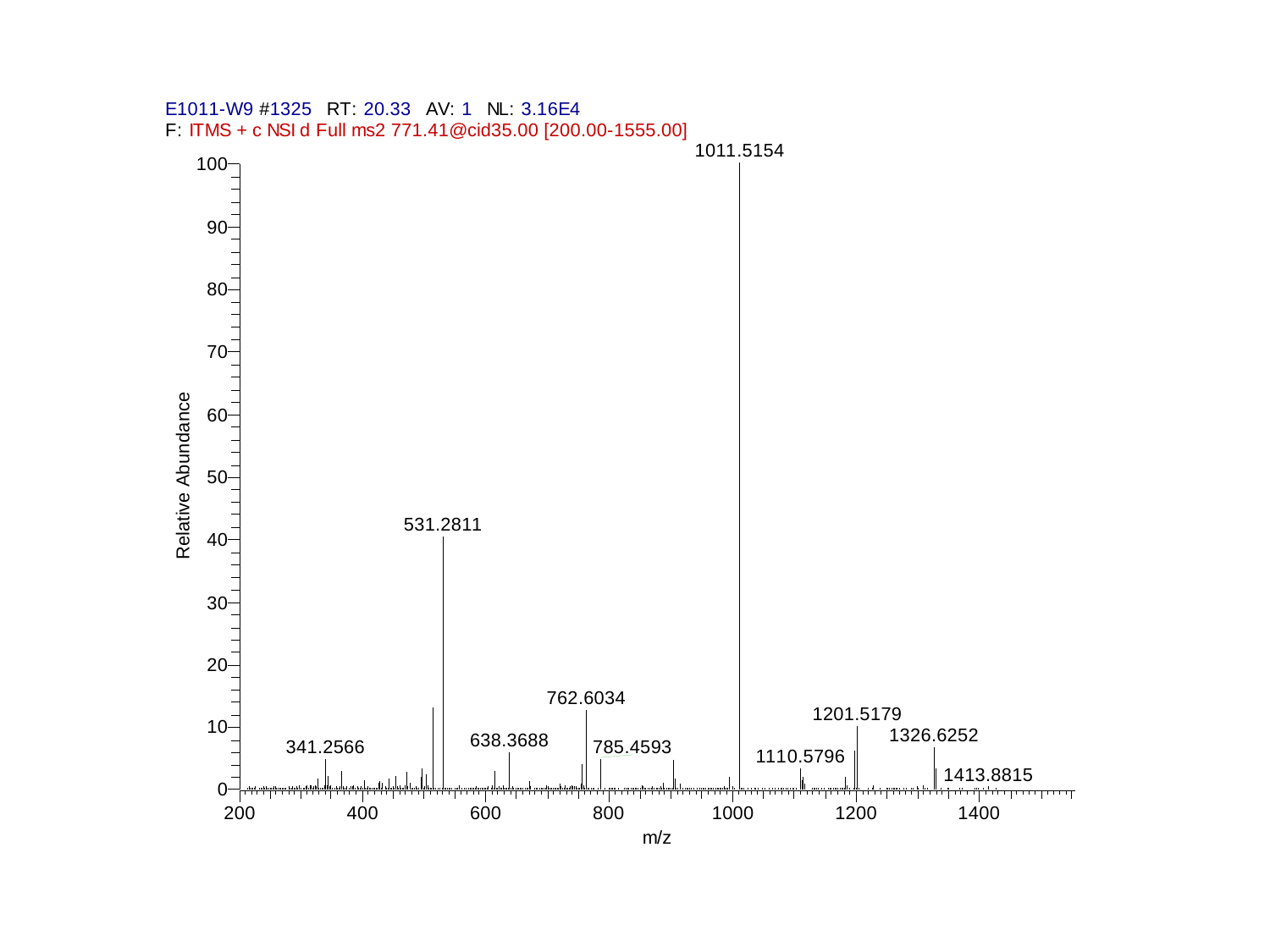

## Slide 93
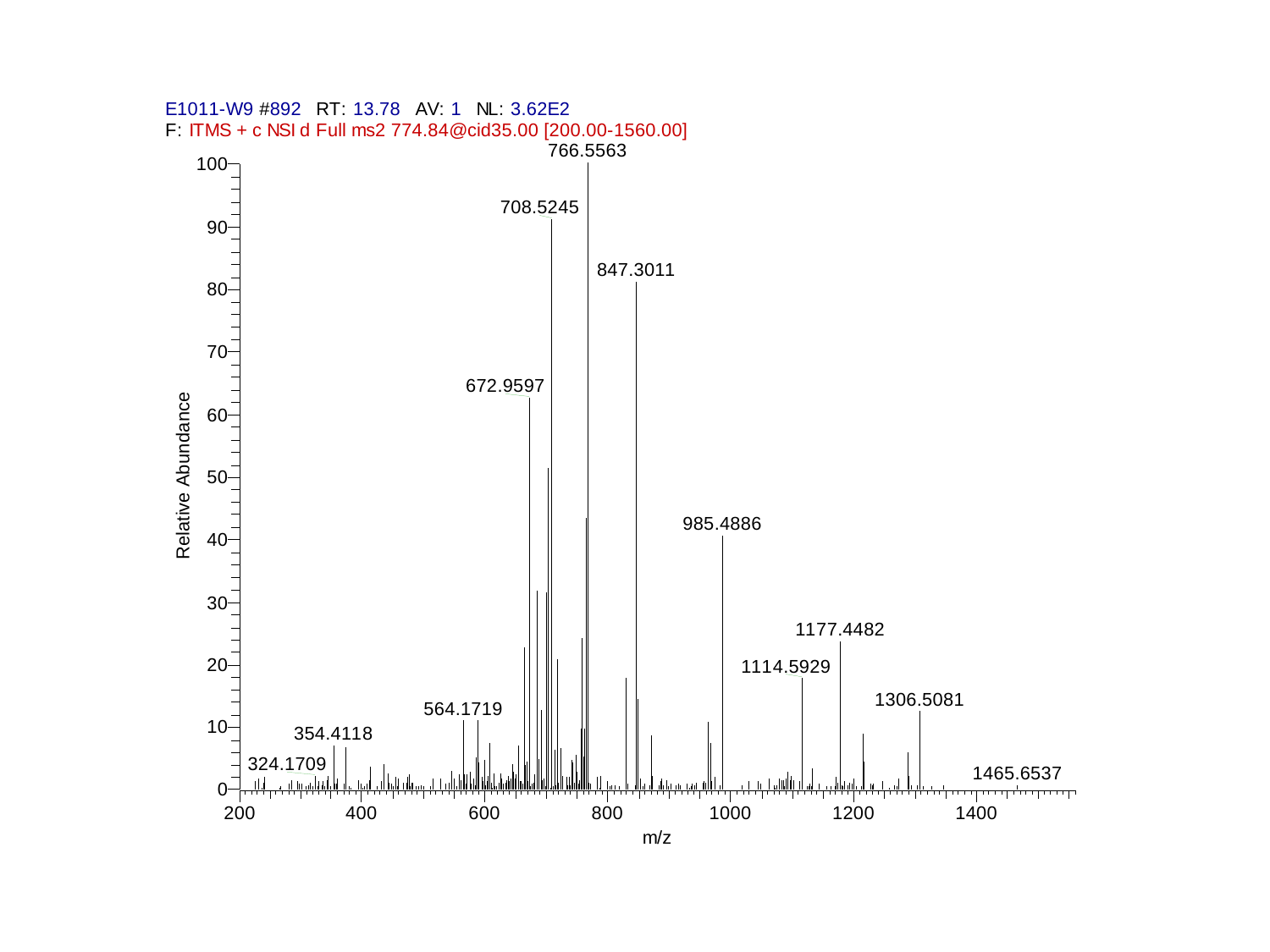

## Slide 94
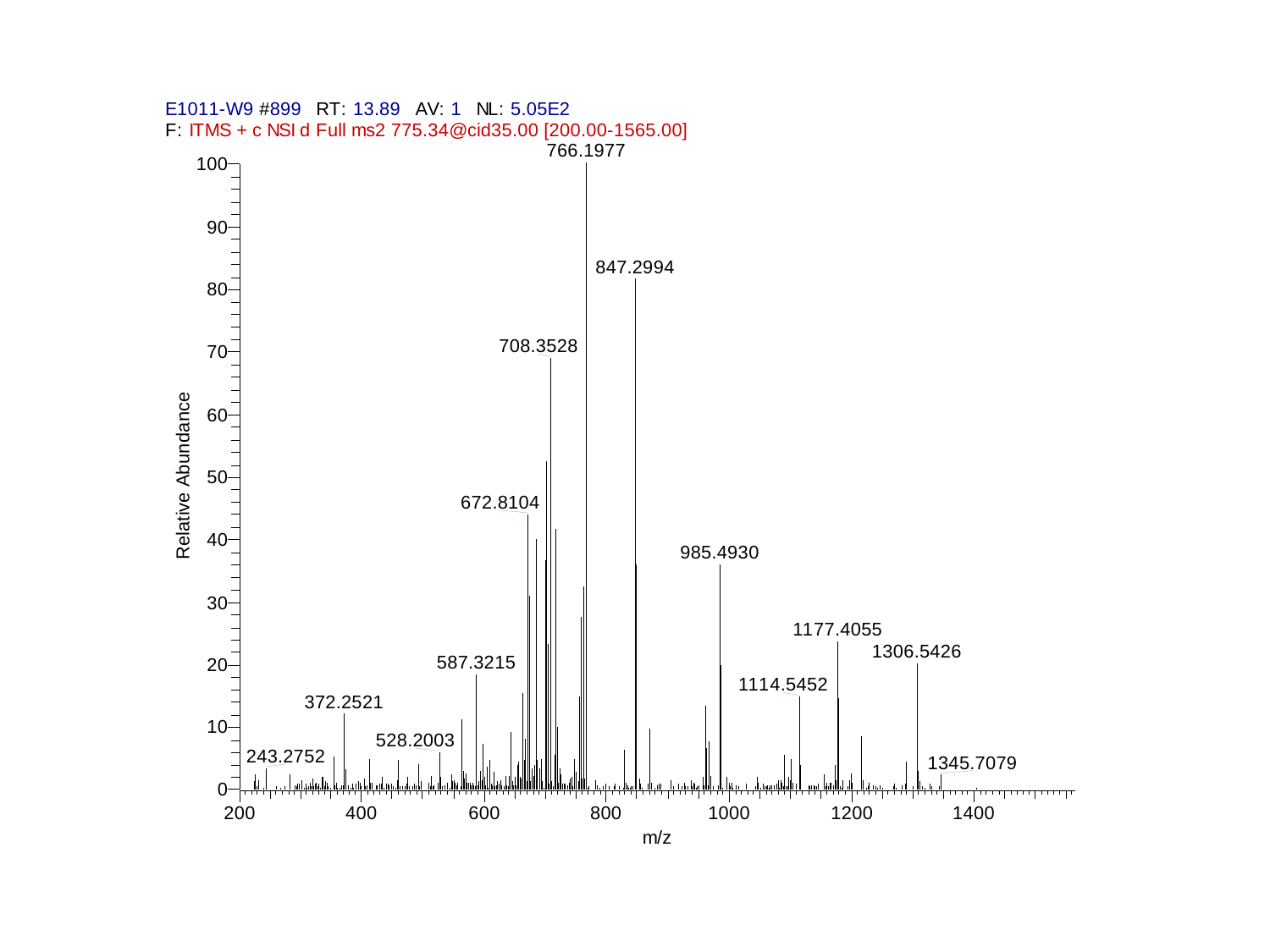

## Slide 95
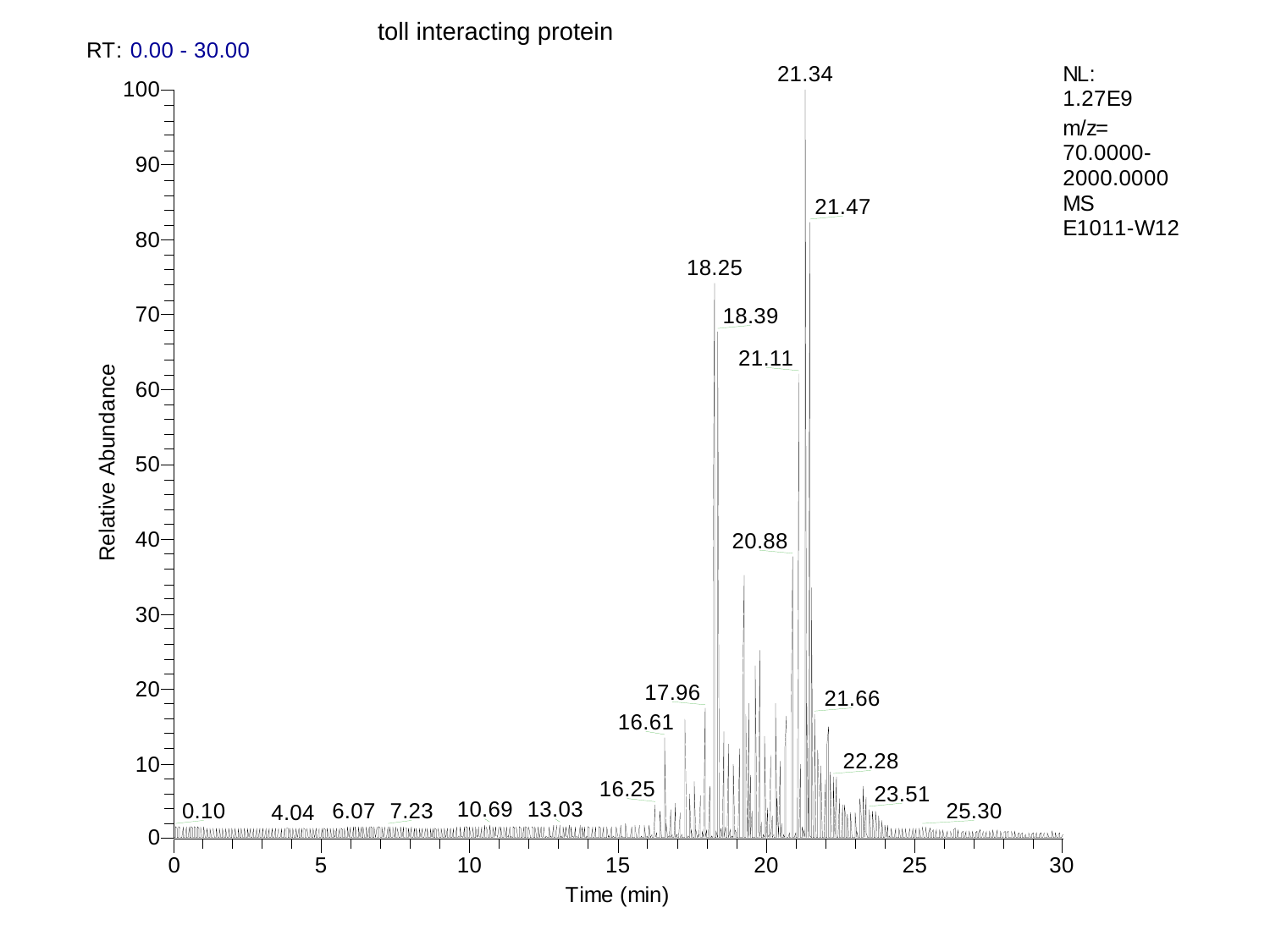

toll interacting protein

## Slide 96
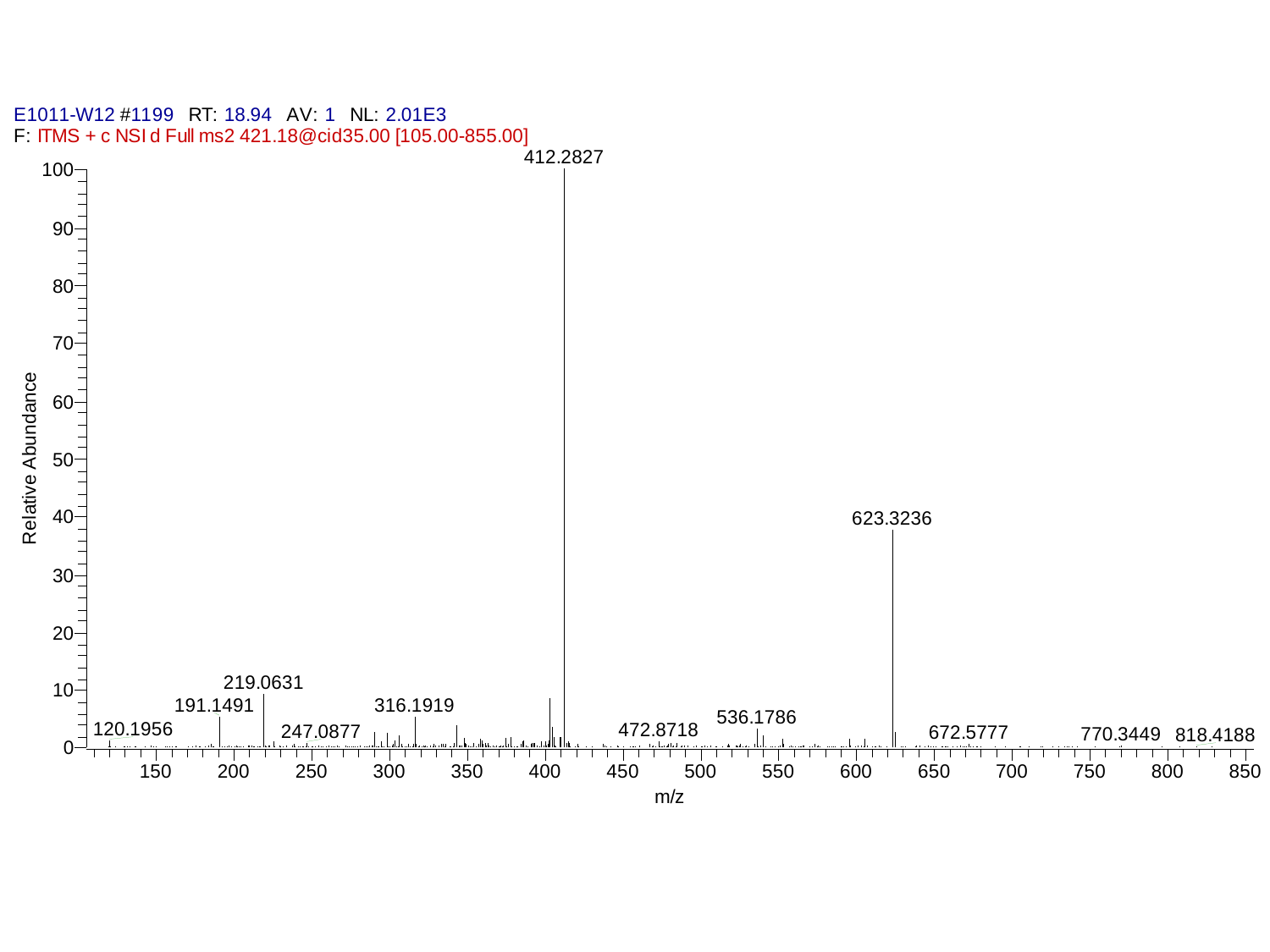

## Slide 97
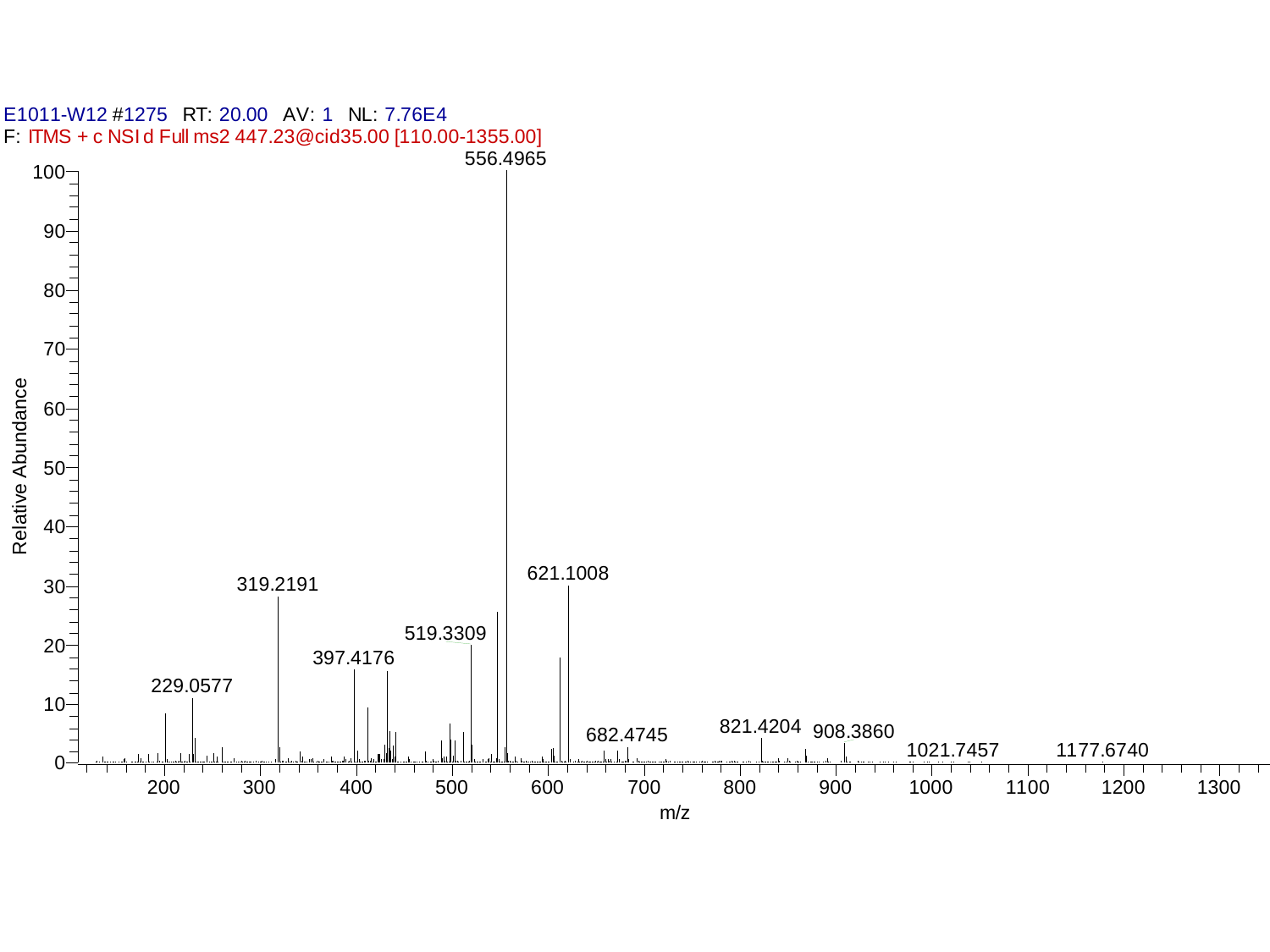

## Slide 98
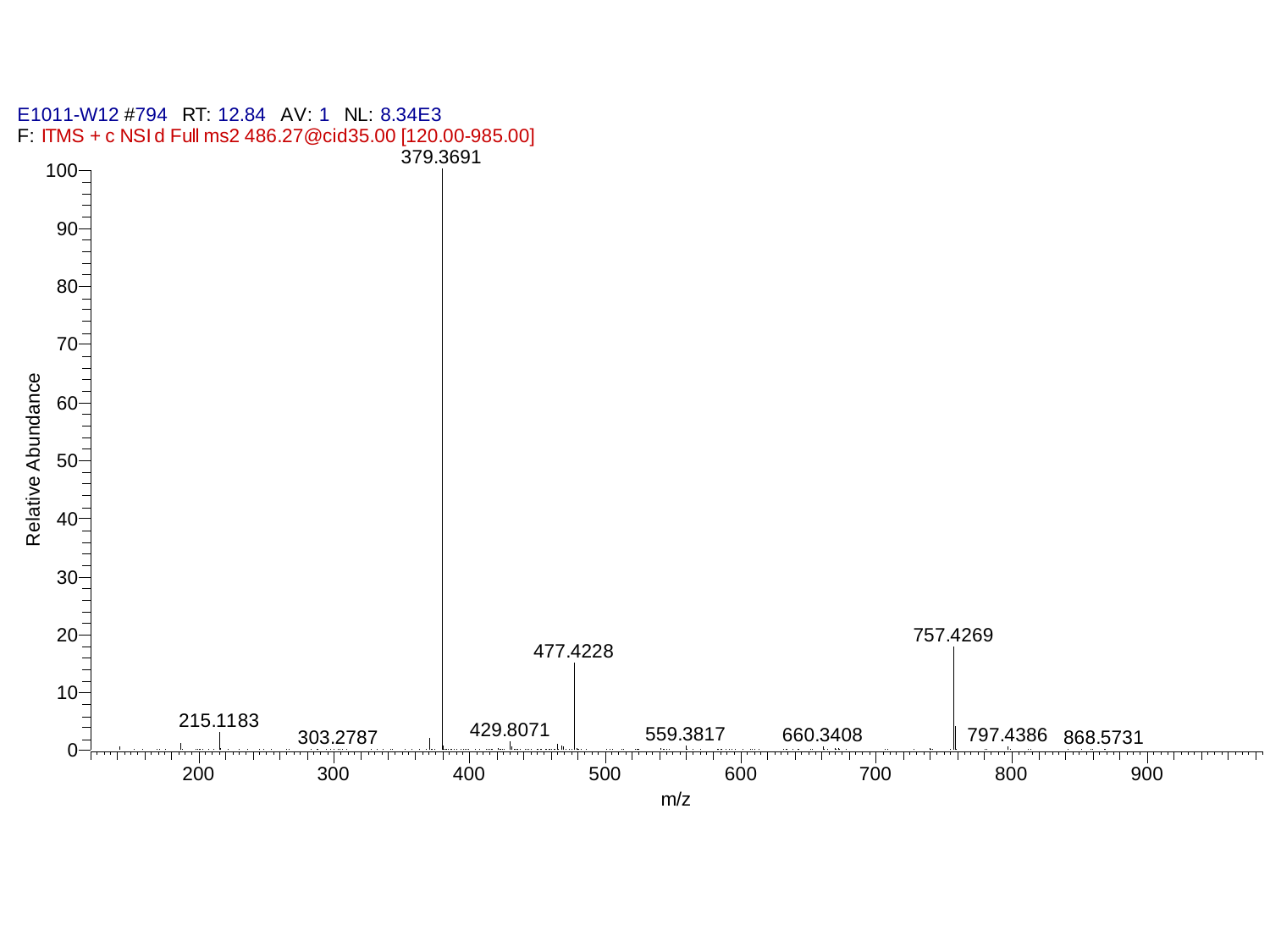

## Slide 99
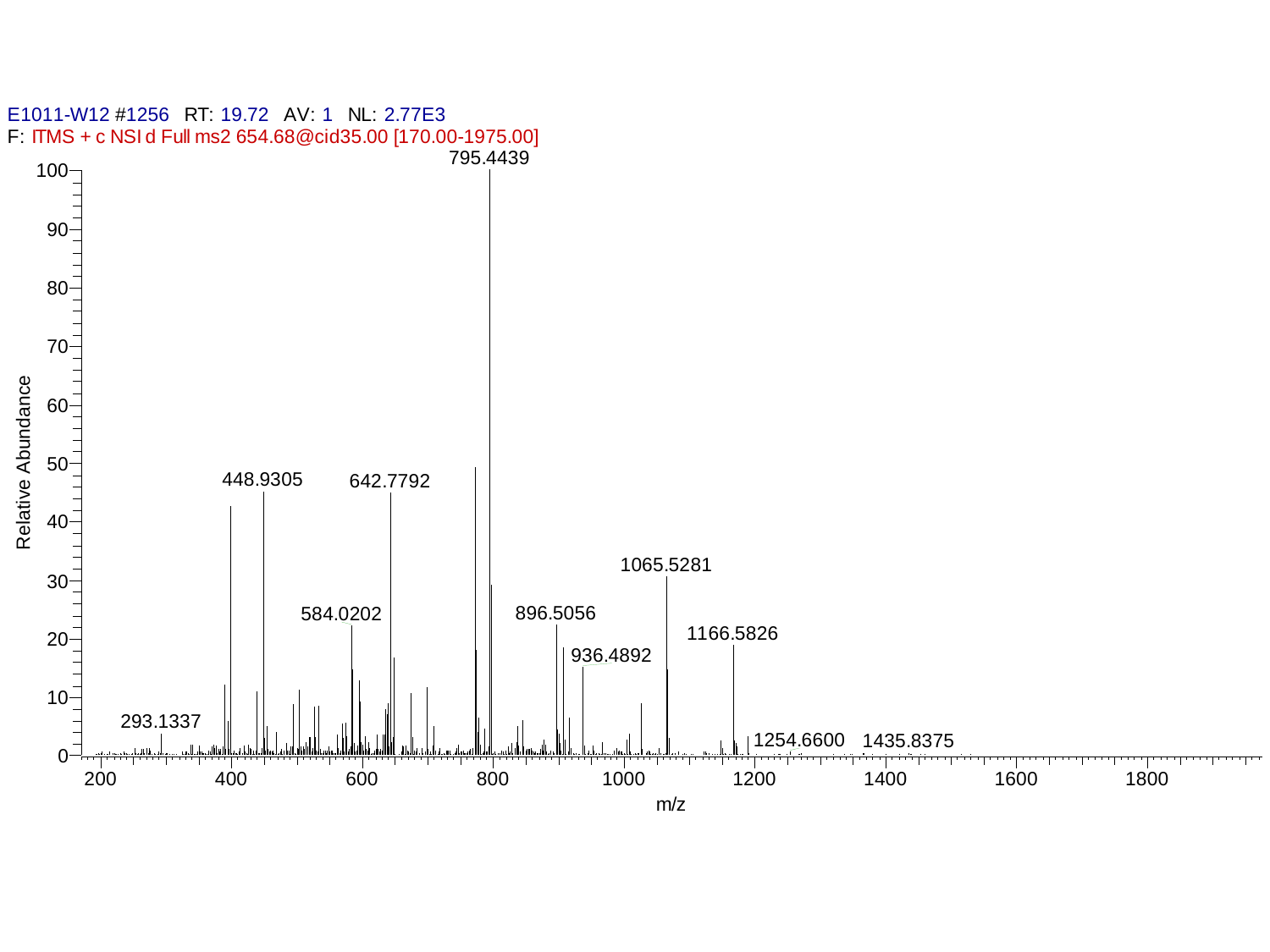

## Slide 100
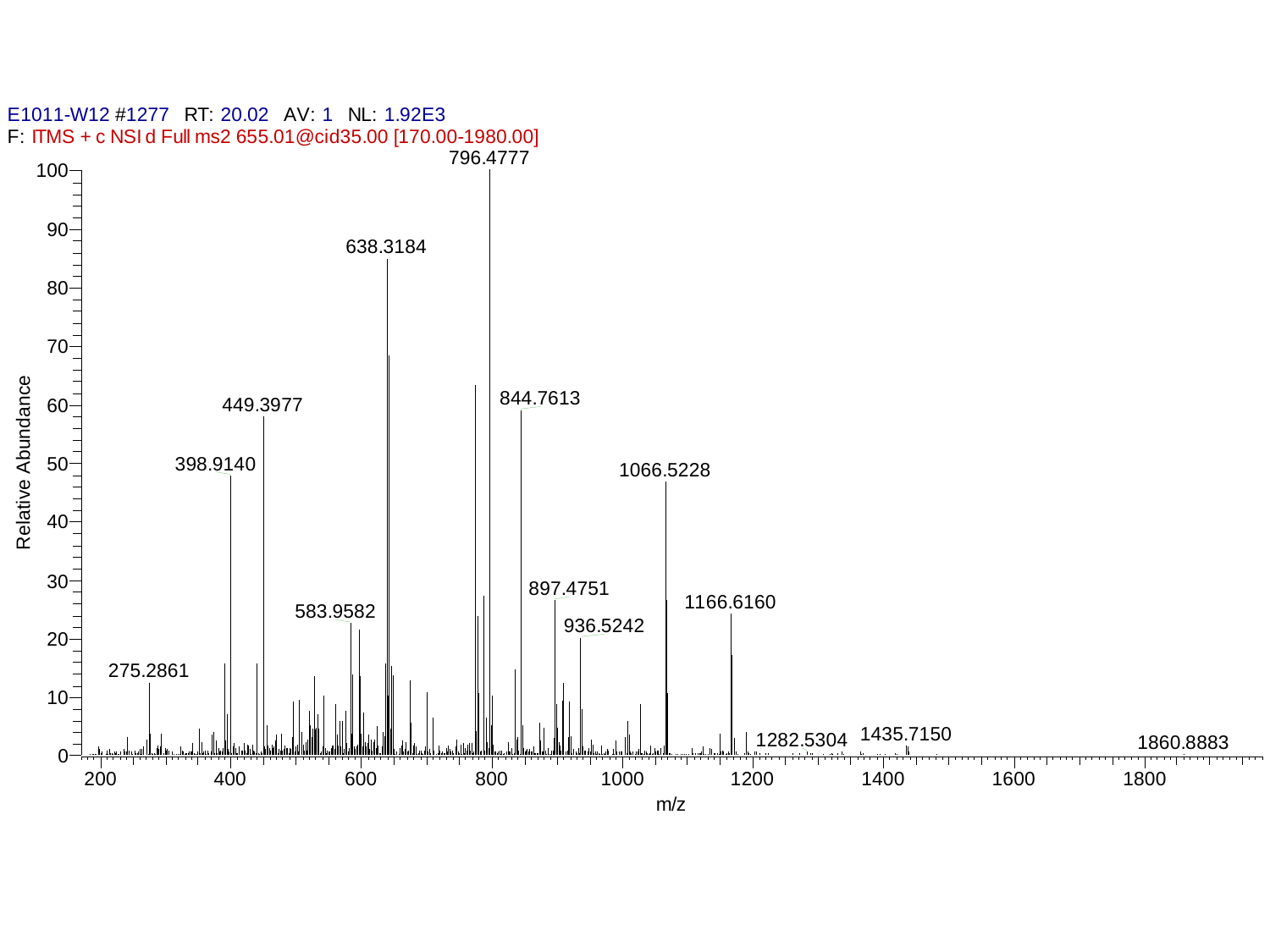

## Slide 101
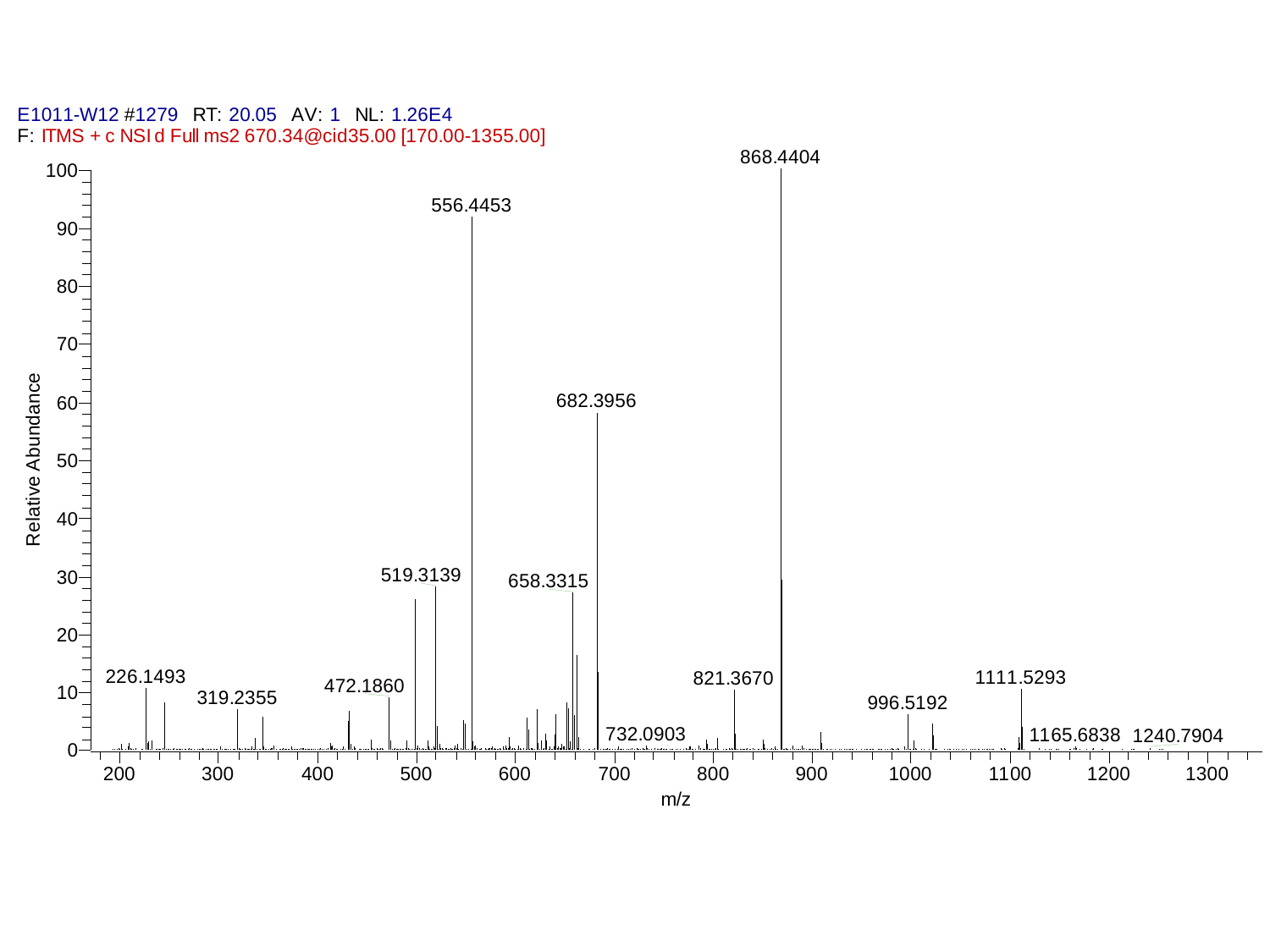

## Slide 102
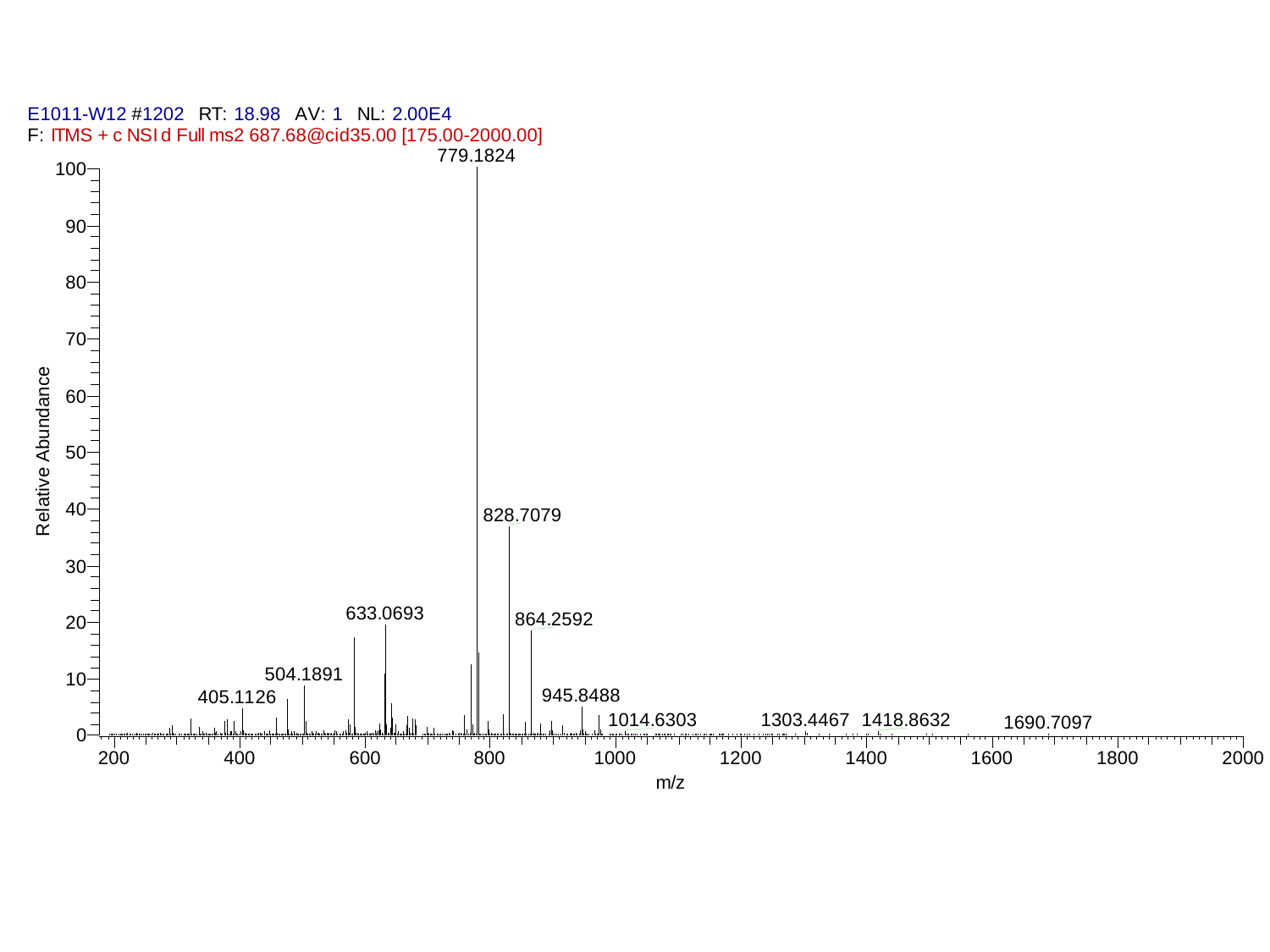

## Slide 103
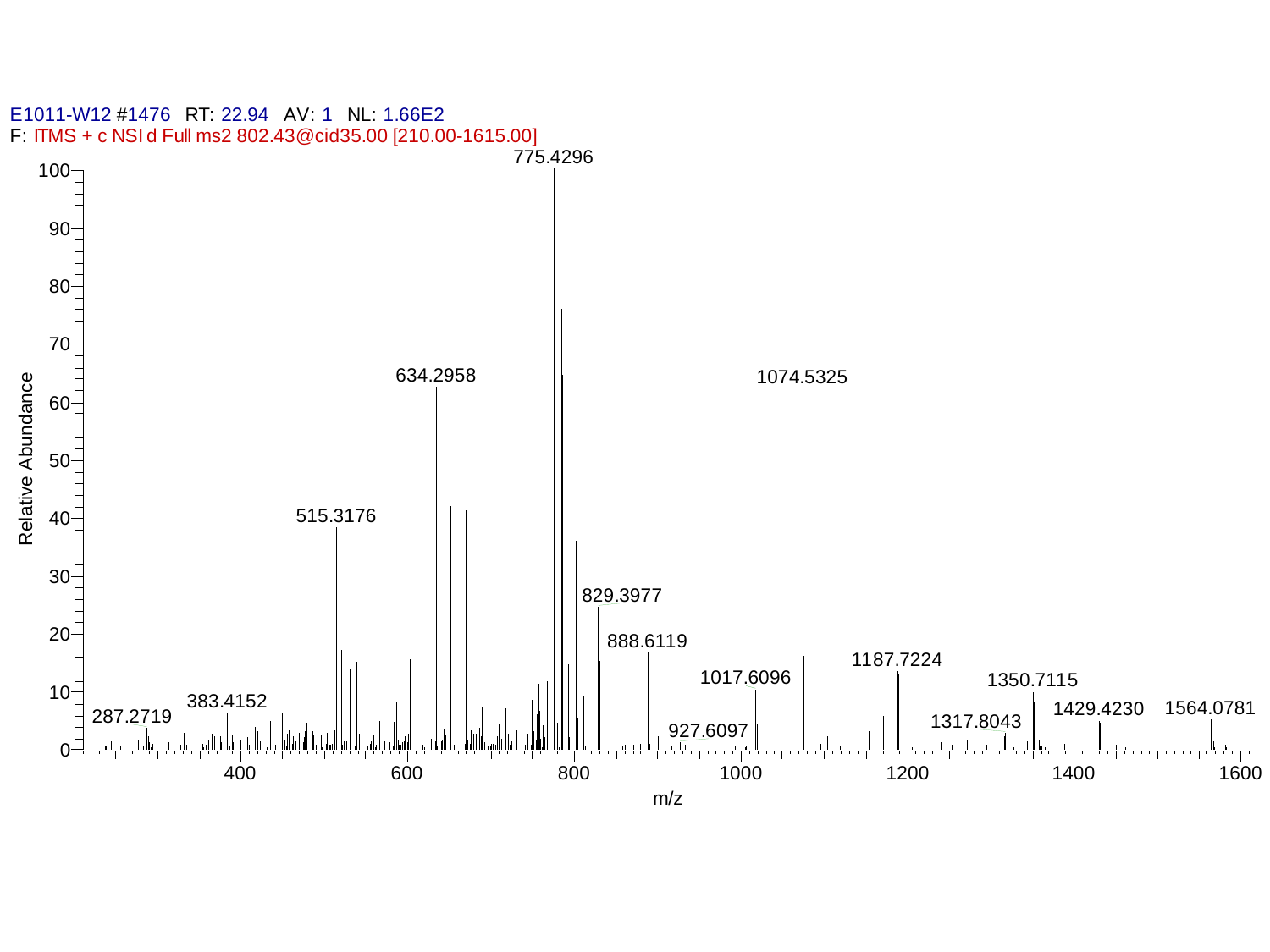

## Slide 104
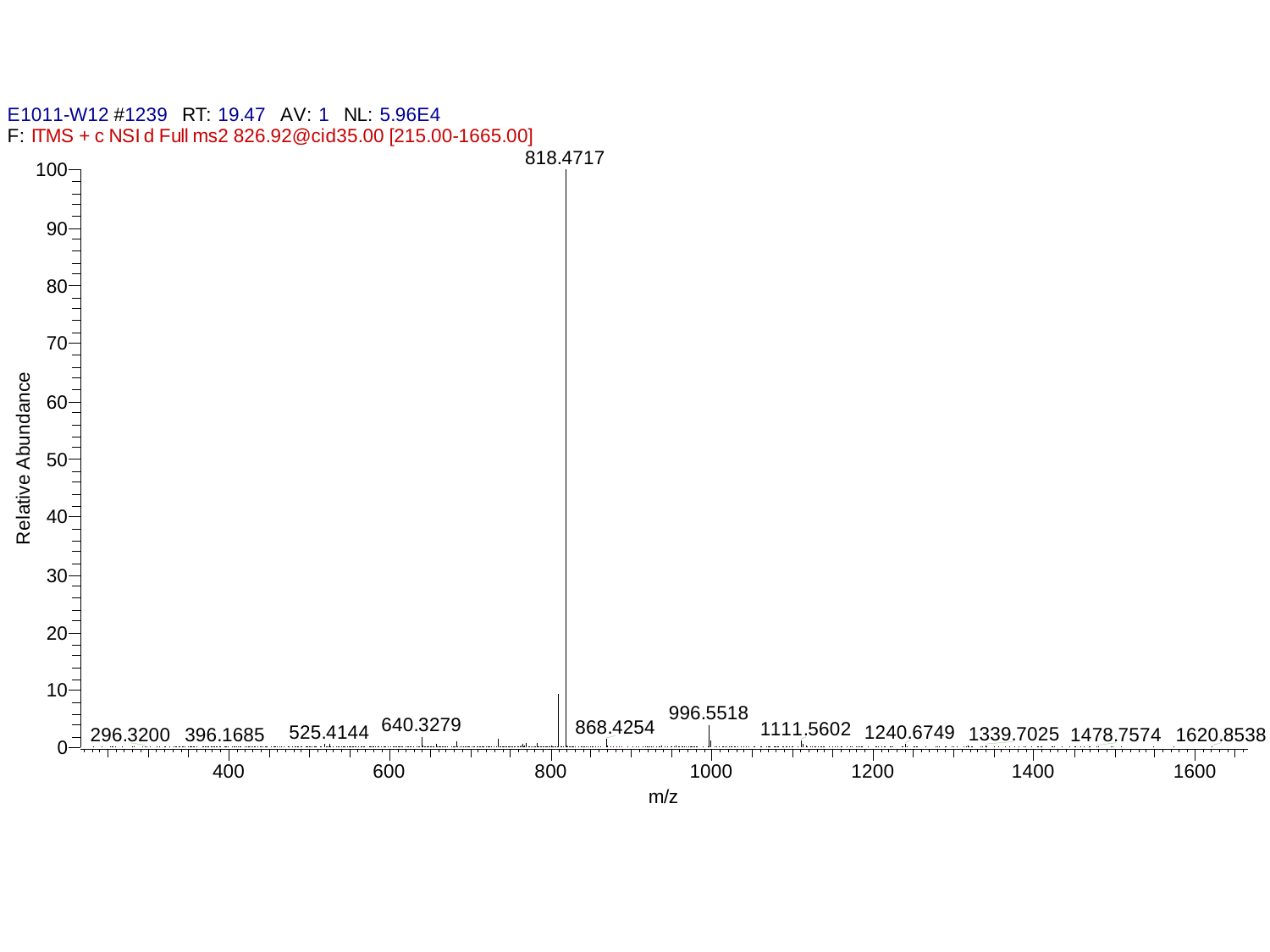

## Slide 105
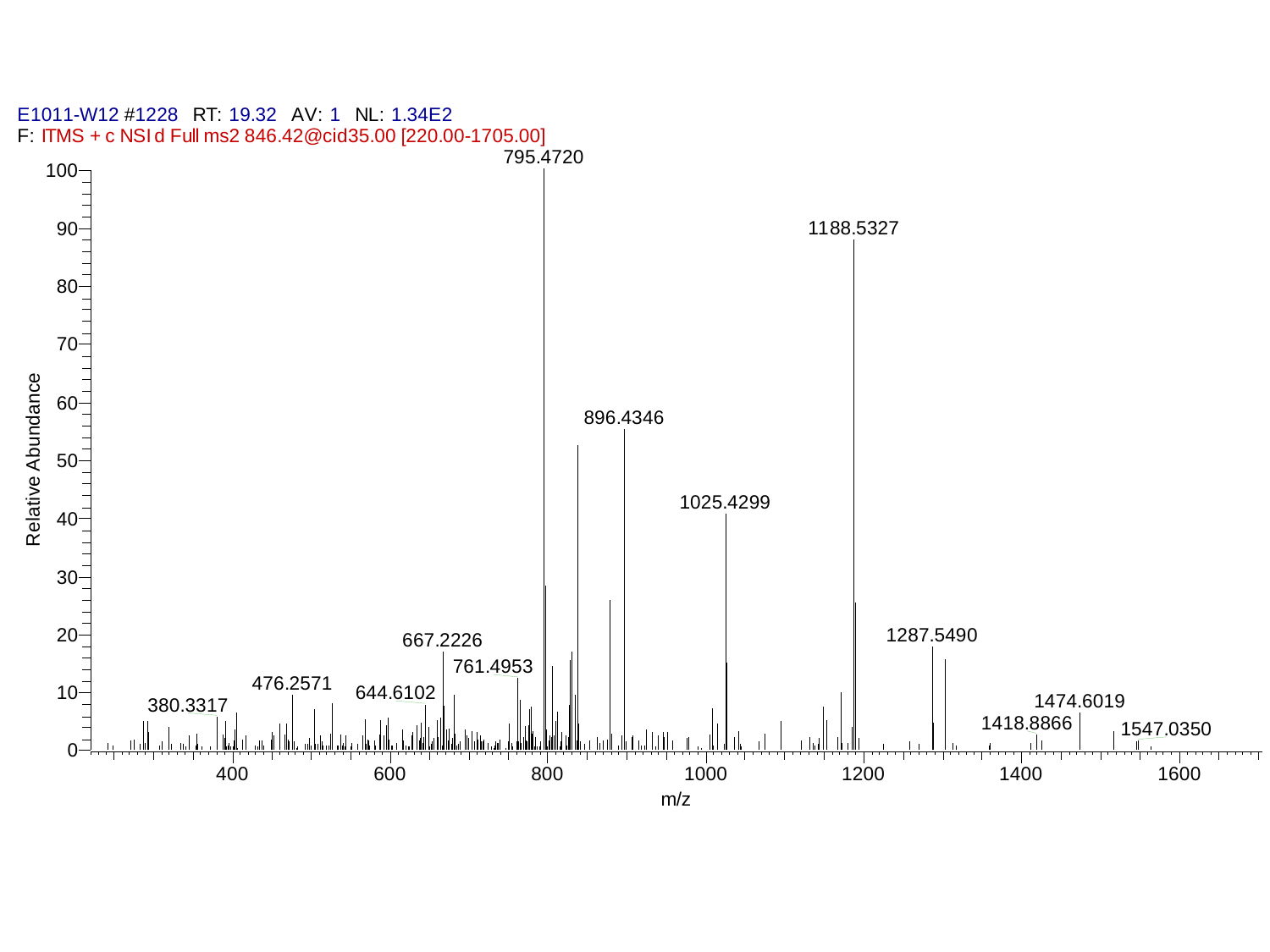

## Slide 106
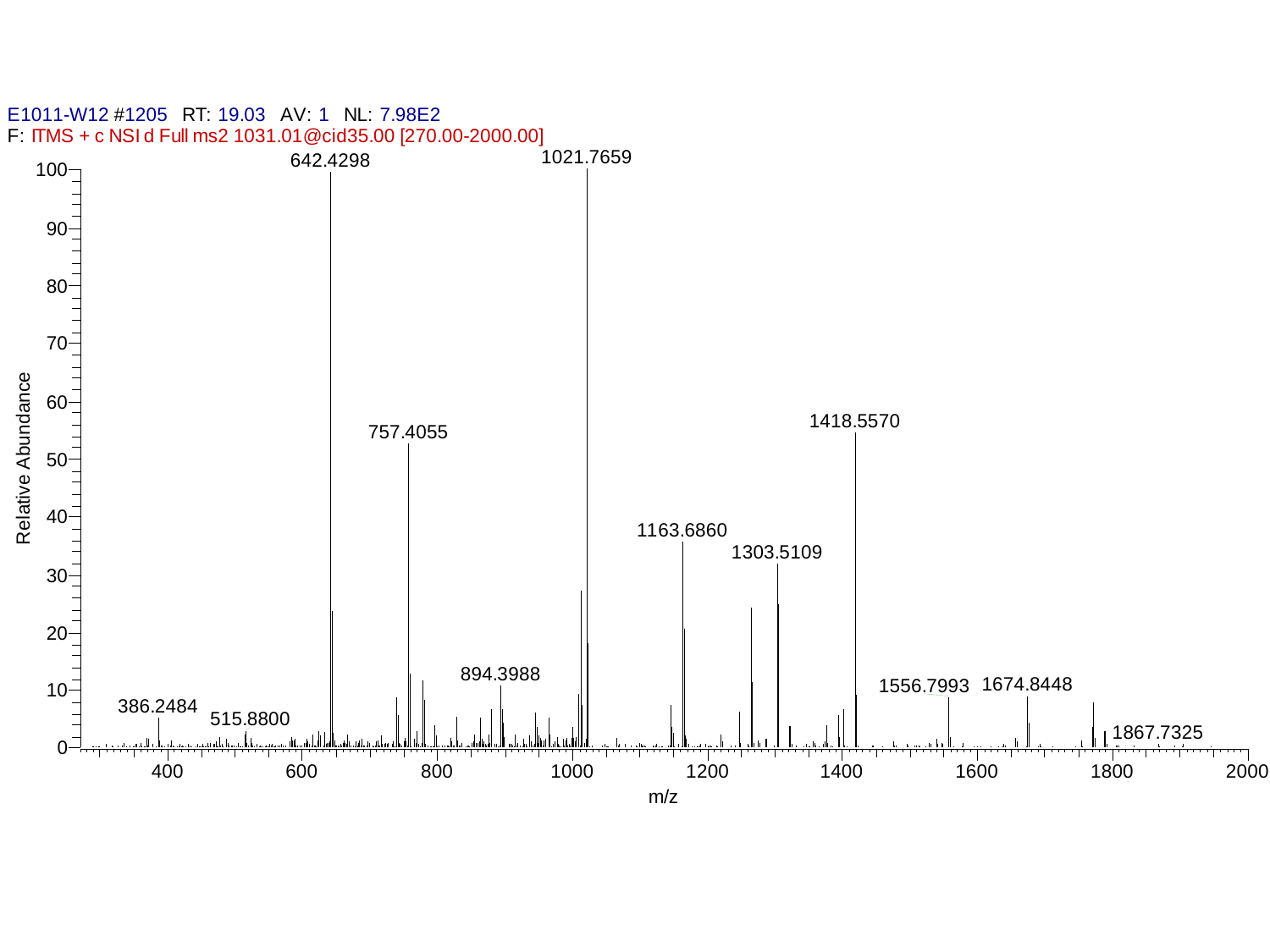

## Slide 107
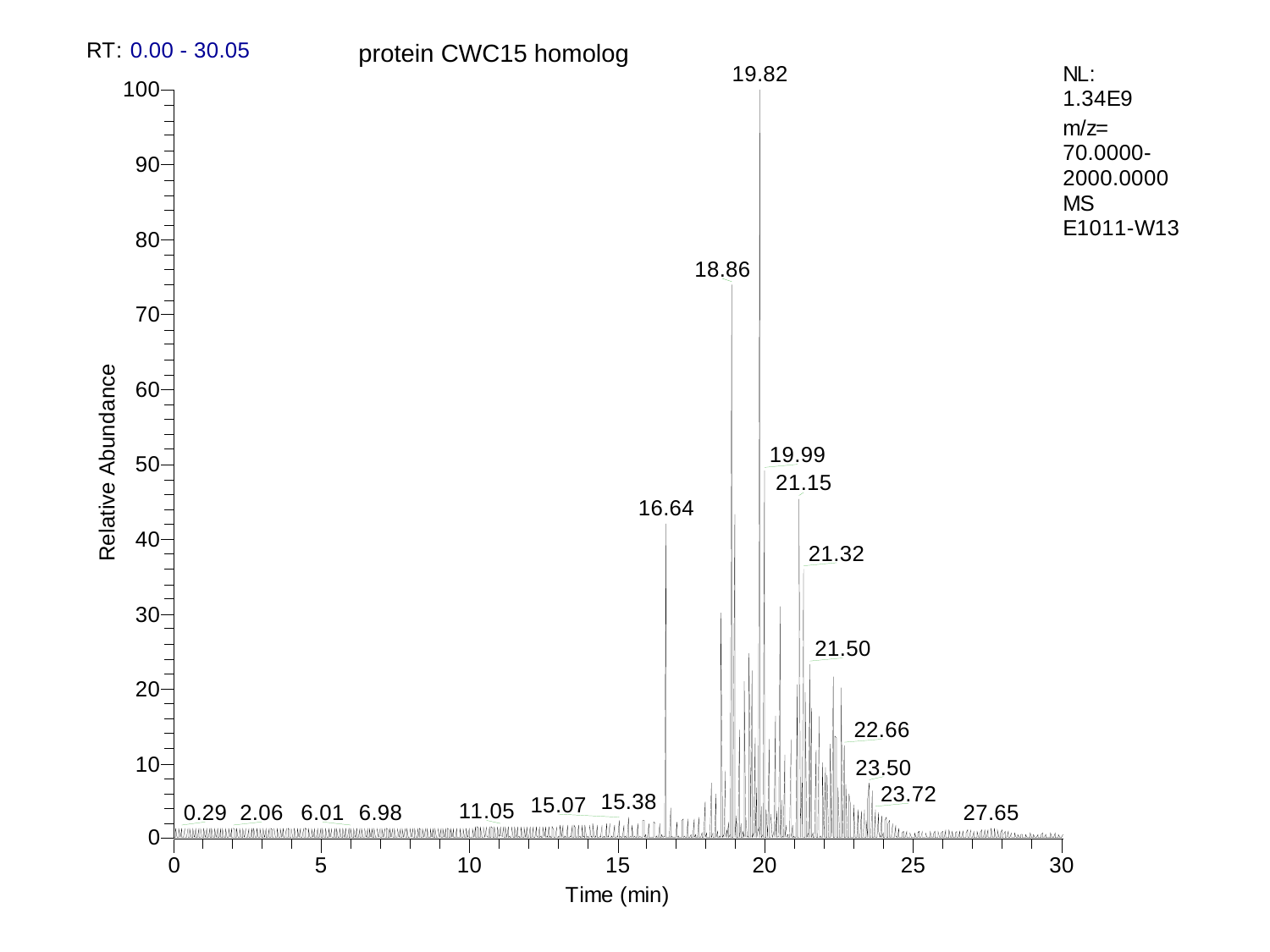

protein CWC15 homolog

## Slide 108
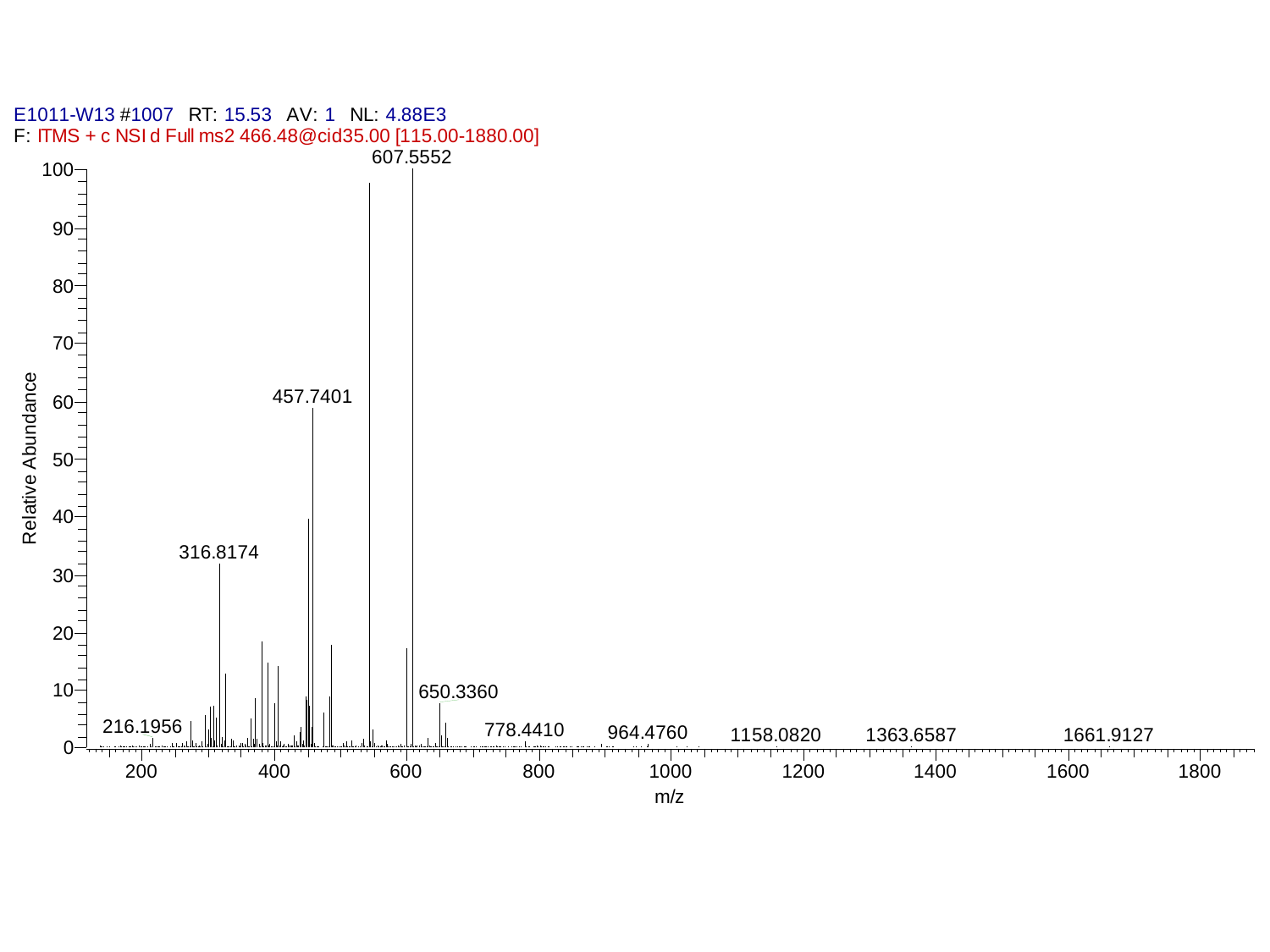

## Slide 109
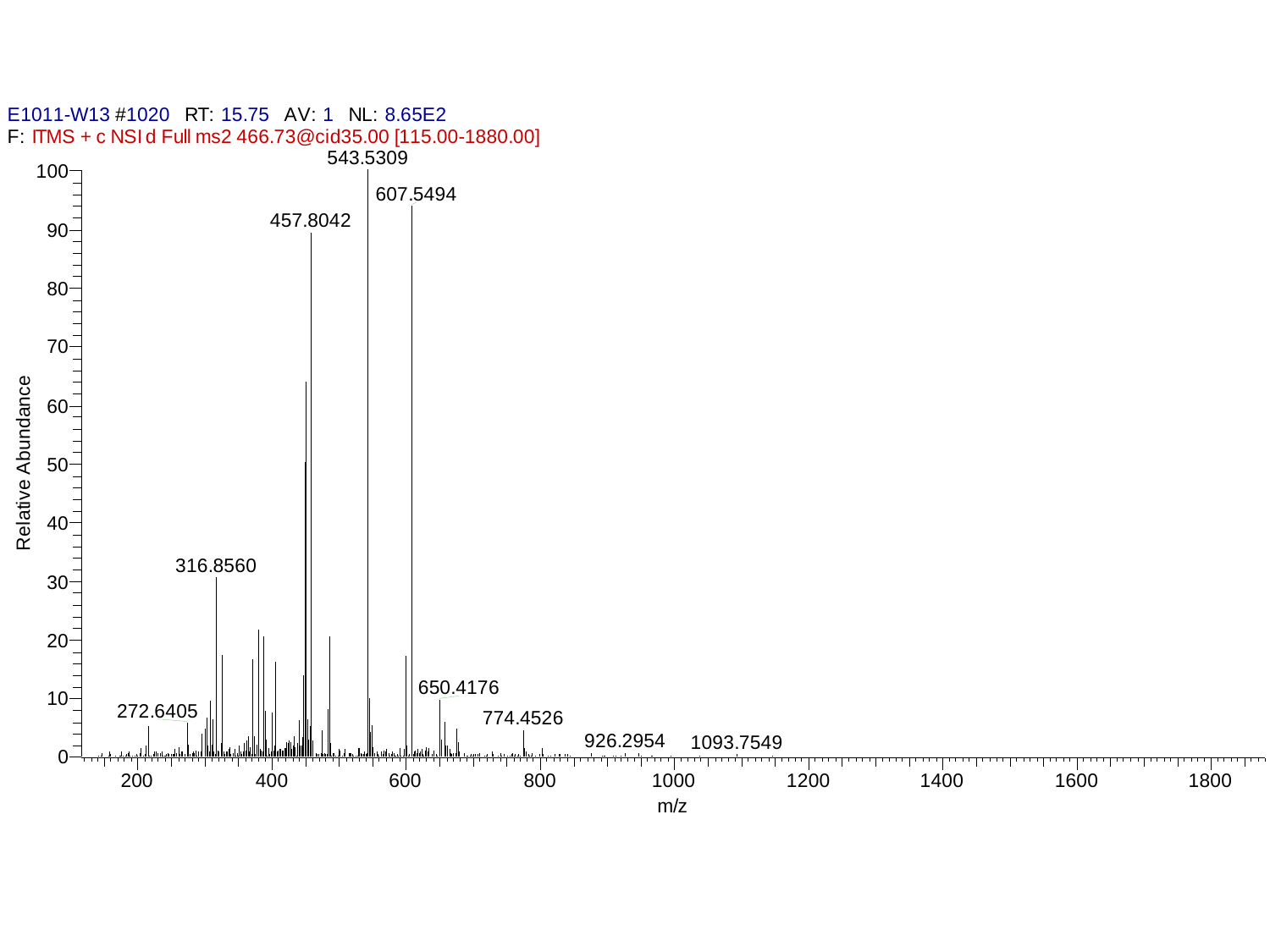

## Slide 110
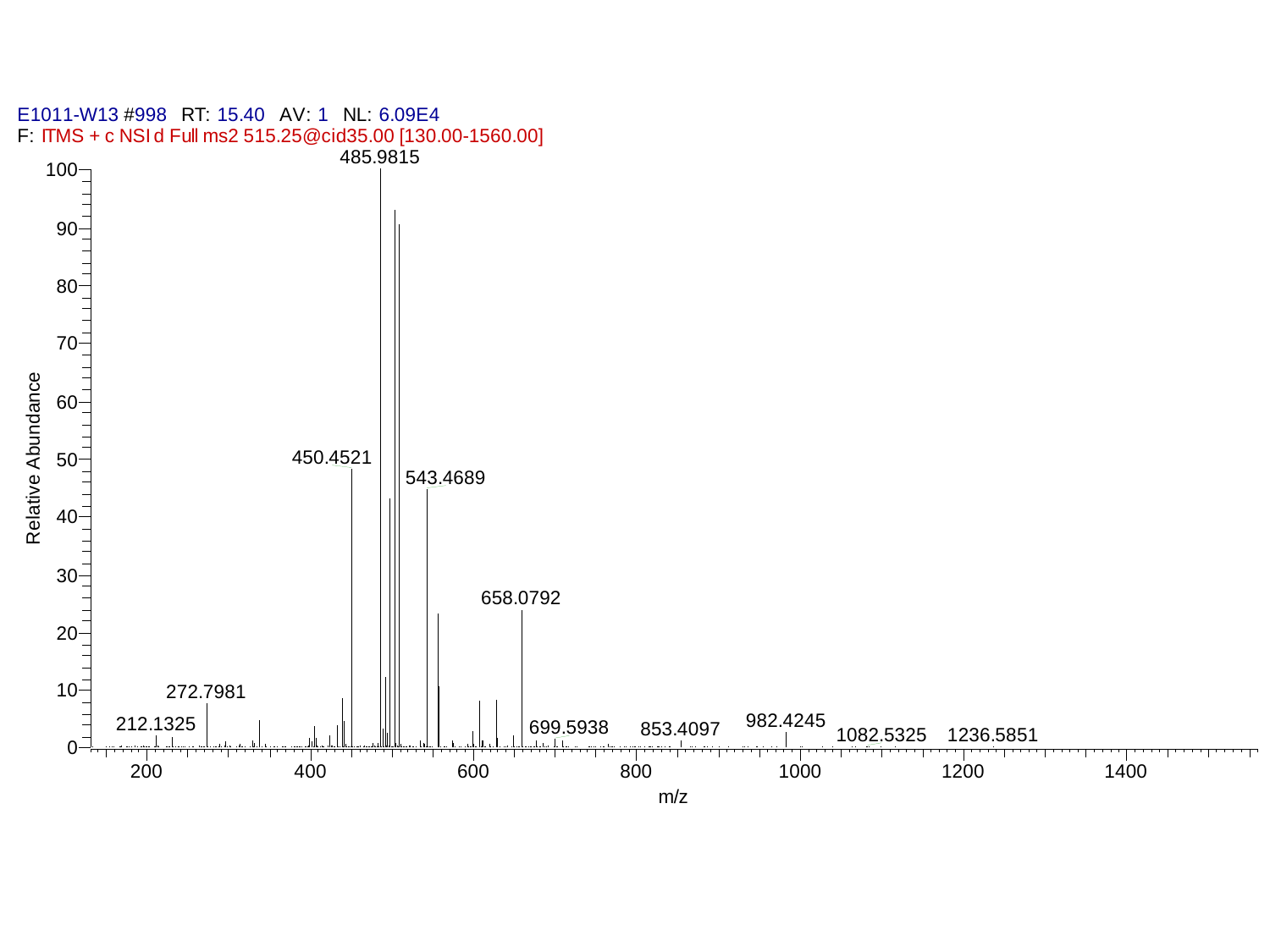

## Slide 111
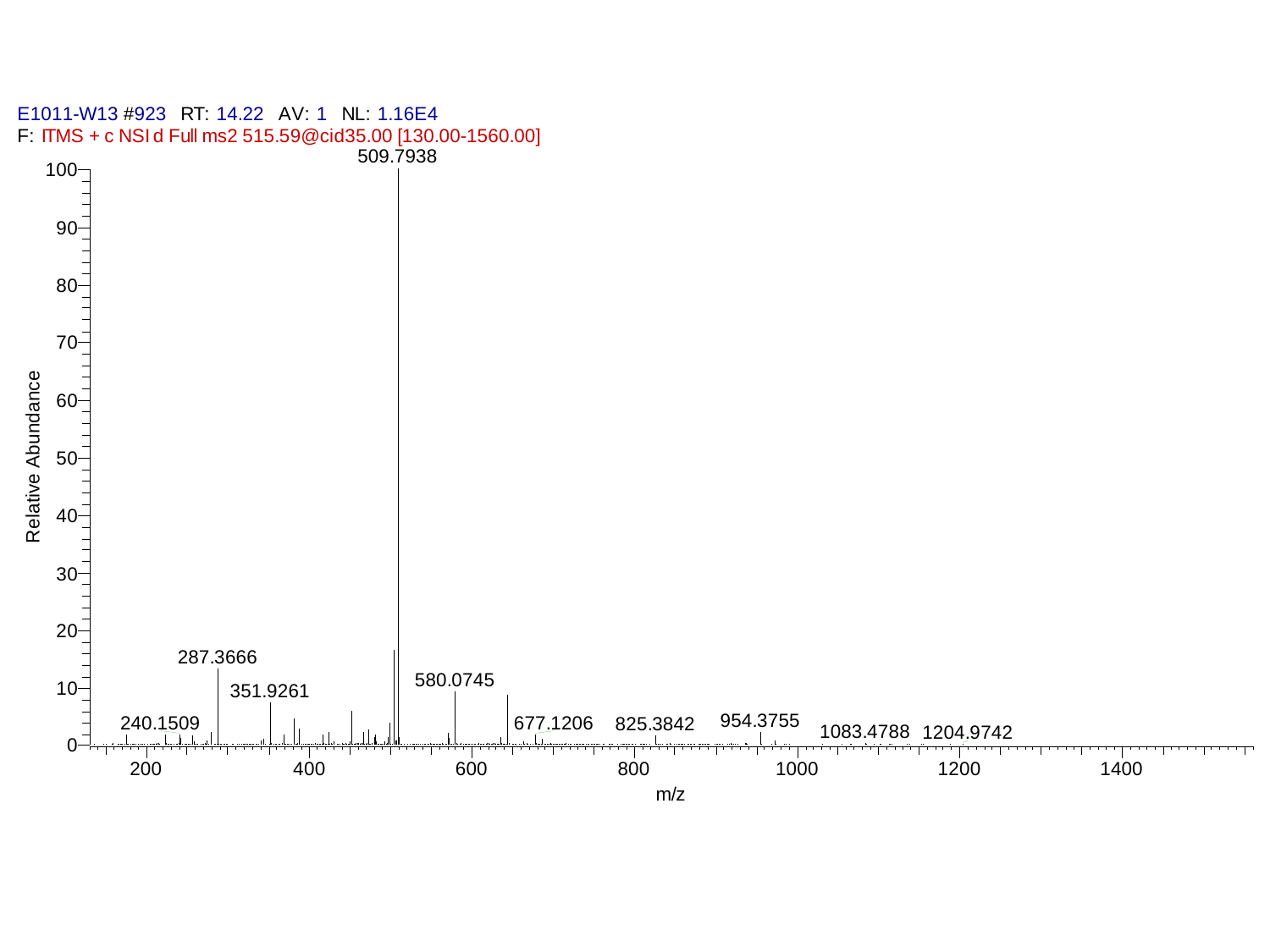

## Slide 112
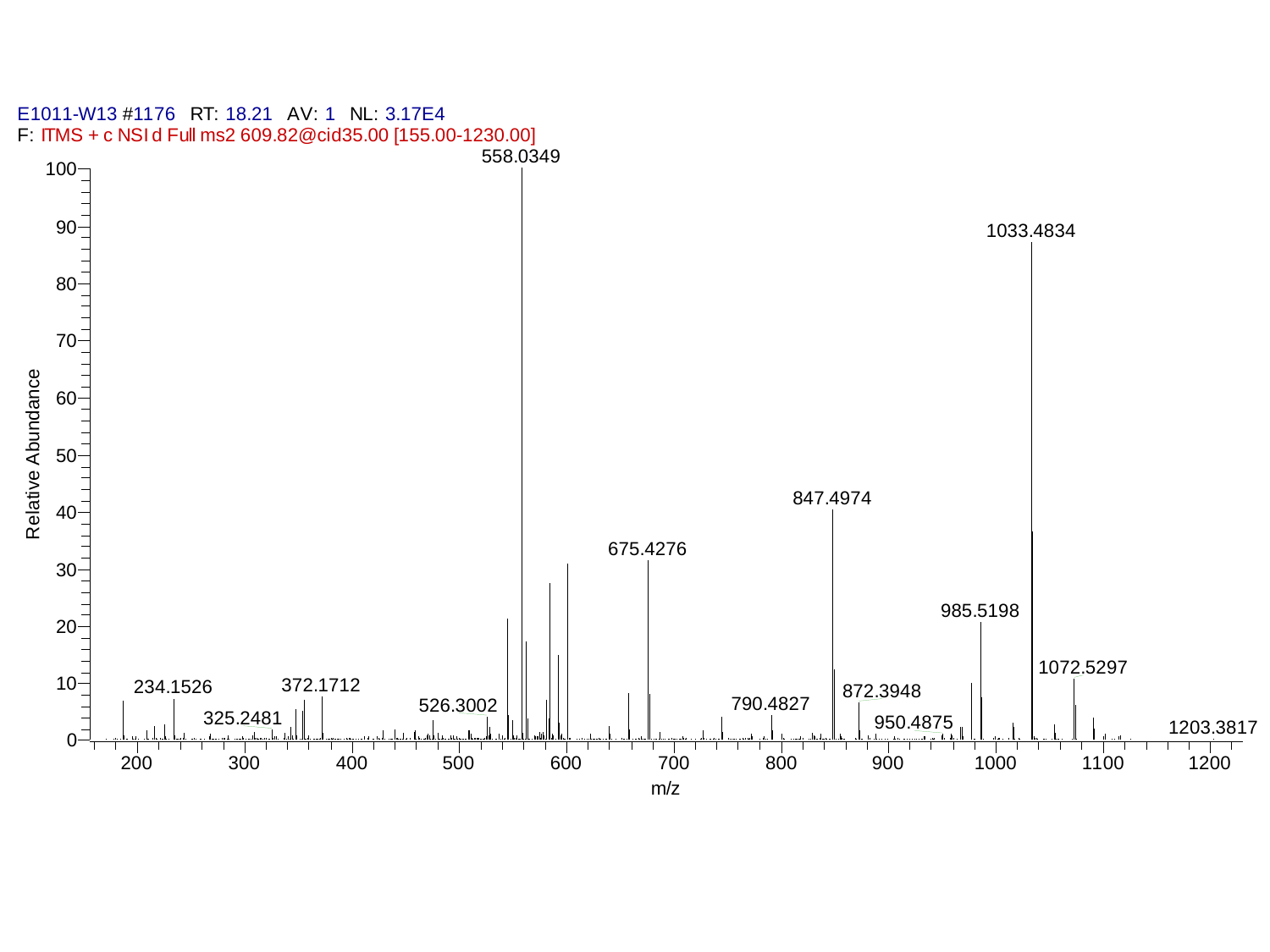

## Slide 113
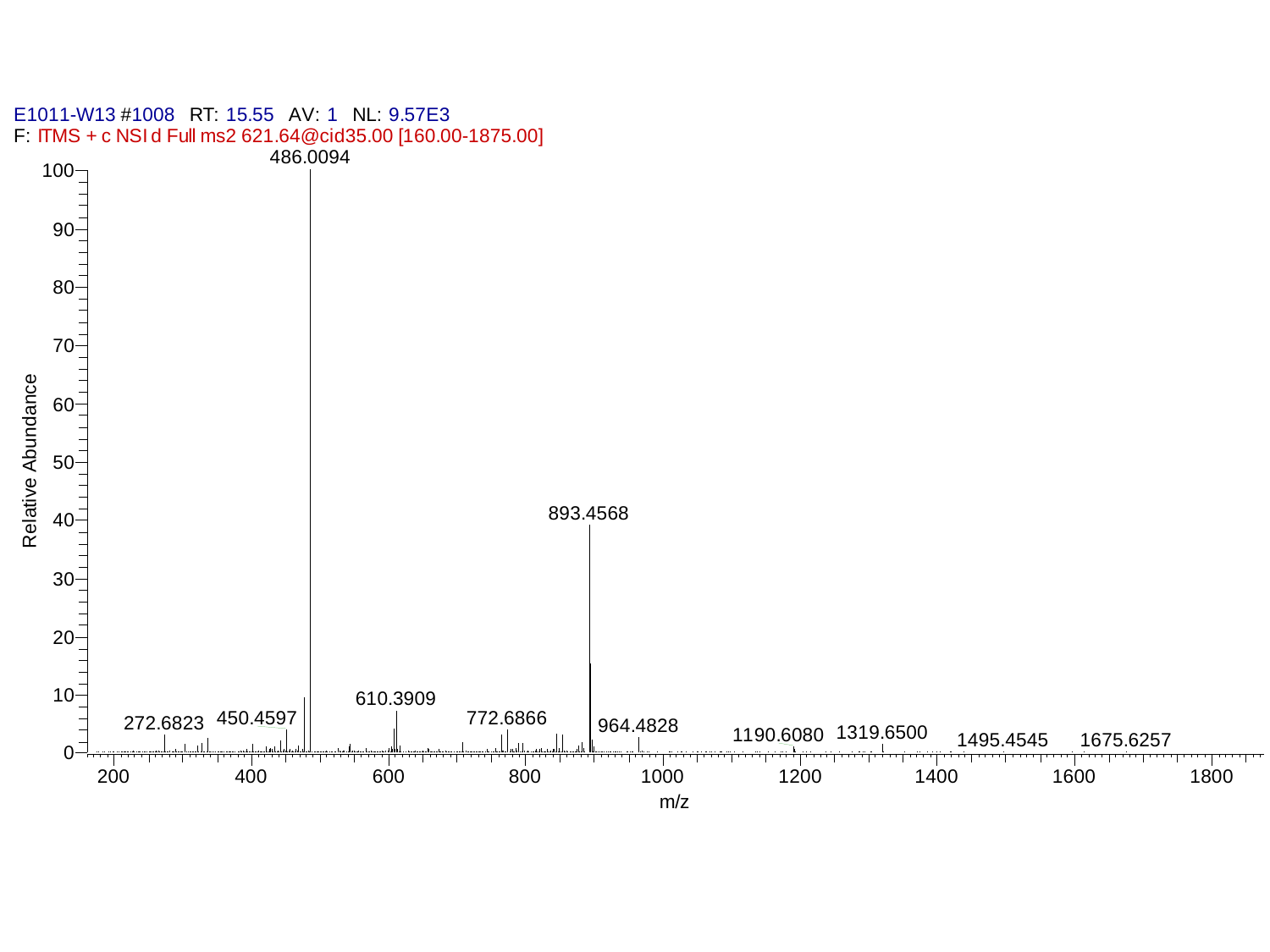

## Slide 114
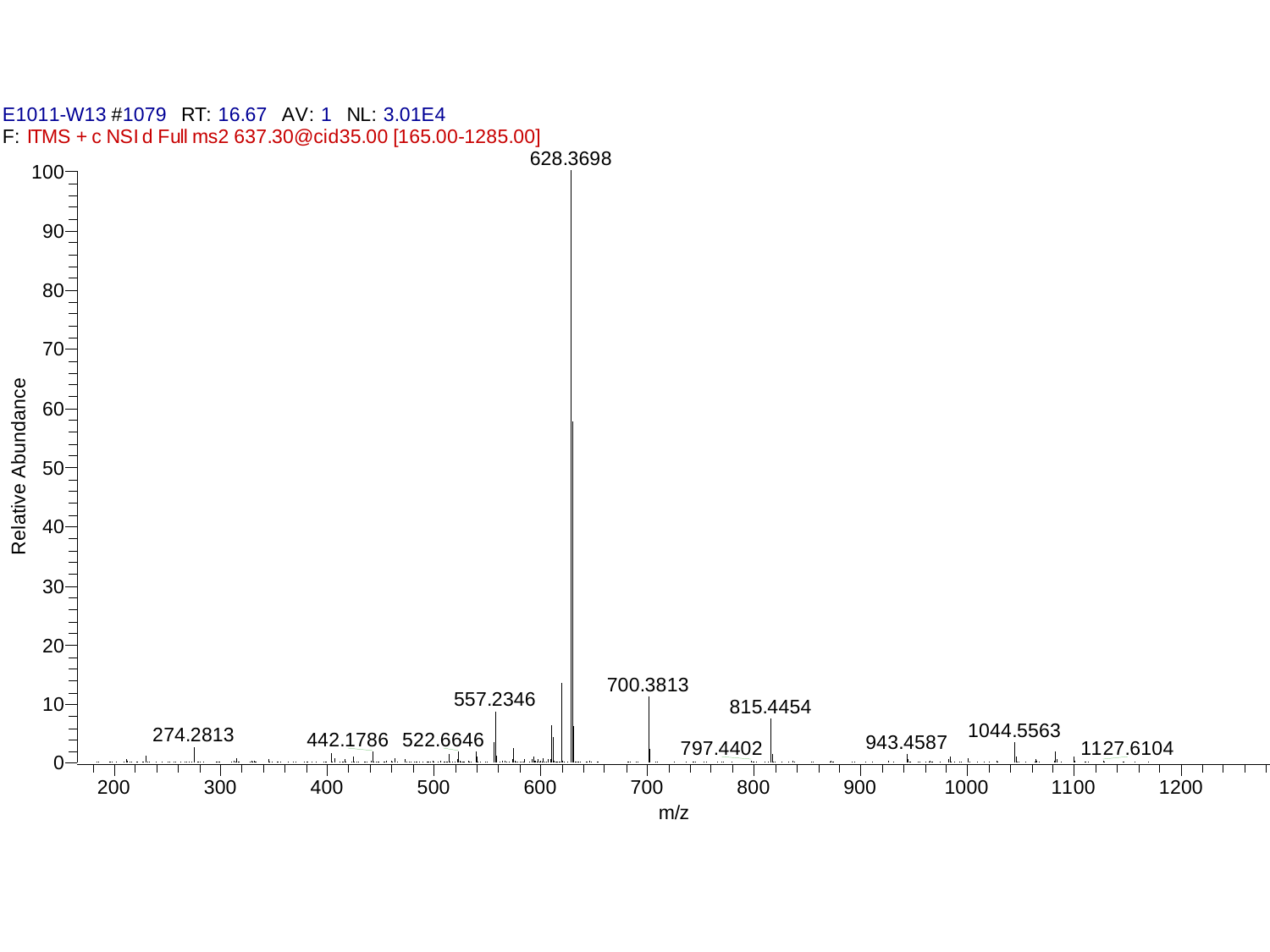

## Slide 115
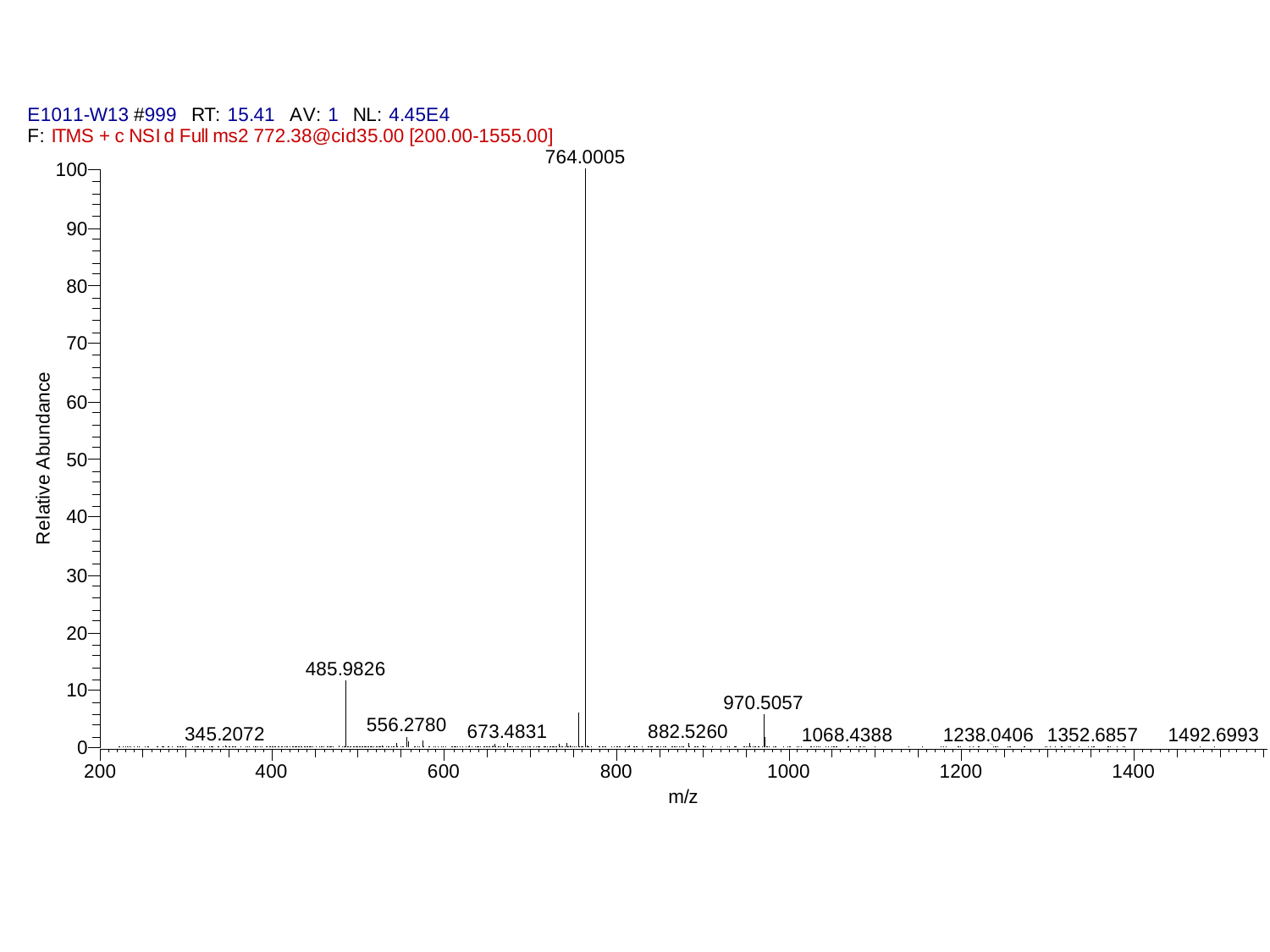

## Slide 116
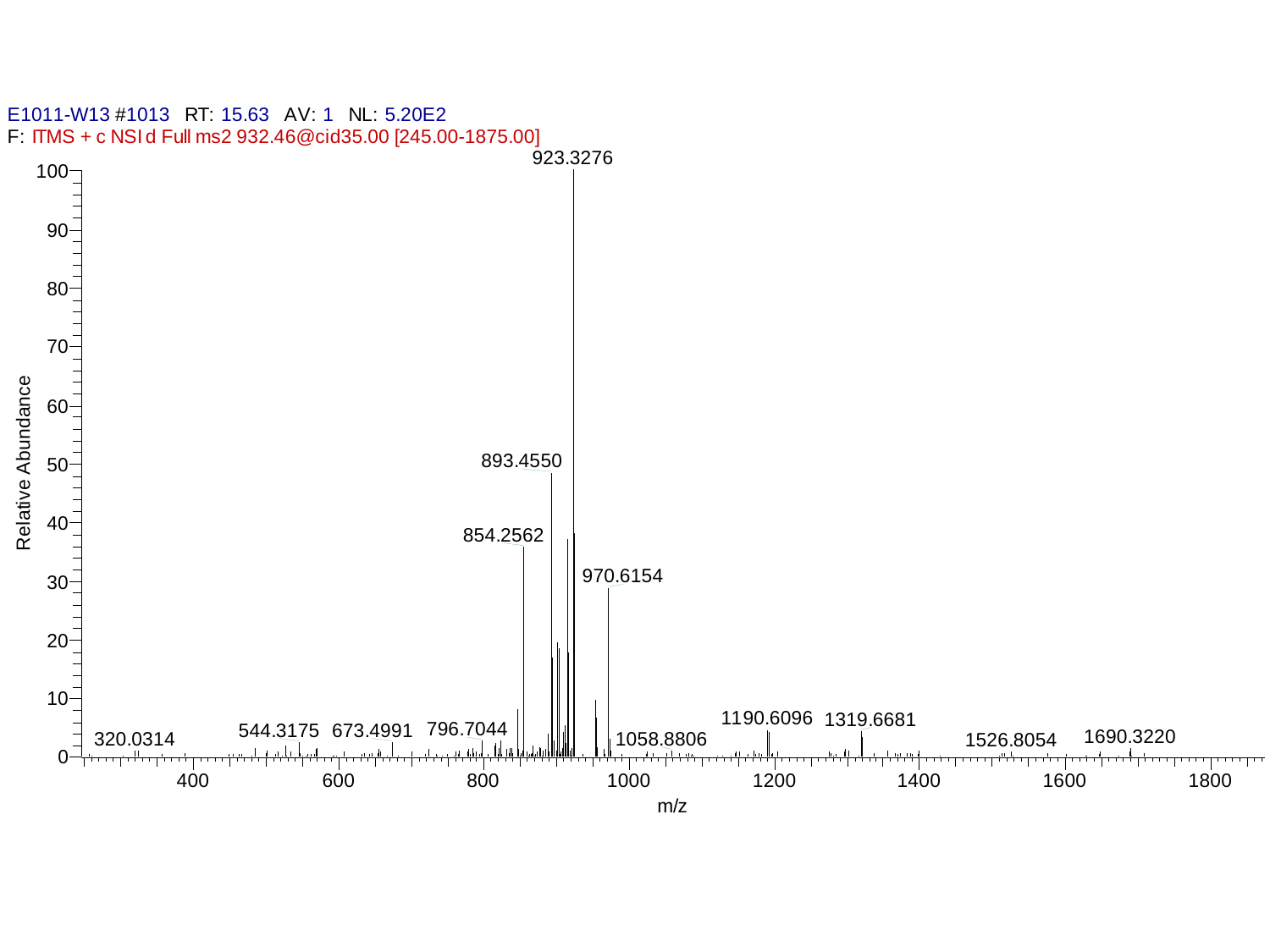

## Slide 117
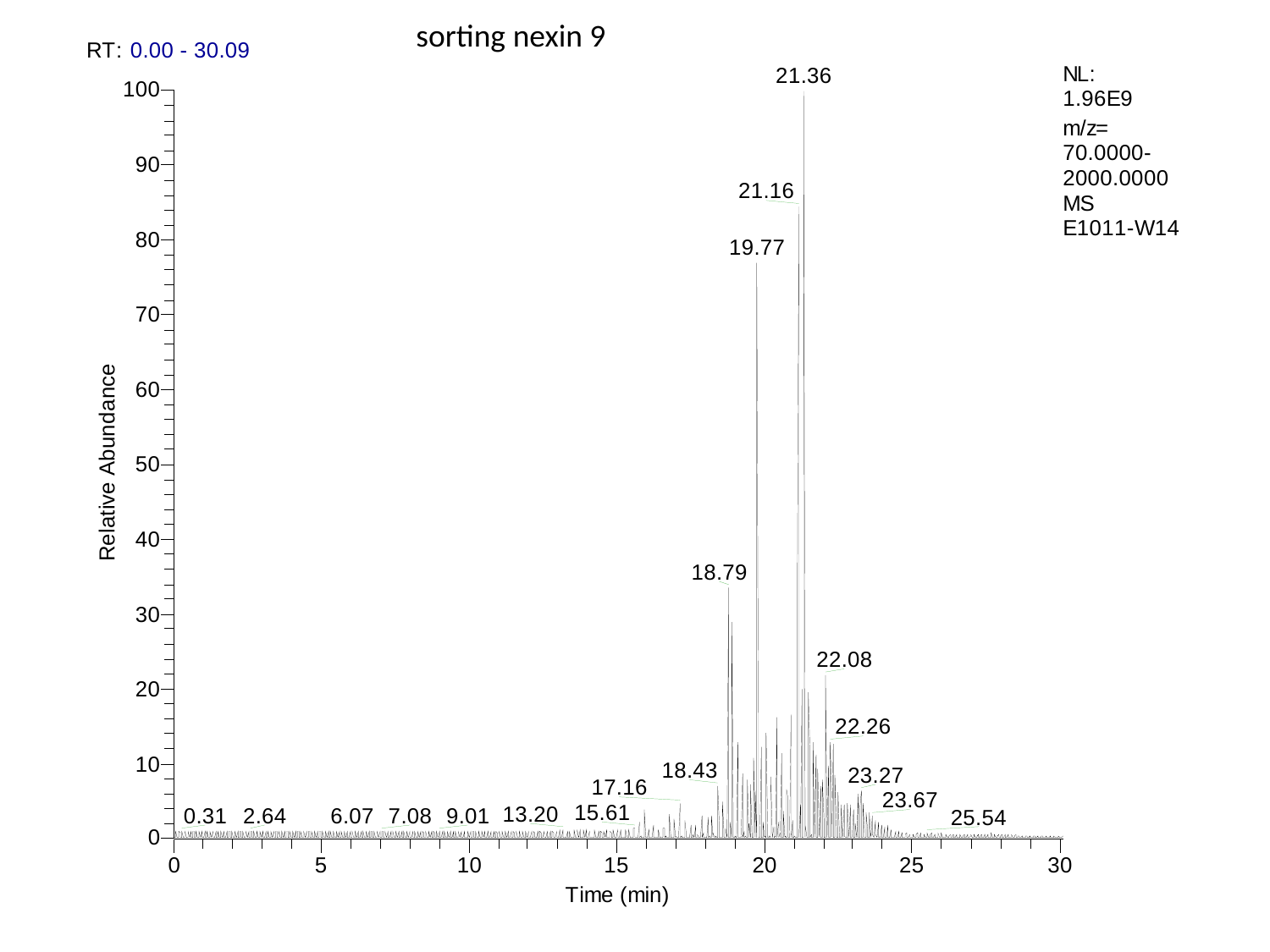

sorting nexin 9

## Slide 118
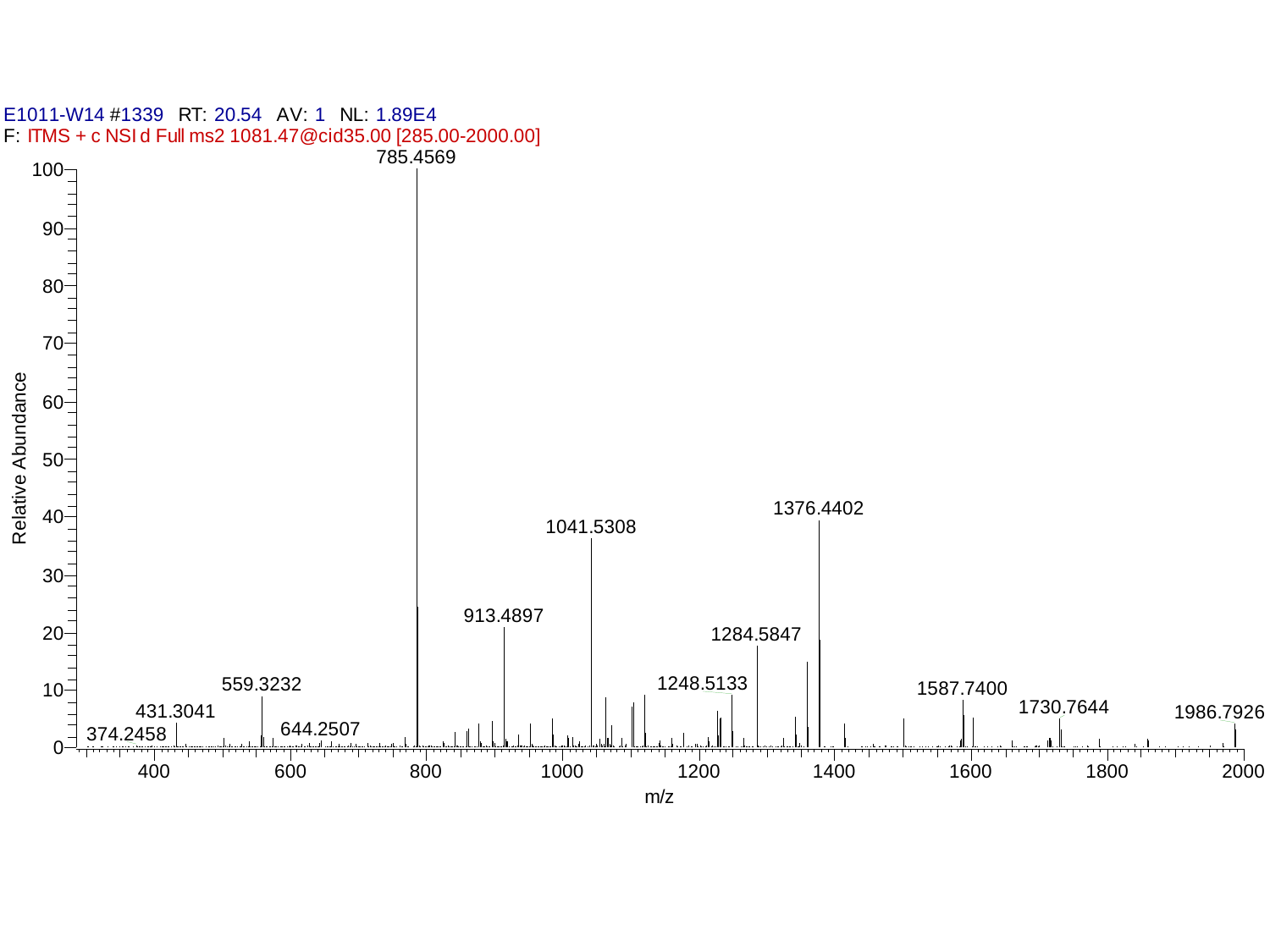

## Slide 119
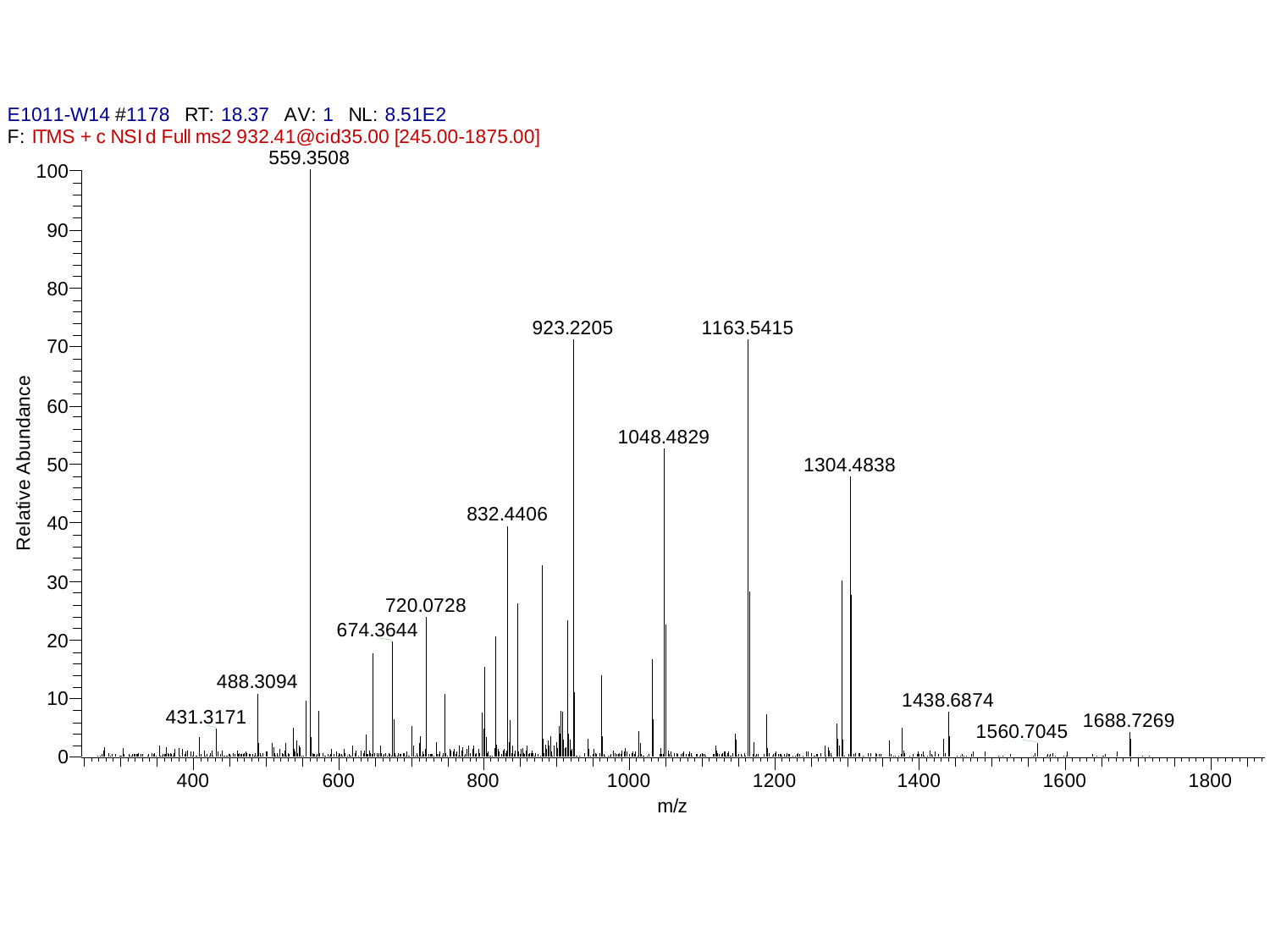

## Slide 120
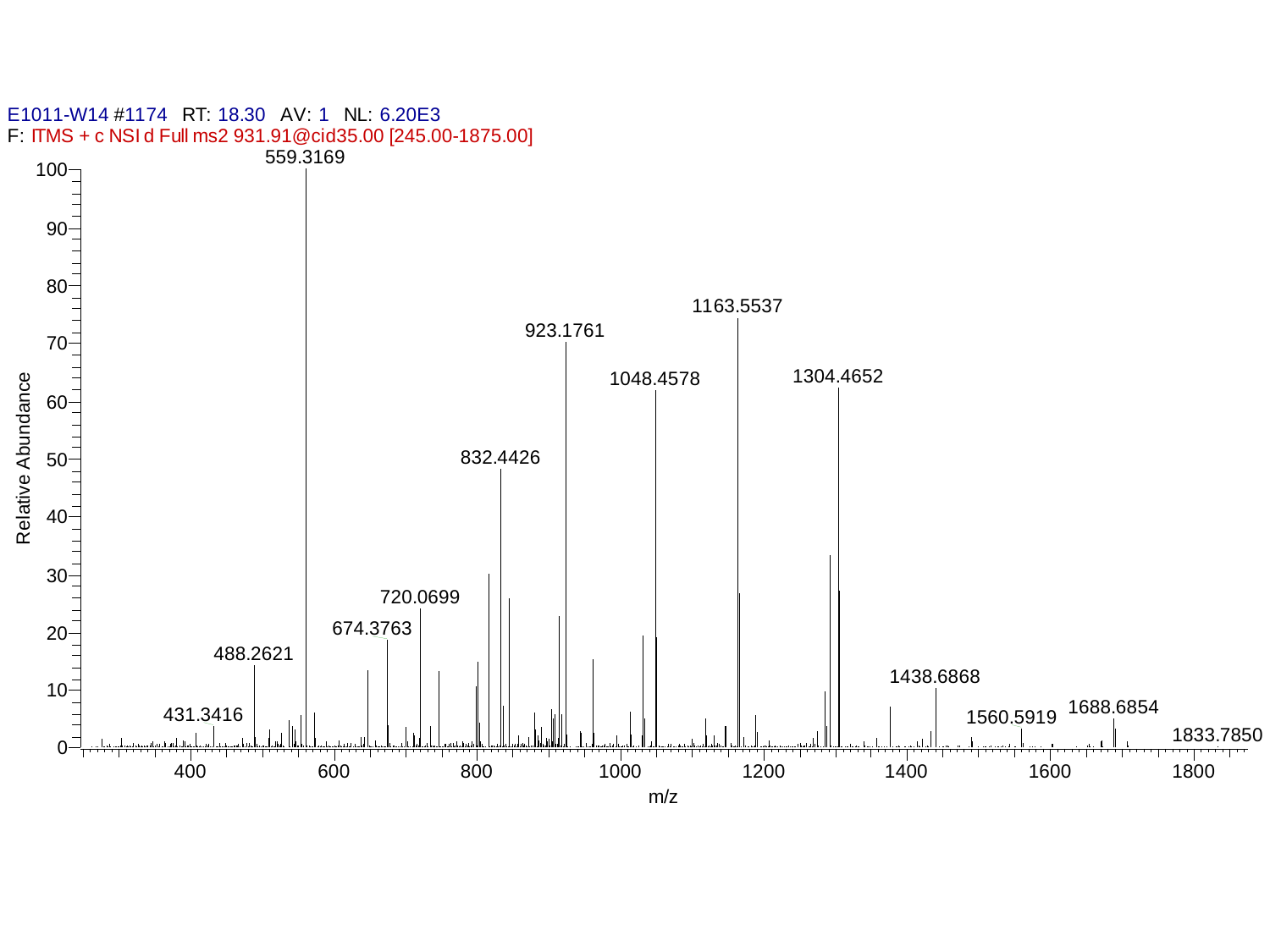

## Slide 121
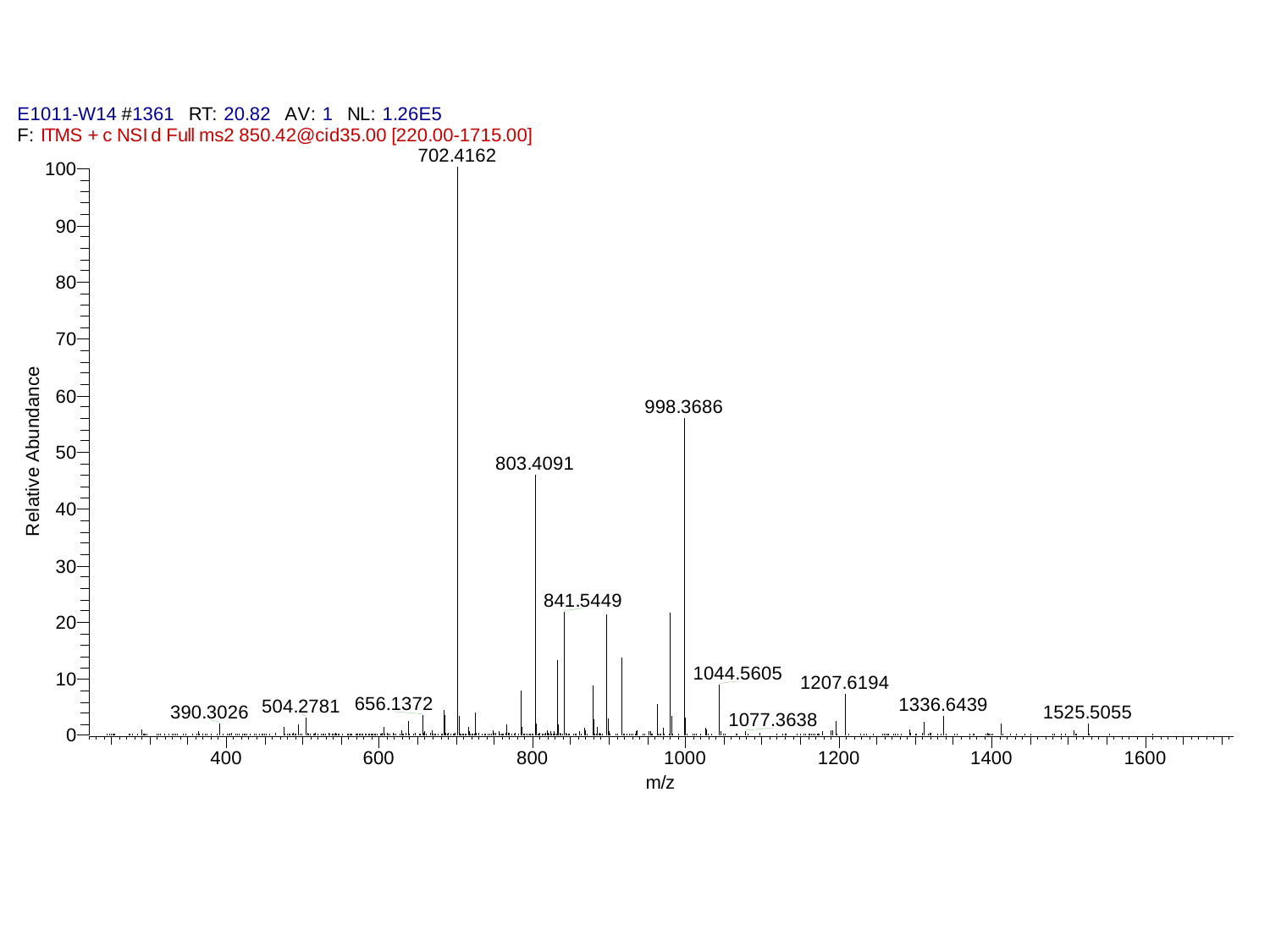

## Slide 122
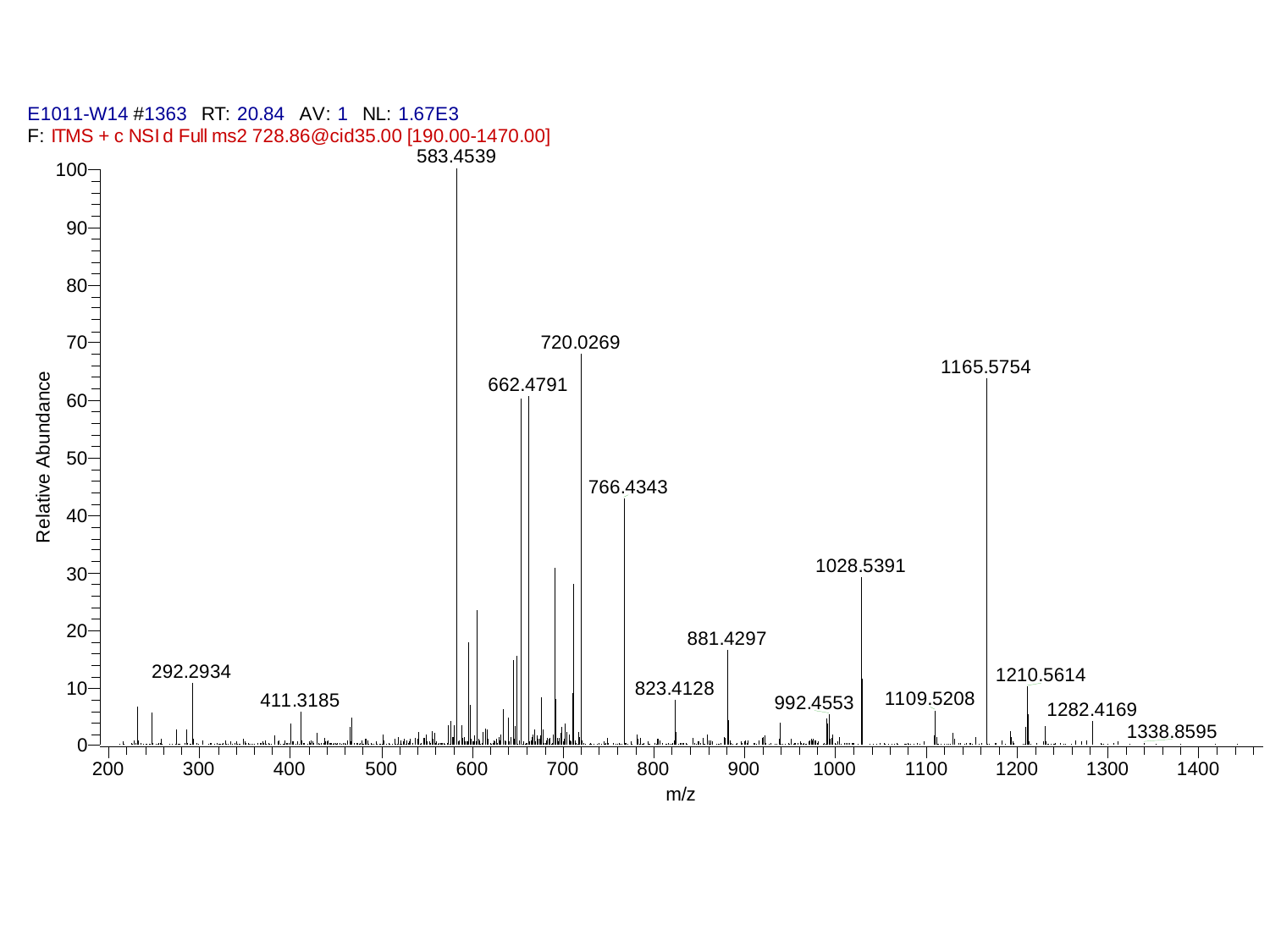

## Slide 123
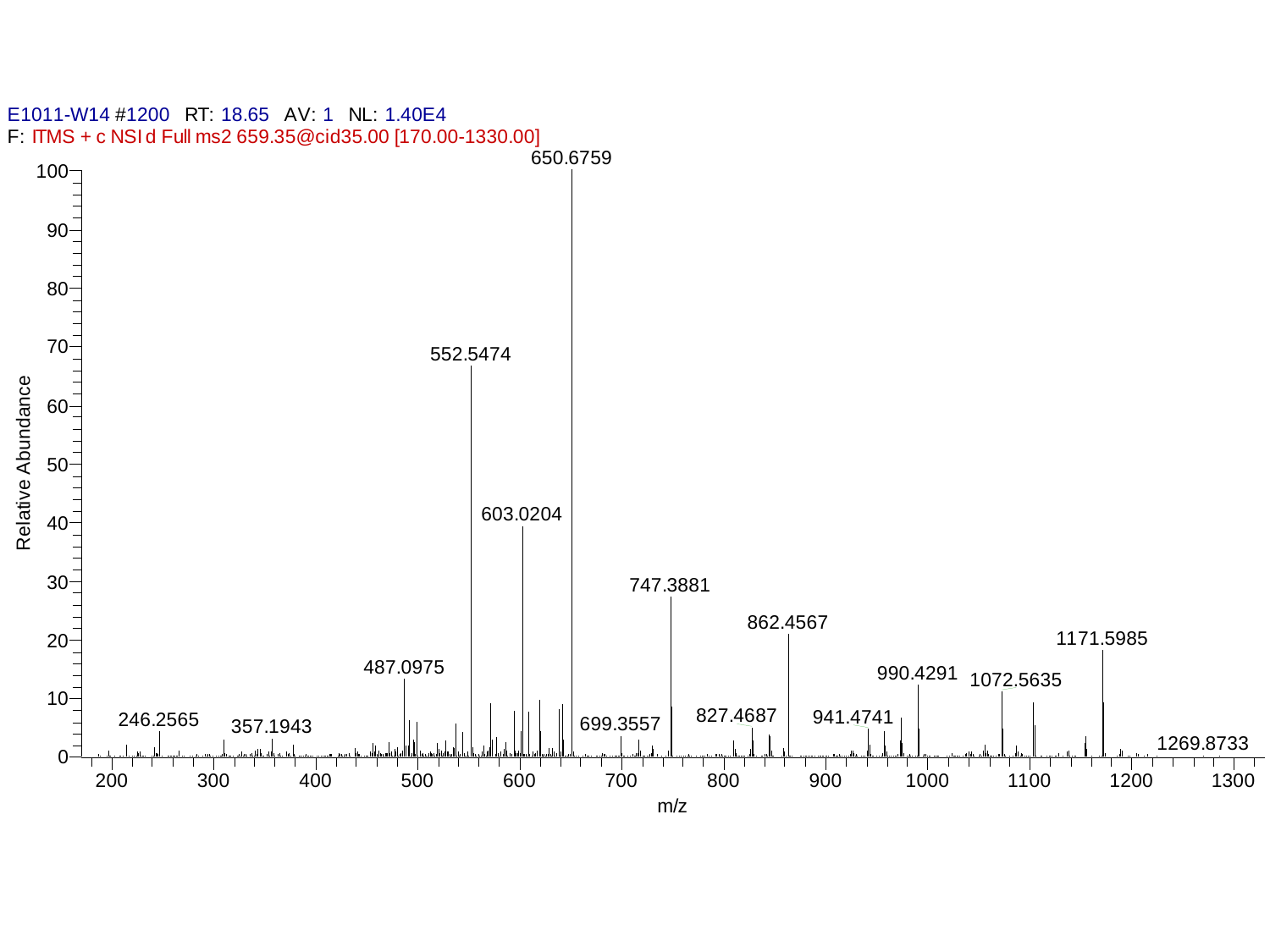

## Slide 124
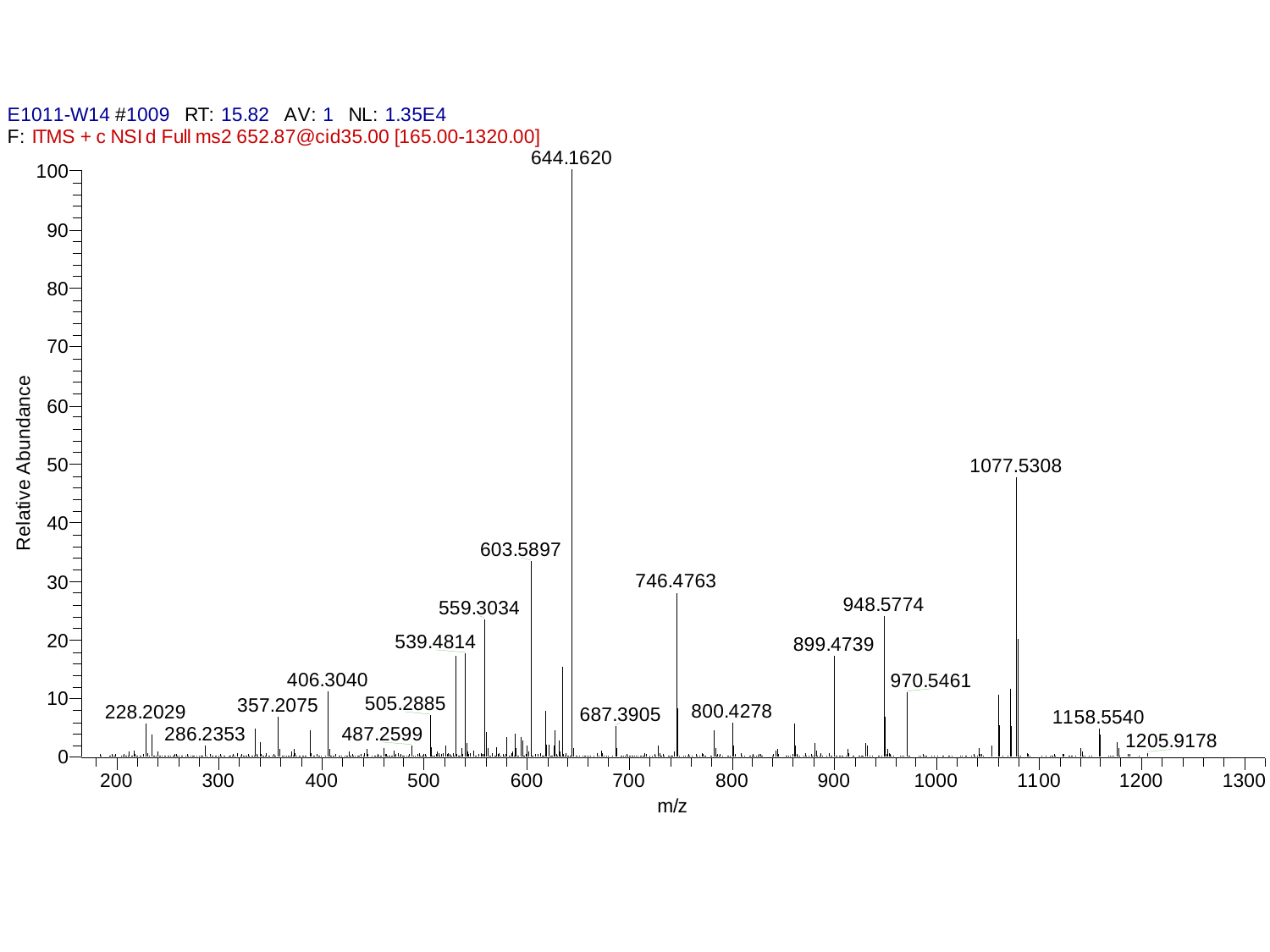

## Slide 125
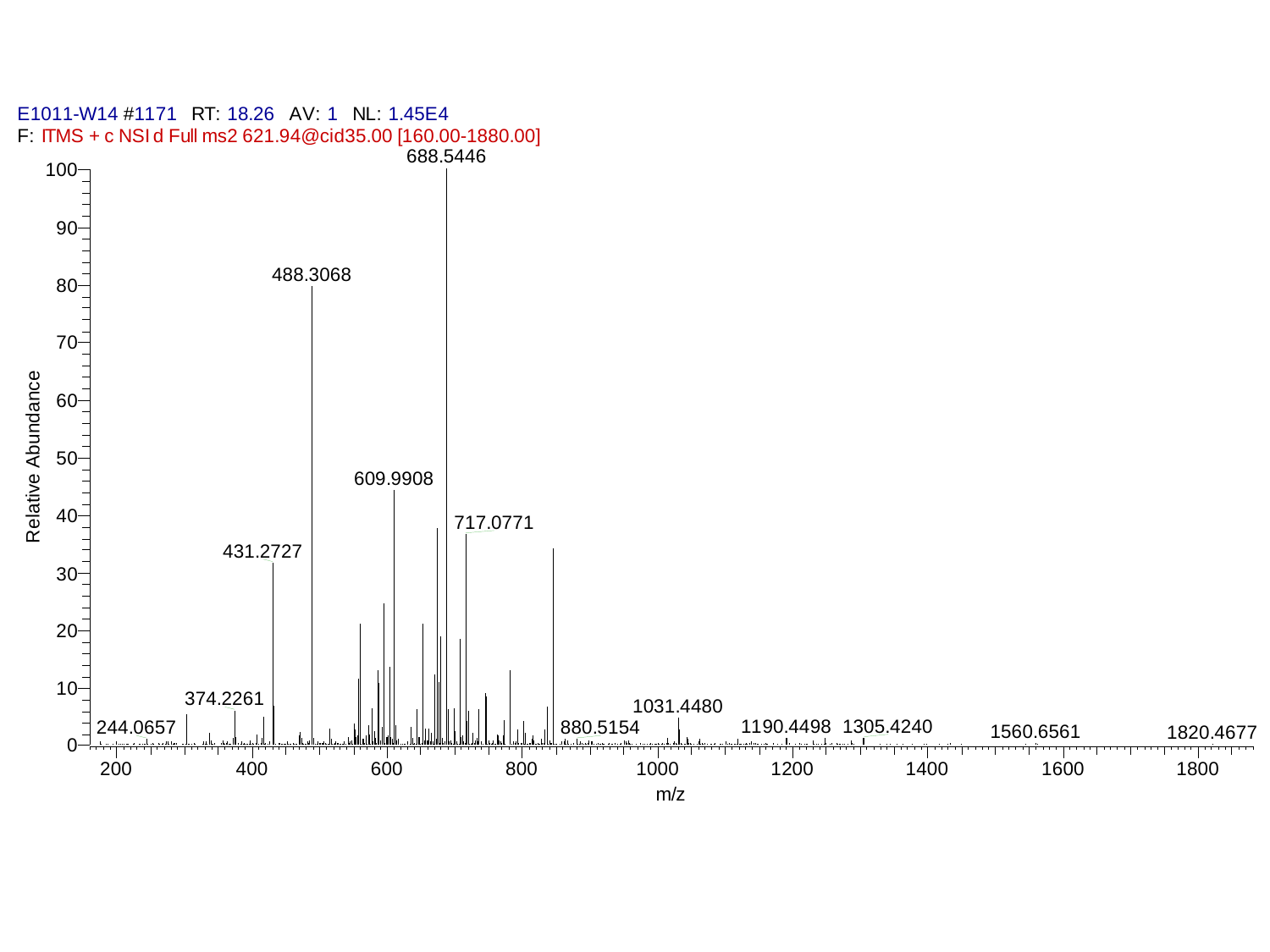

## Slide 126
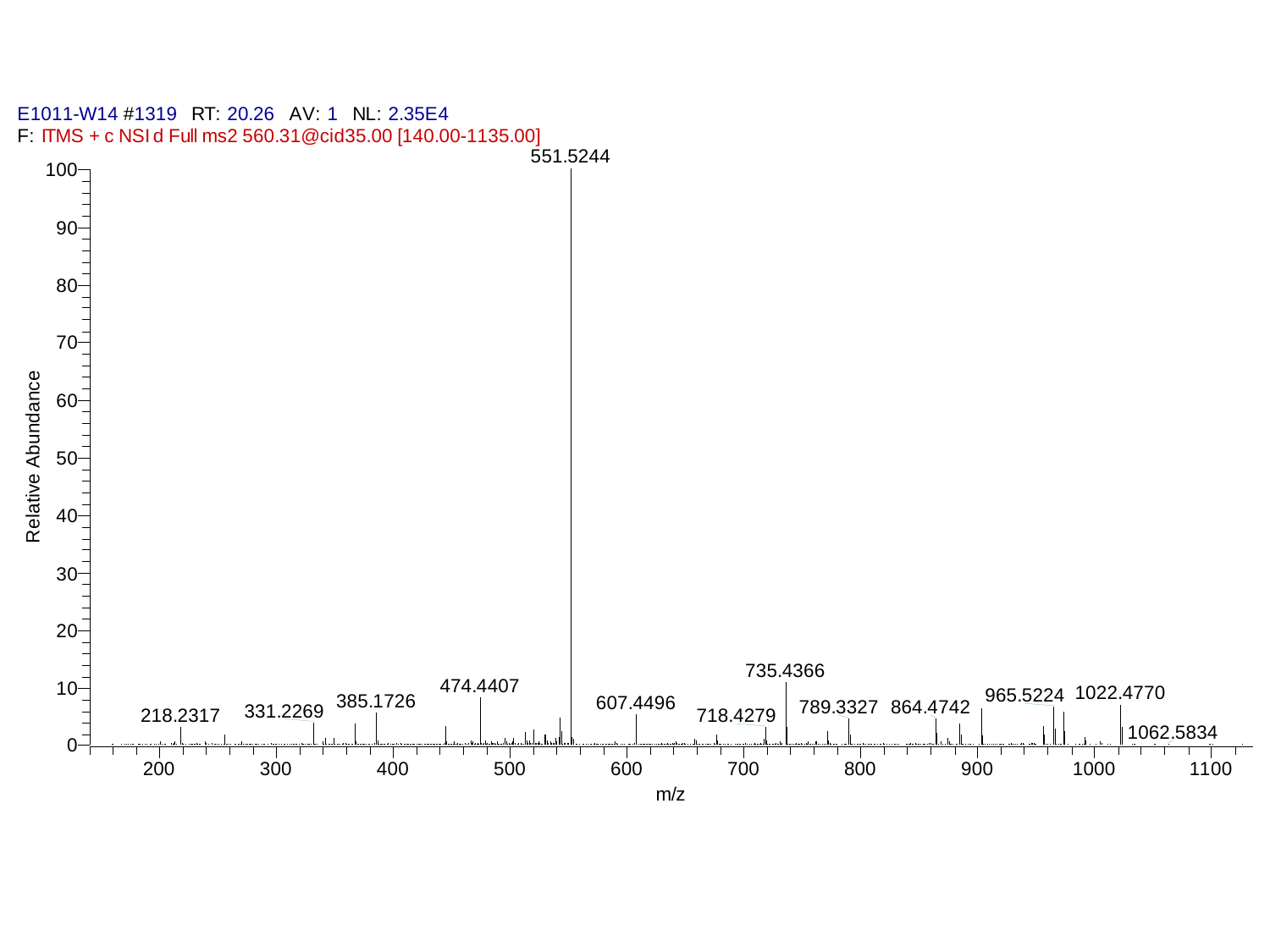

## Slide 127
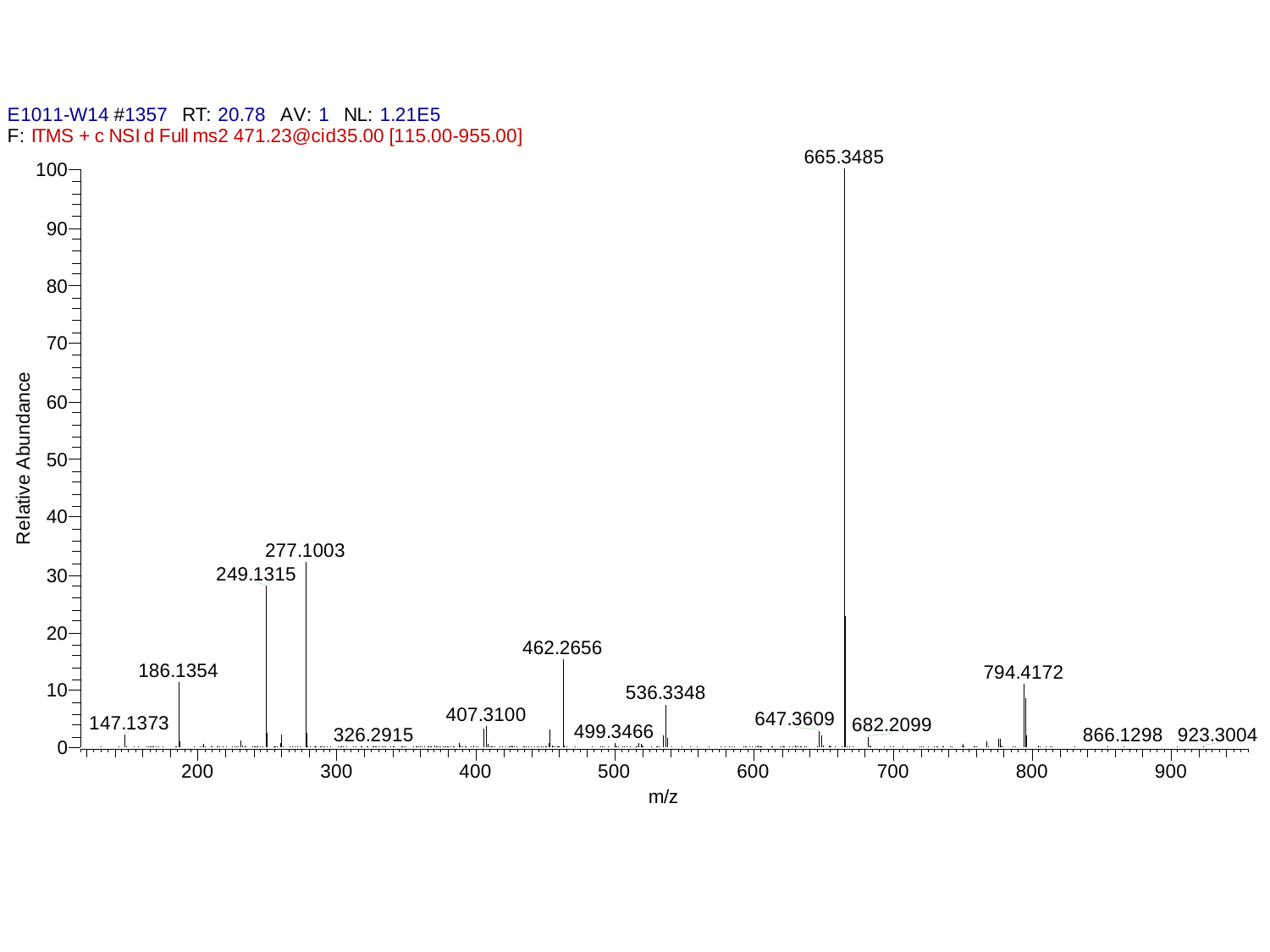

## Slide 128
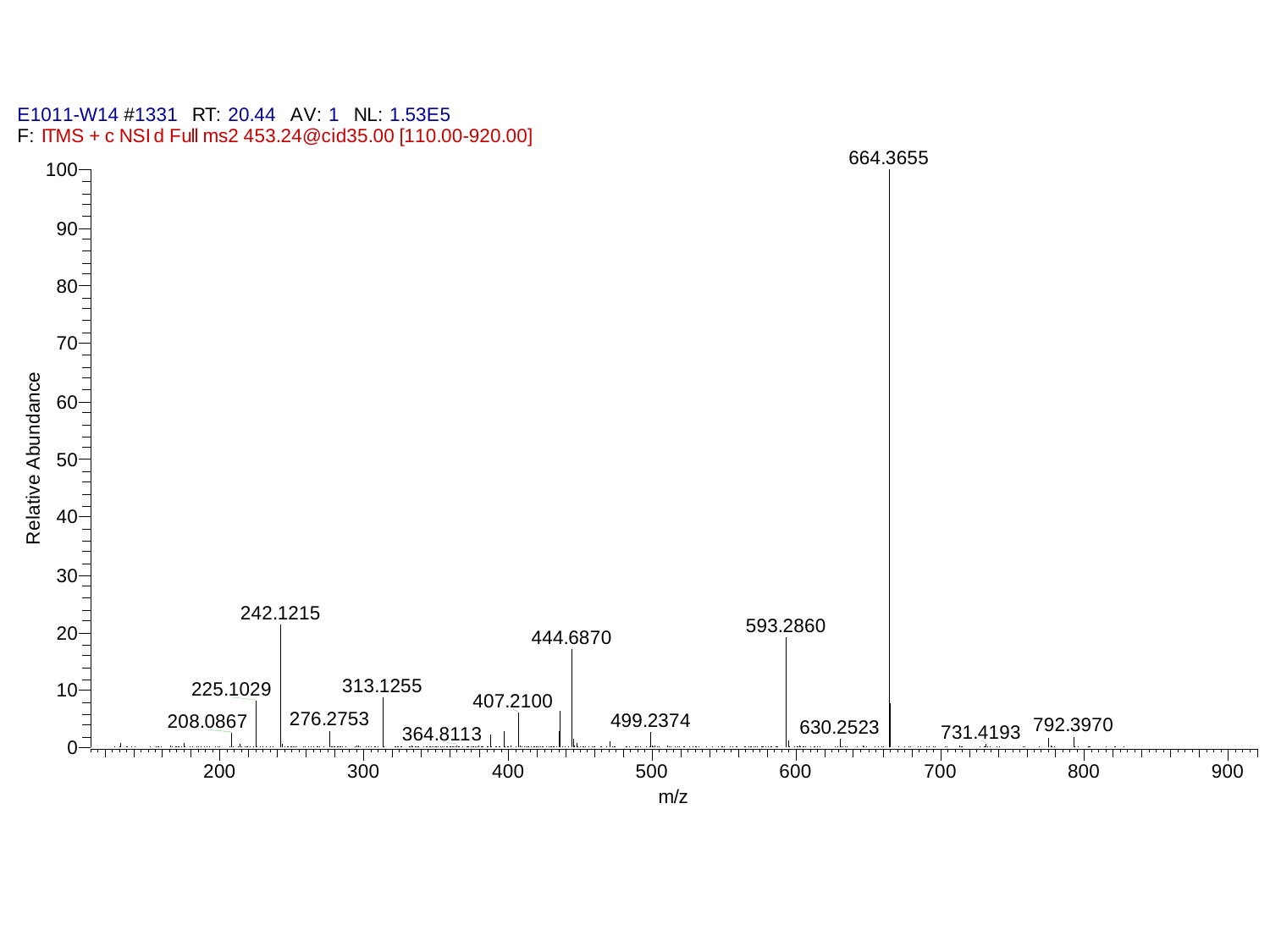

## Slide 129
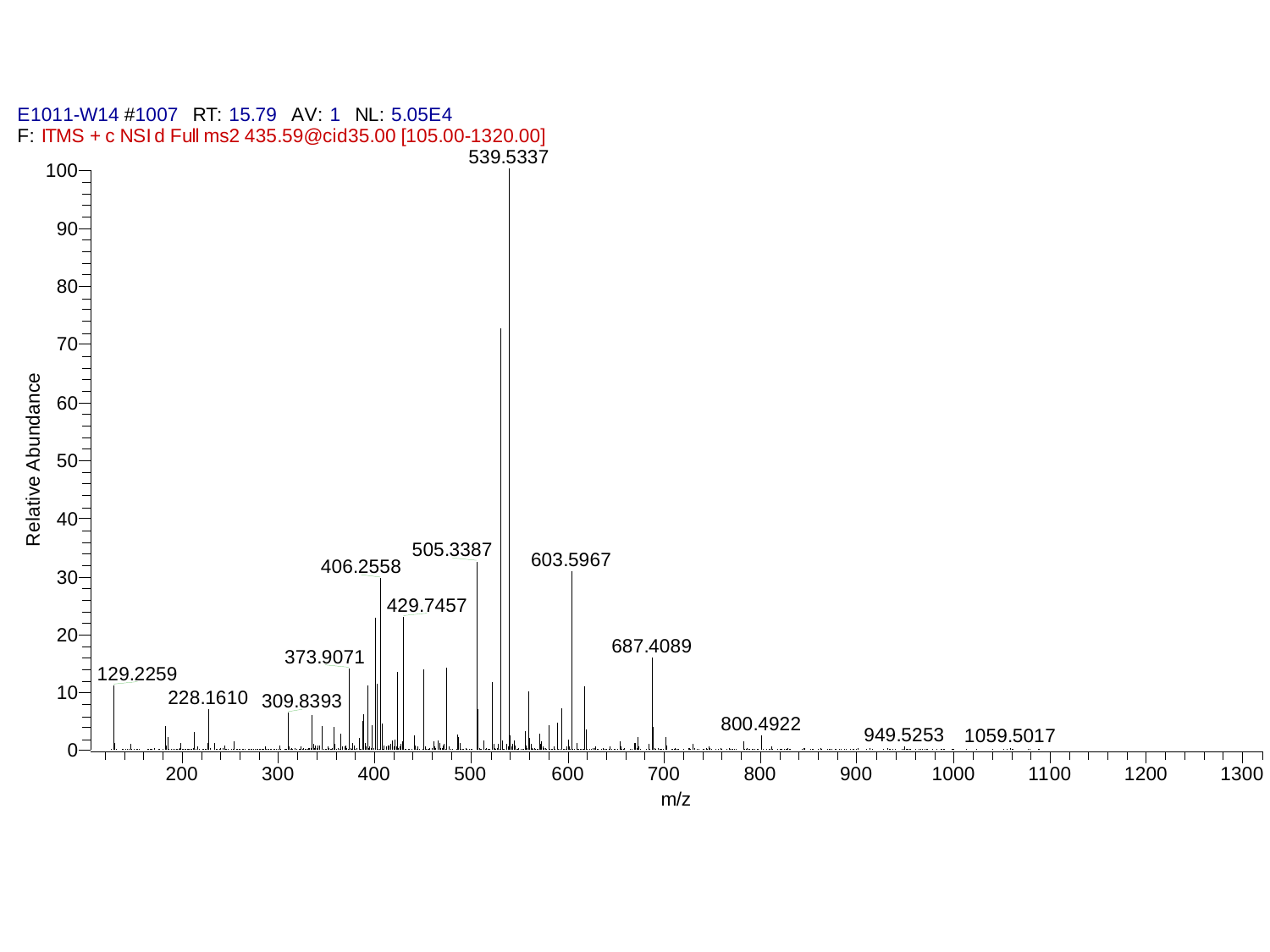

## Slide 130
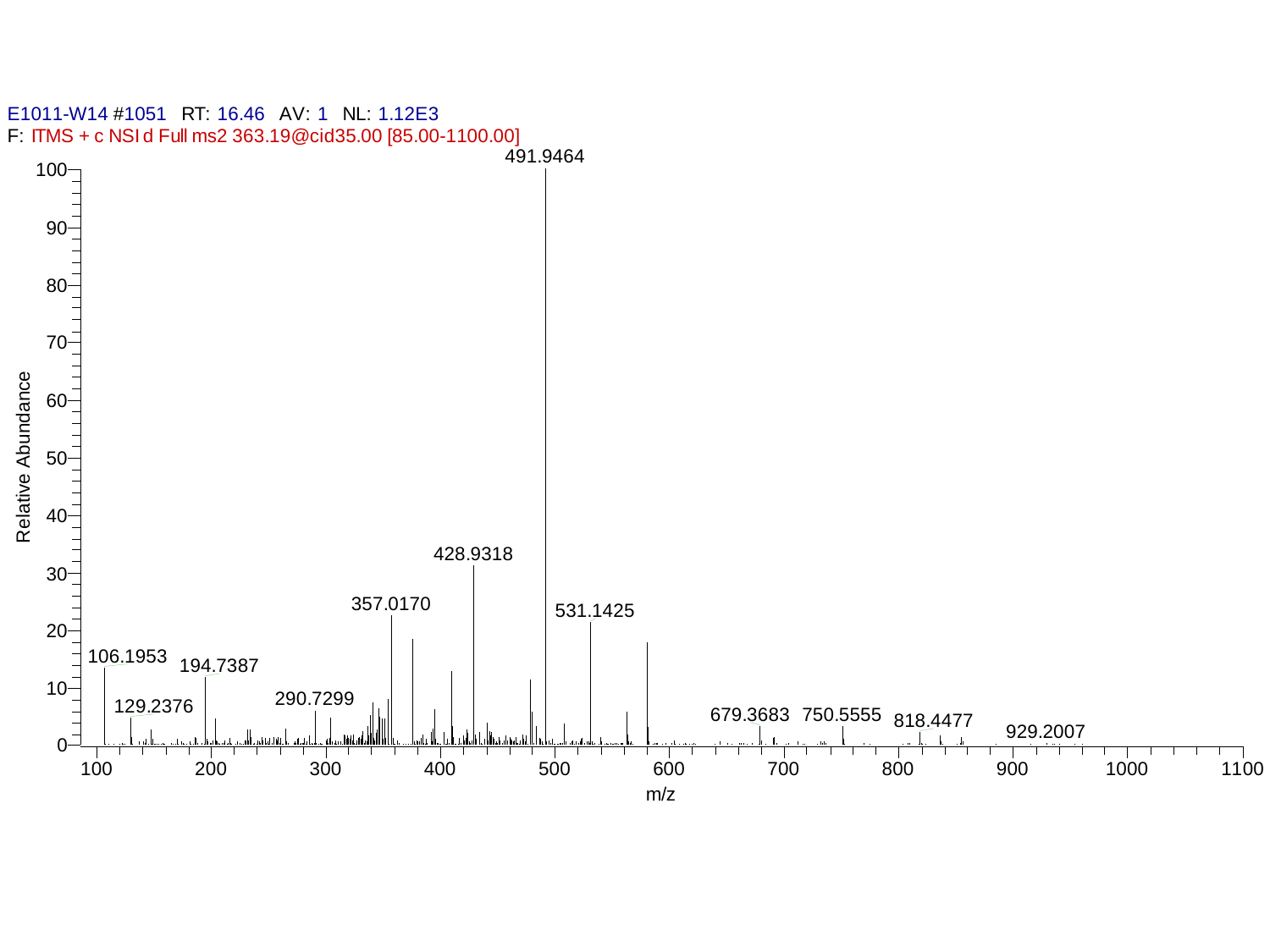

## Slide 131
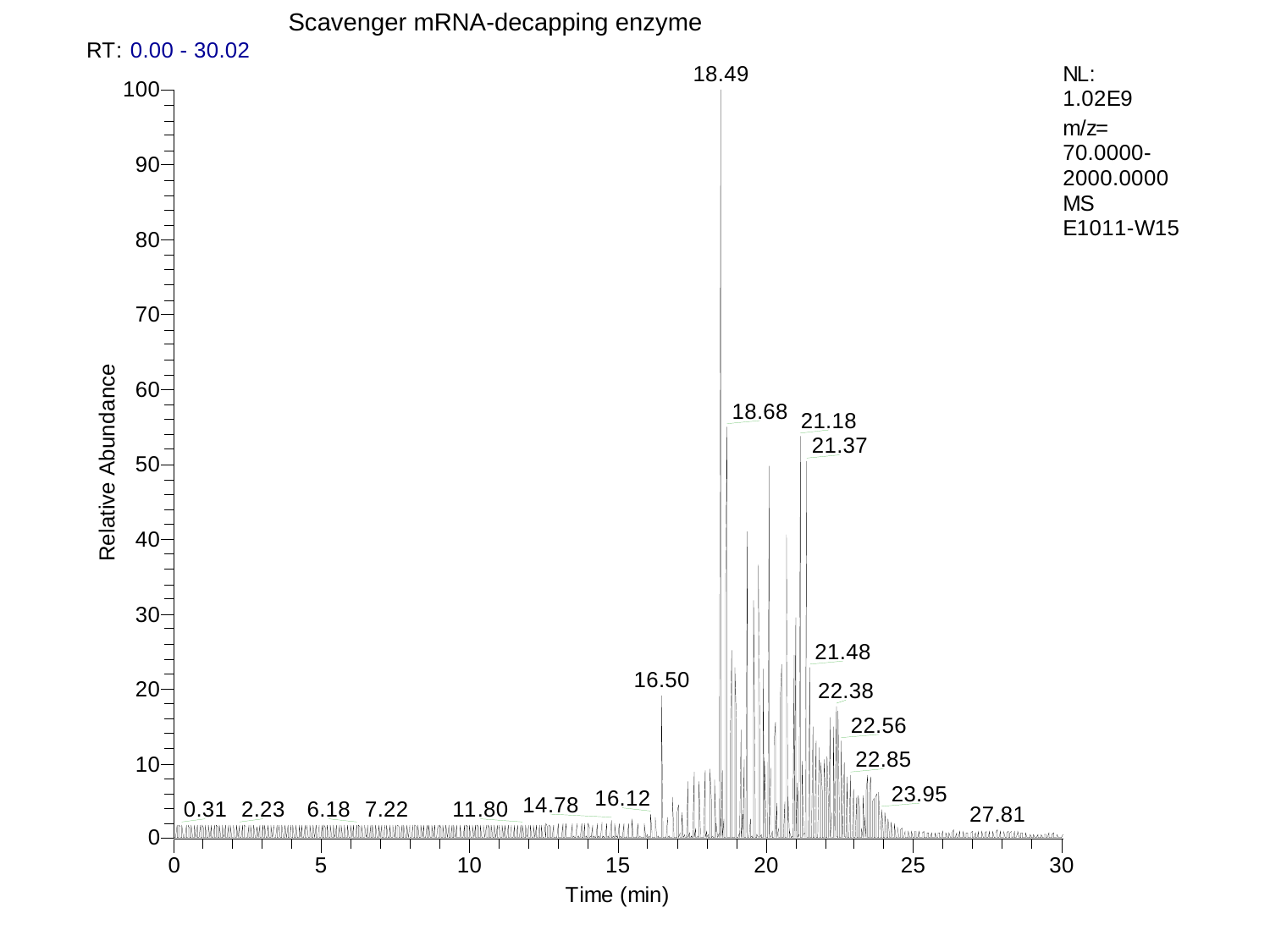

Scavenger mRNA-decapping enzyme

## Slide 132
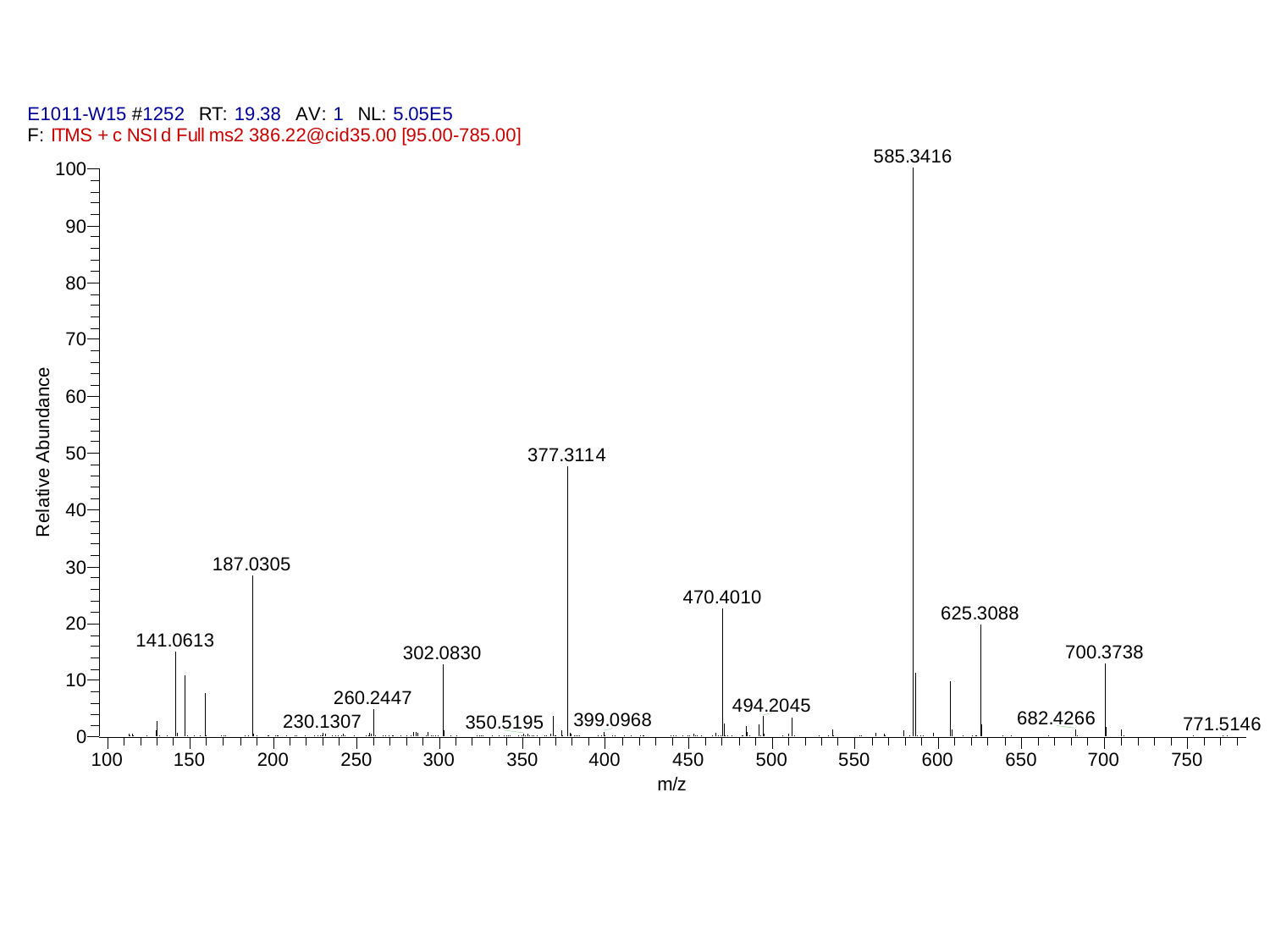

## Slide 133
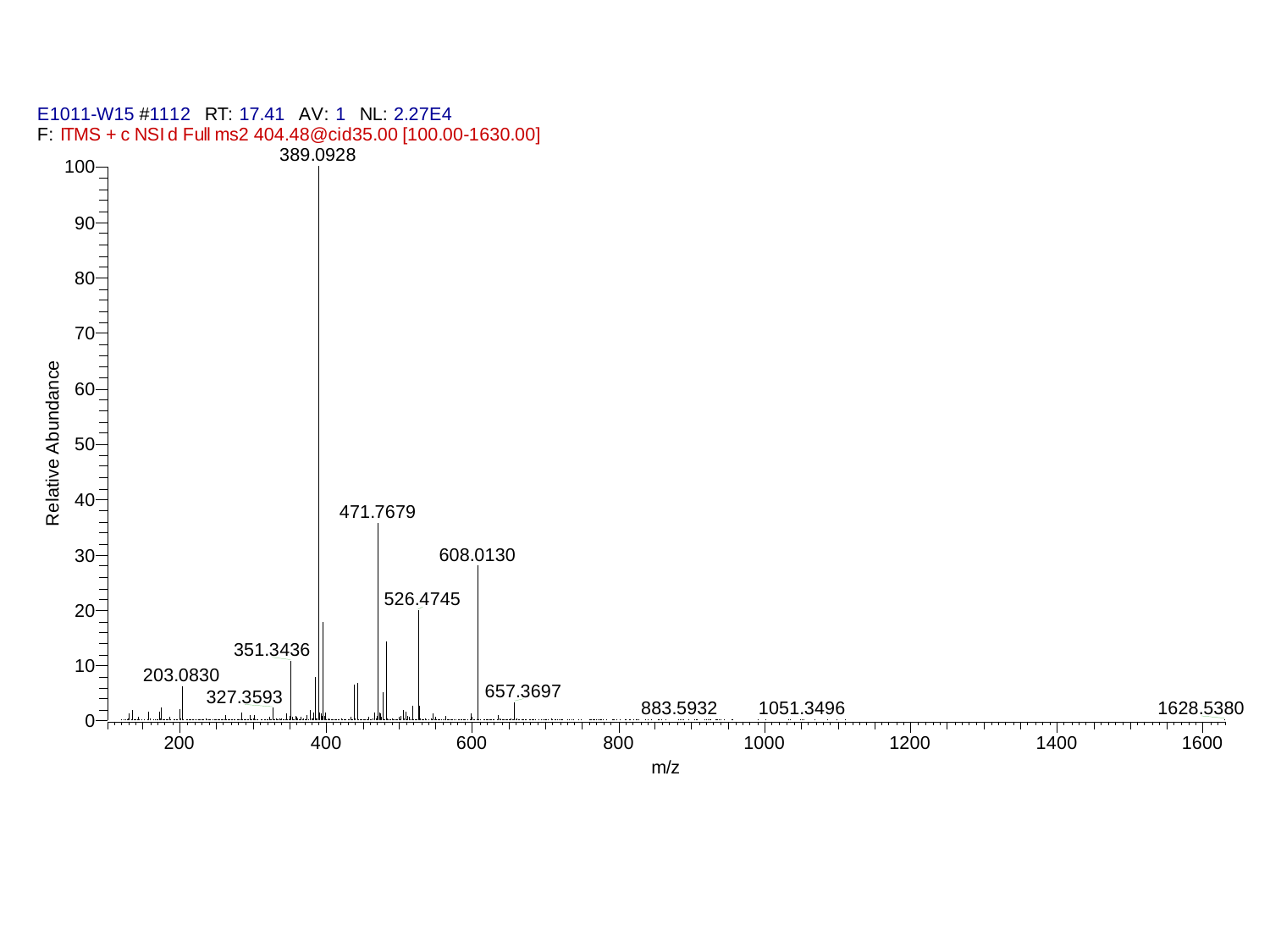

## Slide 134
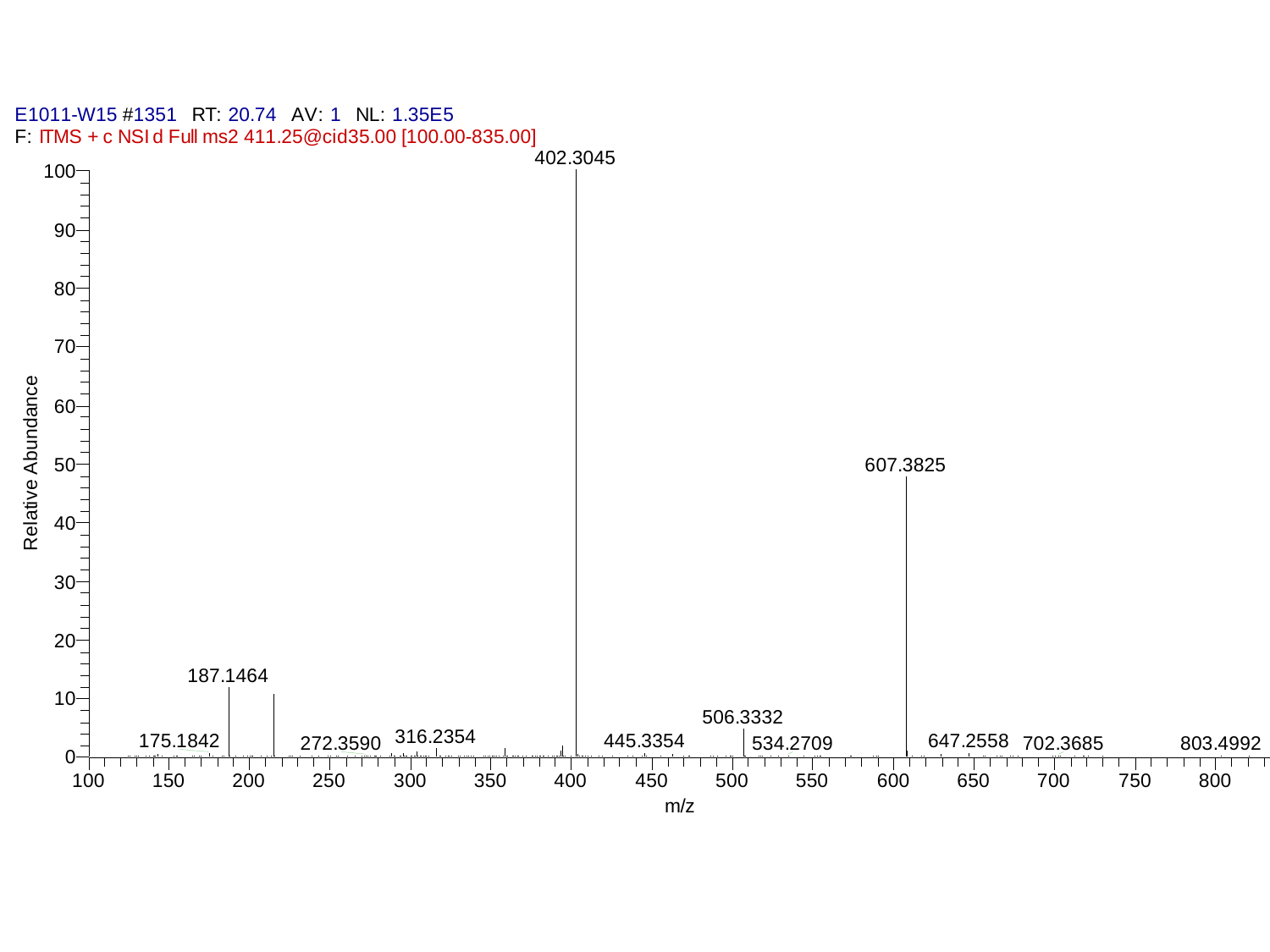

## Slide 135
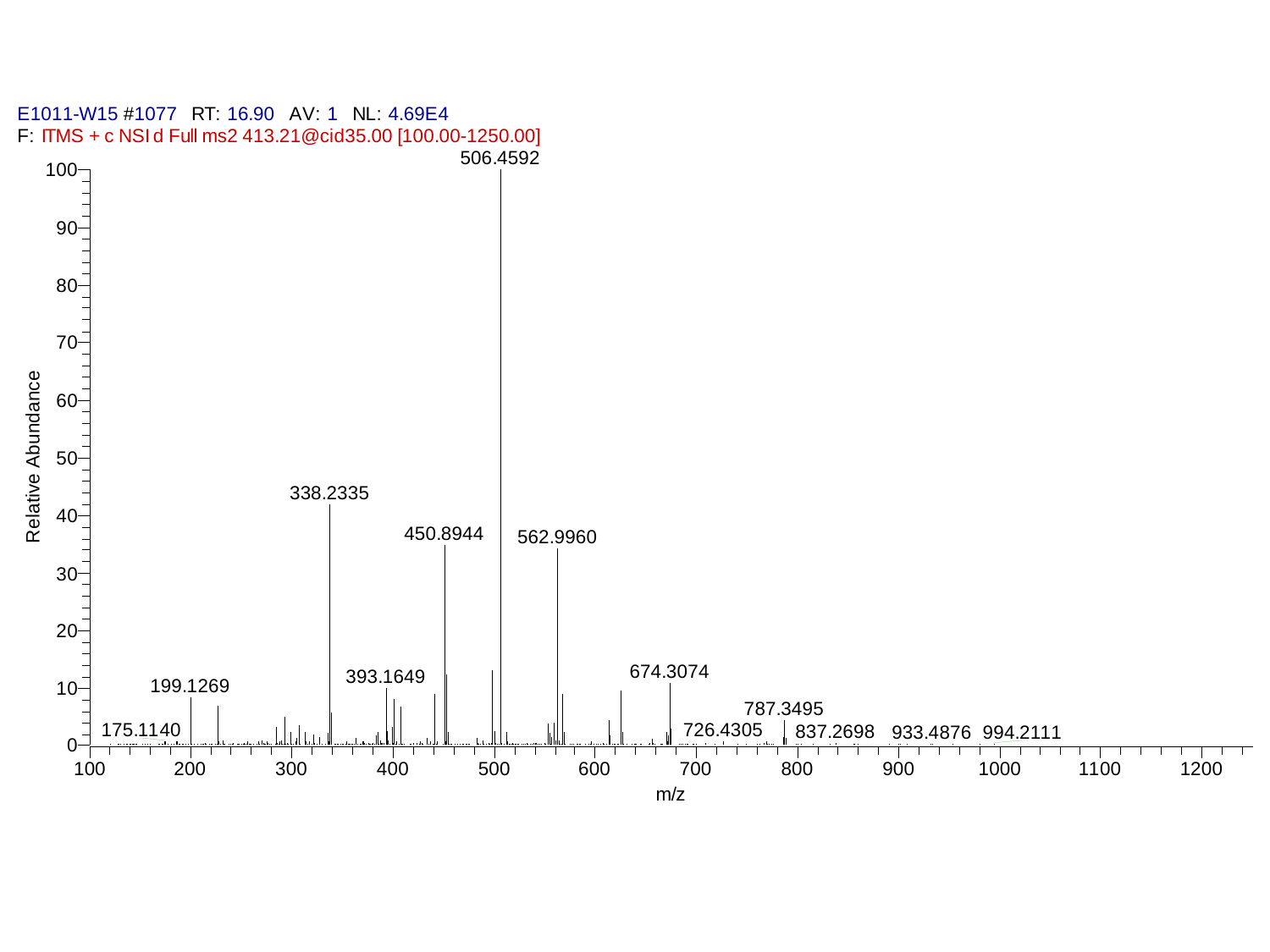

## Slide 136
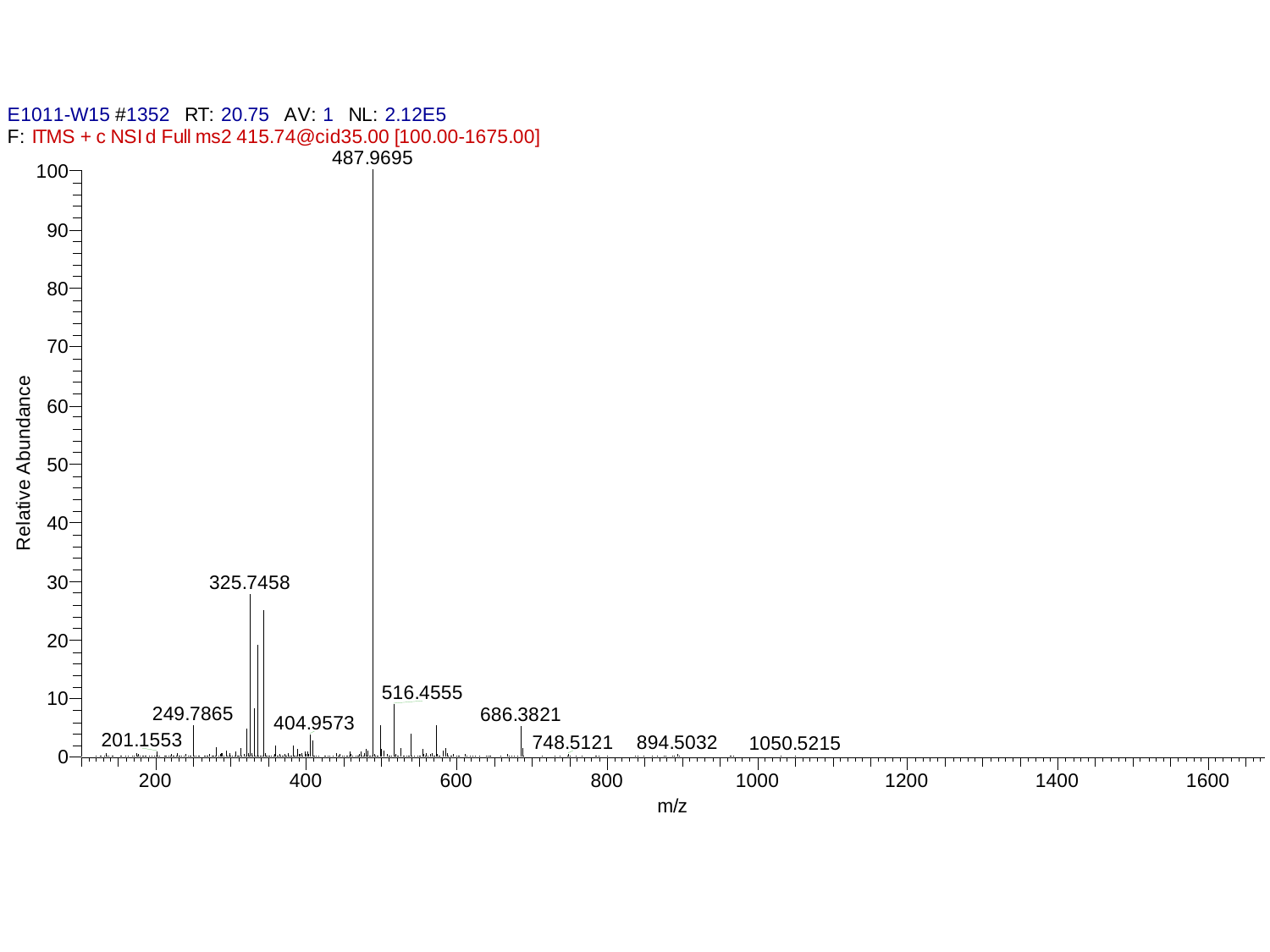

## Slide 137
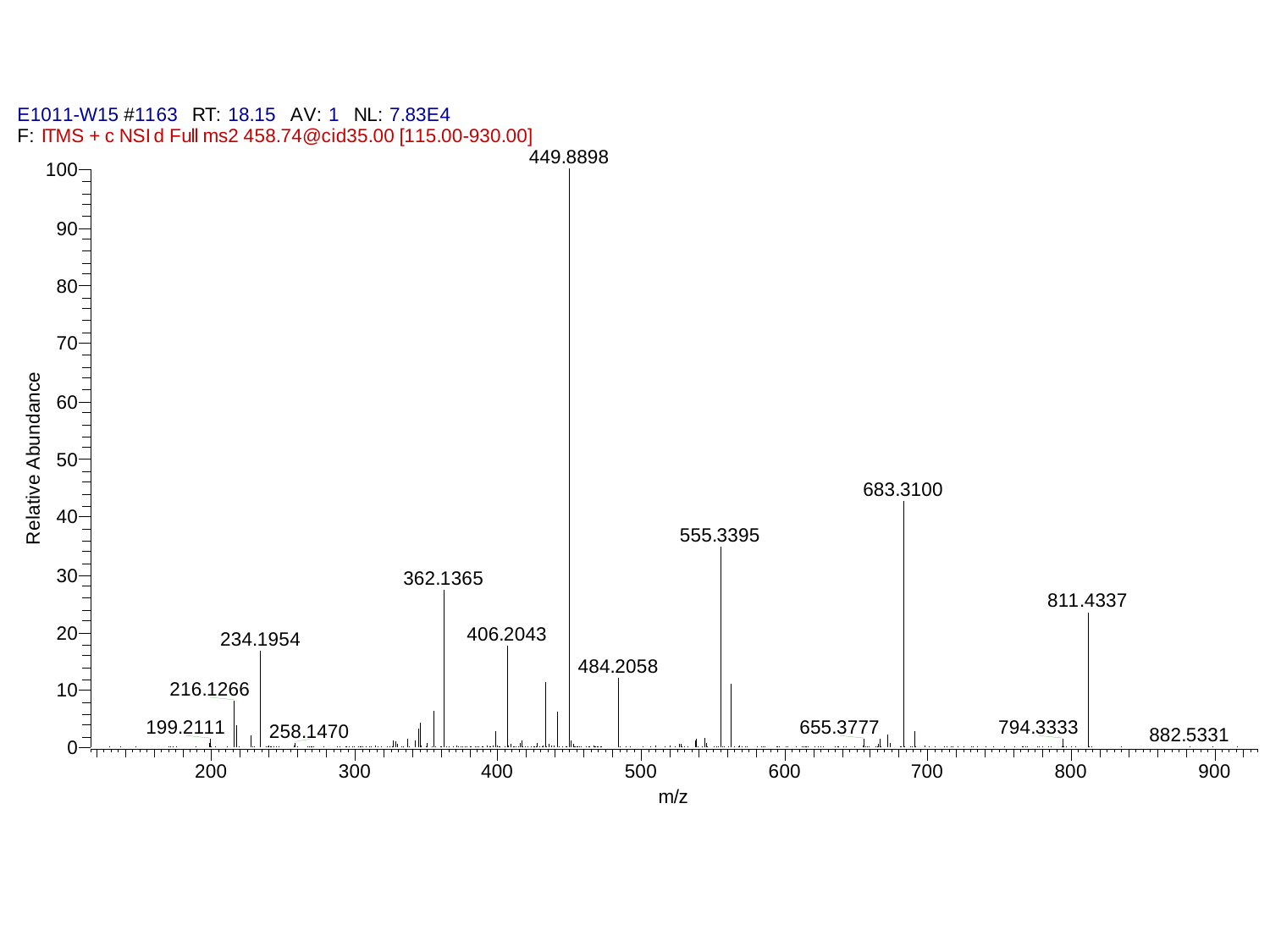

## Slide 138
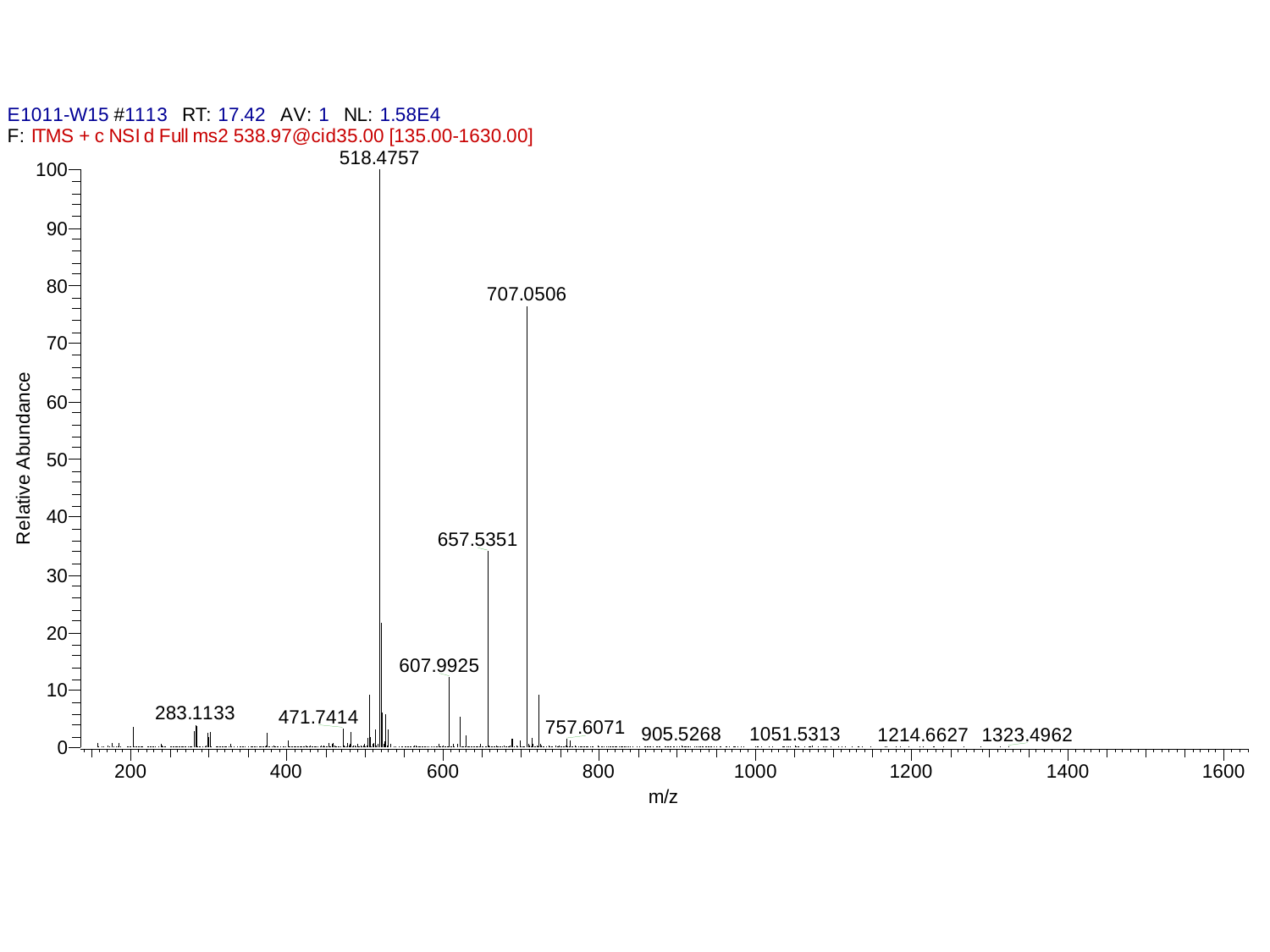

## Slide 139
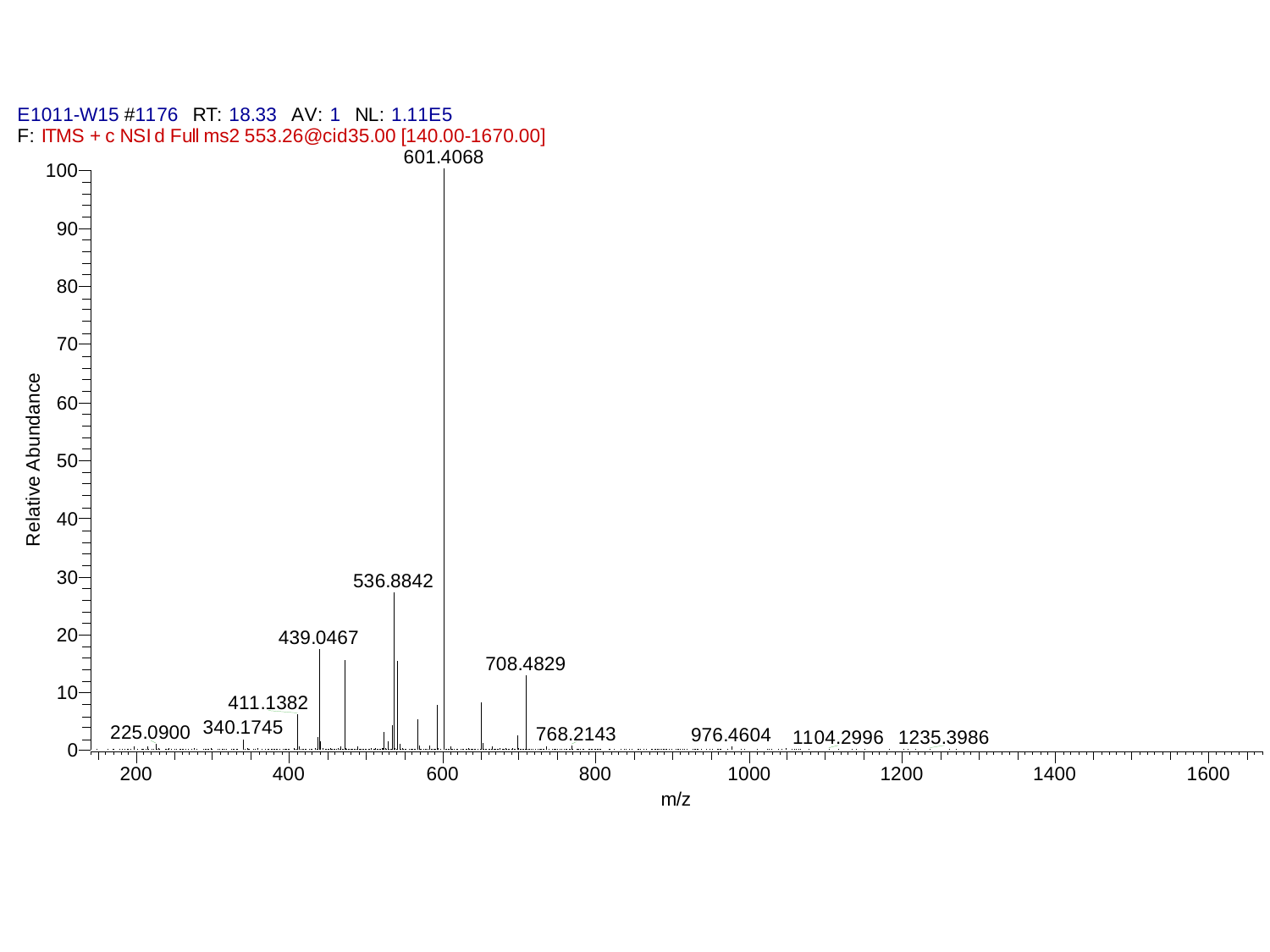

## Slide 140
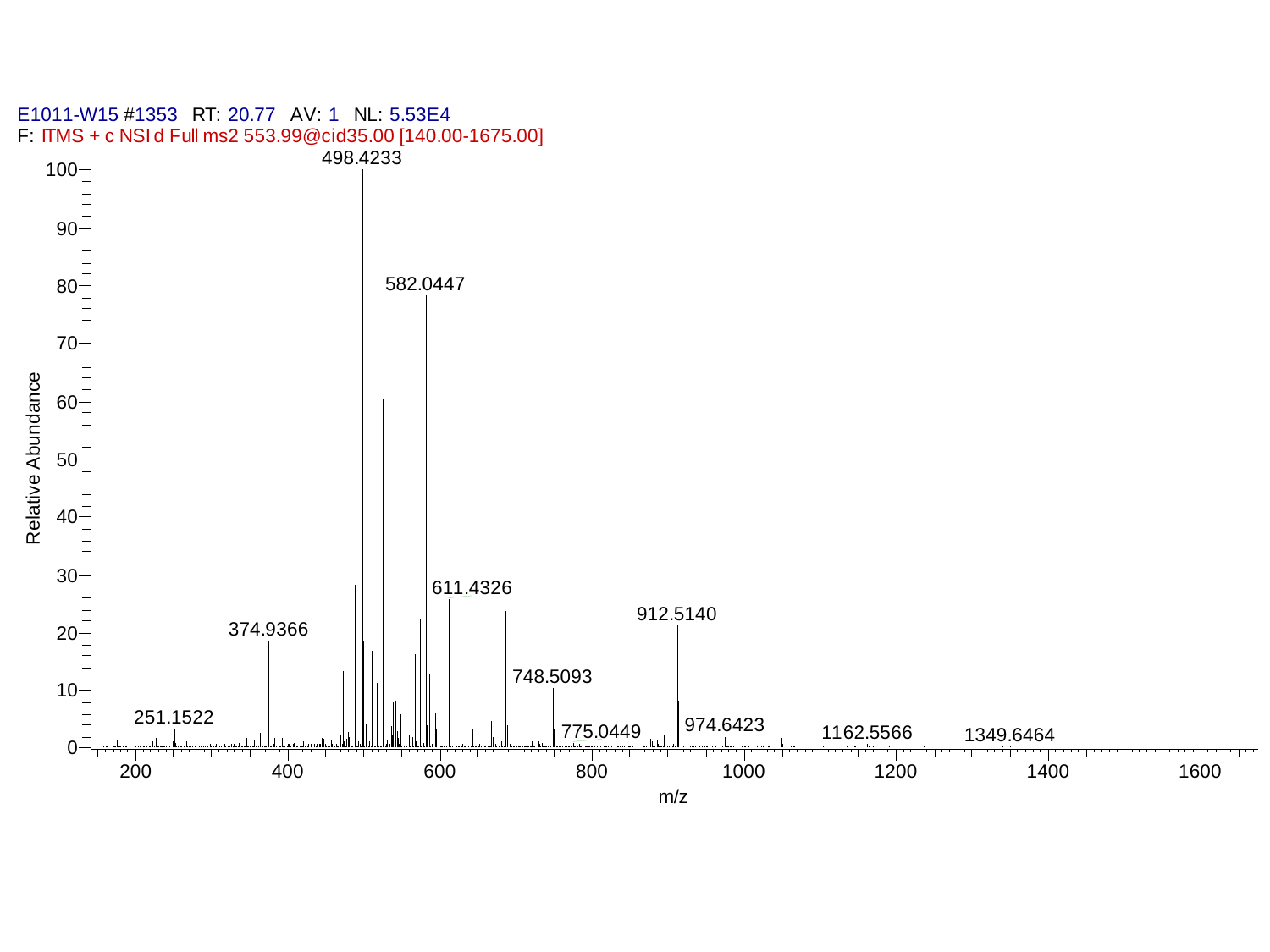

## Slide 141
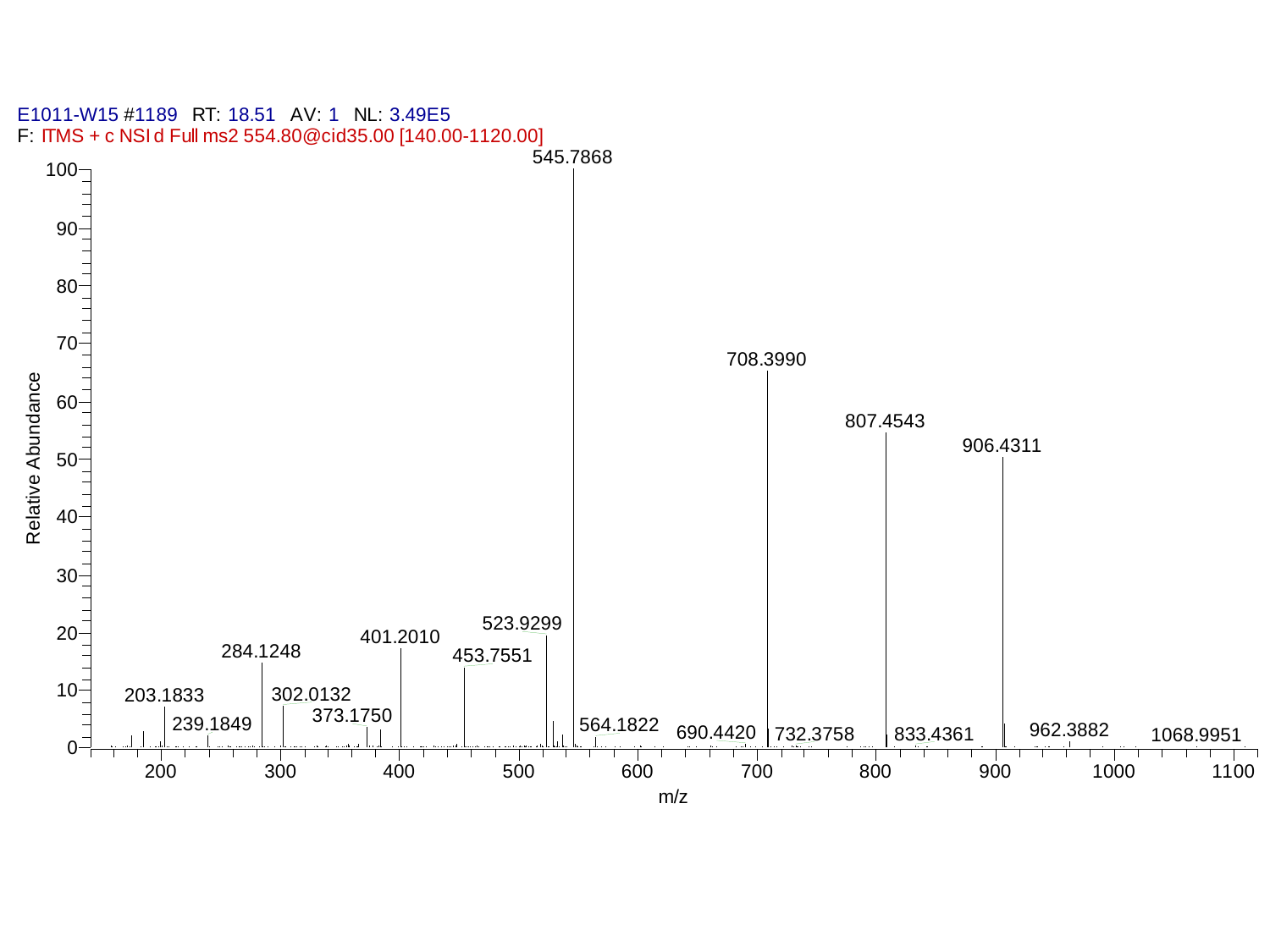

## Slide 142
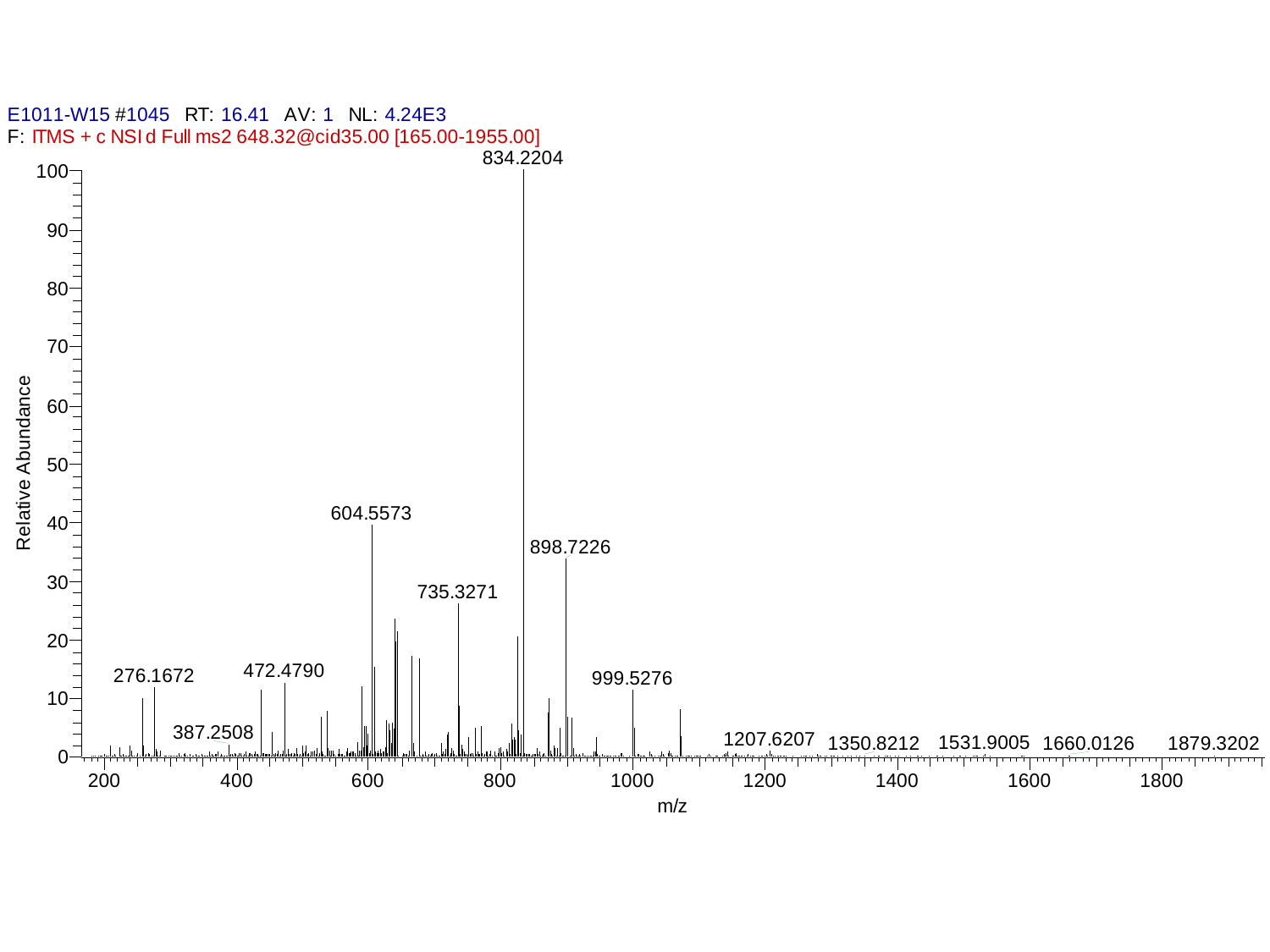

## Slide 143
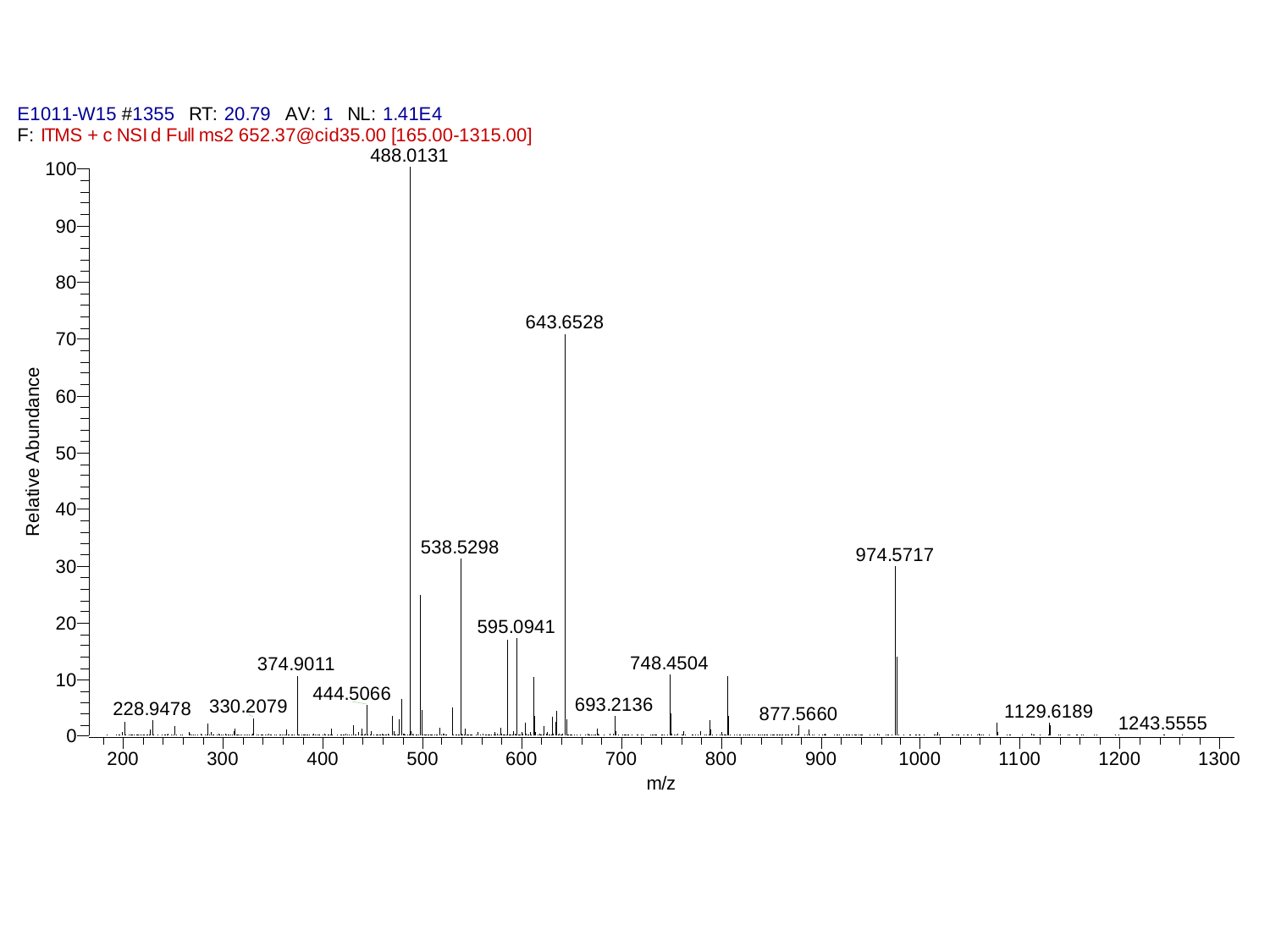

## Slide 144
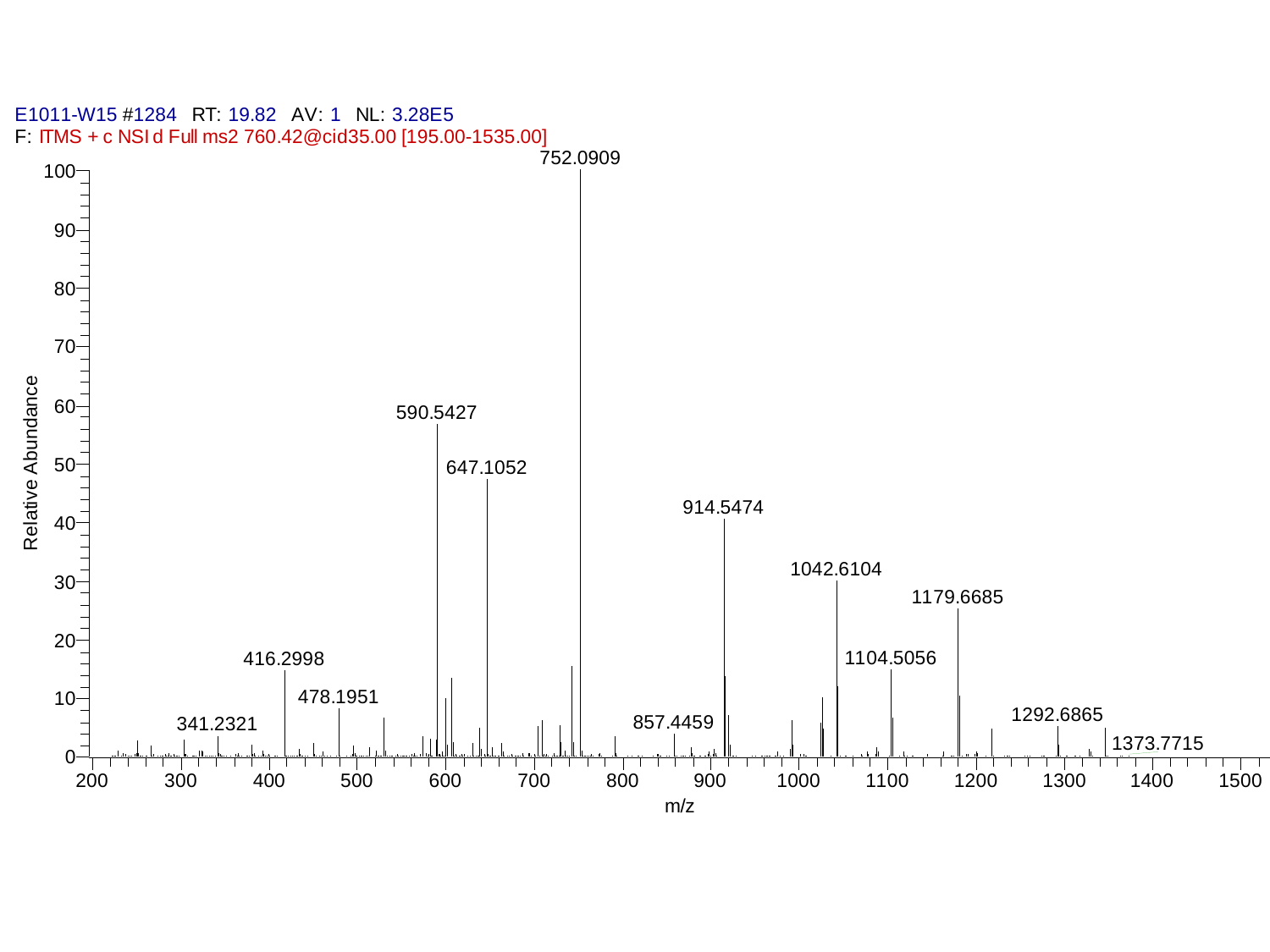

## Slide 145
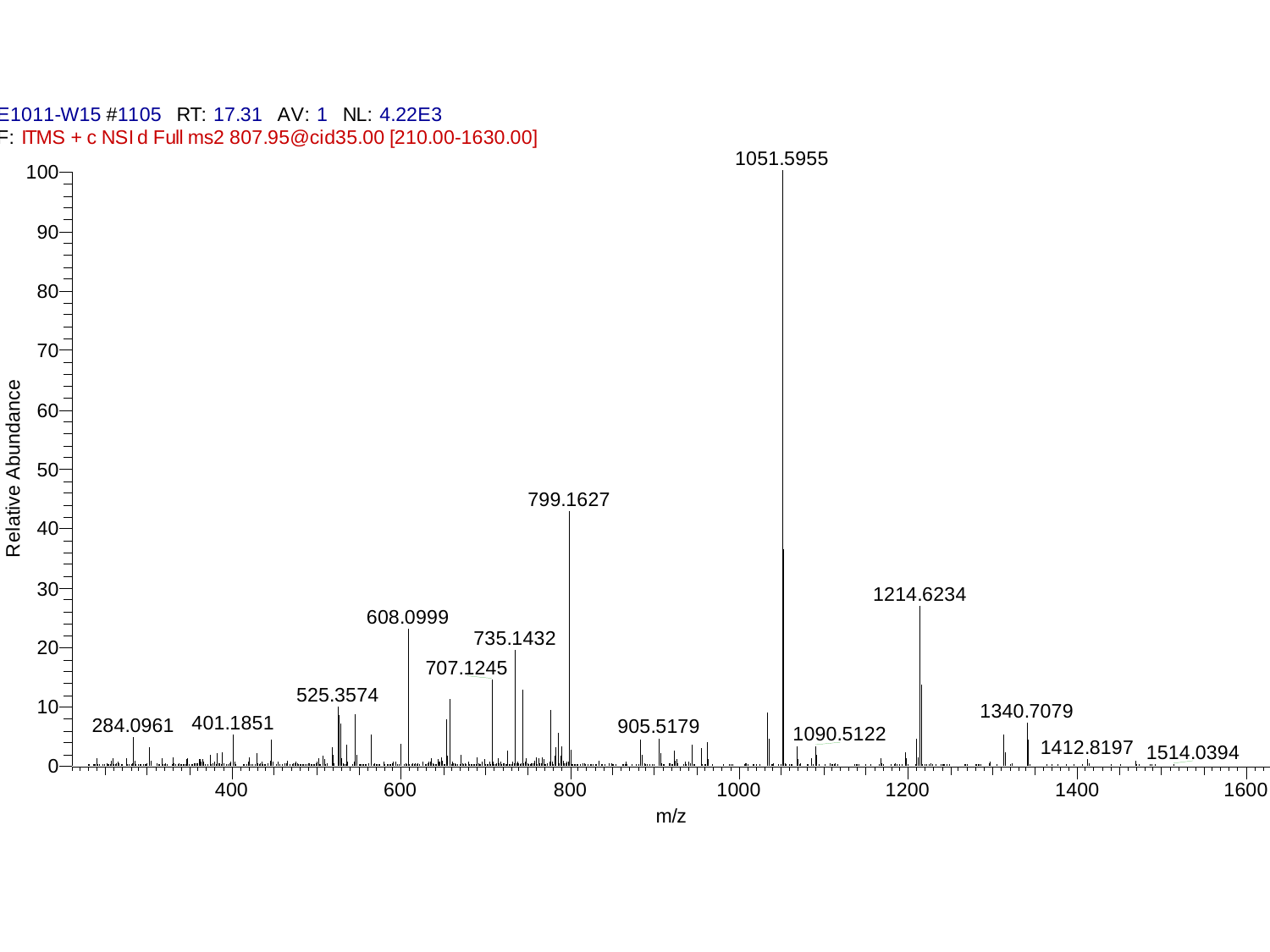

## Slide 146
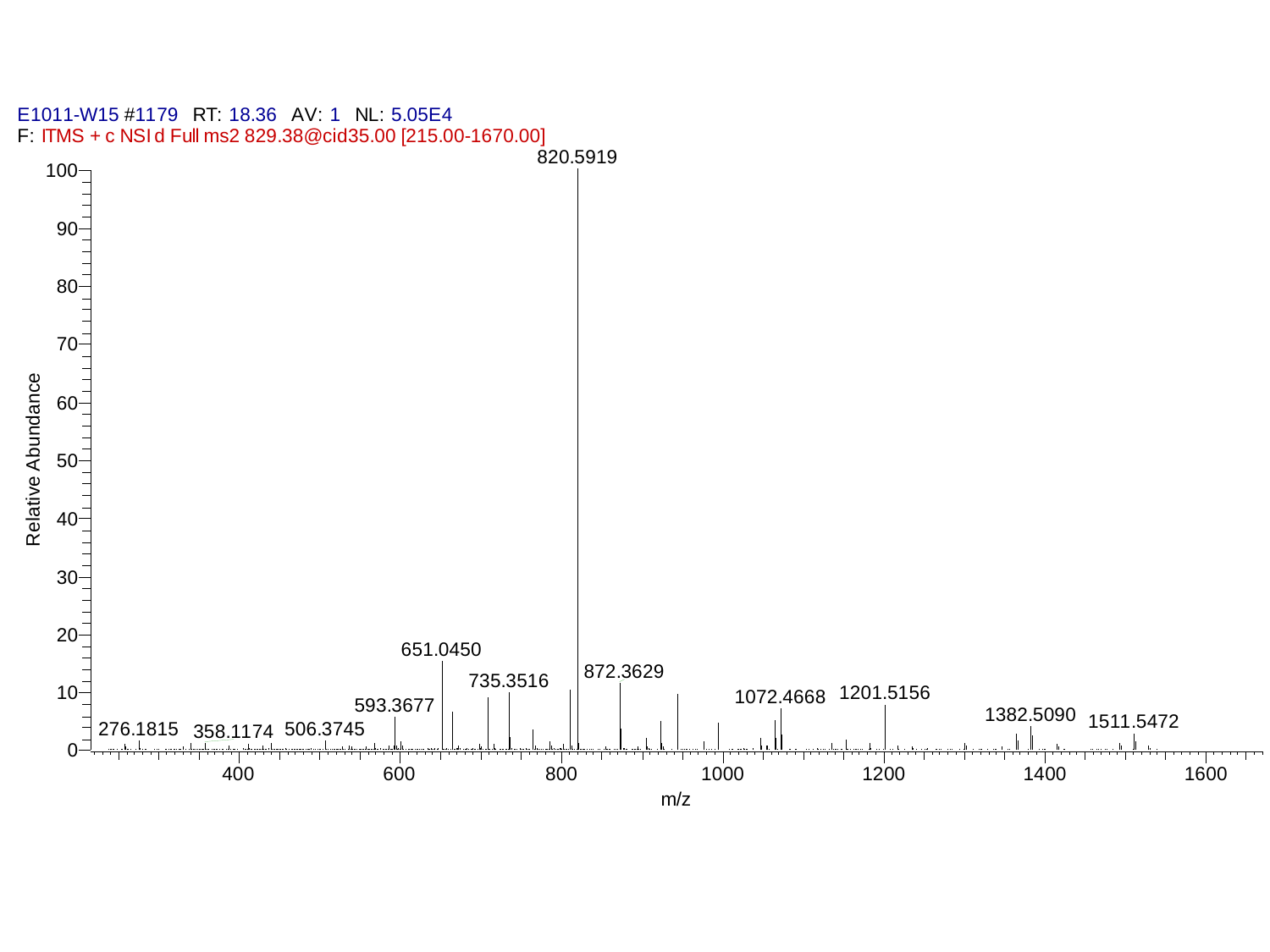

## Slide 147
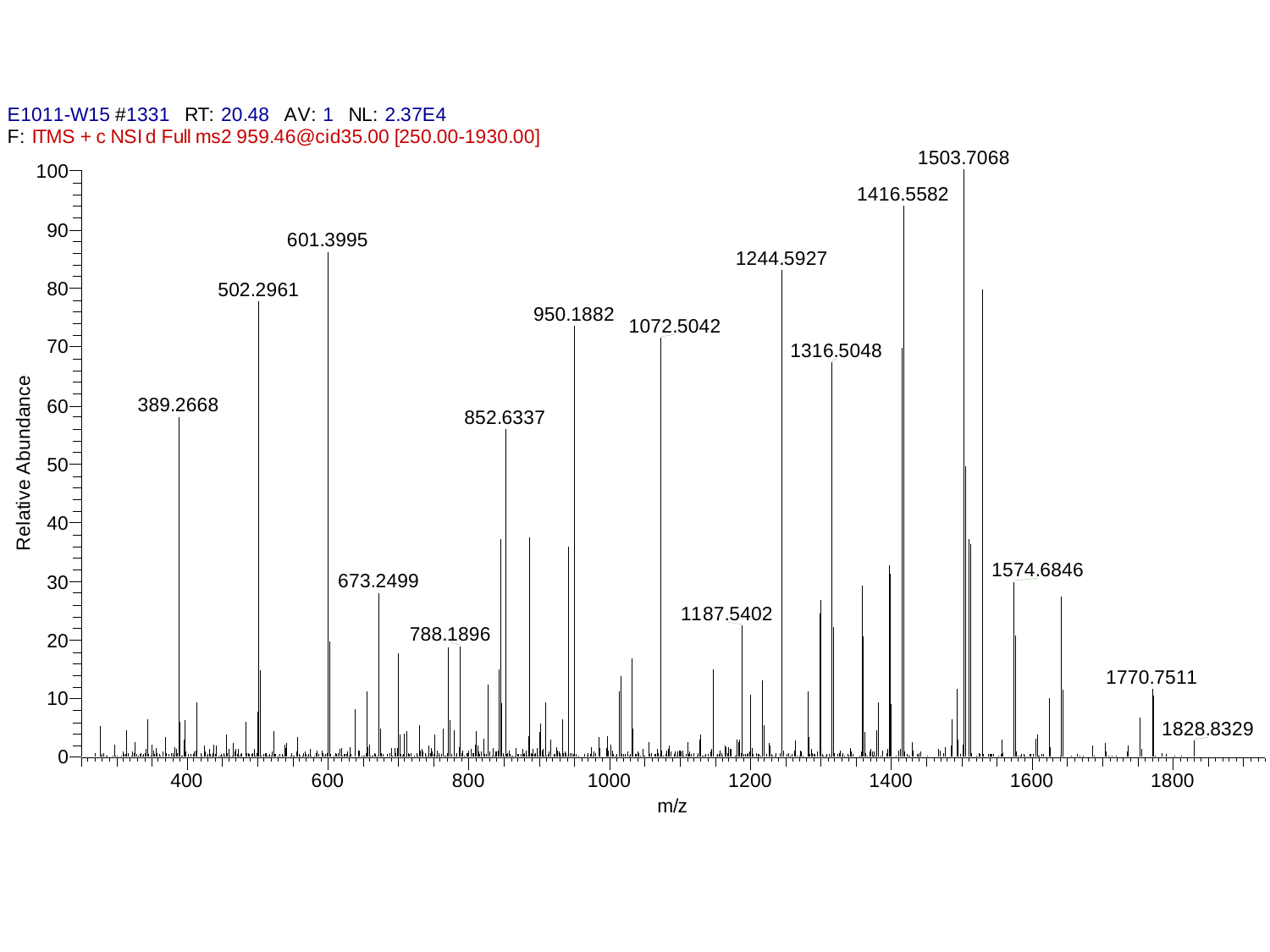

## Slide 148
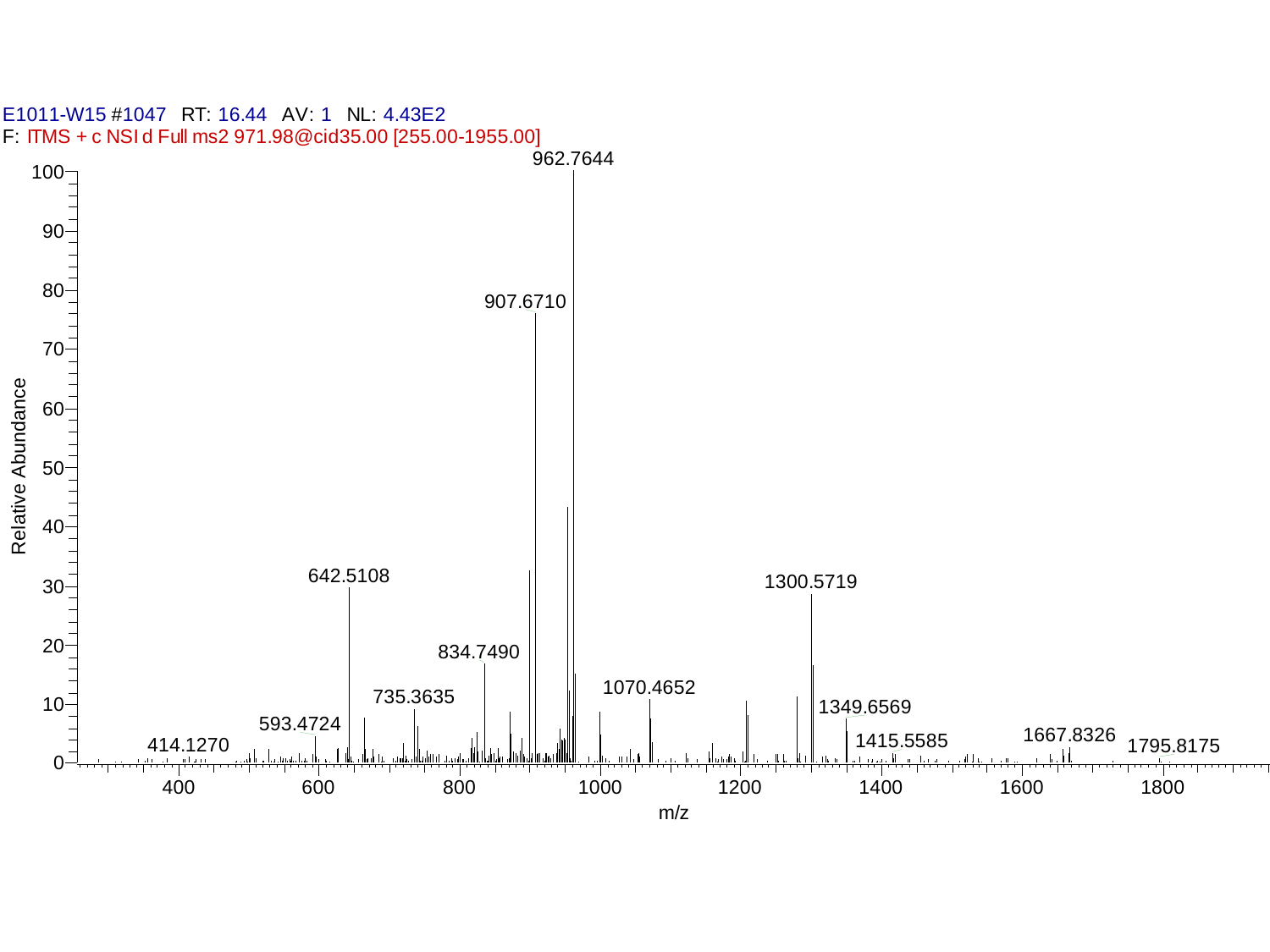

## Slide 149
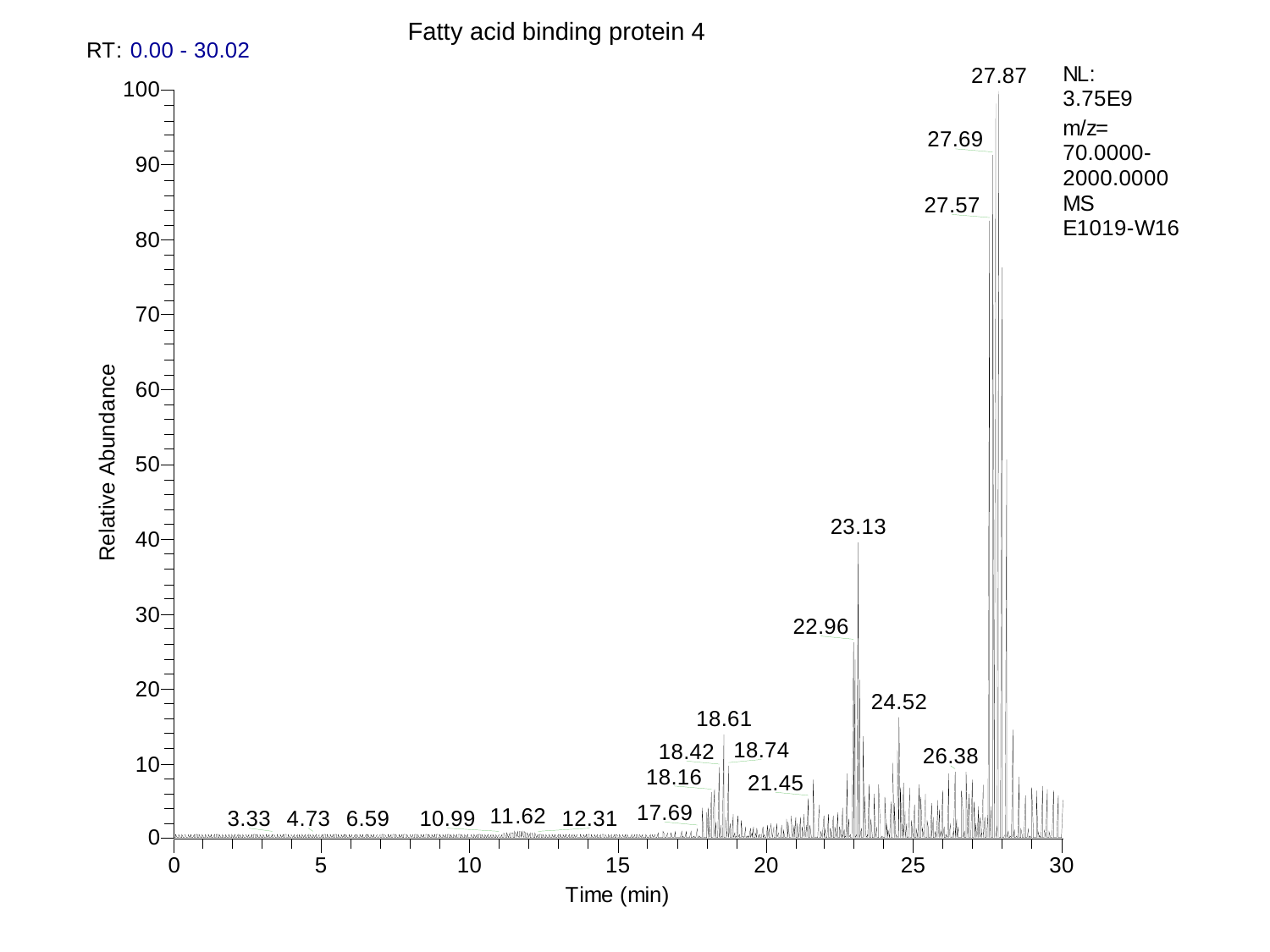

Fatty acid binding protein 4

## Slide 150
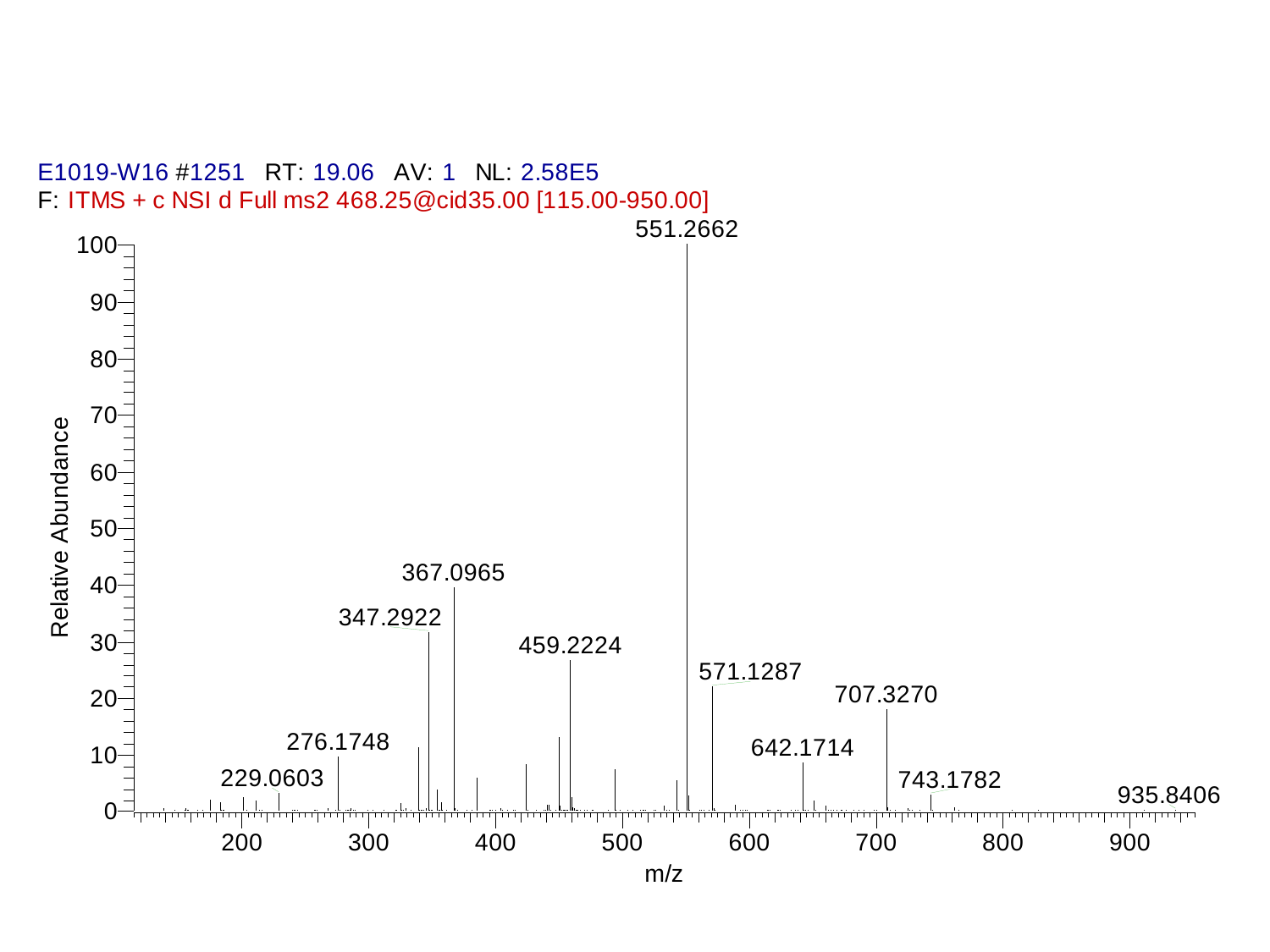

## Slide 151
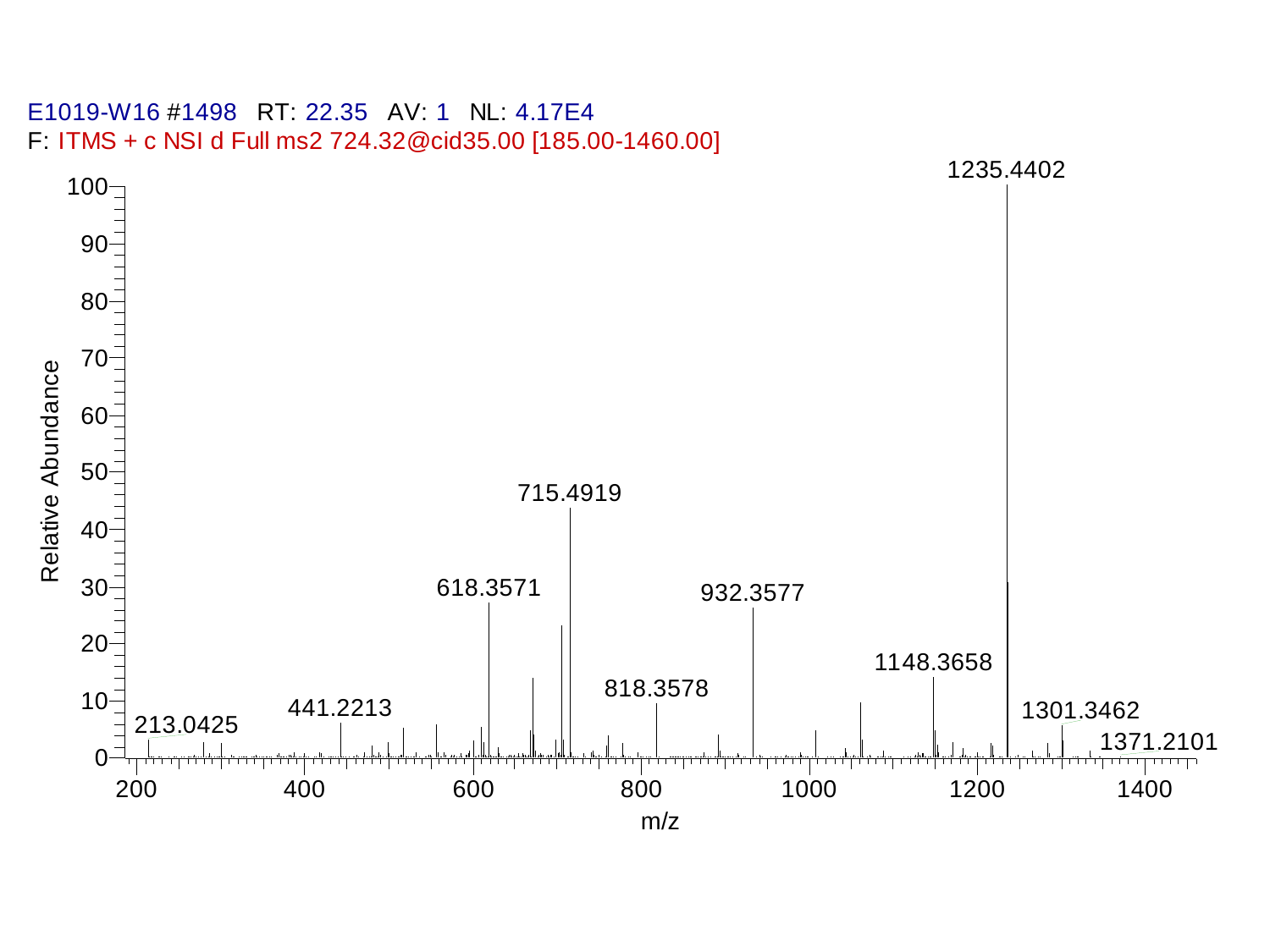

## Slide 152
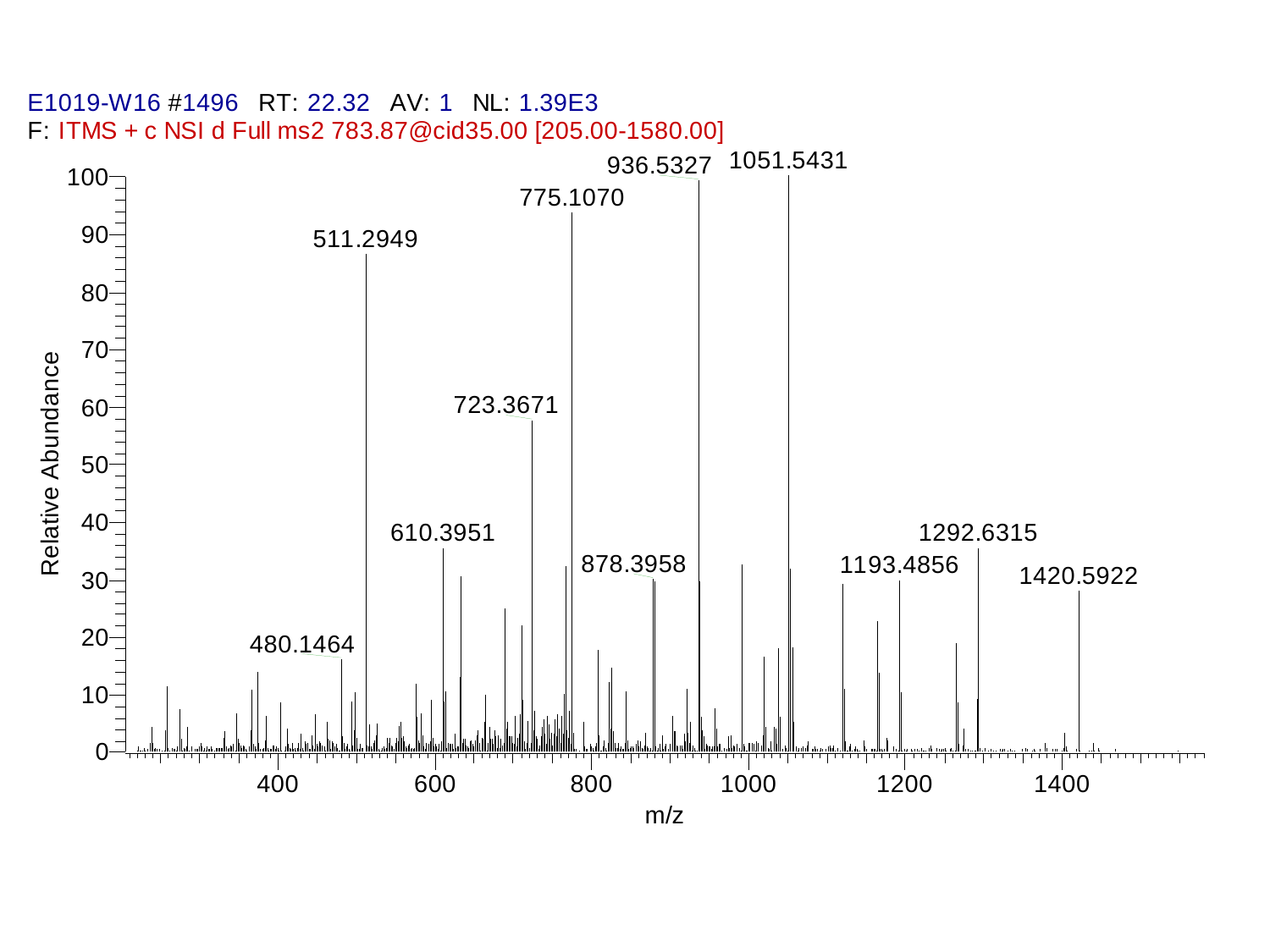

## Slide 153
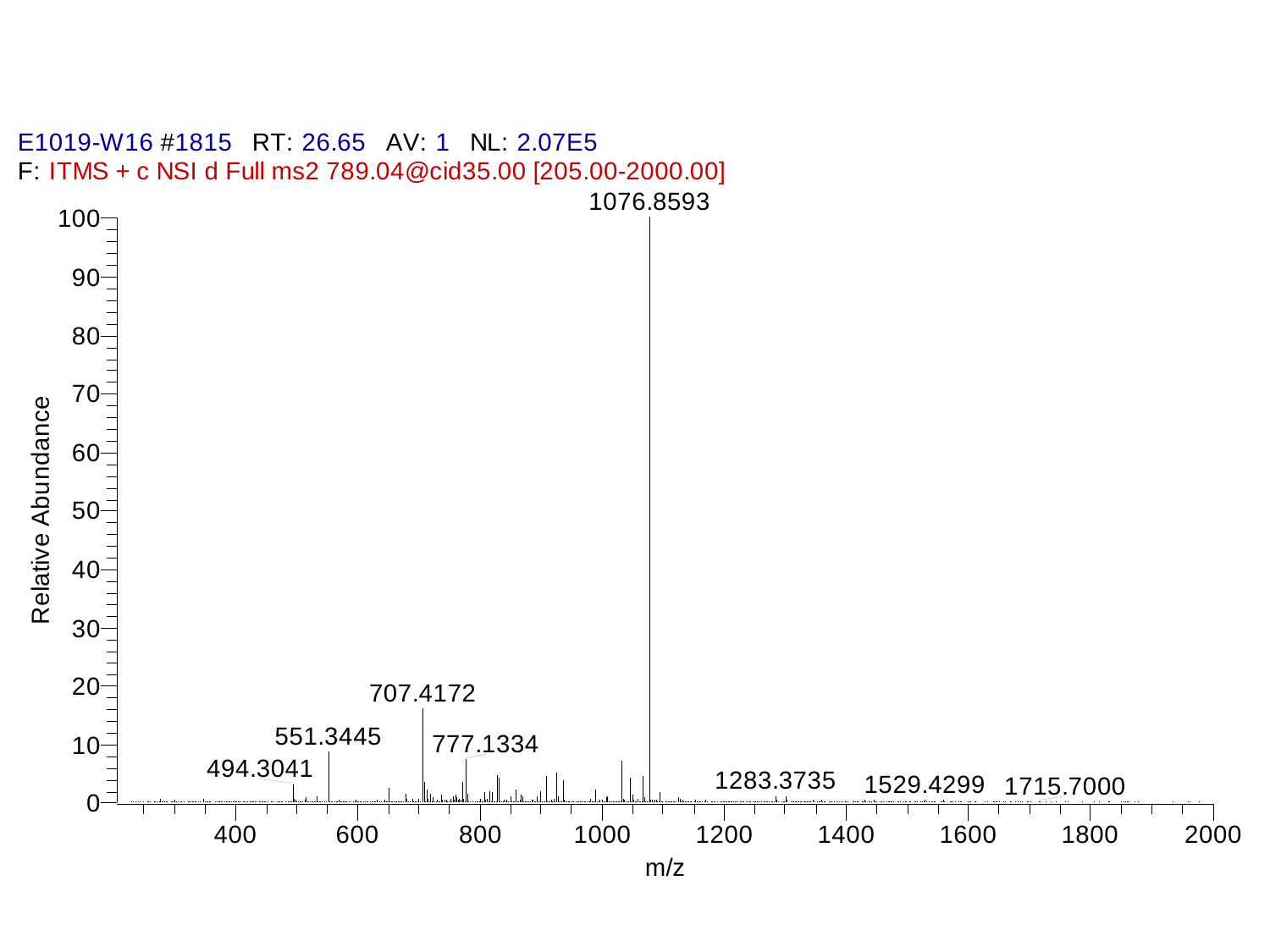

## Slide 154
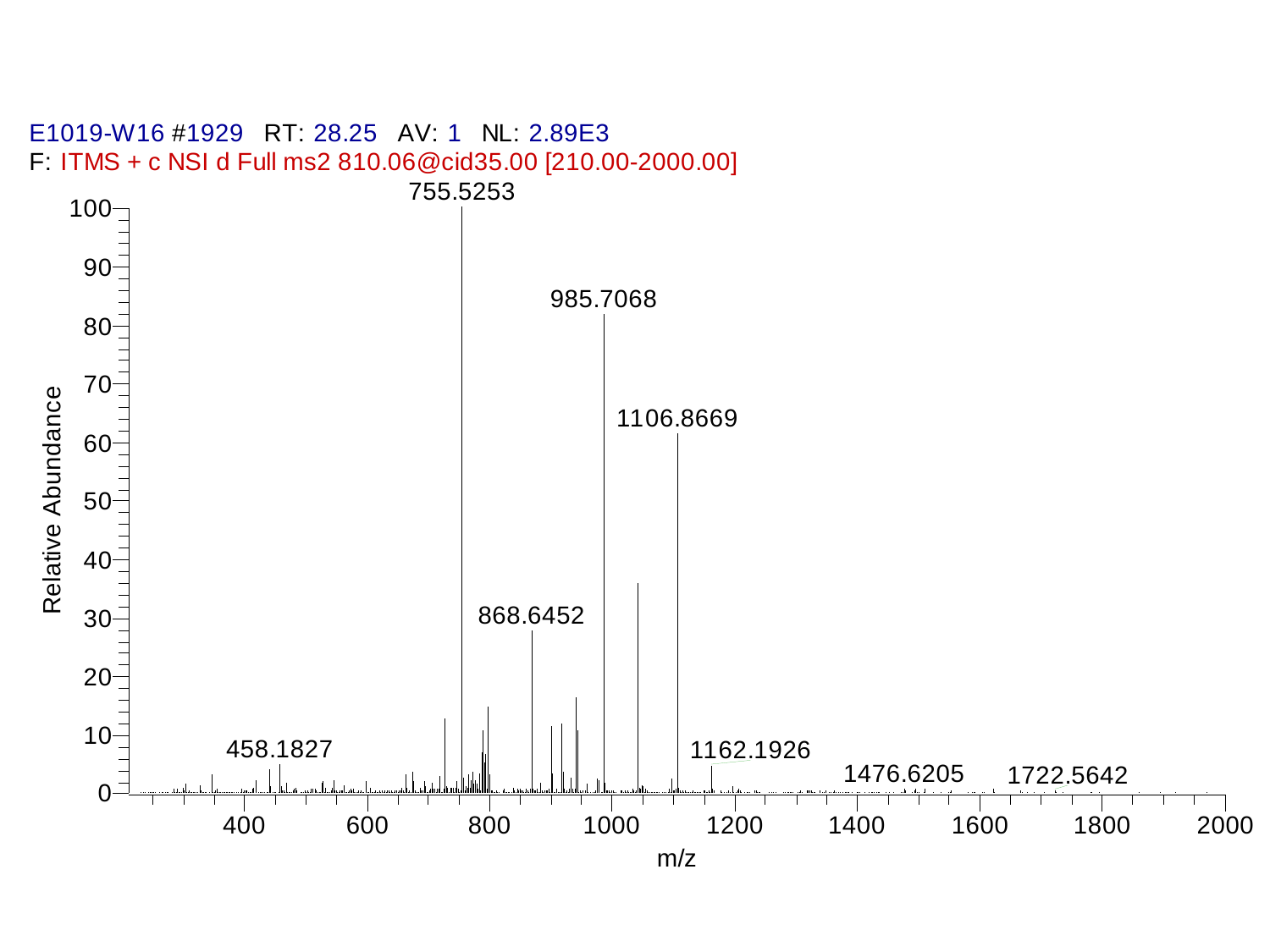

## Slide 155
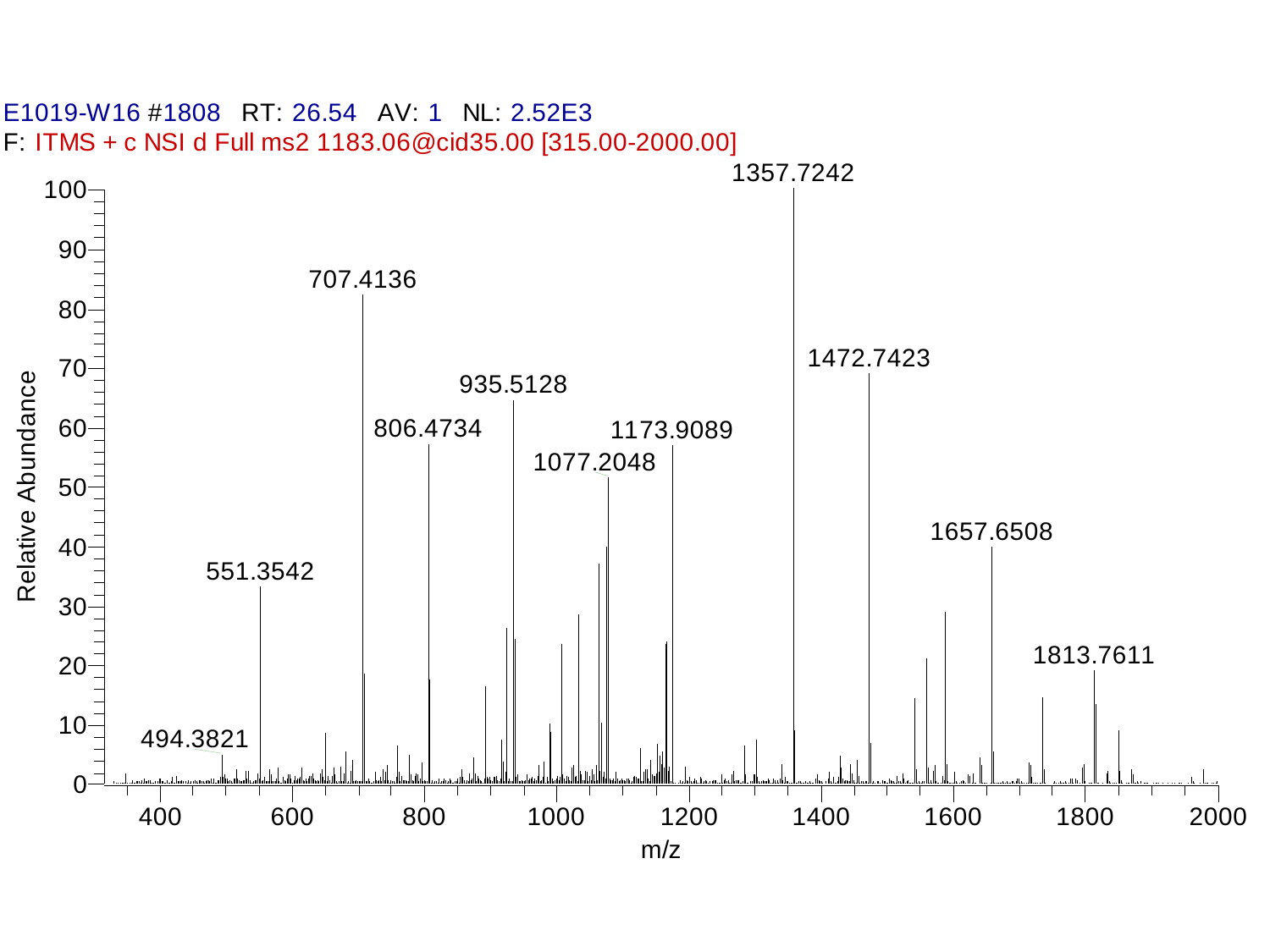

## Slide 156
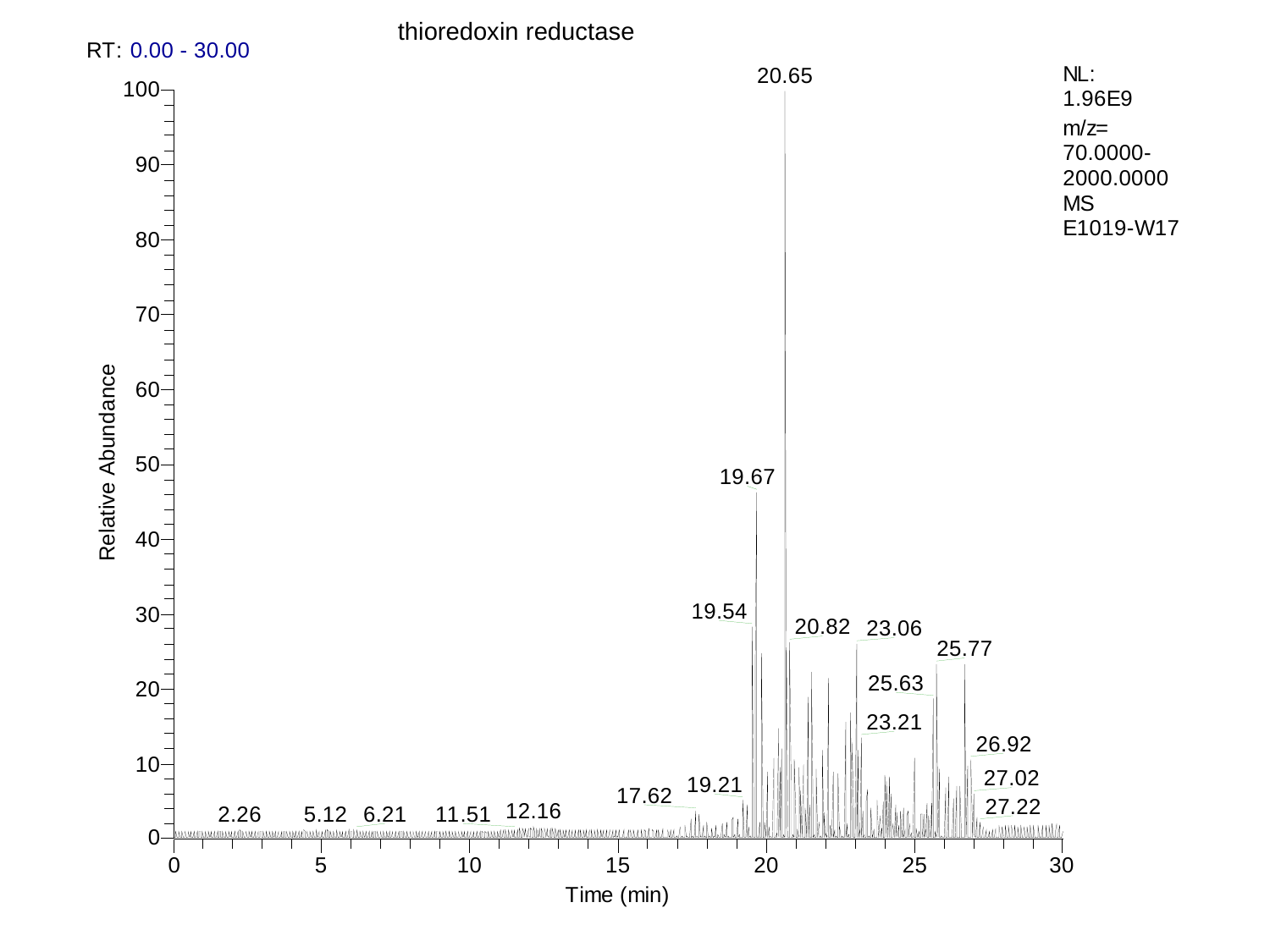

thioredoxin reductase

## Slide 157
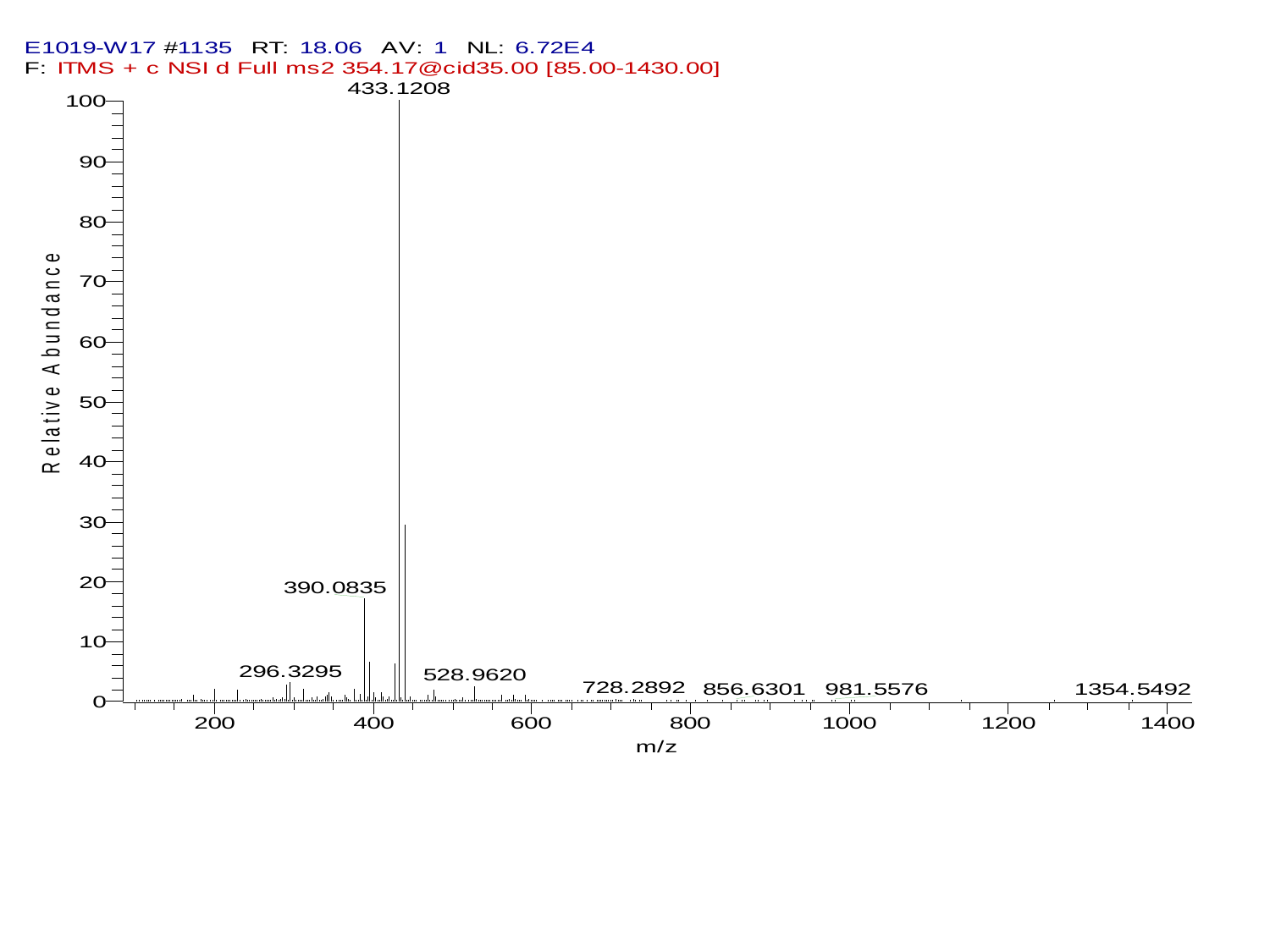

## Slide 158
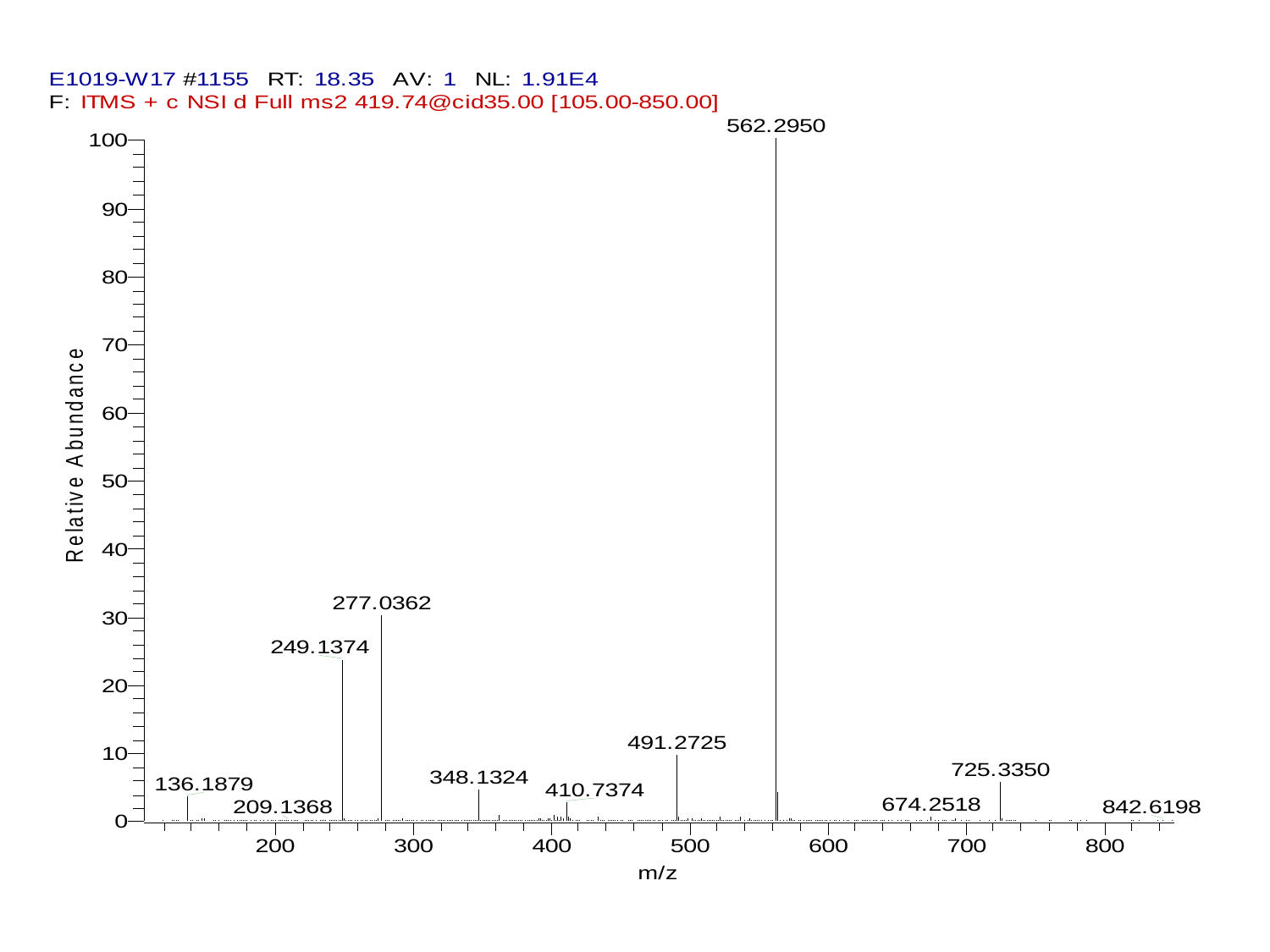

## Slide 159
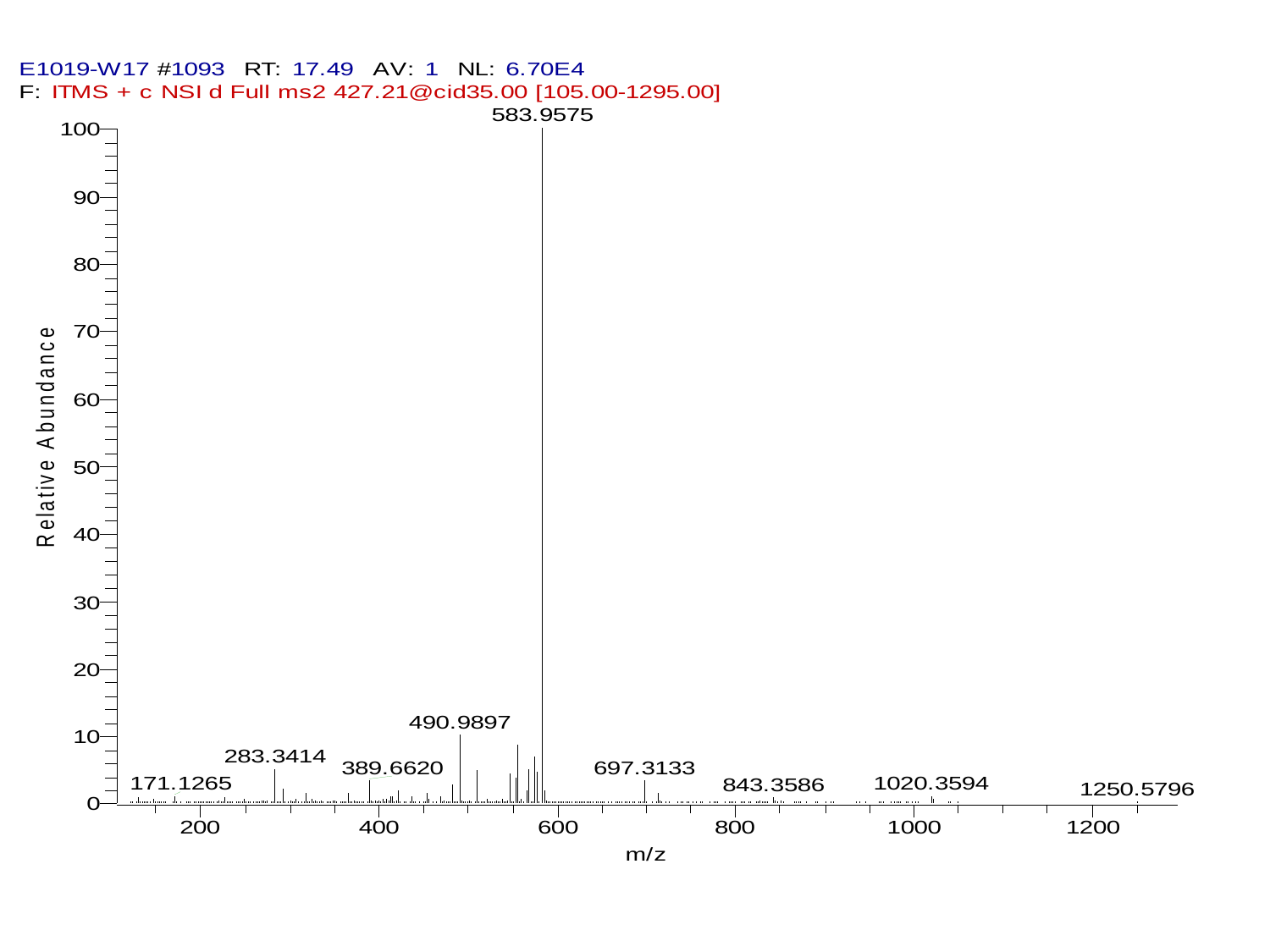

## Slide 160
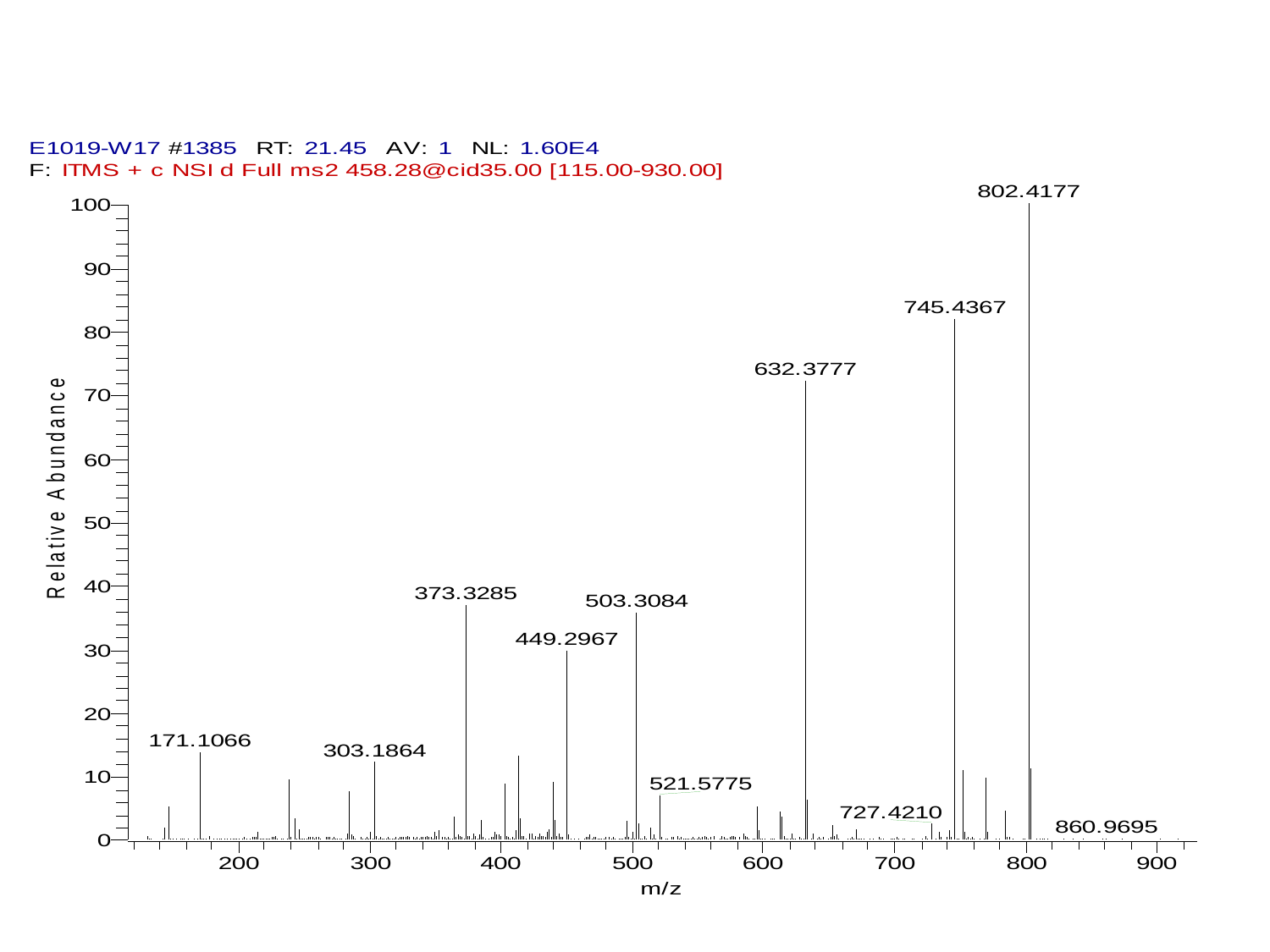

## Slide 161
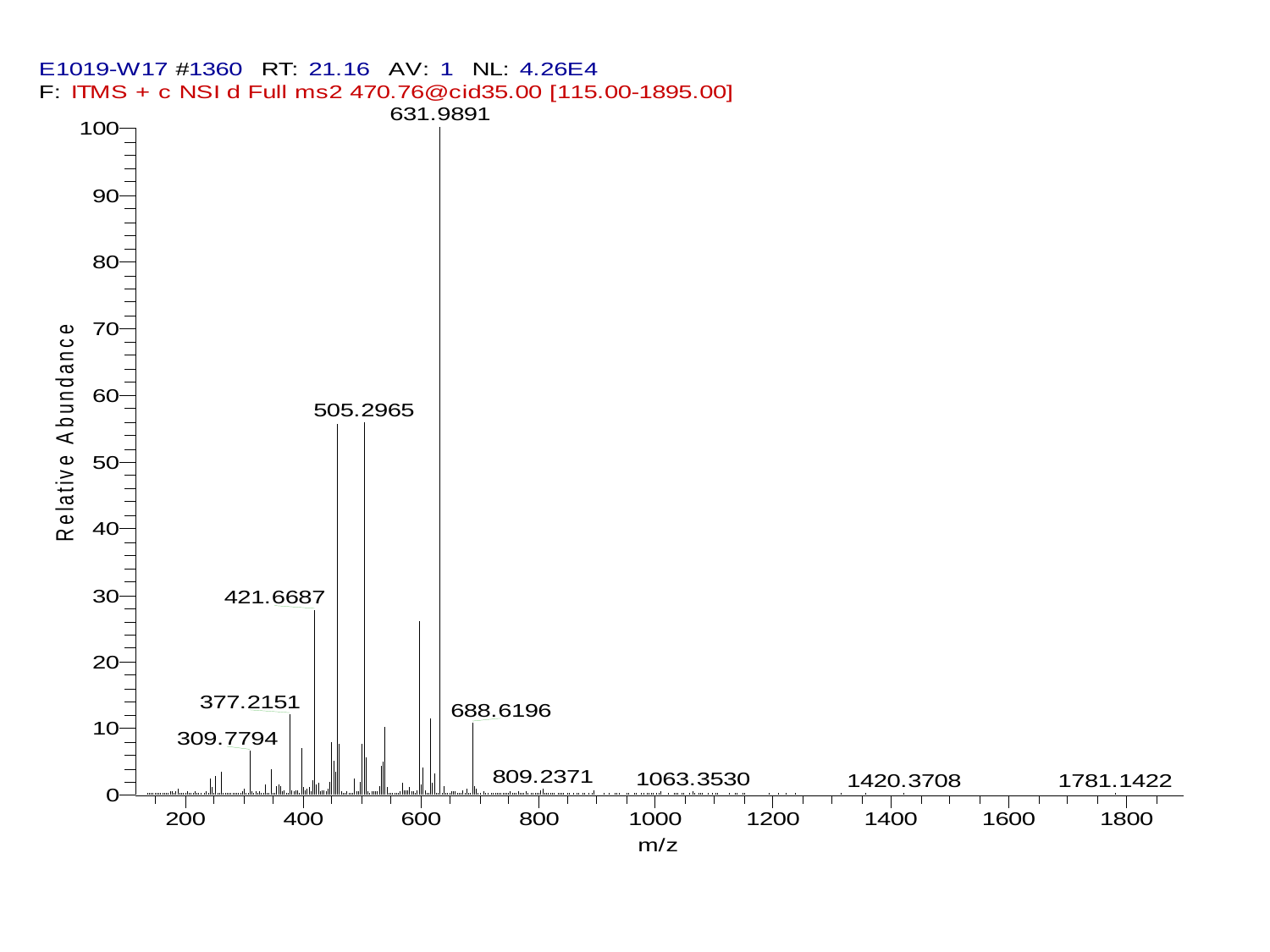

## Slide 162
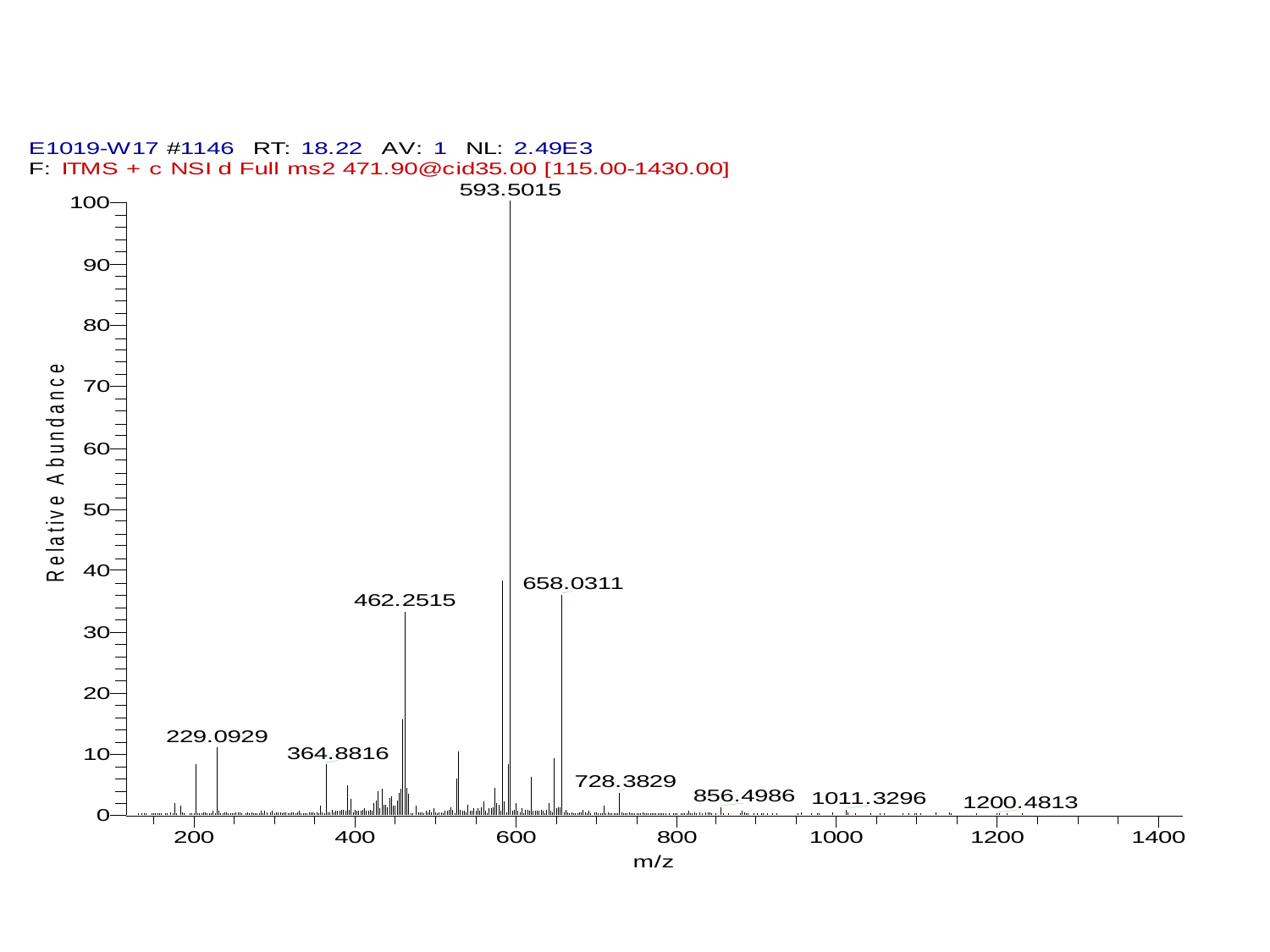

## Slide 163
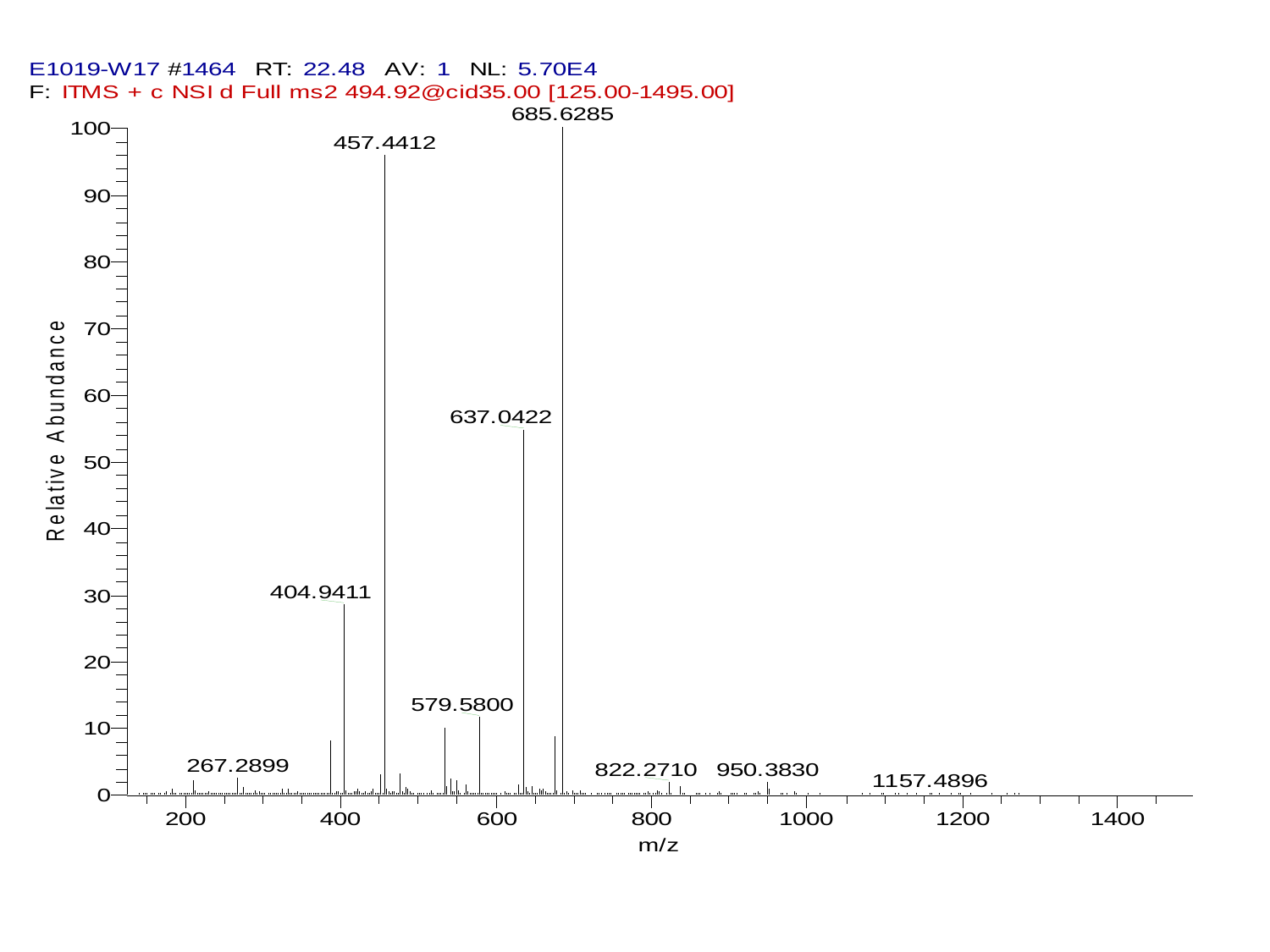

## Slide 164
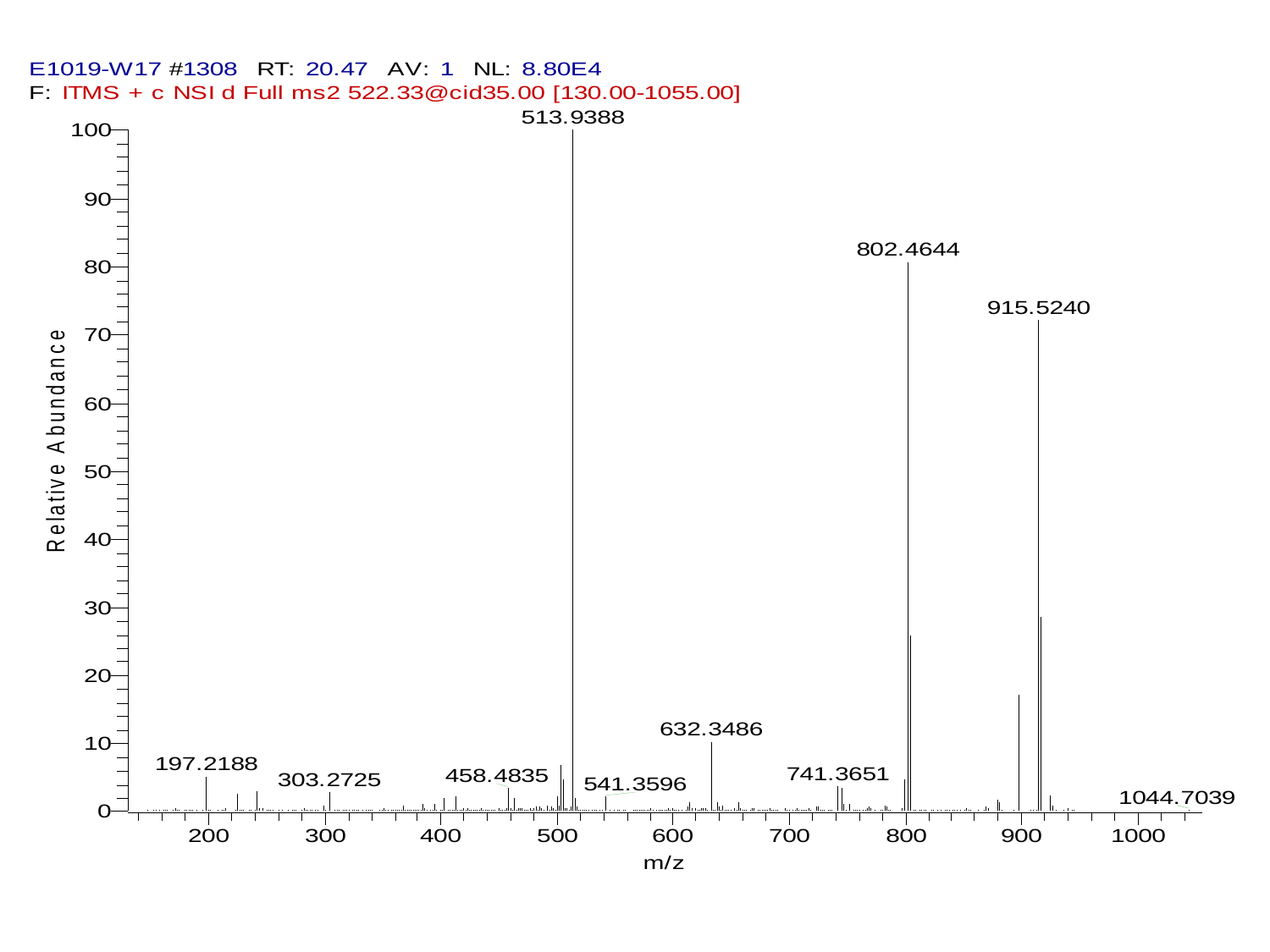

## Slide 165
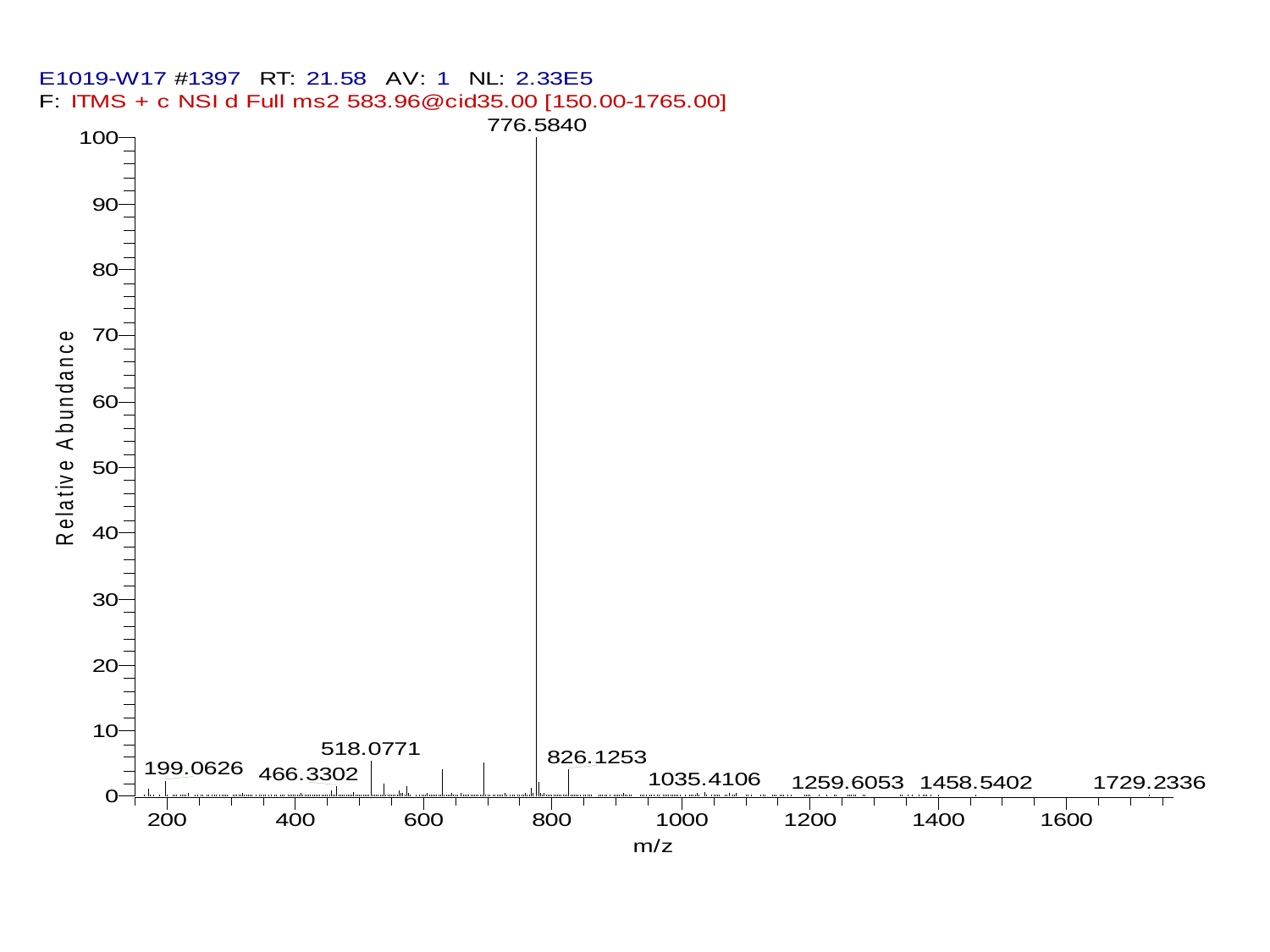

## Slide 166
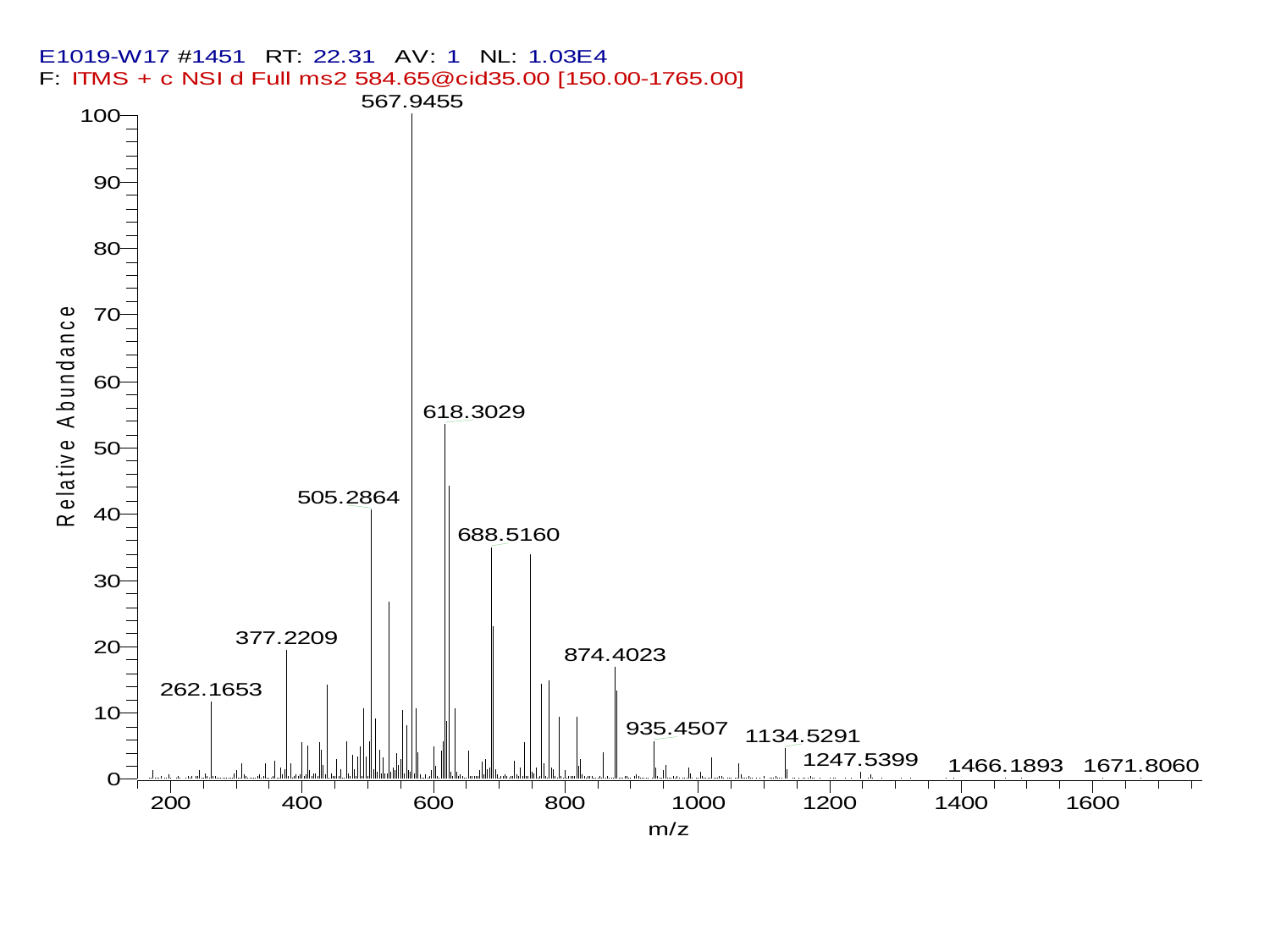

## Slide 167
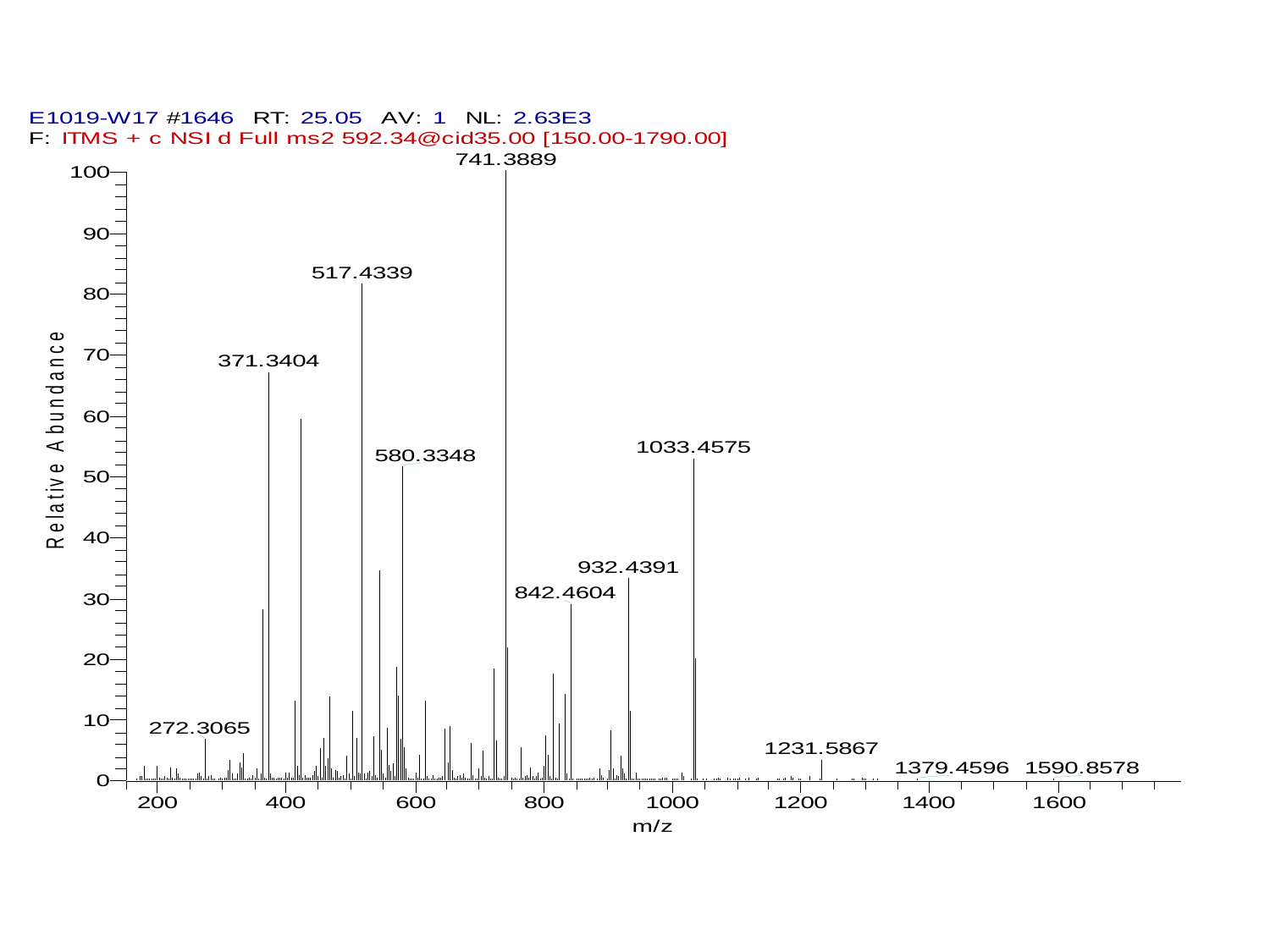

## Slide 168
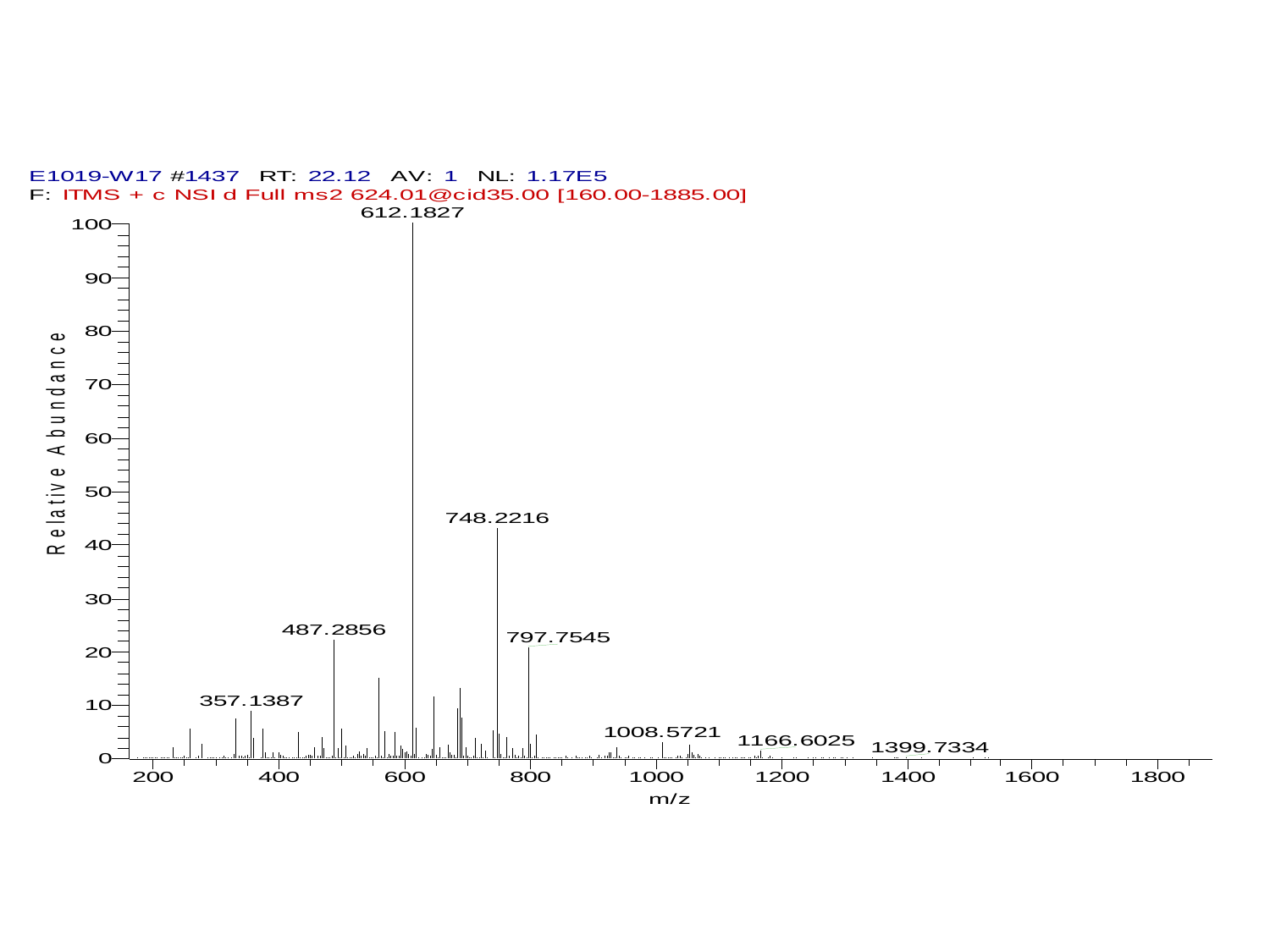

## Slide 169
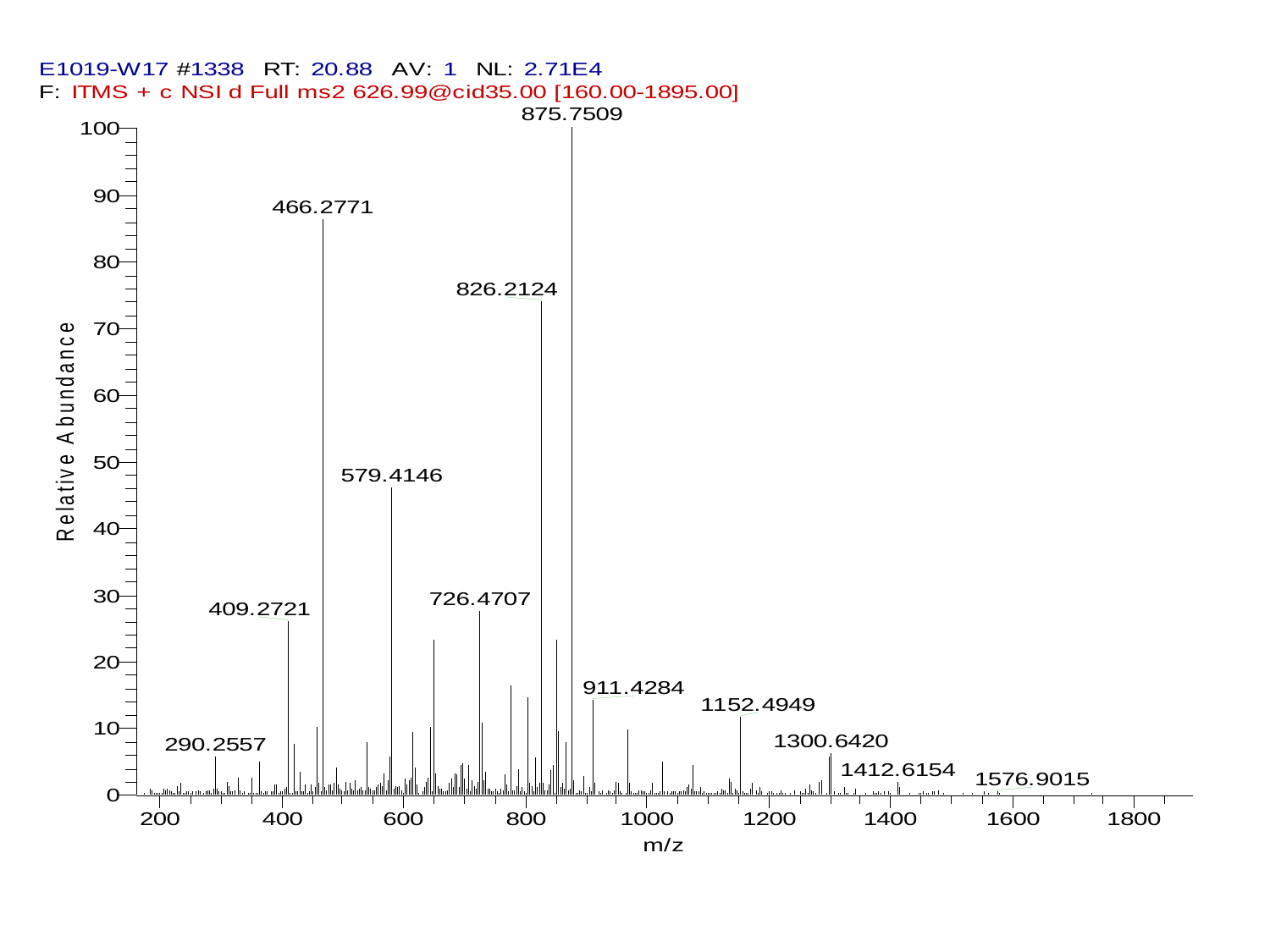

## Slide 170
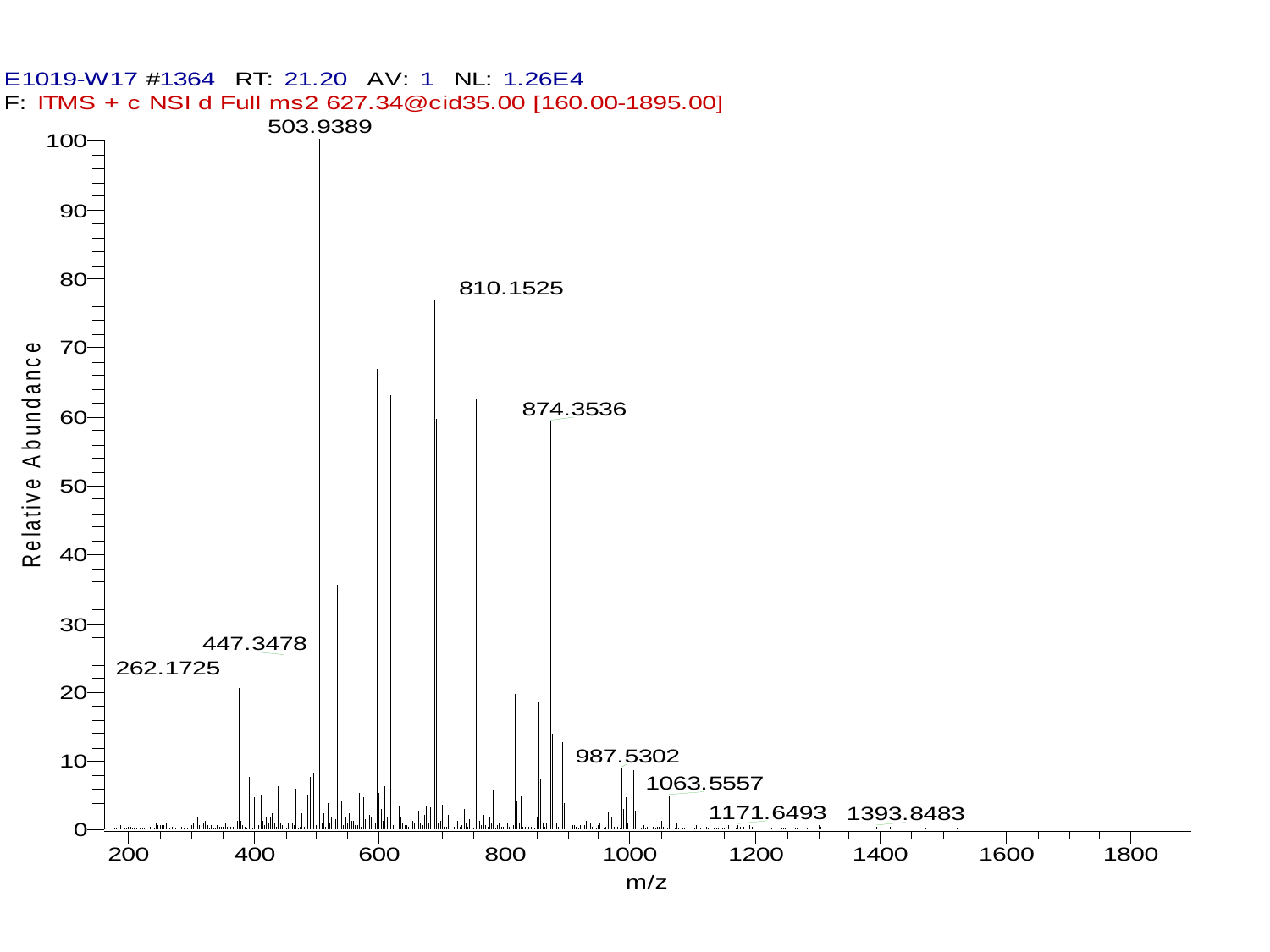

## Slide 171
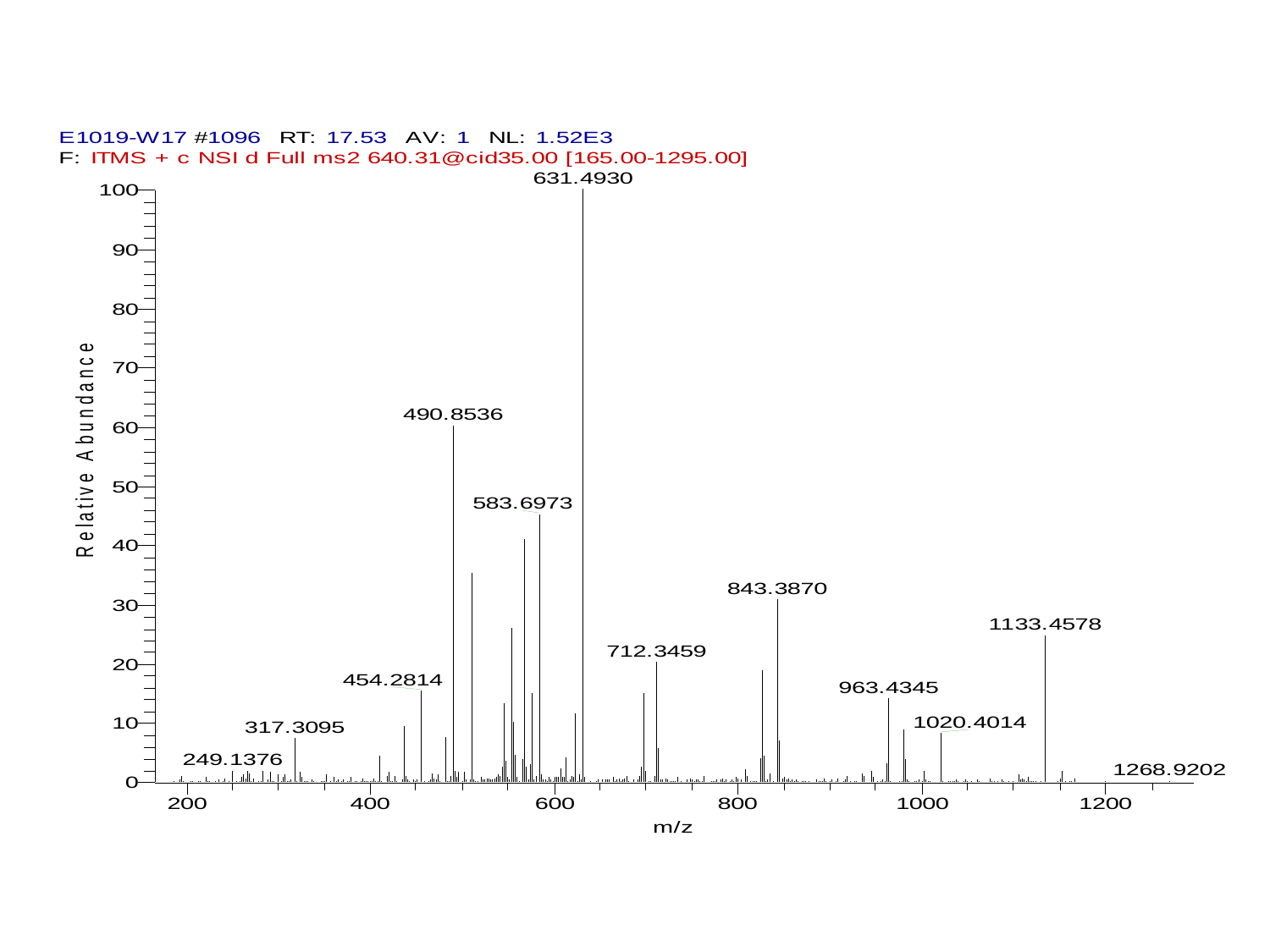

## Slide 172
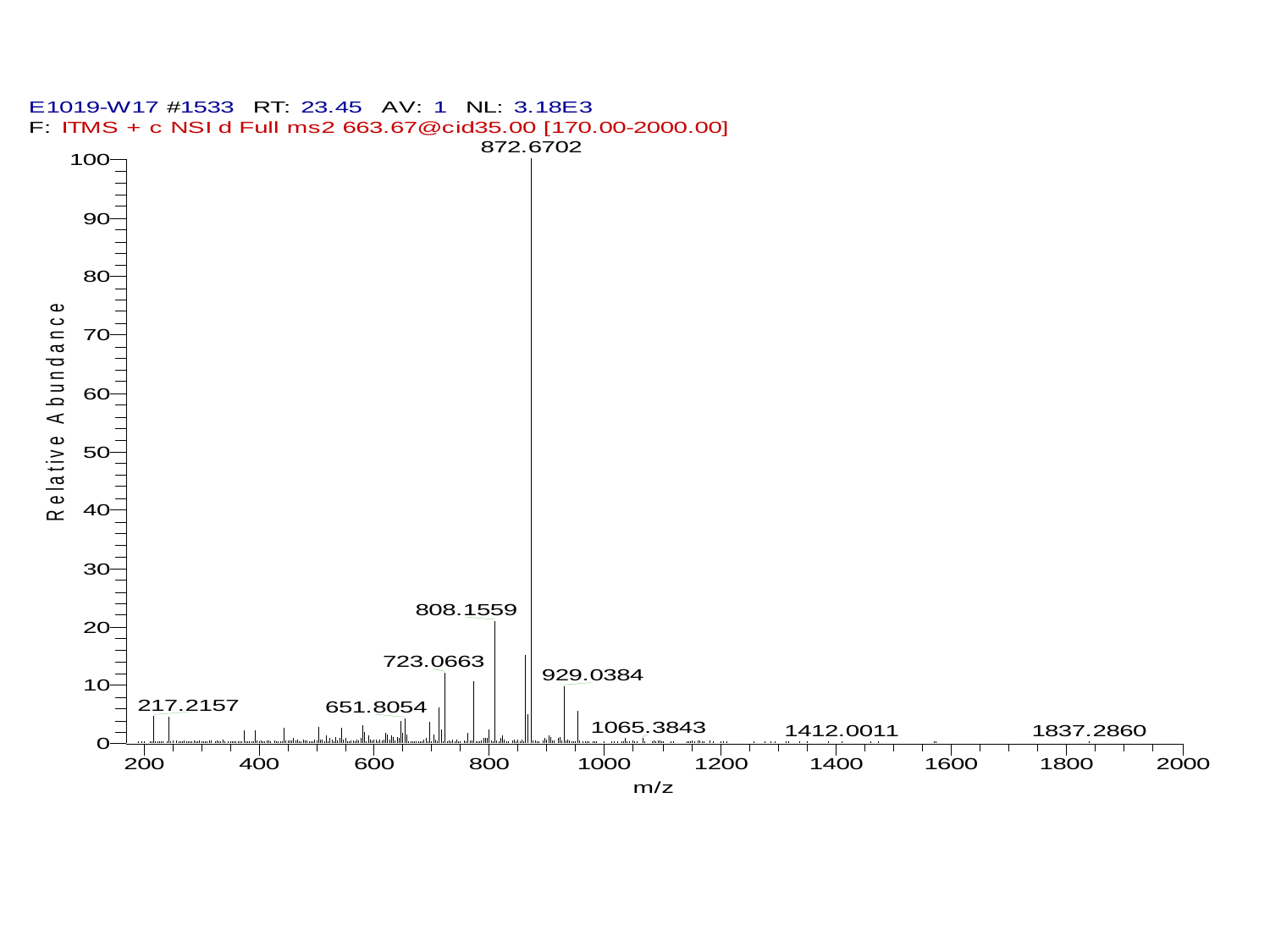

## Slide 173
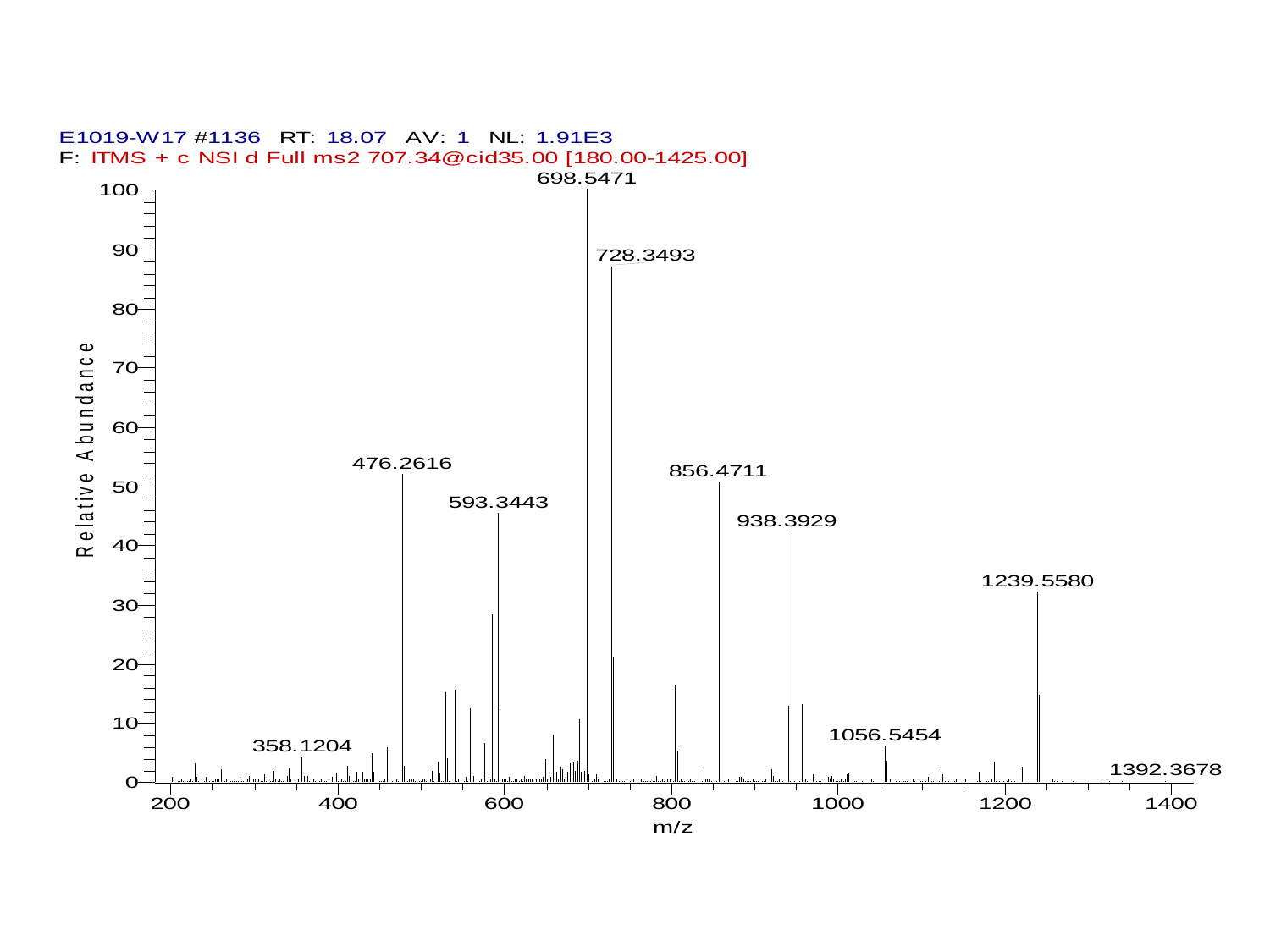

## Slide 174
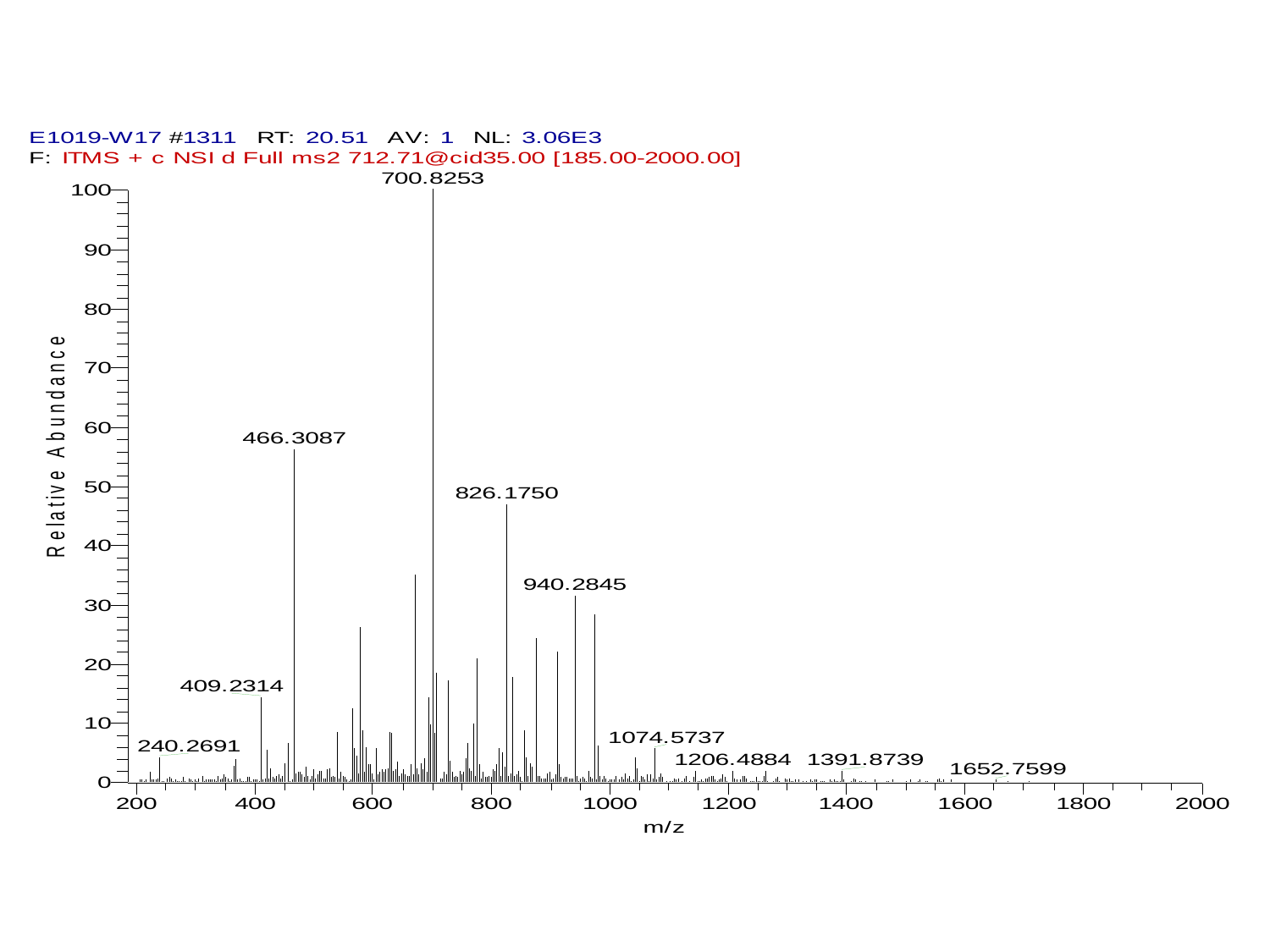

## Slide 175
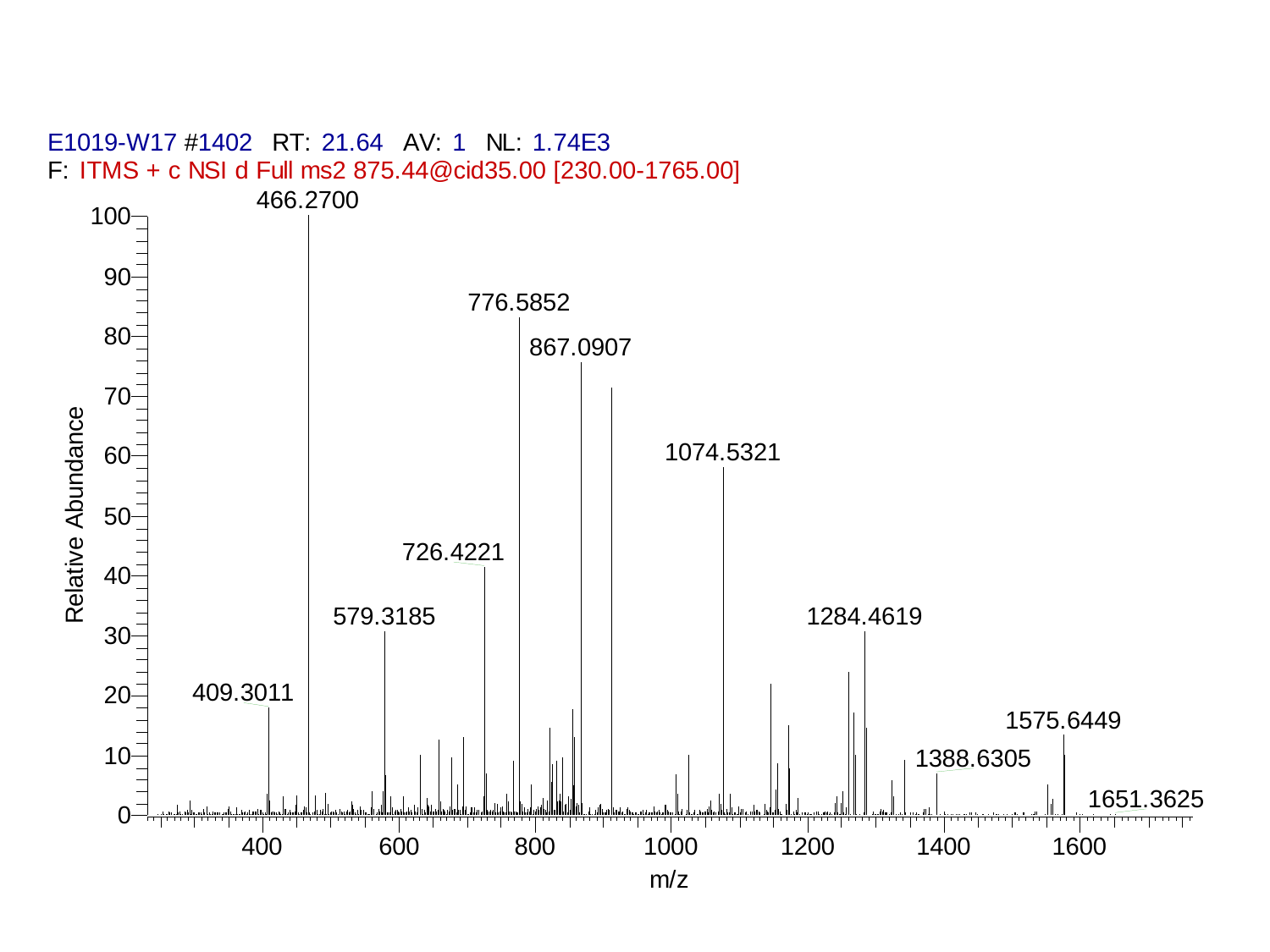

## Slide 176
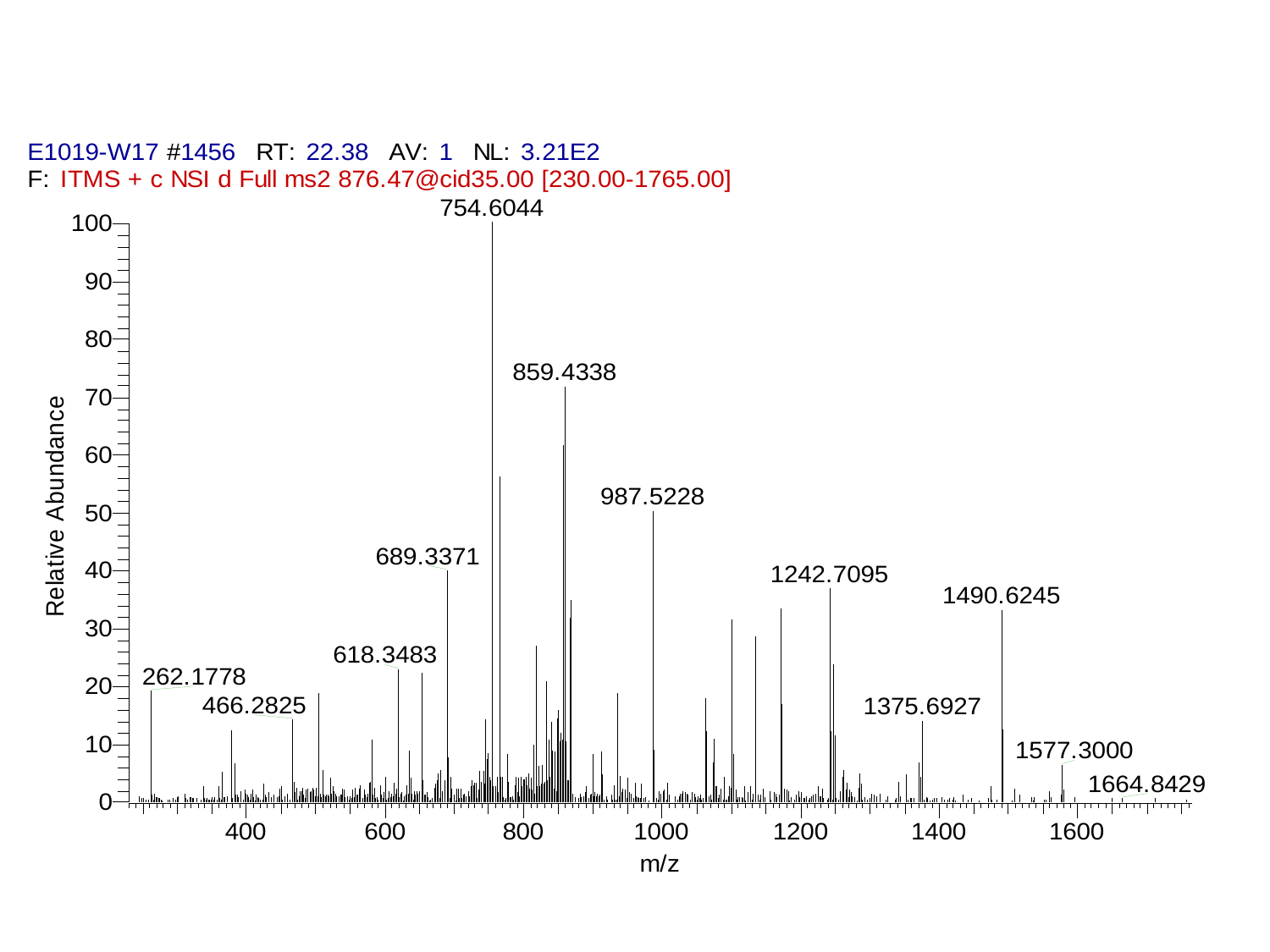

## Slide 177
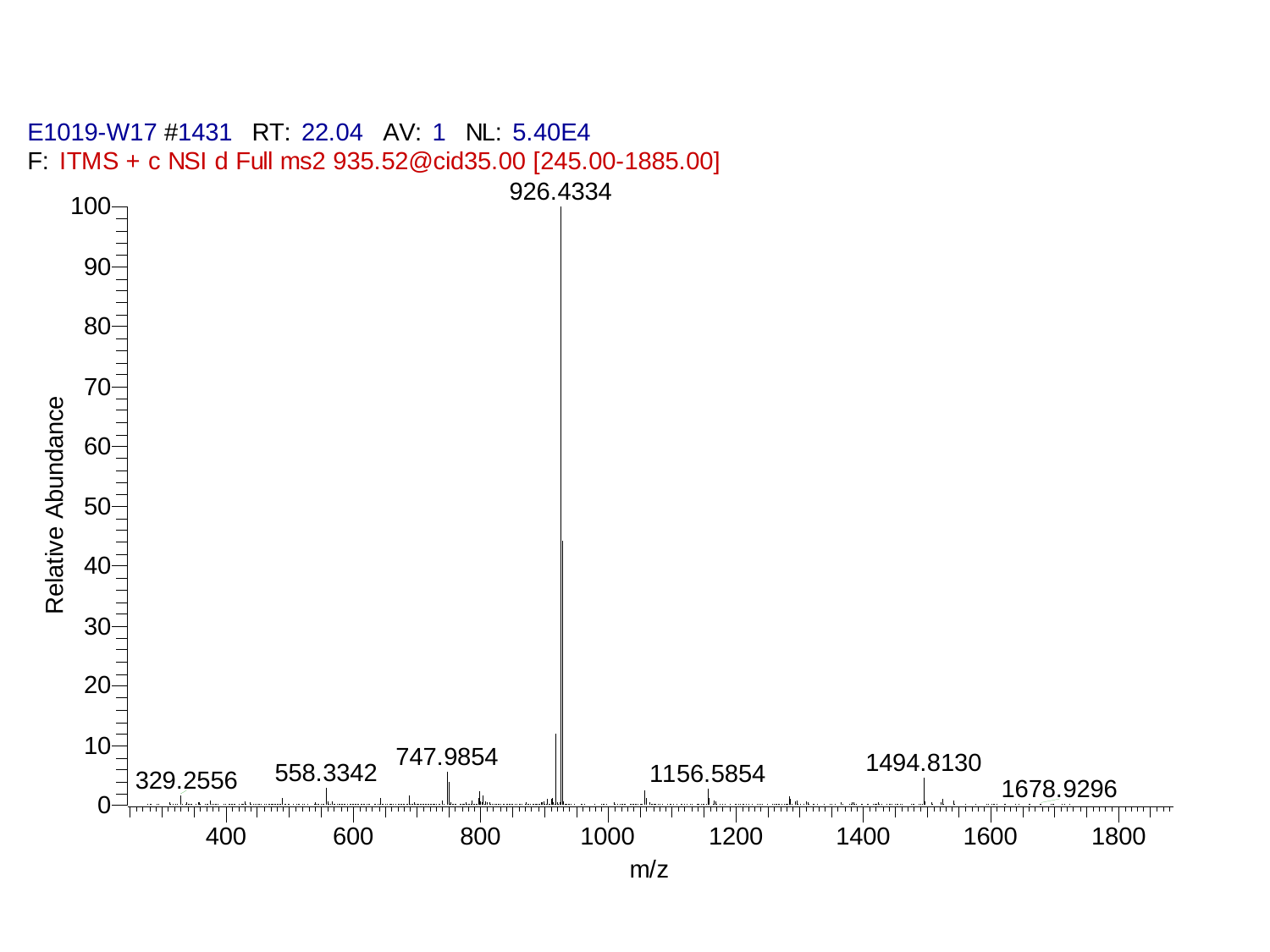

## Slide 178
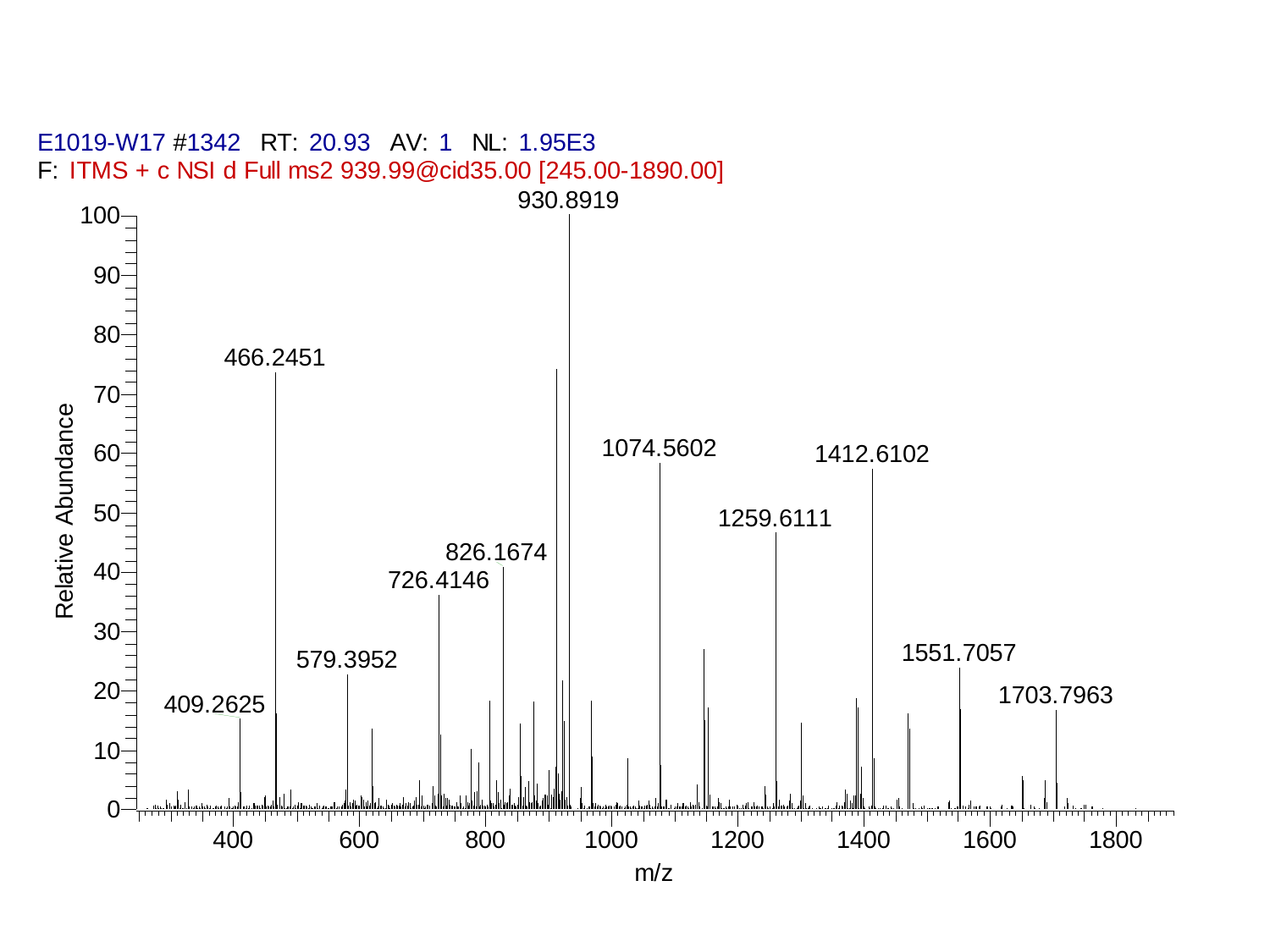

## Slide 179
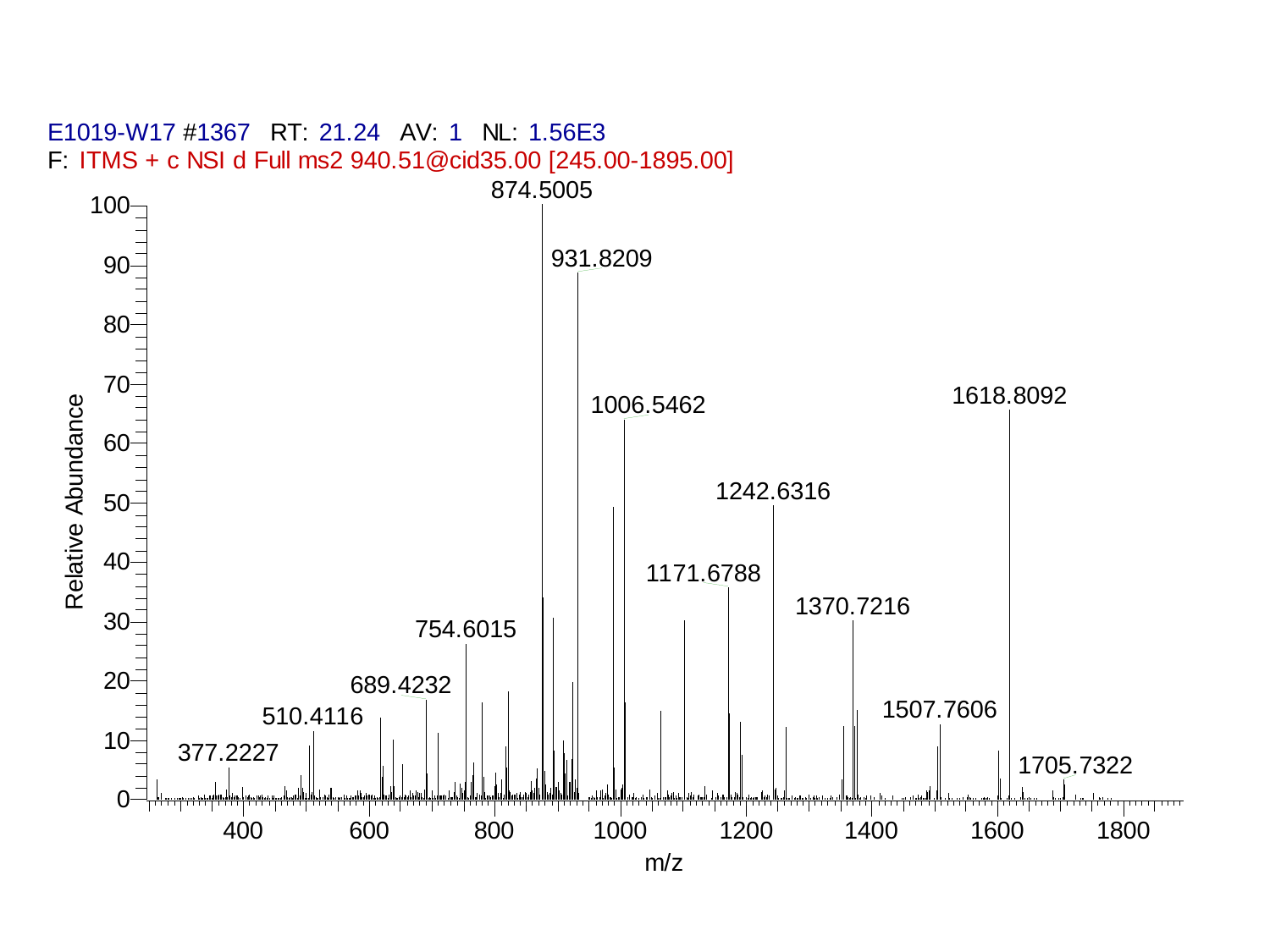

## Slide 180
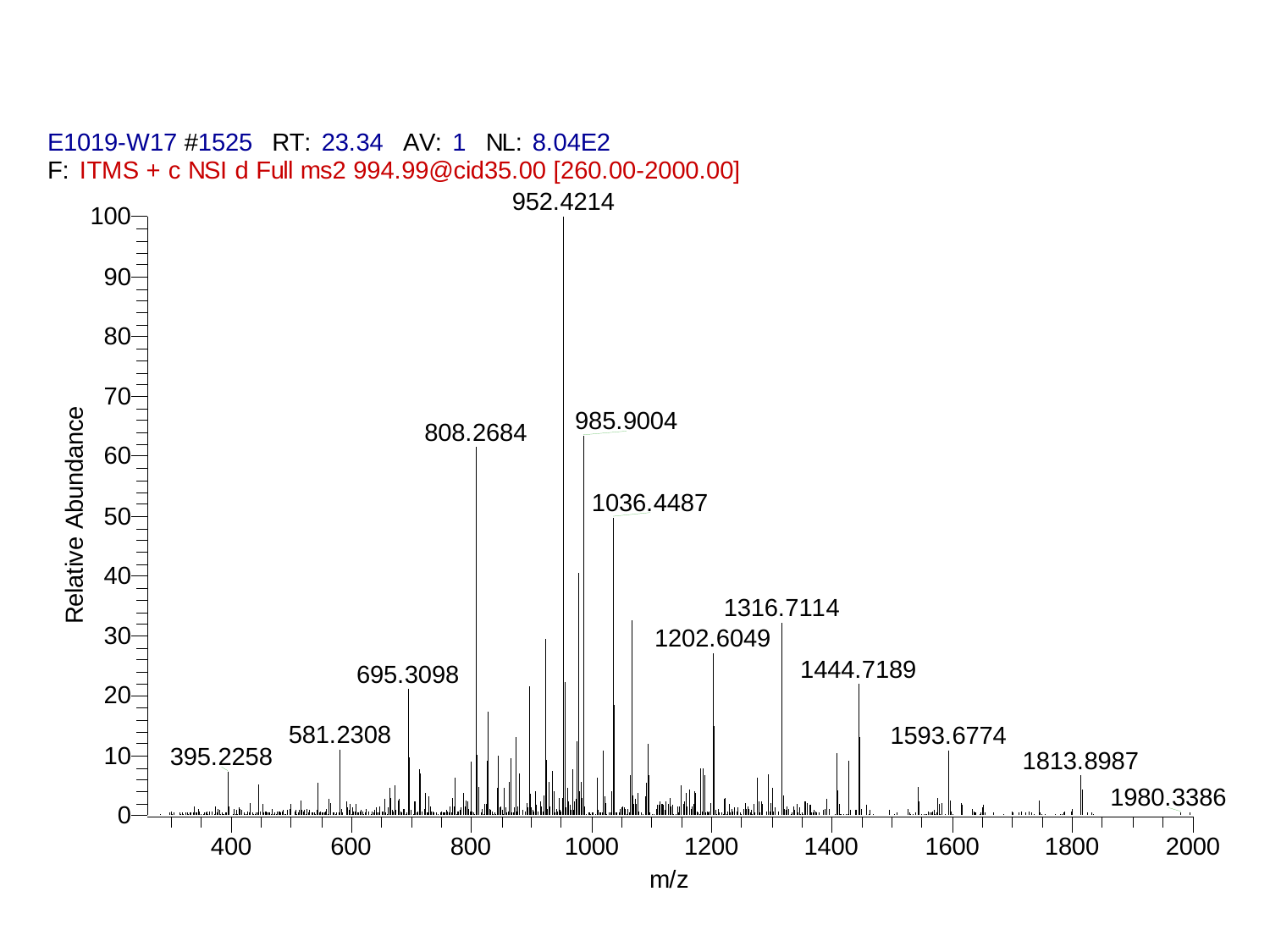

## Slide 181
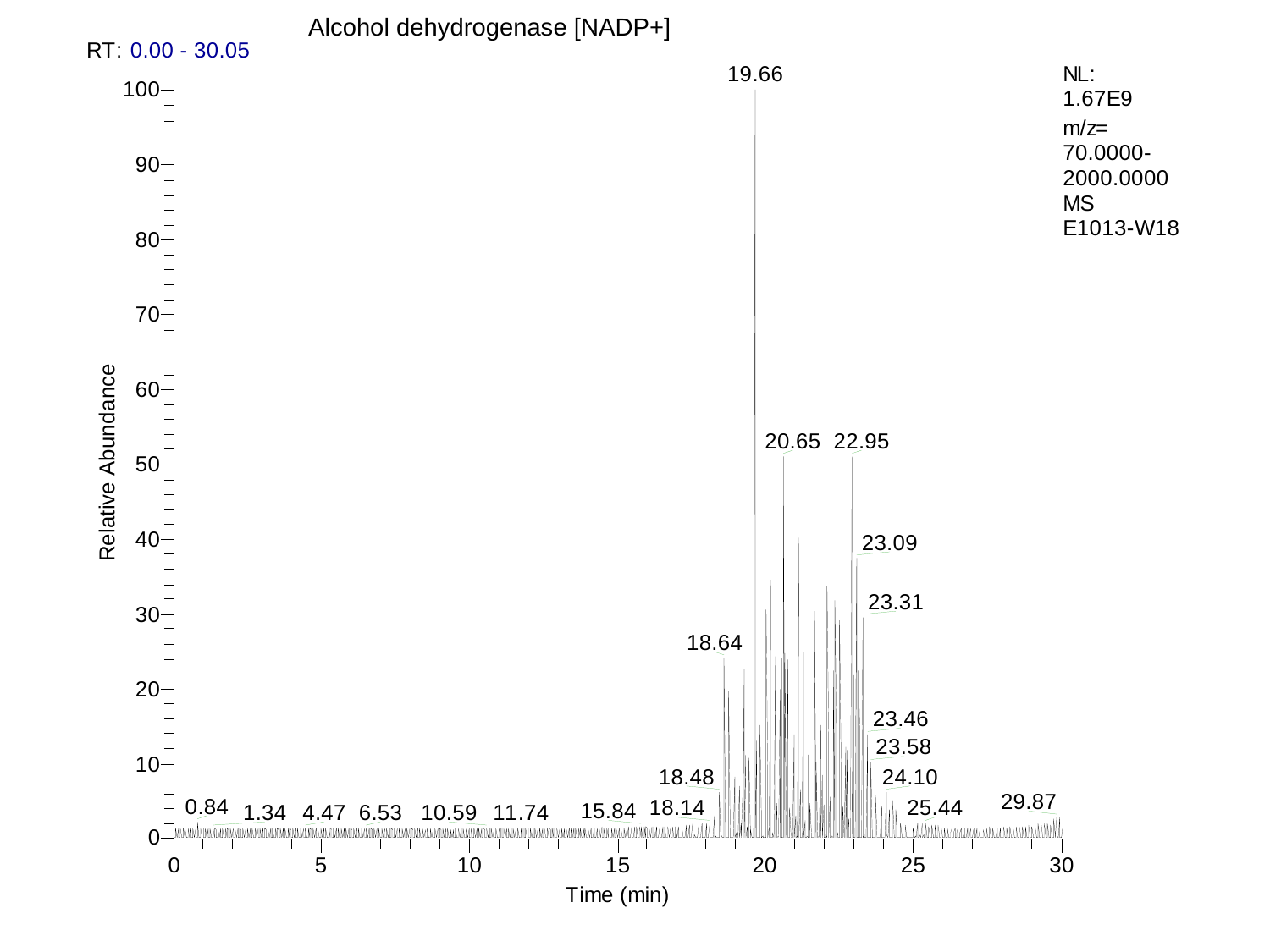

Alcohol dehydrogenase [NADP+]

## Slide 182
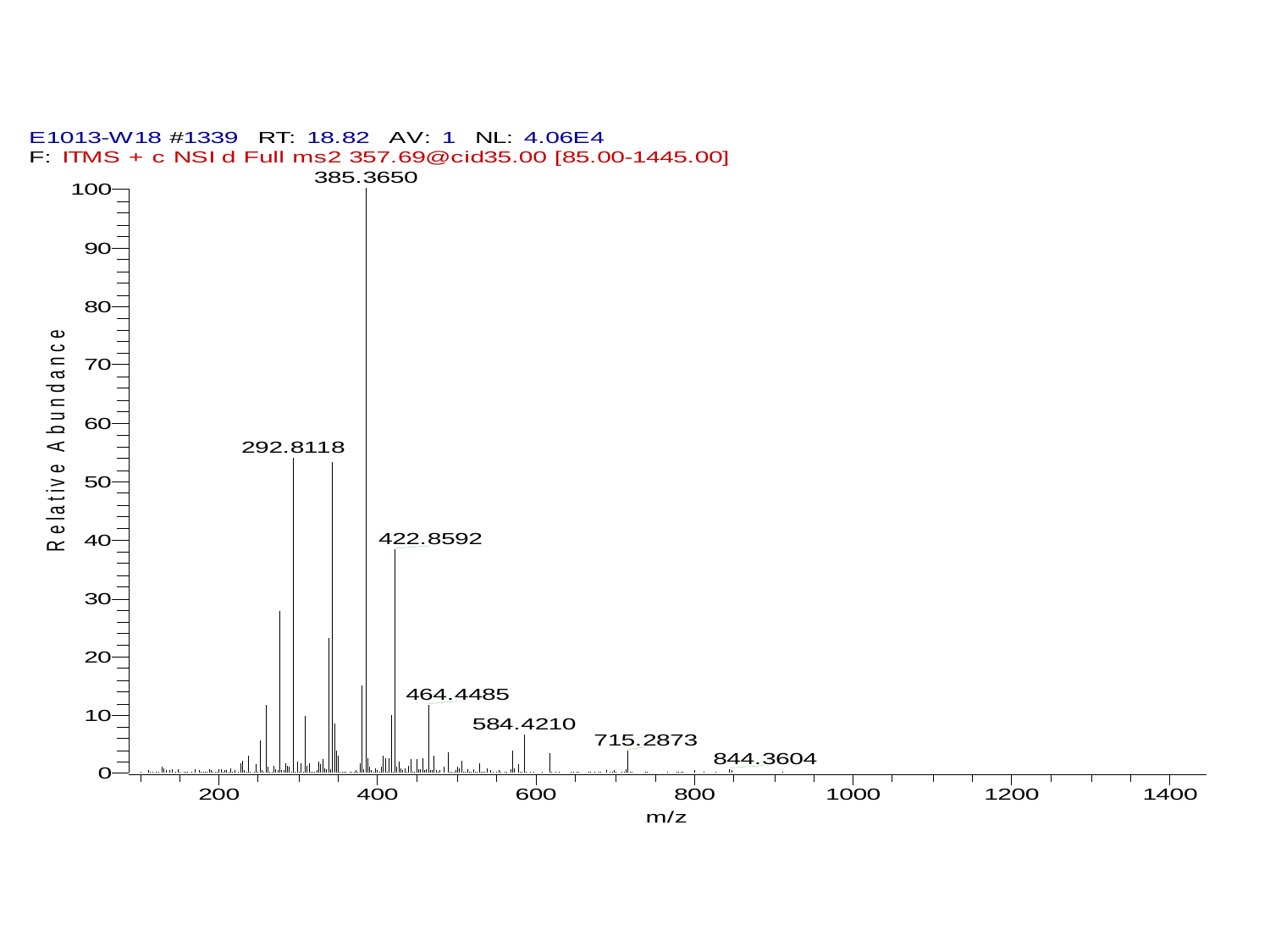

## Slide 183
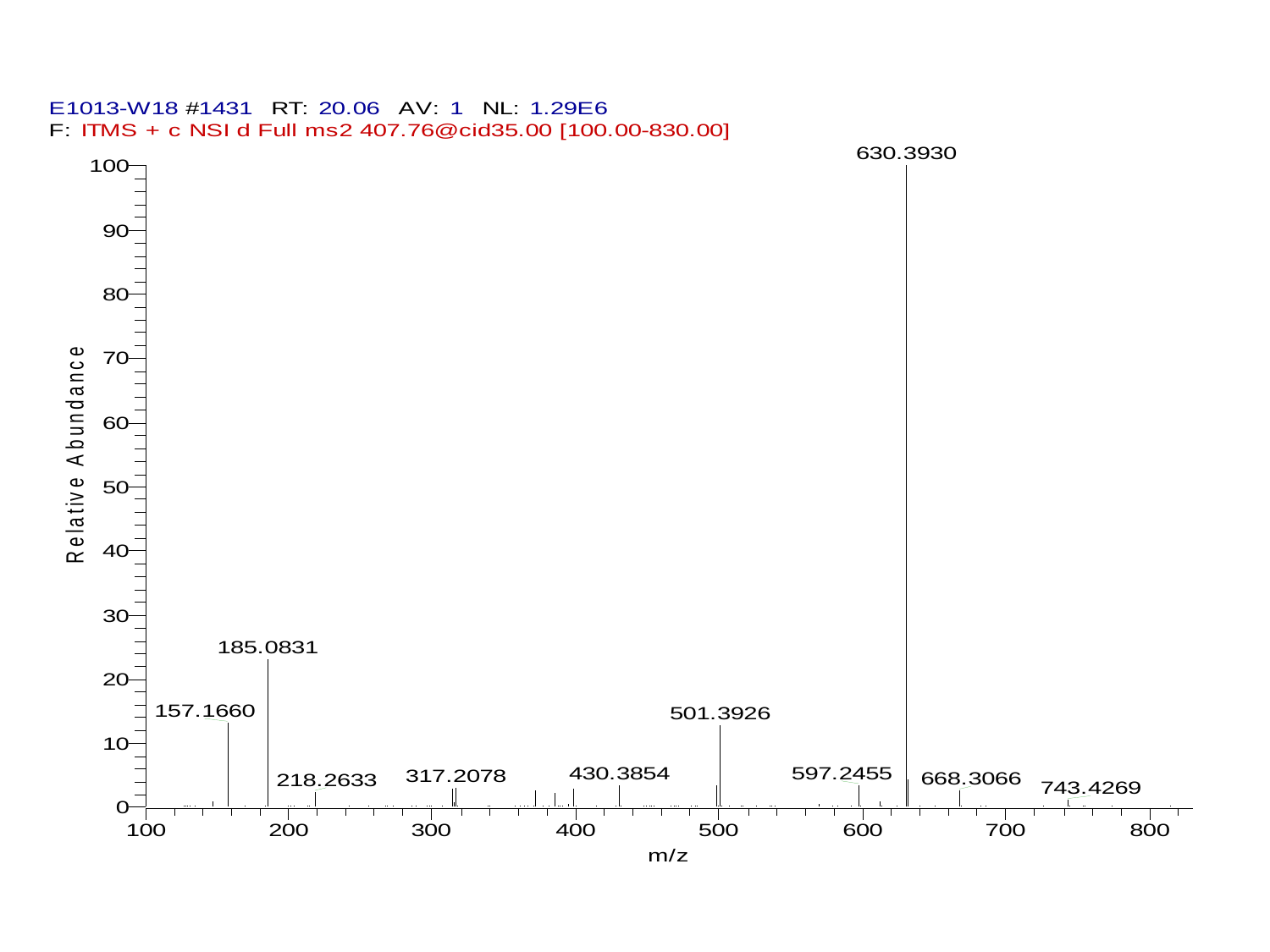

## Slide 184
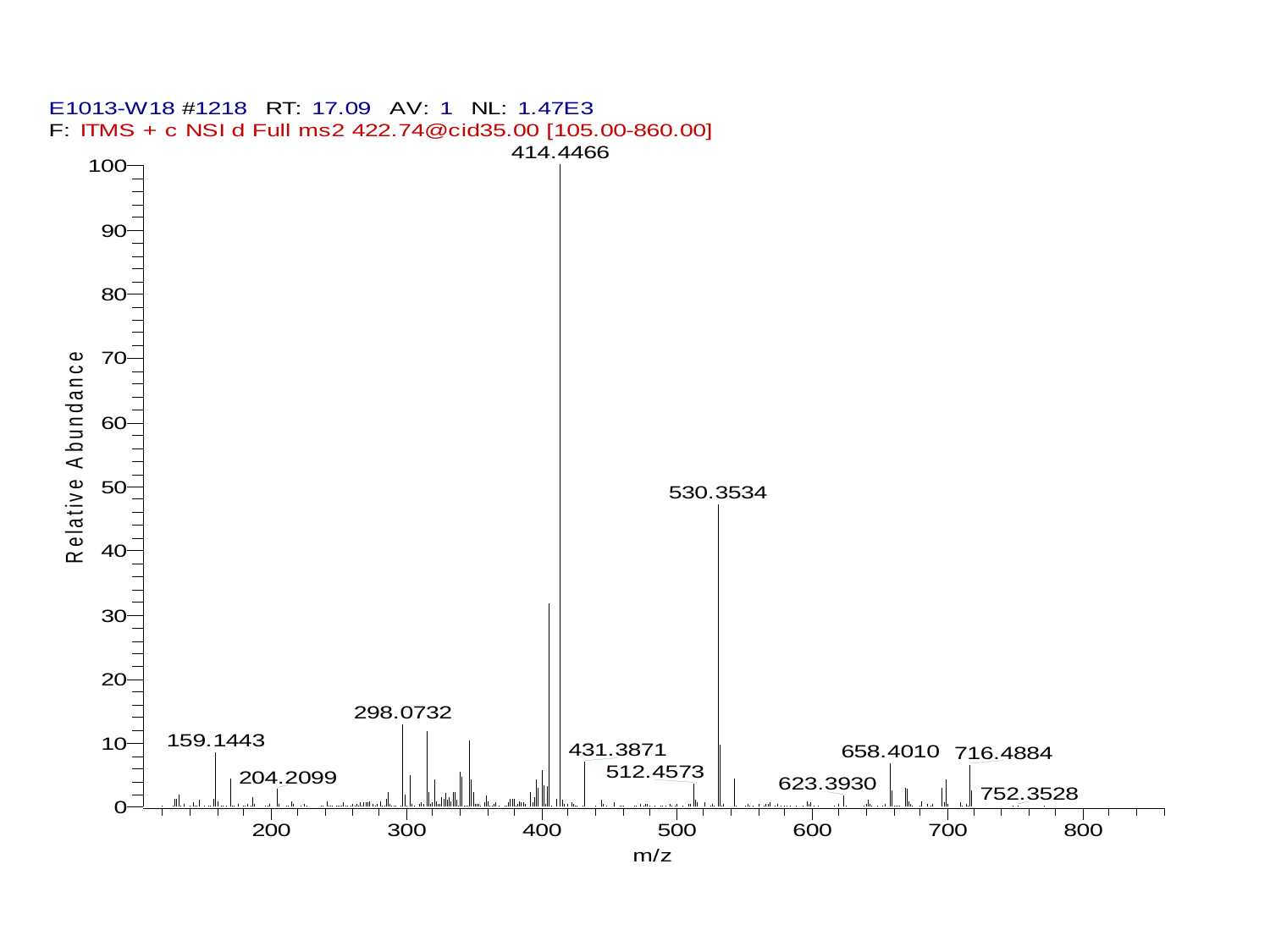

## Slide 185
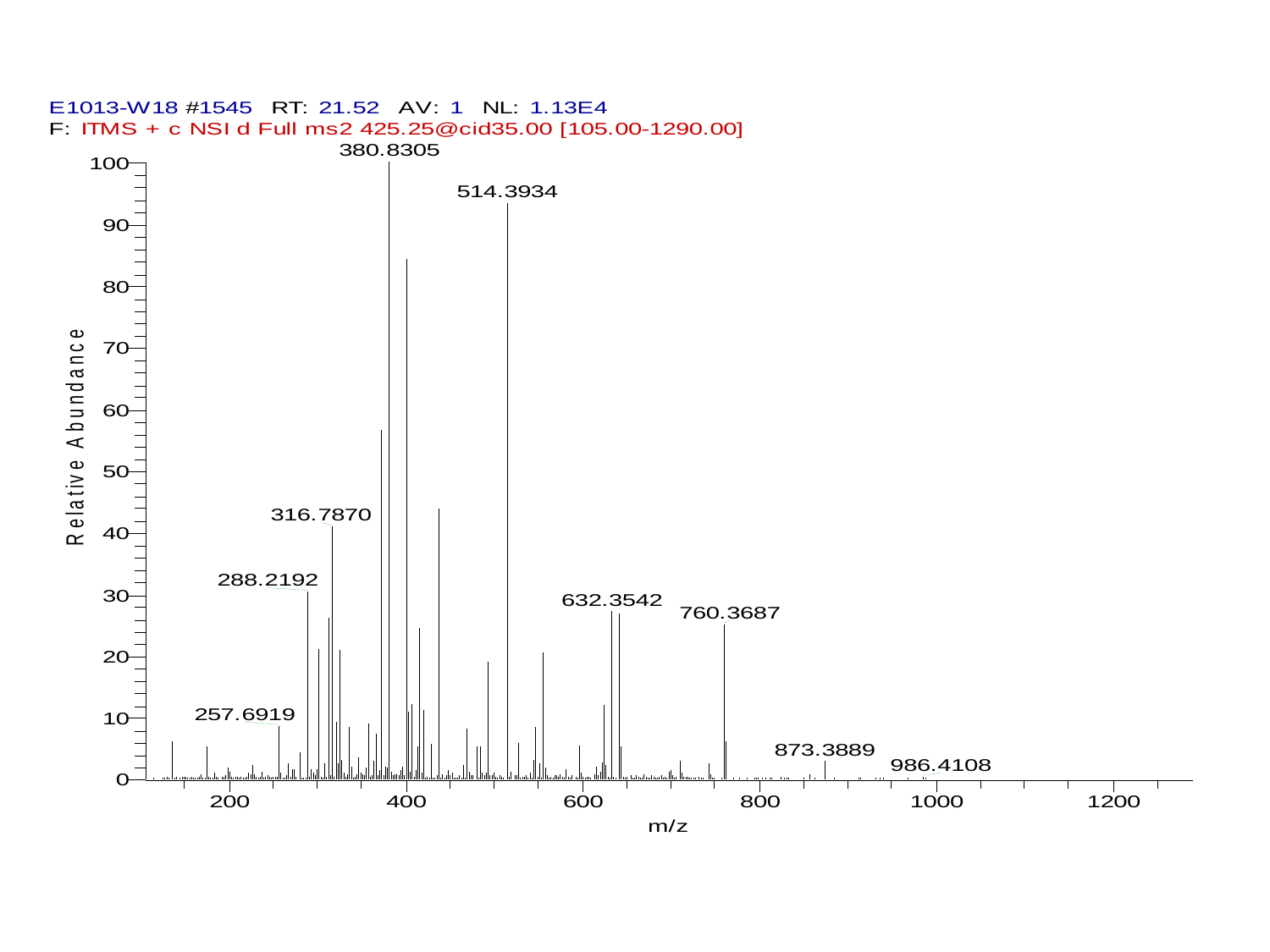

## Slide 186
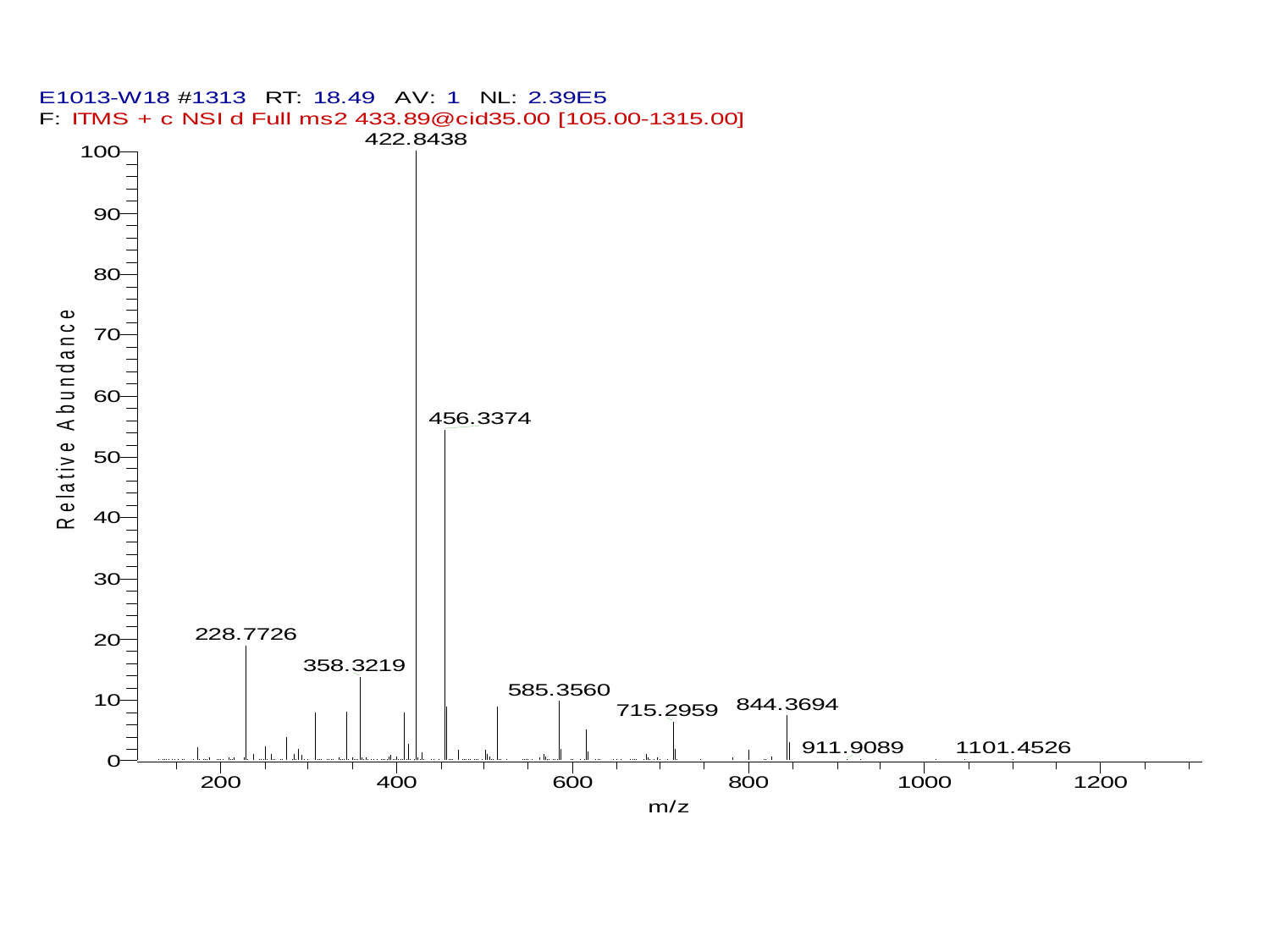

## Slide 187
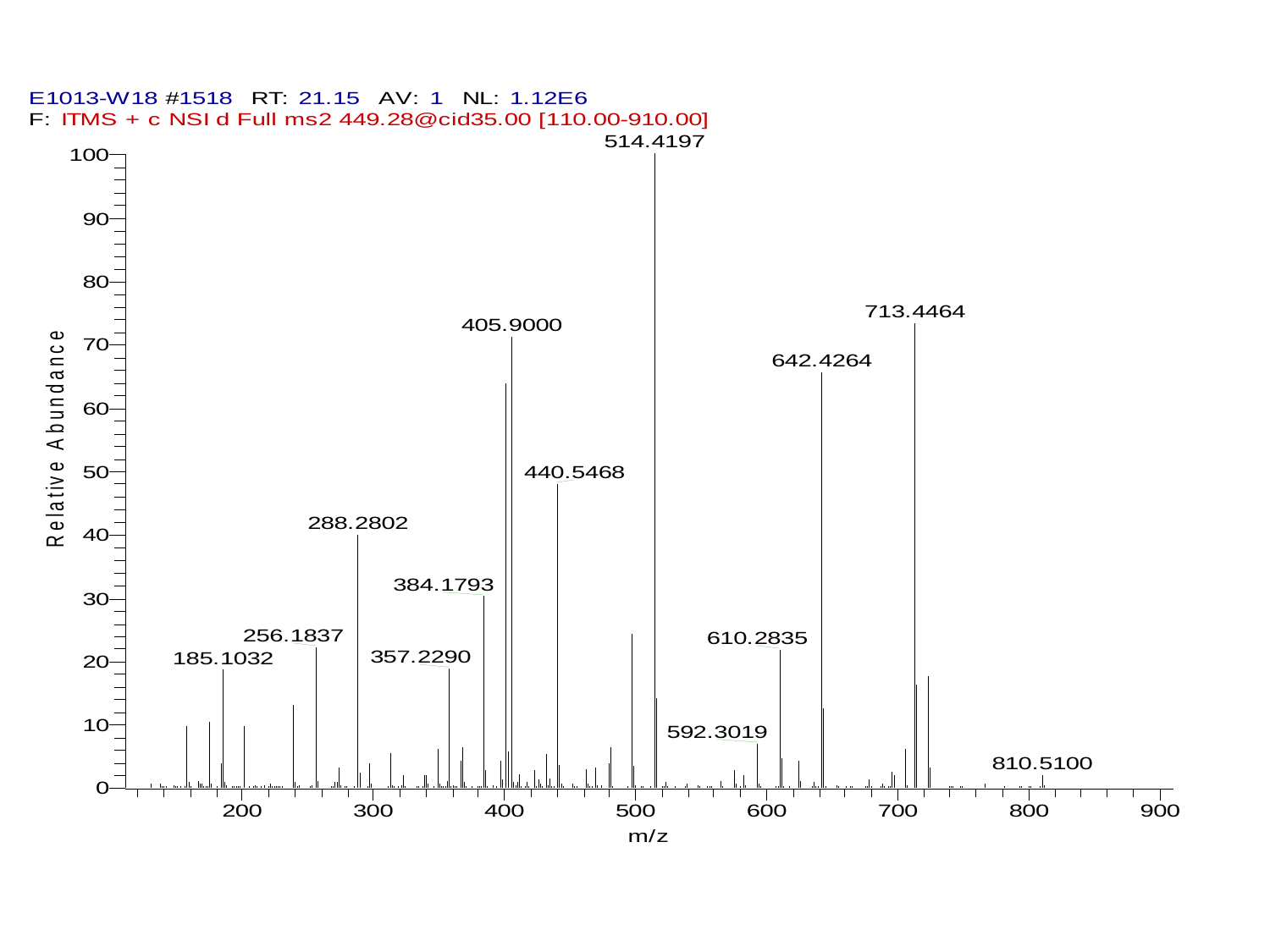

## Slide 188
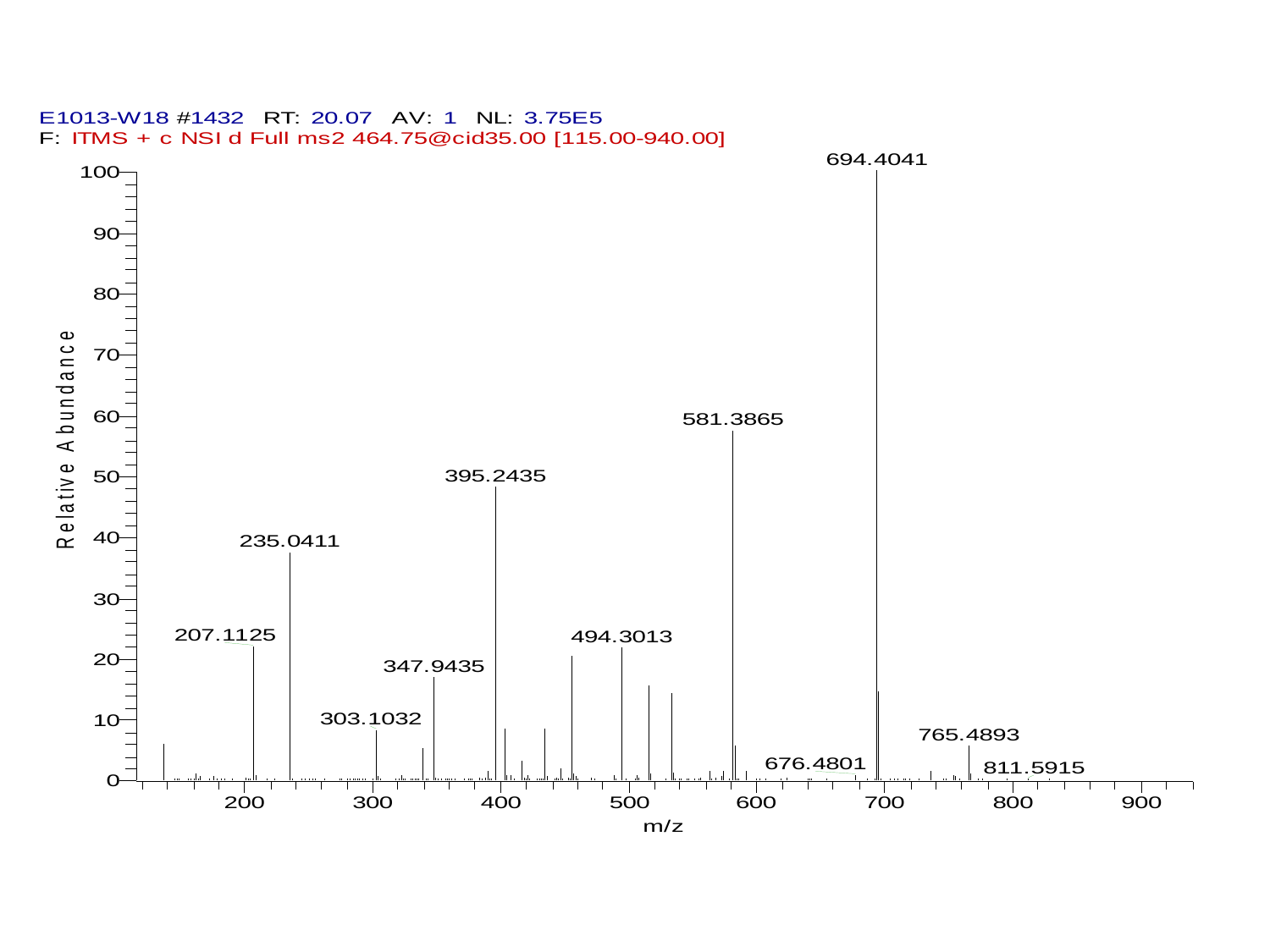

## Slide 189
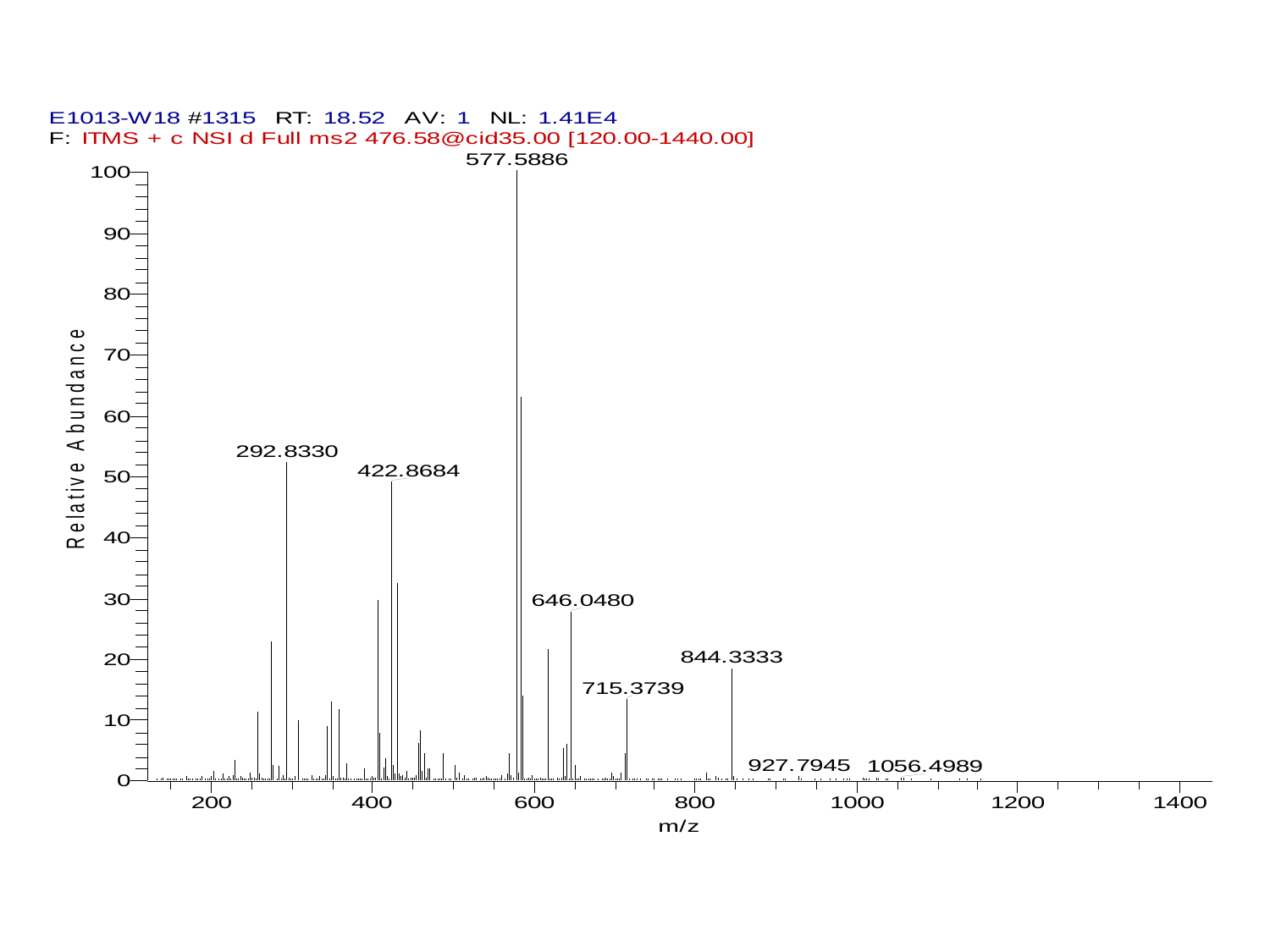

## Slide 190
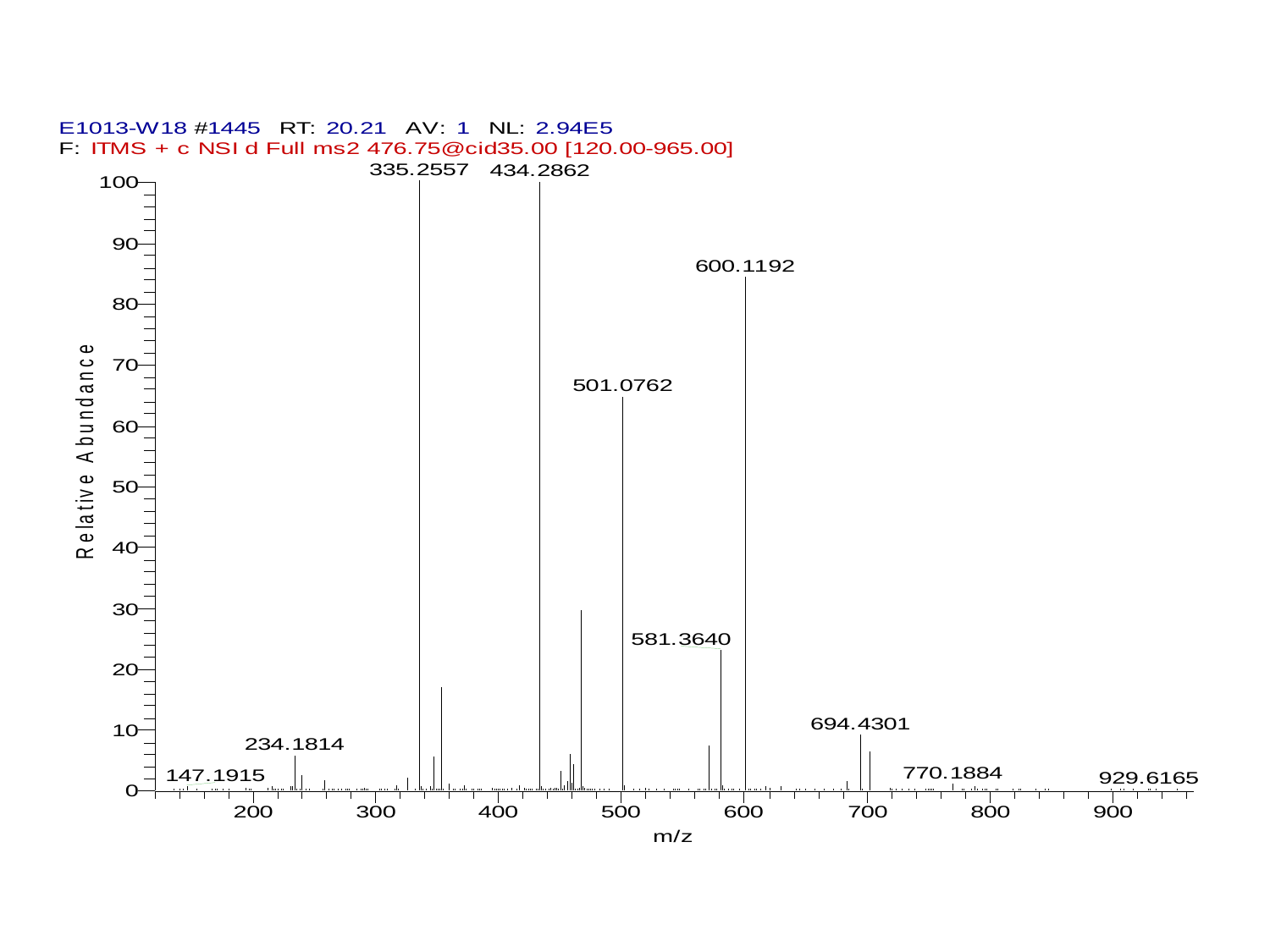

## Slide 191
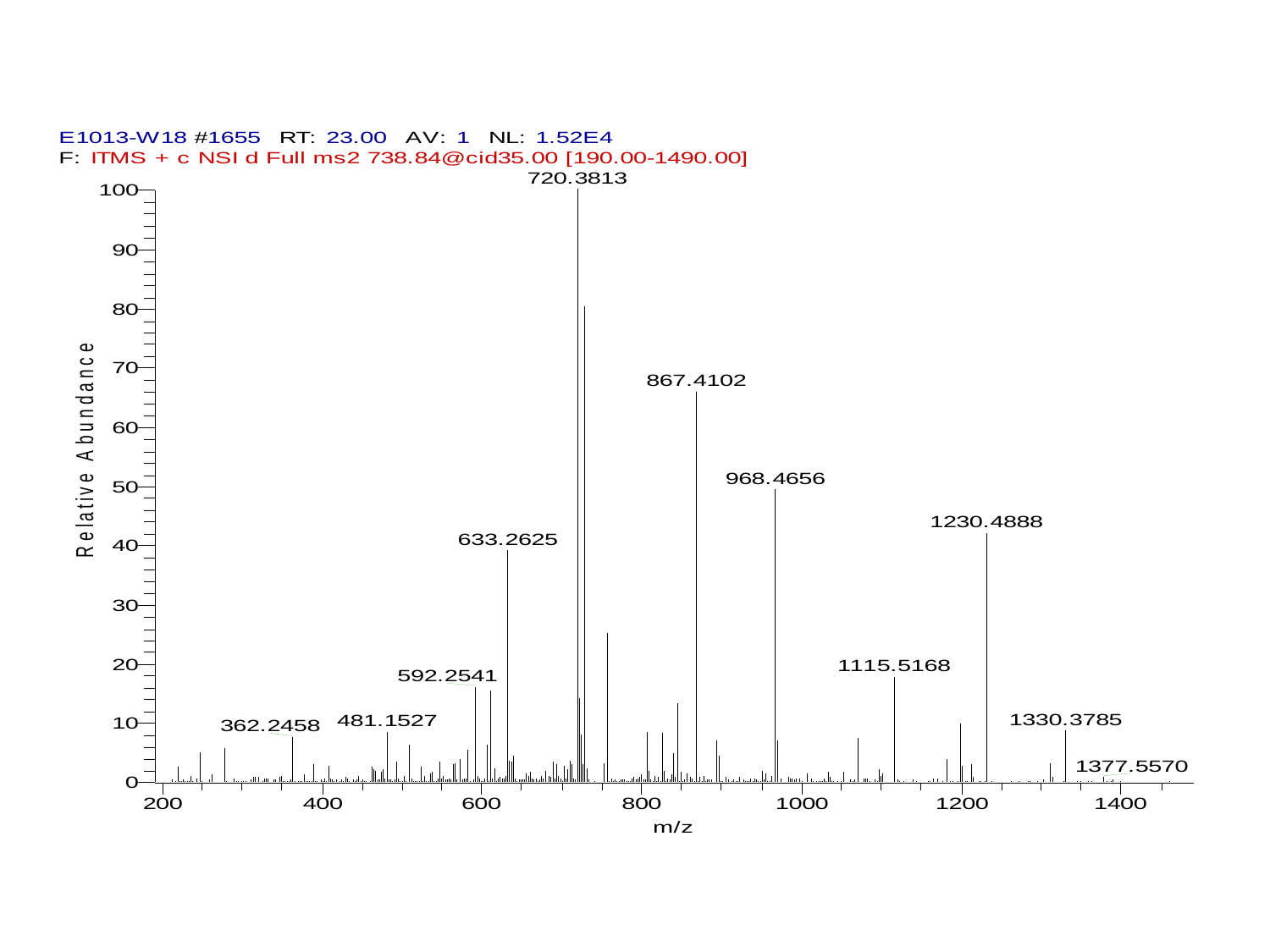

## Slide 192
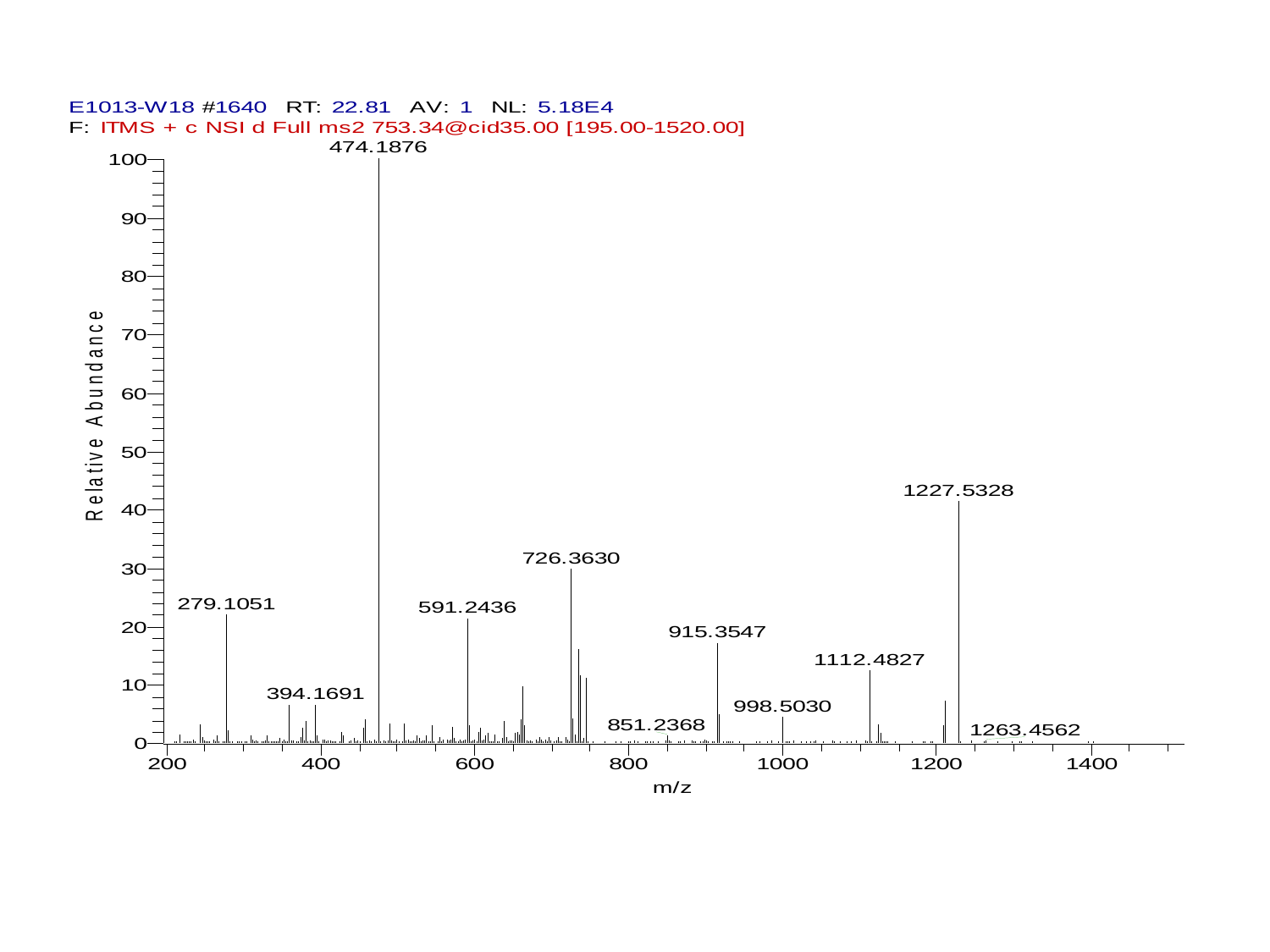

## Slide 193
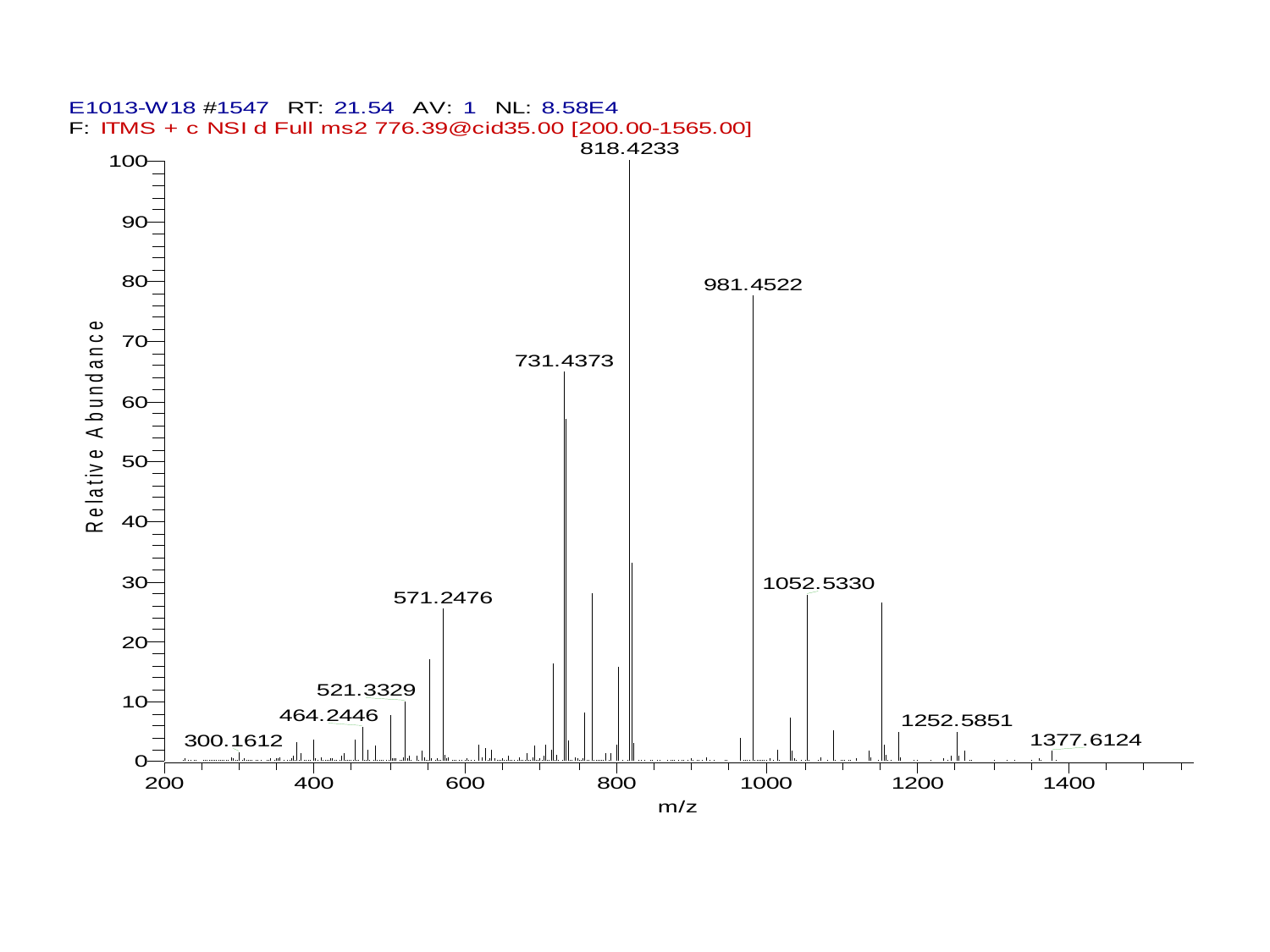

## Slide 194
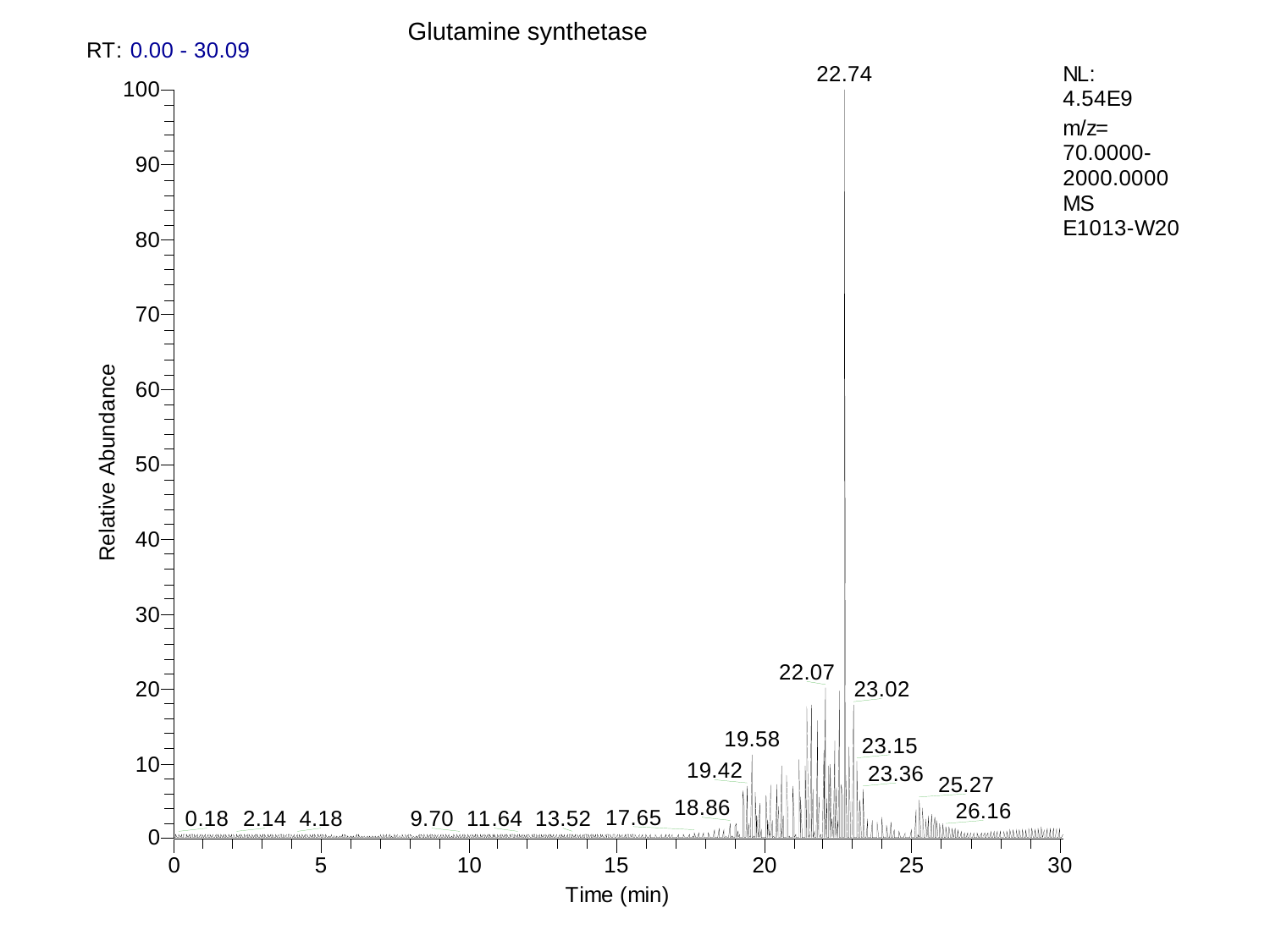

Glutamine synthetase

## Slide 195
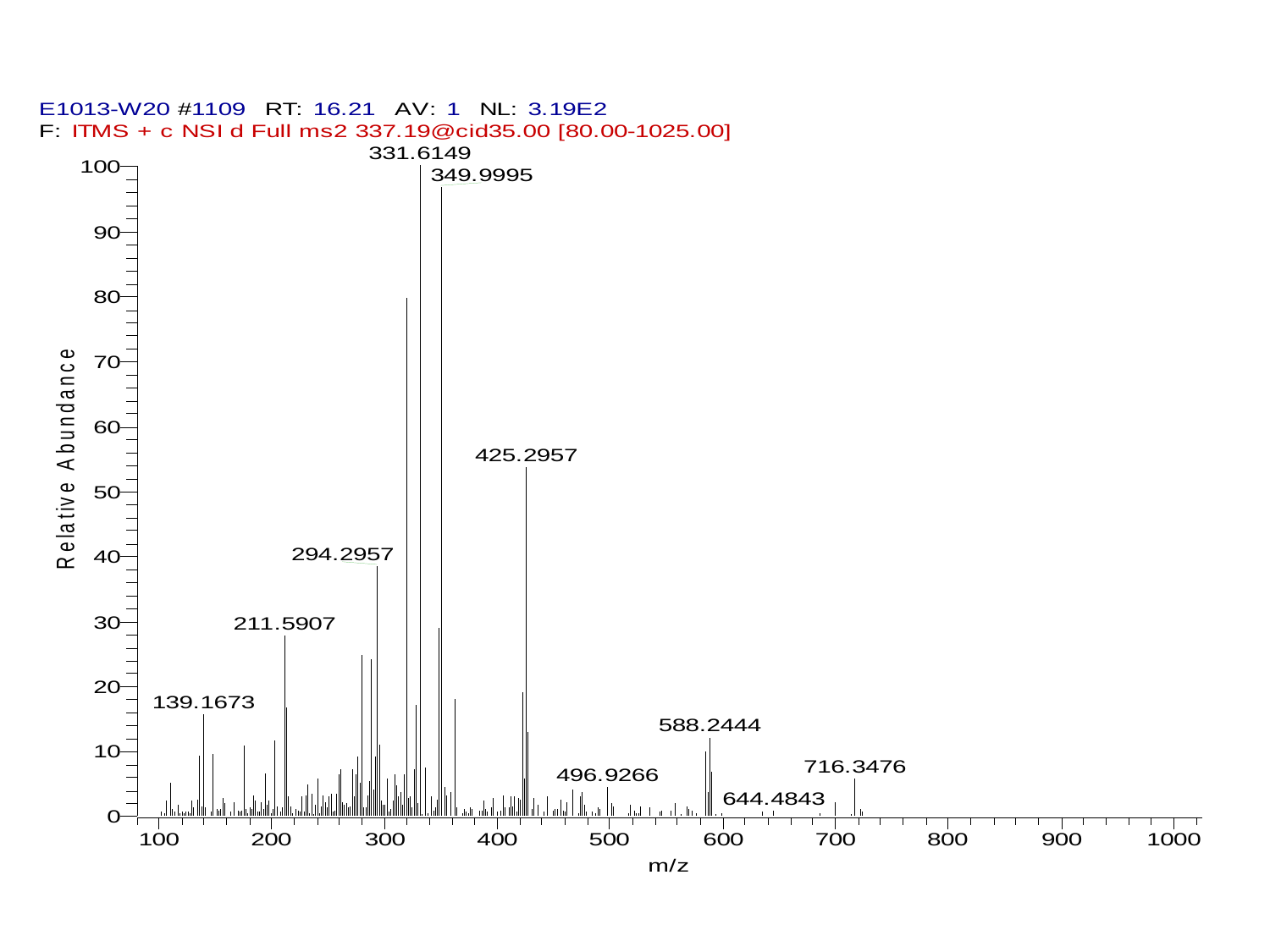

## Slide 196
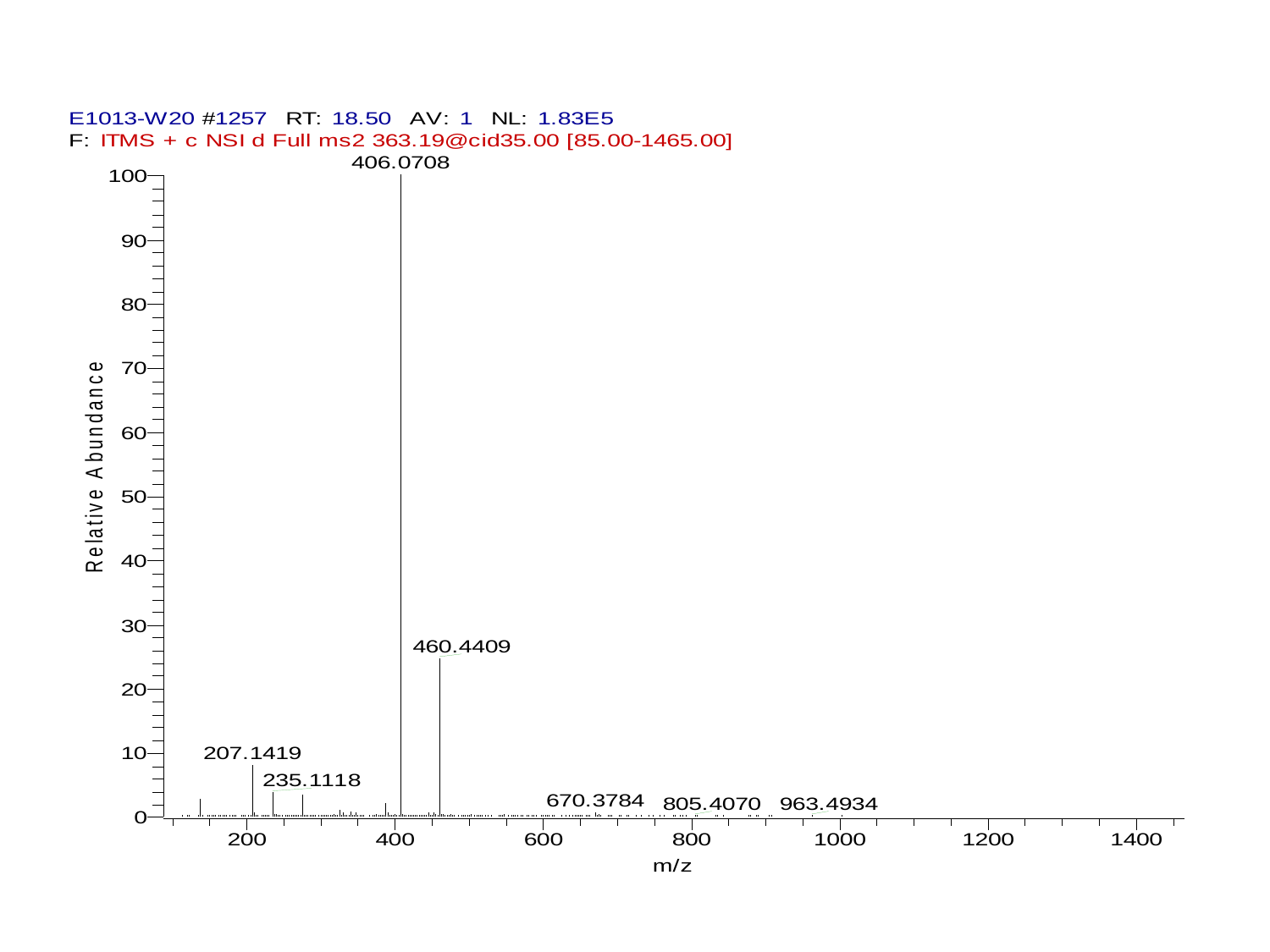

## Slide 197
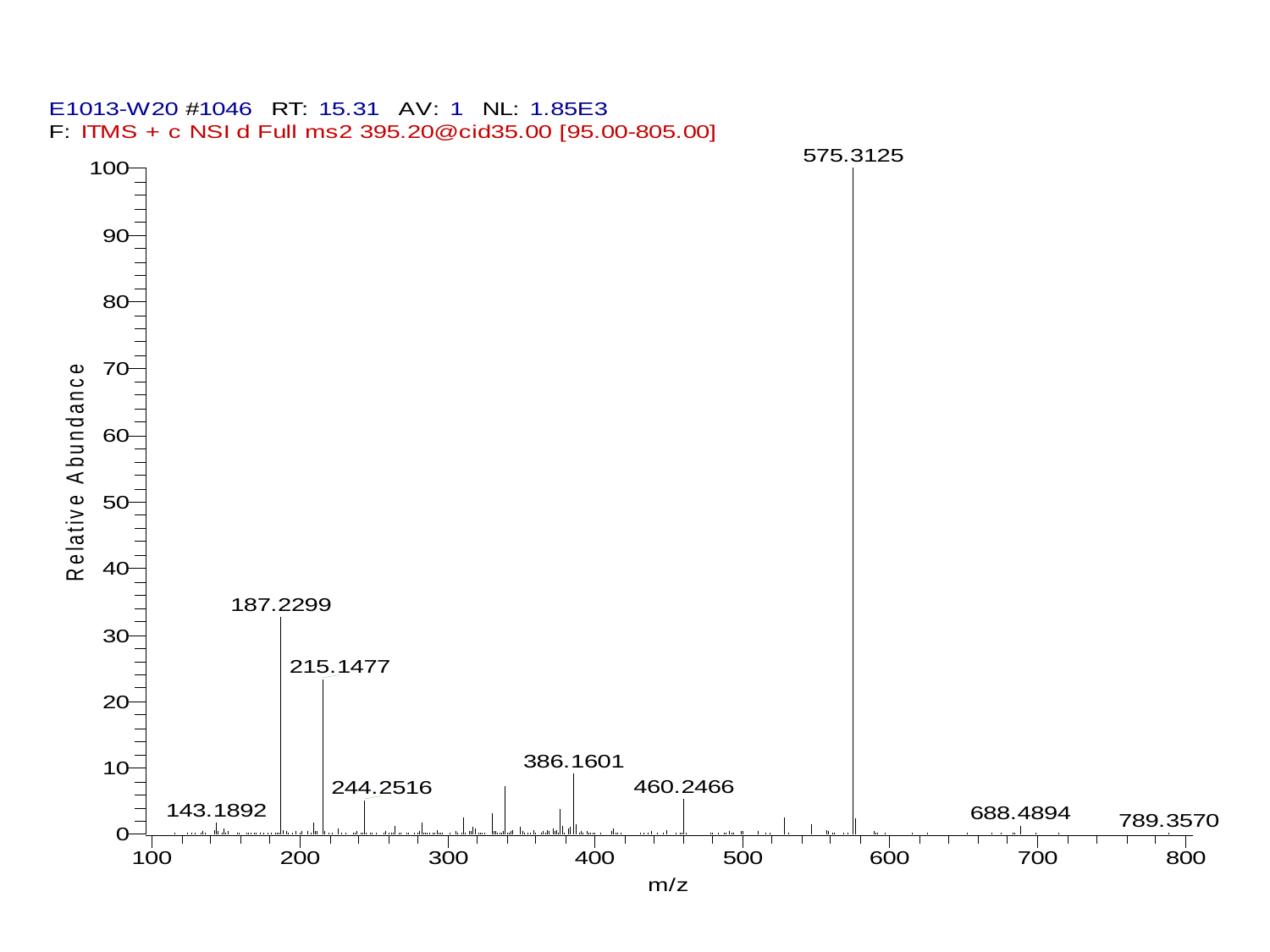

## Slide 198
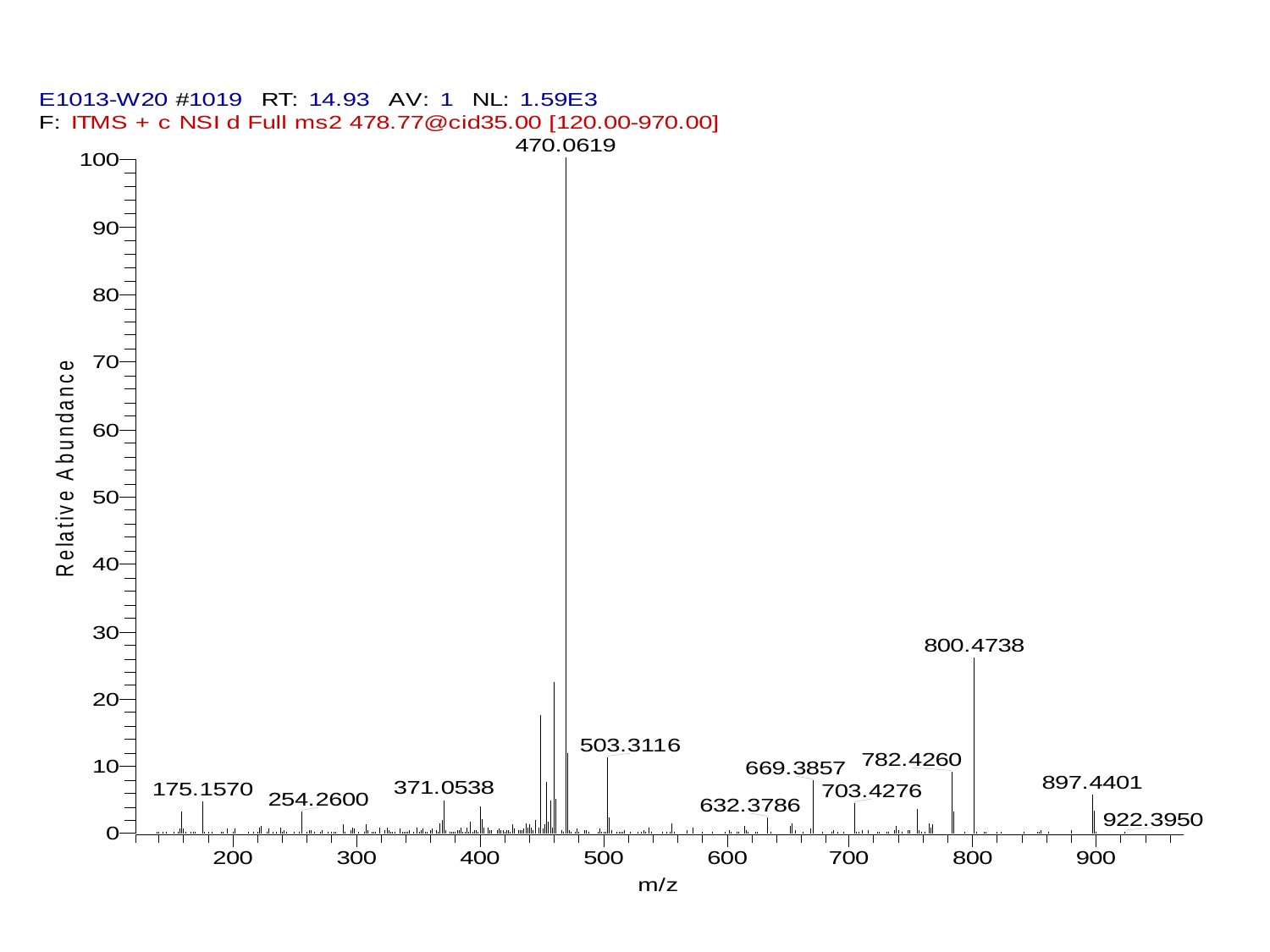

## Slide 199
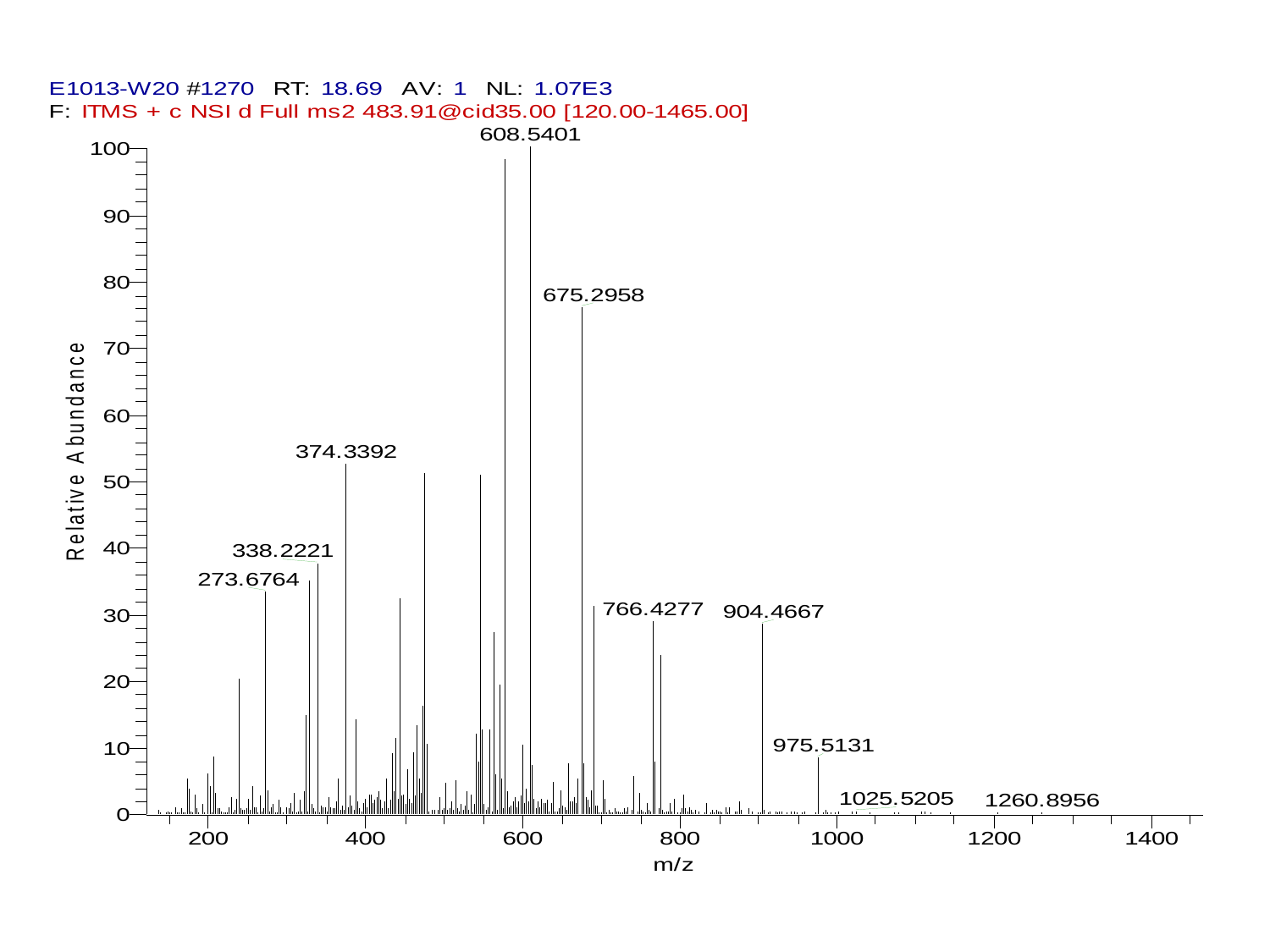

## Slide 200
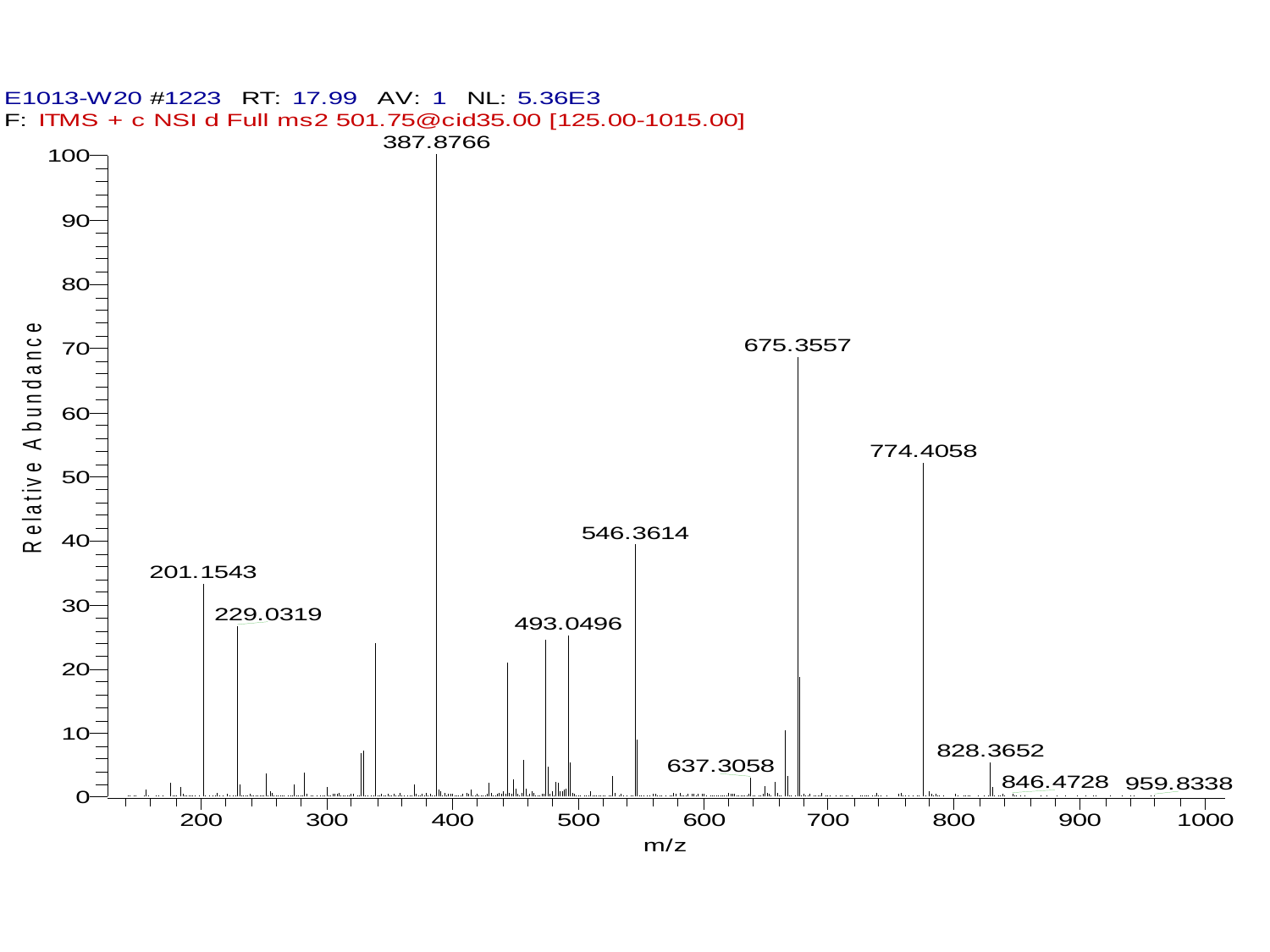

## Slide 201
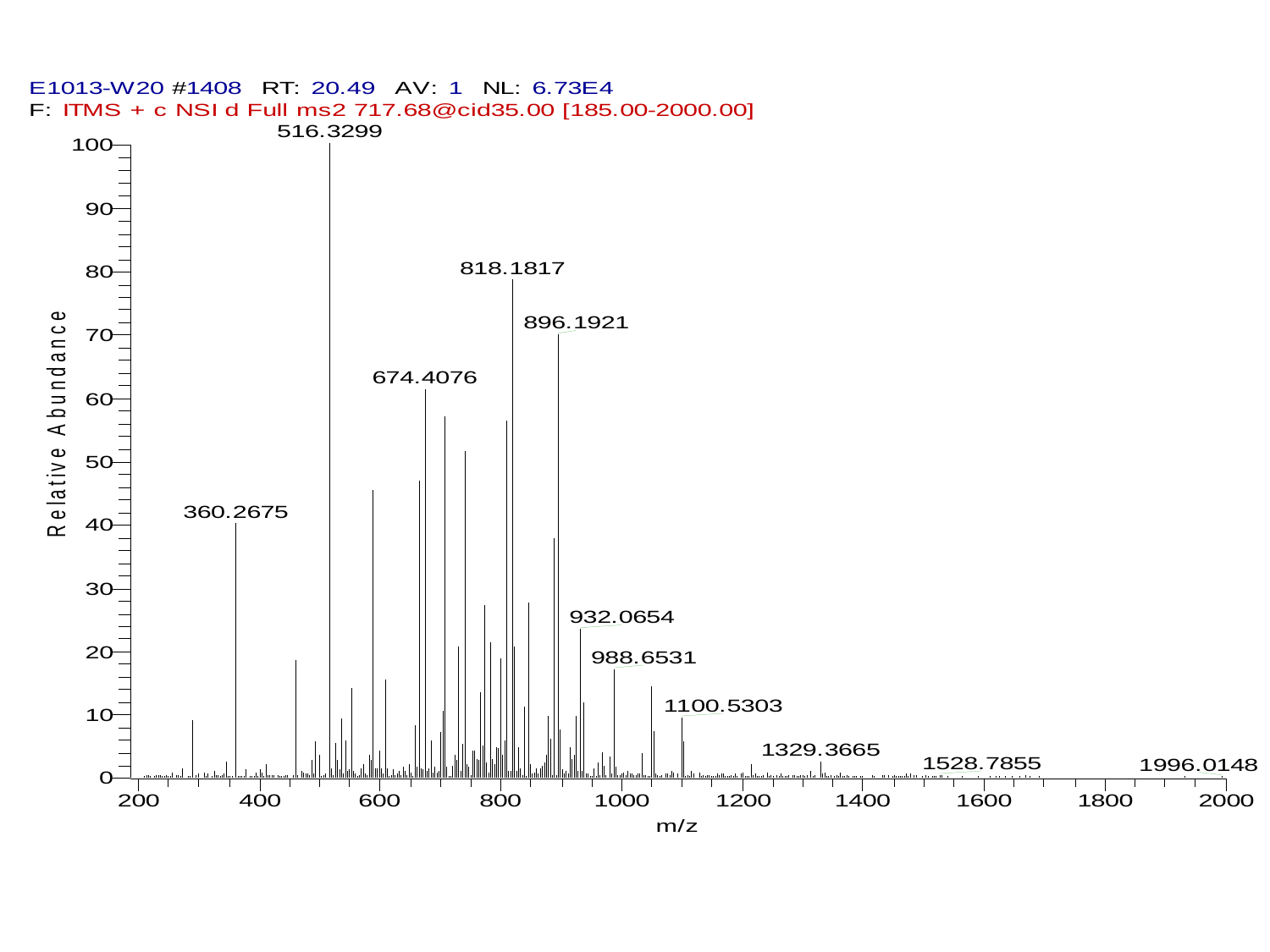

Supplement: Supplementary file 1 [file ijms-19-01242-s001.zip › Supplemenatry figures.pptx]
