# Supplementary material for: Proteomic Identification of the Galectin-1-Involved Molecular Pathways in Urinary Bladder Urothelial Carcinoma
Source: Int J Mol Sci. 2018 Apr 19;19(4):1242. doi: 10.3390/ijms19041242 (PMC5979315; doi:10.3390/ijms19041242)
Supplement: Supplementary file 1 [file ijms-19-01242-s001.zip › supplementary File S1.docx]

*Silver staining*

Briefly, the gel was fixed in fixation solution (ethanol/water/acetic acid, 4/5/1, v/v/v) after electrophoresis and treated with sensitizing solutions [0.5 M sodium acetate, 0.5% (w/v) sodium thiosulphate] for 30 min. After sensitization, the gels were washed and incubated in 0.25%(w/v) silver nitrate solution for 20 min and then developed by incubating with the developing solution [2.5% (w/v) sodium carbonate and 0.015% (v/v) formaldehyde] until the protein spots appeared. Glutardialdehyde was not used in the silver staining due to the protein identification by mass spectrometry.

*Human Gal-1-specific DNA precursor oligonucleotides for siRNA molecules and scrambled control DNA*

The sequences were the following:

Human Gal-1-specific DNA precursor oligonucleotides for siRNA molecules and scrambled control DNA.

The sequences were the following:

Gal-1_120siU: 5’-GATCCCCAACCTGTGCCTGCACTTCATTCAAGAGATGAAGTG CAGGCACAGGTTTTTTTA-3’

Gal-1_120siL: 5’-AGCTTAAAAAAACCTGTGCCTGCACTTCATCTCTTGAATGAAGTG CAGGCACAGGTTGGG-3’

Gal-1_120scU: 5’-GATCCCCCGTACACATACTTGCGCTTTCAAGAGAAGCGCAAG TATGTGTACGTTTTTA-3’

Gal-1_120scL: 5’-AGCTTAAAAACGTACACATACTTGCGCTTCTCTTGAAAGCGC AAGTATGTGTACGGGG-3’

Gal-1_380siU: 5’-GATCCCCCAAGATCAAATGTGTGGCCTTCAAGAGAGGCCACA CATTTGATCTTGTTTTTA-3’

Gal-1_380siL: 5’-AGCTTAAAAACAAGATCAAATGTGTGGCCTCTCTTGAAGGCC ACACATTTGATCTTGGGG-3’

Gal-1_380scU: 5’-GATCCCCAGGACGTGCGCACATATATTTCAAGAGAATATATG TGCGCACGTCCTTTTTTA-3’

Gal-1_380scL:

5’-AGCTTAAAAAAGGACGTGCGCACATATATTCTCTTGAAATATATGTGCGCACGTCCTGGG-3’

**Figure legends for supplementary figures**

**Figure S1.** 11 pairs of 2-DE gels used for the recognition of de-regulated proteins.

**Figure S2.** Mass spectra for the identification of de-regulated proteins.

**Table S1.** The detail mass spectrometry data of matched peptides

| Spot | Protein I.D. | Matched^b^ peptide number | Sequence^a^ |
| --- | --- | --- | --- |
| 1 | ribonuclease/angiogenin inhibitor 1 (RNH1)^a^ | 11 | AKPDFKELTVSNNDINEAGVR ( 2,6.29,0.63,1)  ESLKELSLAGNELGDEGAR (3,5.96,0.53,1)  ELSLAGNELGDEGAR (2,5.28,0.56,1)  ELTVSNNDINEAGVR (2,4.59,0.43,1)  LQALEKDKPSLR (2,4.00,0.45,1)  ASLRELALGSNK (2,3.29,0.28,1)  VNPALAELNLR (2,3.16,0.47,1)  LDDCGLTEAR (3,2.35,0.36,11)  LEDAGVR (2,2.30,0.01,23)  ELALGSNK (2,1.99,0.21,1)  CKDISSALR (3,1.67,0.11,36) |
| 2 | reticulocalbin 1 (RCN1) | 3 | IVDRIDNDGDGFVTTEELK (3,4.55,0.47,1)  IDNDGDGFVTTEELK (2,2.46,0.33,1)  EQFNEFR (2,1.57,0.26,89) |
| 3 | galectin 1 (LGALS1) | 8 | LNLEAINYMAADGDFK (2,6.15,0.64,1)  FNAHGDANTIVCNSK (3,4.66,0.32,1)  LPDGYEFKFPNR (2,3.64,0.54,1)  DGGAWGTEQR (2,3.17,0.44,1)  VRGEVAPDAK (2,2.56,0.29,1)  LPDGYEFK (2,2.08,0.37,3)  SFVLNLGK (2,1.86,0.31,2)  GEVAPDAK (2,1.64,0.31,3) |
| 4 | Tubulin specific chaperone A (TBCA) | 8 | ILENEKDLEEAEEYKEAR (3,6.30,0.55,1)  RLEAAYLDLQR (2,4.10,0.51,1)  MRAEDGENYDIKK (3,4.09,0.46,1)  LEAAYLDLQR (2,3.97,0.56,1)  MRAEDGENYDIK (2,3.59,0.40,2)  AEDGENYDIKK (2,3.33,0.28,2)  KQAEILQESR (2,3.14,0.39,1)  LVKEKVMYEK (2,2.91,0.37,1) |
| 5 | ubiquitin conjugating enzyme E2 K (UBE2K) | 9 | LWAHVYAGAPVSSPEYTKK (3,6.34,0.59,1)  LWAHVYAGAPVSSPEYTK (2,5.69,0.66,1)  EVLKSEETSKNQIK (2,3.97,0.46,1)  GEIAGPPDTPYEGGR (2,3.71,0.62,1)  EFKEVLKSEETSK (2,3.44,0.54,1)  IPETYPFNPPK (2,3.42,0.42,1)  NAVIVALSSK (2,2.73,0.29,1)  KIENLCAMGFDR (2,2.72,0.42,1)  QNPEMFKQTAR (3,1.70,0.20,1) |
| 6 | polyamine-modulated factor 1 (PMF1) | 5 | LLDTMVDTFLQK (2,4.07,0.52,1)  HVQKQEAENQQLADAVLAGR (3,3.45,0.36,1)  ELVAVLREPE (2,3.10,0.46,1)  LVAAGSYQR (2,2.75,0.47,1)  RHEGSSSESVPPGTTISR (3,2.3,0.11,72) |
| 7 | Unidentified | - |  |
| 8 | stathmin 1 (STMN1) | 17 | SKESVPEFPLSPPK (2,3.71,0.58,1)  NKESKDPADETEAD (2,3.64,0.58,1)  ESKDPADETEAD (2,3.40,0.65,1)  MEANKENREAQMAAK (3,3.25,0.40,12)  AIEENNNFSK (2,3.14,0.37,1)  MAEEKLTHK (2,3.06,0.42,1)  EKDKHIEEVR (2,2.95,0.44,8)  SHEAEVLK (2,2.91,0.32,2)  KLEAAEERR (2,2.86,0.09,11)  DKHIEEVR (3,2.80,0.39,2)  KLEAAEER (2,2.77,0.15,1)  EAQMAAKLER (2,2.58,0.37,1)  EHEKEVLQK (2,2.54,0.49,8)  KSHEAEVLK (3,2.50,0.20,3)  ESVPEFPLSPPK (2,2.43,0.46,1)  KKDLSLEEIQK (2,2.27,0.18,120)  REHEKEVLQK(4,2.59,0.18,325) |
| 9 | toll interacting protein (TOLLIP) | 8 | LRLGYAVYETPTAHNGAK (3,4.37,0.48,1)  VEDKWYSLSGR (2,3.85,0.16,1)  LGYAVYETPTAHNGAK (2,3.74,0.45,1)  GPVYIGELPQDFLR (2,3.60,0.37,3)  LGYAVYETPTAHNGAKNPR (2,2.94,0.19,1)  ITPTQQQR (2,1.65,0.26,39)  AFSMDDR (2,1.44,0.22,12)  QGKVEDKWYSLSGR (2,1.38,0.22,1) |
| 10 | protein CWC15 homolog (CWC15) | 5 | GKGEGDLSQLSK (2,3.85,0.55,1)  QTTQDAPEEVR (2,3.03,0.45,2)  YRQTTQDAPEEVRNR (3,2.96,0.47,1)  EQEQKAEEERIR (3,2.00,0.35,21)  QTTQDAPEEVRNR (3,1.70,0.34,42) |
| 11 | sorting nexin 9 (SNX9) | 11 | SGNWESSEGWGAQPEGAGAQR (2,6.11,0.68,1)  SSSYFKDSESADAGGAQR (2,5.15,0.48,1)  SYIEYQLTPTNTNR (2,4.19,0.52,1)  VKESDKLVATSK (2,4.04,0.56,1)  PGTEQYLLAK (2,3.94,0.51,1)  ITLQDKQNMVK (2,3.68,0.36,1)  YKHFDWLYER (2,2.34,0.39,1)  FEEEFIK (2,2.34,0.23,3)  FGSAIPIPSLPDKQVTGR (3,2.26,0.40,1)  LQAWMTR (2,2.10,0.11,2)  CEAVGKFTK (3,1.84,0.12,20) |
| 12 | scavenger mRNA-  decapping enzyme (DCPS) | 12 | VNEASGDGDGEDAVVILEK (2,6.35,0.62,1)  ELDVEEAHAASTEEK (2,4.94,0.61,1)  KRELDVEEAHAASTEEK (3,4.54,0.46,1)  SLRDLTPEHLPLLR (3,4.17,0.43,1)  NILHQGQEAILQR (2,4.14,0.09,1)  TTVVYPATEKHLQK (2,3.72,0.57,1)  DLTPEHLPLLR (2,2.53,0.32,1)  LIRETGDDYR (3,2.44,0.23,2)  ADDPLLK (2,2.40,0.19,1)  TTVVYPATEK (2,2.07,0.30,2)  LLQEAQQS (2,2.02,0.35,2)  TLTFALR (2,1.46,0.32,5) |
| 13 | fatty acid binding protein 4 (FABP 4) | 5 | LVSSENFDDYMKEVGVGFATR(2,5.05,0.68,1)  STITLDGGVLVHVQK(2,4.11,0.46,1)  NTEISFILGQEFDEVTADDRK(3,4.02,0.52,1)  LVSSENFDDYMK(2,3.79,0.6,1)  EVGVGFATR(2,2.12,0.44,1) |
| 14 | thioredoxin reductase 1 (TRXR1) | 13 | KLMHQAALLGQALQDSR (2,6.11,0.65,1)  MIEAVQNHIGSLNWGYR (2,5.56,0.55,1)  KVVYENAYGQFIGPHR (2,5.42,0.64,1)  LMHQAALLGQALQDSR (3,4.75,0.43,1)  EKKVVYENAYGQFIGPHR (3,4.63,0.59,1)  VVYENAYGQFIGPHR (2,4.36,0.60,1)  VEETVKHDWDR (2,3.52,0.50,1)  KVMVLDFVTPTPLGTR (3,3.47,0.38,9)  IGEHMEEHGIK (2,3.41,0.47,1)  QFVPIKVEQIEAGTPGR (2,3.40,0.39,1)  KIGLETVGVK (2,3.10,0.33,1)  IGLETVGVK (2,2.65,0.22,1)  LYAGSTVK (2,1.57,0.10,5) |
| 15 | alcohol dehydrogenase [NADP+] (AKR1A1) | 14 | GLEVTAYSPLGSSDR (2,4.23,0.62,1)  VFDFTFSPEEMK (2,3.93,0.48,1)  HHPEDVEPALRK (3,3.63,0.30,2)  AVPREELFVTSK (2,3.17,0.45,1)  YGRSPAQILLR (3,3.00,0.06,72)  HHPEDVEPALR (3,2.82,0.26,4)  SPAQILLR (2,2.79,0.17,1)  YALSVGYR (2,2.67,0.41,1)  DAGHPLYPFNDPY (2,2.55,0.34,1)  ALEALVAK (2,2.48,0.28,1)  WQVQRK (2,2.28,0.03,10)  QLNALNKNWR (2,1.87,0.32,3)  EELFVTSK (2,1.73,0.26,2)  QLNALNK (2,1.28,0.12,72) |
| 16 | N-acetylneuraminate synthase (sialic acid synthase) (NANS) | 7 | GSDHSASLEPGELAELVR (2,5.63,0.59,1)  VGSGDTNNFPYLEK (2,4.84,0.49,1)  HITLDKTWK (3,3.28,0.39,6)  SELEFKFNR (2,2.69,0.34,1)  ALERPYTSK (2,2.61,0.37,3)  GYPPEDIFNLVGKK (2,2.33,0.29,1)  KALERPYTSK (3,1.82,0.16,3) |
| 17 | glutamine synthetase (GS) | 7 | RLTGFHETSNINDFSAGVANR (3,6.35,0.51,1)  LTGFHETSNINDFSAGVANR (2,6.25,0.63,1)  AYGRDIVEAHYR (3,3.52,0.25,1)  RPAETNLR (2,3.05,0.39,1)  RHQYHIR (3,2.77,0.32,1)  DIVEAHYR (2,2.42,0.38,1)  TLDSEPK (2,1.42,0.13,20) |

^a^ The numbers in parentheses correspond to z, Xcorr, dCn, Rsp parameters of TurboSequest respectively. ^b^Matched peptide number: number of peptides matched with protein in MS/MS query.
